# Whole organism transcriptome analysis of zebrafish models of Bardet-Biedl Syndrome and Alström Syndrome provides mechanistic insight into shared and divergent phenotypes

**DOI:** 10.1186/s12864-016-2679-1

**Published:** 2016-05-03

**Authors:** Timothy L. Hostelley, Sukanya Lodh, Norann A. Zaghloul

**Affiliations:** Division of Endocrinology, Diabetes and Nutrition, Department of Medicine, University of Maryland School of Medicine, 660 W. Redwood Street, Howard Hall 487, Baltimore, MD 21201 USA

## Abstract

**Background:**

Bardet-Biedl Syndrome (BBS) and Alström Syndrome are two pleiotropic ciliopathies with significant phenotypic overlap between them across many tissues. Although BBS and Alström genes are necessary for the proper function of primary cilia, their role in defects across multiple organ systems is unclear.

**Methods:**

To provide insight into the pathways underlying BBS and Alström phenotypes, we carried out whole organism transcriptome analysis by RNA sequencing in established zebrafish models of the syndromes.

**Results:**

We analyzed all genes that were significantly differentially expressed and found enrichment of phenotypically significant pathways in both models. These included multiple pathways shared between the two disease models as well as those unique to each model. Notably, we identified significant downregulation of genes in pathways relevant to visual system deficits and obesity in both disorders, consistent with those shared phenotypes. In contrast, neuronal pathways were significantly downregulated only in the BBS model but not in the Alström model. Our observations also suggested an important role for G-protein couple receptor and calcium signaling defects in both models.

**Discussion:**

Pathway network analyses of both models indicate that visual system defects may be driven by genetic mechanisms independent of other phenotypes whereas the majority of other phenotypes are a result of genetic players that contribute to multiple pathways simultaneously. Additionally, examination of genes differentially expressed in opposing directions between the two models suggest a deficit in pancreatic function in the Alström model, that is not present in the BBS model.

**Conclusions:**

These findings provide important novel insight into shared and divergent phenotypes between two similar but distinct genetic syndromes.

**Electronic supplementary material:**

The online version of this article (doi:10.1186/s12864-016-2679-1) contains supplementary material, which is available to authorized users.

## Background

Many Mendelian disorders are pleiotropic, characterized by dysfunction across multiple organ systems. Deciphering the underlying molecular etiology of such diseases can be difficult due to the complexity of interpreting the role of a single gene in multiple disparate cell types. This complexity remains even for syndromes in which the subcellular defect is relatively well understood. For example, the ciliopathies are caused by mutations in genes encoding proteins associated with primary cilia or associated structures. However, the molecular pathways disrupted by loss of function in various ciliopathy genes remain relatively unclear. Two ciliopathies in particular, Bardet-Biedl Syndrome (BBS) and Alström Syndrome represent pleiotropic syndromes with overlapping features, but for which the primary molecular defects underlying those features are not well understood.

Alström Syndrome is a rare autosomal recessive, monogenic disorder caused by mutations in the *ALMS1* gene. The *ALMS1* gene is located on chromosome 2p13 and consists of 23 exons, which encode for a 461-kDa protein [1, 2]. Over 100 different disease-causing mutations have been identified in the *ALMS1* gene, the majority of which are nonsense and frameshift mutations occurring in exons 8, 10, or 16 [1, 2]. The reported prevalence of Alström is less than one per million in the general population, with less than one thousand documented cases. Bardet-Biedl Syndrome (BBS) is also a rare autosomal recessive disorder, but exhibits significant genetic heterogeneity with 20 associated genes identified to date [3] The most common gene mutated in BBS is *BBS1*, located on chromosome 11q13, which accounts for approximately 25 % of BBS cases [4]. The prevalence of BBS is 1 in 160,000 in the general population, but as high as 1 in 13,500 in a few isolated communities [4]. Both Alström and BBS are classified as ciliopathies due to the localization of their proteins to the basal body of the primary cilia and the subsequent ciliary dysfunction that occurs when these proteins are disrupted. ALMS1 may be involved in intracellular trafficking and protein transport from the Golgi to the primary cilium [2]. Several of the BBS proteins, including *BBS1*, form a protein complex called the BBSome, which is involved in the transport of molecules to the cilium and along the ciliary shaft [5, 6].

Given the potential similarities in intracellular localization and function, it is not surprising that many of the hallmark clinical features of Alström and BBS are shared between them. Both are uniquely characterized among the ciliopathies by highly penetrant early-onset obesity [2]. They are also both characterized by progressive neurosensory deficits beginning in the first years of life that typically results in hearing loss in adolescence and blindness by adulthood [1, 2, 4, 7]. They exhibit similar endocrine and reproductive defects, such as hypogonadotropic hypogonadism as well as resulting infertility. Renal disease and respiratory illnesses are also features commonly found in both disorders [1, 4]. Despite these similarities, a number of differences between the disorders are present, particularly in the rates of type 2 diabetes mellitus (T2DM), developmental defects, and cognitive deficits. Alström is characterized by highly penetrant early onset T2DM with 70 % prevalence by the age of 20, while in BBS the prevalence is as low as 2–6 %[2]. Developmental delays are common in BBS. They can also be present in Alström but with much lower penetrance, and are generally global, although it can be specific to certain areas of development. BBS is characterized by polydactyly, which can affect all four limbs or only the upper or lower limbs [4], but polydactyly is not present in Alström. Cognitive deficits present in BBS can vary from mild to severe with many patients requiring special schooling. Development of autism spectrum disorder and psychosis is also present [4]. In contrast, the majority of Alström patients exhibit normal levels of intelligence although mild developmental delay of fine motor skills has been observed, likely as a result of sensory deficits [1].

Due to the high degree of phenotypic overlap between these disorders, they are often mistaken for one another, resulting in misdiagnosis. Here, we set out to characterize the global molecular profiles of each disorder in an effort to determine the underlying etiology distinguishing each and to identify common pathways that may be shared between both. We generated zebrafish models of both disorders by targeted knockdown approaches as previously described [8, 9]. Using whole transcriptome analysis by sequencing of RNA isolated from whole zebrafish larvae, we examined gene expression changes in each model and identified pathways altered as a result. Our findings revealed gene expression signatures that offer insight into the mechanisms driving unique and common features across the disorders. These include potentially common mechanisms underlying obesity and retinal degeneration, hallmarks of both disorders, and suggest genetic mechanisms for susceptibility to cognitive delay in BBS but not Alström. Taken together, these findings shed light onto the molecular etiology of two distinct but similar ciliopathies, providing important insight into the extent of similarity as well as important differences between them.

## Results

### Identification of differentially expressed genes

To identify differentially expressed genes in Alström Syndrome (Alms) and Bardet-Biedl Syndrome (BBS), we generated zebrafish models of either syndrome using splice blocking morpholinos (MOs) targeting either *alms1* or *bbs1* transcripts that were previously validated to suppress protein production without introduction of off-target effects [9]. MOs were injected into wild-type zebrafish embryos. At 48 h post fertilization (hpf) two replicates of RNA, from pools of 20 embryos per treatment as well as control embryos injected with control MO were collected for whole transcriptome sequencing. To fully capture all organ systems that may be impacted by disruption of either *bbs1* or *alms1*, we isolated total RNA from whole zebrafish at 48 hpf, a time point at which most organ systems have developed and begin to be functional. MO-driven knockdown at 48 hpf was verified by western blot (Additional file 1: Figure S1.) RNA from each sample was sequenced to a depth of ~110 million reads with 91–100 million reads aligned to the zebrafish genome. Of these, between 85 and 88 % were mapped to exonic regions.

**Fig. 1:**
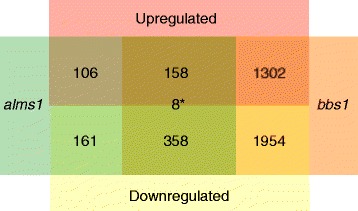
Differentially expressed genes in BBS and Alström models. Numbers of genes upregulated (*red*) or downregulated (*yellow*) in *alms1* MO (*green*) injected embryos, *bbs1* MO (*orange*) injected embryos or both compared to standard control MO injected embryos. * Denotes number of genes changed in both but having opposite changes in expression

A total of 791 genes were significantly differentially expressed in the *alms1* knockdown compared to control. This included 265 that were upregulated and 526 that were downregulated (Fig. 1). Suppression of *bbs1* resulted in a substantially larger set of genes with differential expression levels. A total of 3780 genes were found to have significant changes in expression, with 1467 genes upregulated and 2313 genes downregulated (Fig. 1). To examine the extent of similarities between the two syndrome models, we analyzed the sets of differentially expressed genes and identified overlap between them. 524 genes were differentially expressed in both *alms1* and *bbs1*-depleted larvae, 157 that were upregulated in both and 358 that were downregulated in both (Fig. 1). Interestingly, only 8 genes exhibited changes in gene expression in opposing directions between the two disorders (Fig. 1). We also examined the dataset to identify gene expression changes that were unique to each model. 267 genes were differentially expressed only with loss of *alms1*; 106 were upregulated and 161 downregulated. Loss of *bbs1* resulted in 3256 genes whose gene expression was uniquely changed, 1302 that were upregulated and 1954 that were downregulated. For each model, more genes were found to be downregulated than upregulated, suggesting the widespread negative impact of suppression of each gene. Additionally, almost 5 times more genes were differentially expressed in *bbs1* compared to *alms1*, perhaps indicating dysfunction across a greater number of cell types or pathways in BBS.

### Gene expression changes in BBS model suggest deficiencies in neuronal and visual pathways

**Fig. 2:**
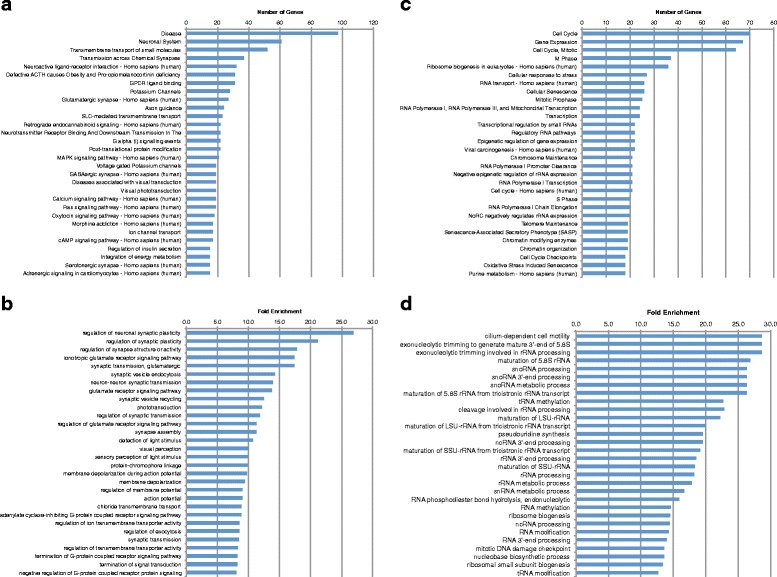
Top up- and downregulated pathways and enriched GO terms in BBS model. **a** Top 30 downregulated pathways by number of genes. **b** Top 30 downregulated GO terms by fold enrichment. **c** Top 30 upregulated pathways by number of genes. **d** Top upregulated GO terms by fold enrichment

Given the large number of differentially expressed genes in each model, we set out to categorize genes to assess their potential relevance to disease phenotypes. To do so and to more clearly elucidate the molecular profile of our BBS zebrafish model, we sought to identify the pathways and gene ontologies enriched in the differentially expressed genes. We queried the ConsensusPathDB to identify enriched pathways in the dataset [10]. Pathways with at least 2 differentially expressed genes were pulled from the Reactome and Kegg databases and a cutoff q-value of <0.05 was used. Enriched gene ontology (GO) terms in the dataset were identified using the enrichment analysis tool of the Gene Ontology Consortium [11]. Biological process GO terms and the *Danio rerio* database were used as parameters with a cutoff p-value of <0.05. The 30 most highly changed, by number of genes or fold enrichment, in each case are shown in Fig. 2.

In total, 92 pathways were downregulated in the BBS model. Aside from the very broad grouping of “disease”, the most highly enriched pathway was the neuronal system, with 61 genes downregulated, accounting for 22.3 % of the genes involved in this pathway (Fig. 2a). This included genes such as *grik1* (−3.74 log fold change, LFC), *kcnv1* (−3.53 LFC), *kcnk3* (−3.42 LFC), and *slc17a7* (−3.35 LFC), all of which were significantly downregulated (Additional file 2: Table S1 and Additional file 3: Table S2). Other highly enriched neuronal pathways included transmission across chemical synapses, which contained 37 downregulated genes, neuroactive ligand-receptor interaction with 32 genes, and axon guidance with 24 genes (Fig. 2a). This also included specific neuronal functional pathways such as Glutamatergic and GABAergic pathways, containing 28 and 21 genes, respectively. In addition, an important neuronal pathway that was highly enriched among the downregulated pathways was defective ACTH and pro-opiomelanocortinin deficiency (POMCD), with 31 downregulated genes (Fig. 2a). Pro-opiomelanocortinin (POMC) is directly related to regulation of satiety and hyperphagia and is known to be disrupted in BBS [12–14]. It is processed through several enzymatic steps to produce melanocyte-stimulating hormone (MSH) and corticotrophin (ACTH) both of which are implicated in other forms of obesity such as melanocortin receptor mutations and Cushing’s [15, 16]. In all, pathways related to neuronal function accounted for approximately one-third of the downregulated pathways.

Perhaps unsurprisingly, some of the most highly enriched pathways were the visual pathways, consistent with the centrality of retinal degeneration to the BBS phenotype. These pathways included visual transduction and visual phototransduction, both with 19 genes downregulated. These included genes required for photoreceptor response, such as rhodopsin, *rho* (−4.67 LFC) and *rhol* (−3.17 LFC), *guca1b* (−4.18 LFC), *rbp1* (−3.84 LFC) and several opsins, *opn1mw2* (−3.74 LFC), *opn1lw2* (−3.36 LFC), *opn1sw2* (−2.98 LFC), and *opn1sw1* (−2.83 LFC).

Several other pathways with general intracellular and signaling roles were also significantly downregulated in the BBS model. For example, the G protein coupled receptor (GPCR) ligand binding pathway was heavily effected with 31 genes downregulated as well as G alpha (i) signaling events with 22 genes, or 10 % of the genes involved in the pathway. This may be consistent with the important link between ciliary localization of GPCRs and the role of BBS proteins in ciliary trafficking or trafficking of GPCRs specifically [17] Calcium signaling was also enriched among the downregulated pathways. 11.4 % (19) of the genes involved in the calcium-signaling pathway were downregulated. The presence of several neuronal and visual pathways is consistent with observed phenotypes of the syndrome. BBS patients are characterized by retinal degeneration and cognitive impairment [2, 18], potentially as a result of disruption of neuronal signaling pathways [17]. In addition, two pathways directly related to energy homeostasis were among the most enriched pathways in downregulated genes. These were related to insulin secretion and integration of energy metabolism. The former may be directly related to glucose homeostasis and evidence suggests that it may be directly perturbed in BBS [19, 20], but both pathways are likely relevant to overall defects in energy metabolism, consistent with the obesity phenotype that is a hallmark of BBS.

169 GO terms were enriched among the downregulated genes (Fig. 2b). The top downregulated GO terms were highly consistent with the top downregulated pathways. The most highly enriched GO terms included several related to the neuronal and visual systems such as: regulation of neuronal synaptic plasticity and regulation of synapse structure or activity, and phototransduction, detection of light stimulus, and visual perception. Several GPCR GO terms were also found, including termination of GPCR signaling pathway and negative regulation of GPCR protein signaling (Fig. 2b).

Although the number of genes upregulated in the BBS model was considerably lower than the number downregulated, these genes encompassed a much greater number of pathways. This is likely due to the smaller number of differentially expressed genes found in each upregulated pathway. In total, 170 pathways were upregulated in the BBS model. The majority of the upregulated pathways are those involved with the cell cycle, including mitosis and the S phase, and those involved with transcription, including several RNA polymerases (Fig. 2c). 159 GO terms were enriched among the upregulated genes and were consistent with the upregulated pathways (Fig. 2d). The majority were involved in RNA processing such as: exonucleolytic trimming involved in rRNA processing, maturation of 5.8S rRNA, tRNA methylation, rRNA processing, and ribosome biogenesis. These observations suggest a previously underappreciated role for BBS genes in regulation of cell cycle events, transcription and translation. Interestingly, the most highly enriched GO term among the upregulated genes is cilium-dependent cell motility consistent with the known roles of BBS genes in ciliogenesis and cilia function [5, 13].

### Analysis of pathways disrupted in Alström model suggests importance of phototransduction signaling

**Fig. 3:**
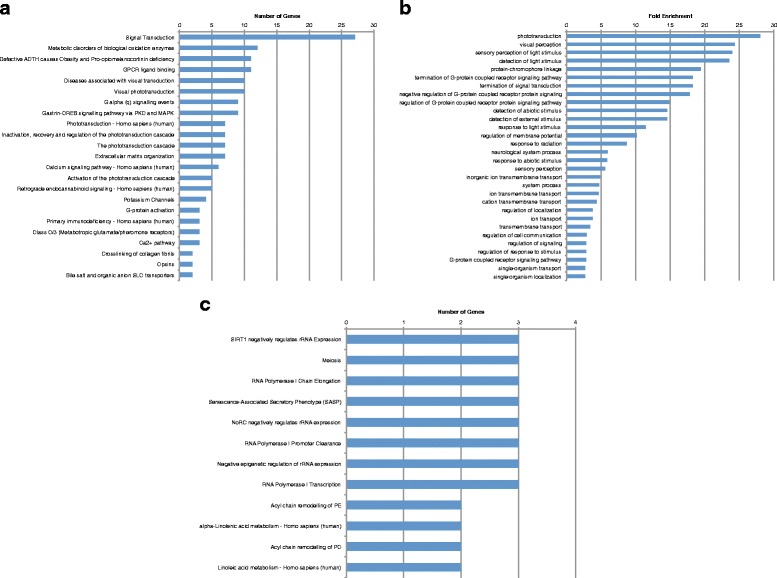
Top up- and downregulated pathways and enriched GO terms in Alström model. **a** Top downregulated pathways by number of genes. **b** Top downregulated GO terms by fold enrichment. **c** Top upregulated pathways by number of genes

We next characterized the gene expression signature, pathways and gene ontologies enriched in larvae targeted for *alms1* expression. The Alström model exhibited fewer enriched pathways, with less genes contained, as well as fewer enriched GO terms, compared to the BBS model. Only 23 pathways were represented in the set of downregulated genes (Fig. 3a) and 47 GO terms (Fig. 3b). There were no significantly upregulated pathways or enriched GO terms in the Alström model.

Similar to the BBS model, many of the downregulated pathways involved the visual pathways including, diseases associated with visual transduction and visual phototransduction. Other enriched visual pathways include: inactivation, recovery and regulation of the phototransduction cascade and the phototransduction cascade, each with 7 downregulated genes, as well as, activation of the phototransduction cascade with 5 of the 12 involved genes (45 %) being downregulated. This is consistent with the retinal degeneration phenotype that is likewise characteristic of Alström [1, 2, 21]. Furthermore, the POMC pathway was also downregulated in *alms1* as it was in *bbs1*, consistent with hyperphagia induced obesity observed in the disorder [1]. Pathways involved with GPCR and calcium signaling were found to be downregulated in *alms1* as they were in *bbs1*. However, in each of the cases mentioned, considerably fewer genes were found in the pathways in *alms1* than were found in *bbs1*.

47 GO terms were enriched among the downregulated genes. Consistent with the downregulated pathways, the most highly enriched GO terms associated with downregulated genes were all involved with the visual system, including phototransduction, visual perception, and detection of light stimulus (Fig. 3b). Several GPCR related GO terms were also highly enriched, such as termination of GPCR signaling pathway and regulation of GPCR protein signaling pathway. Although these terms reflect a high degree of overlap between the BBS and Alström models, the neuronal pathways that were prevalent in *bbs1*-depleted larvae were noticeably absent from both the downregulated pathways and enriched GO terms in *alms1*-deficient animals. This may be consistent with absent or very mild cognitive impairment in Alström patients [1], contrasting with BBS.

### Overlap of differential gene expression between BBS and Alström models

**Fig. 4:**
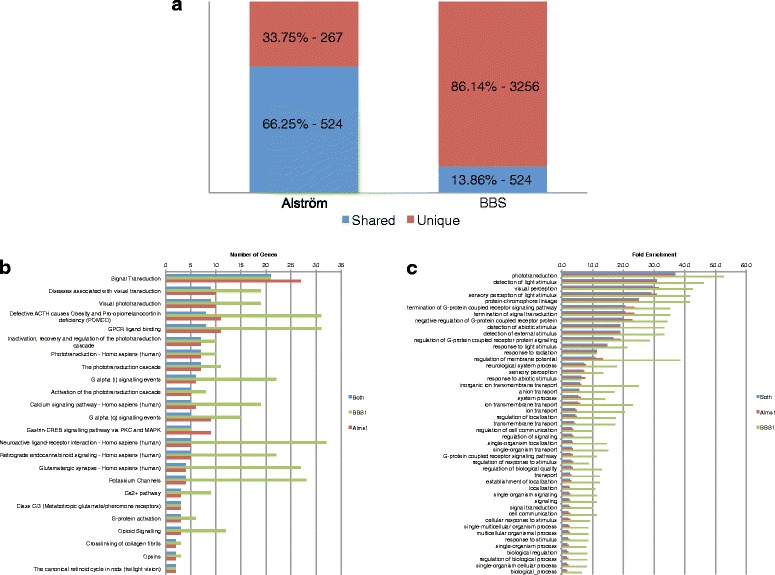
Overlap of differential expression between BBS and Alström models. **a** Percentage and number of differentially expressed genes found unique to each disease model (*red*) and shared across both models (*blue*). **b** Downregulated pathways by number of genes downregulated in both models. **c** Downregulated GO terms by fold enrichment of genes downregulated in both models. Number of genes or GO terms downregulated in the BBS model (*green*), in the Alström model (*red*) or both (*blue*) indicated

The phenotypic overlap between BBS and Alström often results in their being mistaken for one another. Our whole transcriptome approach allowed us to compare the two models at the molecular level to assess areas of molecular overlap that might underlie similar phenotypes and areas of divergence that might inform the uniqueness of each disorder. We first examined the extent of overlap between the two. Animals with reduced *alms1* had a much larger proportion of differentially expressed genes in common with the BBS model (66 %) than genes unique to itself (34 %) (Fig. 4a). In contrast, the BBS model exhibited a more unique spectrum with 86 % of the differentially expressed genes unique to it and only 14 % in common with the Alström model. This suggests that nearly all of the Alström phenotypes may be shared with BBS, whereas features of BBS are unique to it.

We then looked at the pathways and GO terms downregulated in both models to assess overlap. 24 pathways were downregulated in both models (Fig. 4b). In almost every case, the number of genes downregulated in the shared pathways was greater in the BBS model. The majority of genes differentially expressed in *alms1*-deficient animals were also differentially expressed in animals targeted for *bbs1*. Pathways associated with the visual system were the most commonly downregulated pathways in both models. The POMC pathway was also one of the top downregulated pathways found in both. Other pathways that were downregulated in both disorders include those associated with GPCRs and the calcium-signaling pathway.

47 GO terms were enriched among the downregulated genes found in both models. The 30 terms with the greatest enrichment in the downregulated genes found in both are shown in Fig. 4c. The enriched GO terms were consistent with the pathways, again, with the most highly enriched GO terms associated with the visual system including: phototransduction, detection of light stimulus, visual perception, and sensory perception of light stimulus. Several GPCR terms were also found to be downregulated in both, such as termination of GPCR signaling pathway, negative regulation of GPCR protein signaling, and GPCR signaling pathway.

**Fig. 5:**
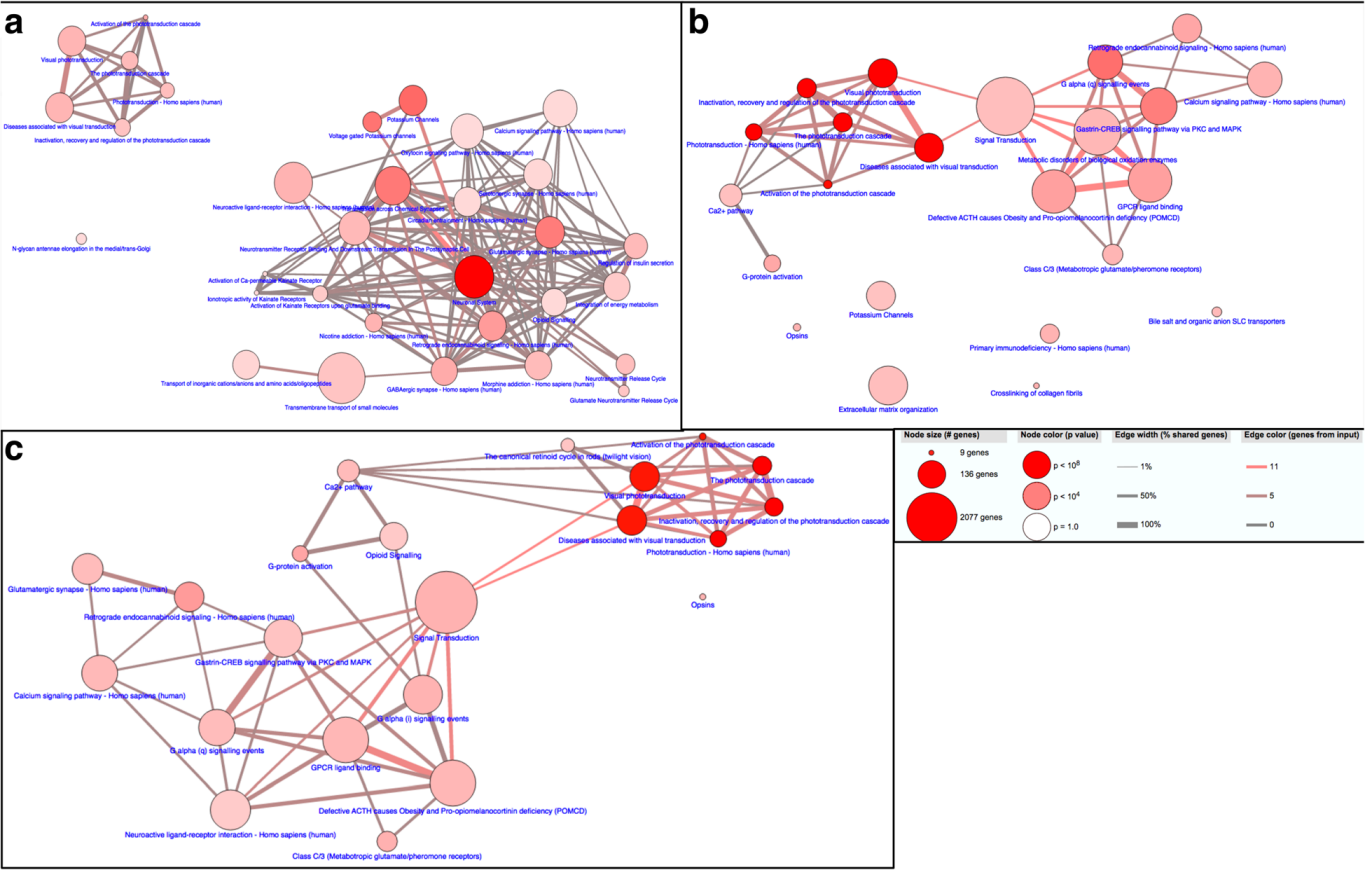
Pathway networks of overlapping genes in BBS and Alström models. Pathway analysis of top 30 downregulated pathways among differentially expressed genes in BBS model (**a**) or in Alström model (**b**). **c** Pathway connectivity of downregulated pathways found in both models. Pathway connections determined by a minimum of 20 % shared genes between pathways and at least 2 genes overlap

To assess the extent to which downregulated pathways may be interconnected we generated pathway networks. For the BBS model, the pathway network revealed two distinct, unconnected, nodes (Fig. 5a). One node encompassed the interconnected visual pathways, while the other incorporated the remainder of the pathways. This larger node reveals the connectivity among the neuronal pathways as well as a high degree of connectivity to the other affected pathways, such as calcium signaling, insulin secretion, and energy metabolism. Given the significantly smaller number of genes and pathways, the Alström model resulted in a simpler, less connected, network (Fig. 5b). In this network we again found that all of the pathways associated with the visual system clustered together with a high degree of interconnectivity between them. However, the visual pathways node was also connected to both the Ca^2+^ pathway and signal transduction. There were also several pathways that showed no overlap with any other pathway including, extracellular matrix organization, potassium channels, and primary immunodeficiency. Finally, to examine the relatedness between the disease models we generated a pathway network for the genes that were downregulated in both models (Fig. 5c). Similar to the Alström model, the visual pathways formed a highly interconnected node, connecting only to the Ca^2+^ pathway and signal transduction. This suggests that overlap between visual signaling and Ca^2+^ or signal transduction may indeed be relevant to both disease models, but perhaps relies on a small subset of genes whose contribution in the BBS model was dwarfed simply by the large number of disrupted genes. The remainder of the shared pathways all showed some degree of overlap among them, with the exception of opsins, although not to the extent that was seen in the BBS model.

Our examination of the overlap between these pathways allowed us to identify genes that exert a greater impact on the spectrum of phenotypes observed in these disorders based on their contribution to a larger number of pathways. For example, downregulation of the G protein genes *gngt1*, *gng7*, and *gng8*, as well as the GABA receptor genes *gabrb2*, *gabrb3*, *gabra1*, *gabra2*, *gabra3*, and *gabrg2* in BBS may be driving the cognitive deficits characteristic of the disorder, as these genes were found in the majority of the downregulated neuronal system pathways. Each of these genes contributed to greater than 20 % of the pathways identified perhaps indicating a broad phenotypic spectrum driven by a relatively small number of genes. Indeed, *gngt1* alone contributed to nearly 60 % of pathways, indicating its potential importance in contributing to multiple phenotypes (Additional file 4: Figure S2A). Additionally, downregulation of rhodopsin *rho*, *gnat1*, *grm1*, *grm5*, *pde6b*, and *opn1mw2* in both Alström and BBS may be major drivers of the retinal degeneration that is characteristic of both disorders, as these genes were found in the majority of the downregulated visual and phototransduction pathways (Additional file 4: Figure S2A-B).

### Gene expression changes provide insight into unique molecular features of BBS

**Fig. 6:**
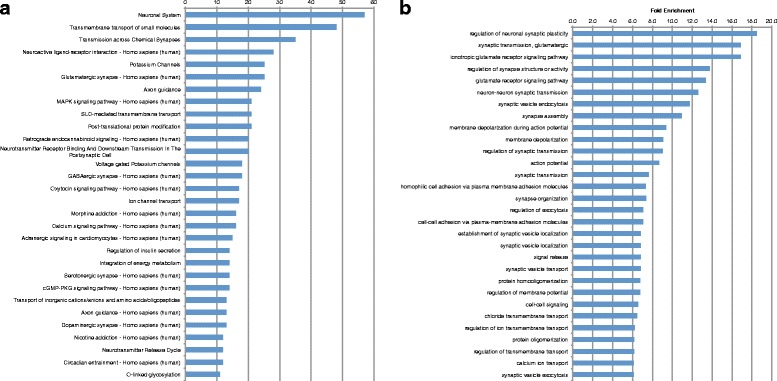
Top downregulated pathways and enriched GO terms unique to the BBS model. **a** Top 30 downregulated pathways by number of genes only differentially expressed in the BBS model. **b** Top 30 downregulated GO terms by fold enrichment of genes only differentially expressed in the BBS model

The majority of the gene expression changes in the BBS model were unique to it, while the majority of gene expression changes in the Alström model were shared with BBS. To assess the molecular profile that distinguishes BBS from Alström, we examined the downregulated pathways and enriched GO terms of the genes that were unique to BBS. 66 pathways were identified among the downregulated genes unique to BBS. The pathways containing the greatest number of genes are shown in Fig. 6a. Many of the most highly downregulated pathways are those related to the neuronal pathways similar to what was seen in the full set of genes downregulated in *bbs1*. However, unlike what was seen in the full set, none of the visual pathways, POMC pathway, or GPCR pathways were found to be downregulated in the gene set unique to *bbs1* indicating that these are the primary areas of overlap with Alström. This also suggests that downregulation of the neuronal system may be a unique characteristic of BBS.

117 GO terms were enriched among the unique to *bbs1* downregulated genes, the GO terms with the greatest enrichment are shown in Fig. 6b. Again, most of the top GO terms were related to the neuronal system, including regulation of neuronal synaptic plasticity, synapse assembly, and synaptic transmission, supporting the evidence that neuronal system defects may be a characteristic marker of BBS that is not shared with Alström. Also absent in the unique enriched GO terms were those related to the visual system and GPCR signaling, similar to the unique downregulated pathways.

### Opposite changes in expression

**Fig. 7:**
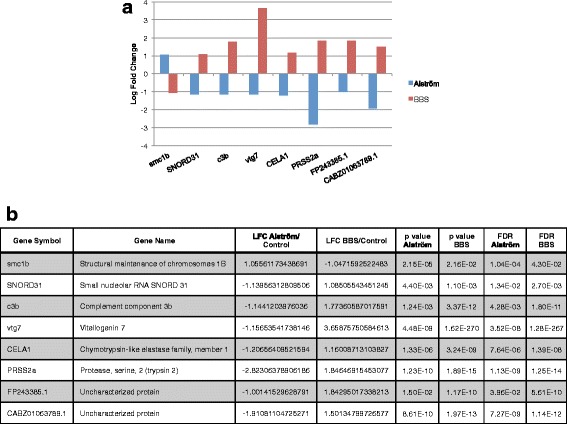
Genes differentially expressed in opposing directions between BBS and Alström models. **a** Log fold change (LFC) of genes differentially expressed in opposite directions between both disease models. **b** Table showing gene names, LFC relative to controls, p-values relative to controls, and false discovery rates (FDR) for 8 genes showing opposing changes in differential expression among BBS and Alström models

Our observations suggested a high degree of overlap between these syndromes. We reasoned, however, that gene expression changes in opposing directions might provide insight into the molecular basis for what differentiates these two distinct syndromes from each other. In total, only 8 genes exhibited expression changes in opposing directions. 2 of these were uncharacterized genes, providing little insight into their function. Only one gene, *smc1b*, was upregulated in the Alström model and downregulated in BBS (Fig. 7) while the remaining 7 genes were all downregulated in Alström but upregulated in BBS. Interestingly, 2 of these 7 genes have relevance to pancreatic function. These include chymotrypsin-like elastase family, member 1, *cela1*, a pancreatic enzyme that is not expressed in the pancreas in human, and protease serine 2 (trypsin 2), *prss2a*, encoding a member of the trypsin family of serine proteases. Chymotrypsin-like elastase family members are the major components of pancreatic elastase, which cleave tropoelastin at one of two conserved hydrophobic domains [22, 23]. *CELA1* has also been linked to chronic pancreatitis, as patients with chronic pancreatitis commonly have very low levels of elastase [24]. *PRSS2A* is an exocrine pancreas protease, secreted by the pancreas and cleaved to its active form in the small intestine for the breakdown of proteins to amino acids. Once activated, *PRSS2A* cleaves peptide linkages involving the carboxyl group of lysine or arginine [25]. It is noteworthy that 2 of the 8 genes that had opposite changes in expression between the disorders are pancreatic enzymes, suggesting that the pancreatic enzymes may play a role in the differences observed between Alström and BBS. To further investigate the relevance of this observation, we examined all downregulated genes in the Alström model to assess potential effects on other pancreatic enzymes. We found 3 other pancreatic proteases that were either downregulated in *alms1*, trypsin (*try*) and chymotrypsinogen B1 (*ctrb1*), or upregulated in *bbs1*, chymotrypsin-like (*ctrl*), suggesting a role for the pancreatic proteases specifically in producing some of the differences between these 2 disorders. To validate these observations we assessed expression of these 5 pancreatic proteases in both models relative to control by qRT-PCR. Expression changes relative to controls exhibited similar trends, with reduced expression in the Alström model and either increased or unchanged in the BBS model (Additional file 5: Figure S3).

## Discussion and conclusions

In this study, we have undertaken a comprehensive transcriptomic approach in models of BBS and Alström syndrome with the goal of identifying genetic pathway mechanisms underlying phenotypes associated with these disorders. Our use of the zebrafish model allowed for assessment of gene expression across the whole organism, an approach that is particularly relevant to multi-organ syndromes. Although our assessments were limited to the window of analysis to embryonic and early larval stages due to the use of transient knockdown to generate the disease models, the nature of these syndromes as developmental disorders suggests that these may be the most relevant time points for onset of most phenotypes associated with disease. Indeed, the majority of the hallmark features of either disorder manifest within the first or second decade of life, consistent with developmental or early childhood onset [1, 2, 4, 7]. Our global analysis revealed important insights into the pathways likely contributing to disease phenotypes. These included known mechanisms, such as the POMC deficiency reported in BBS models to underlie hyperphagia and obesity [13, 14], as well as perhaps novel mechanisms, such as the neuronal pathways identified that may relate to cognitive impairment. Taken together, the results of these analyses offer novel candidate genes and pathways for further exploration in the study of these disorders and related ciliopathies.

Our observations in the BBS model shed light onto the potential role of BBS genes as well as phenotypes driven by their dysfunction. For example, perhaps the most prominent feature of BBS is retinal degeneration characterized by rod-cone dystrophy [4, 19]. Previous reports have attributed this feature to the importance of the photoreceptor primary cilium and trafficking within it [26]. Our findings suggest the specific perturbation of genes in the phototransduction pathway, such as *rho* (−4.67 LFC), *gngt1* (−2.38 LFC), and *pde6g* (−1.80 LFC), or the visual transduction pathway, such as *gnat1* (−3.32 LFC), *cnga1* (−3.64 LFC), and *pde6b* (−1.71 LFC). It is unclear if this disruption is a cause or consequence of other primary factors driving retinal degeneration, but they potentially offer novel factors to examine in models of BBS or in patients. Interestingly, our analysis of the interconnectedness between pathways suggests little overlap between the disrupted vision pathways and neuronal signaling. The exception to this in the BBS model was the presence of the *gngt1* gene, which contributed to both the visual and neuronal pathways. It is unlikely, however, that downregulation of this gene is sufficient for neuronal deficits given the absence of neuronal system effects in the Alström model. This potentially indicates the specificity of the retinal phenotype to that structure, rather than a general consequence of neuronal dysfunction. BBS is characterized by deficits in both functions and it is therefore unsurprising that pathways important to both tissues are perturbed with loss of *bbs1*. In contrast, a higher degree of connectedness was observed between neuronal pathways and energy metabolism regulation, including insulin secretion, potentially implicating neuronal regulation in those defects.

Other pathways disrupted in the BBS model offer novel insight into phenotypes associated with the disorder. For example, a number of basic cellular functions appear to be upregulated in the BBS model. This included cell cycle processes as well as transcriptional and translational regulation. Much evidence supports the role of ciliary proteins in regulation of the cell cycle, given the tight light between ciliogenesis and proliferation [27], but our observations perhaps suggest a more direct role for the BBS genes in regulation of transcription or translation. This may be consistent with the recently proposed role of BBS7 as a transcription factor [28].

**Fig. 8:**
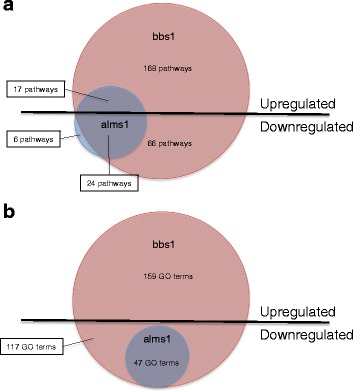
Summary of pathway and GO term analysis results. **a** Pathway analysis summary of upregulated and downregulated enriched pathways found in the BBS model (*red*), the Alström model (*blue*) or both. **b** Gene Ontology (GO) term analysis summary of upregulated and downregulated enriched GO terms found in the BBS model (*red*), the Alström model (*blue*) or both

Because BBS and Alström syndrome are often mistaken for one another we set out to characterize the extent of overlap in differential gene expression between models of both. Surprisingly, the Alström model exhibited far fewer genes that were differentially expressed and the majority of these were shared with BBS. As a result of this, almost all of the pathways and GO terms that are enriched in the Alström model were shared with the BBS model (Fig. 8). In particular, the disruption of vision related pathways and the POMC pathway, as well as GPCR signaling and calcium signaling were common to both models. The relatively fewer disruptions observed in the Alström model may be consistent with the smaller number of phenotypes and organ systems impacted by loss of *alms1*. For example, Alström patients do not typically exhibit cognitive impairment and this was reflected by the noticeable absence of disrupted neuronal signaling in the Alström model. In contrast, the differentially expressed genes in the BBS model were highly enriched in the neuronal pathways, indicative of the centrality of cognitive and perhaps other neuronal defects to the disorder. Similarly, very few genes were upregulated in the Alström model relative to the BBS model. However, similar cellular processes were impacted. These observations might suggest that the ALMS1 protein is perhaps less critical for ciliary function or other basic functions influenced by the basal body to which it and the BBS proteins localize [8, 13, 29–32]. In general, the larger number of genes altered in the BBS model and the larger number of genes contributing to the highly enriched pathways suggests a greater degree of dysfunction than in the Alström model. While these observations offer important insight into the overlap and the discrepancies between the two, shedding light onto unique diagnostic criteria to differentiate between them, they also provide important common mechanisms by which the shared phenotypes are mediated.

Perhaps the most intriguing observation relates to the genes that were differentially regulated in each model but with divergent directionality. Only 8 genes fit this criteria, two of which were uncharacterized and therefore of unknown relevance. Interestingly, however, two of the eight genes were pancreatic proteases downregulated in the Alström model and upregulated in the BBS model. We also identified 3 other pancreatic protease genes that were downregulated in the Alström model but unchanged in the BBS model. Although these enzymes relate specifically to exocrine pancreatic function, they may offer a novel mechanism by which differences in either exocrine or endocrine pancreatic function occur between the two disorders. The prevalence of diabetes in Alström is far higher than that in BBS and is at least in part likely due to deficits in endocrine pancreas function [9]. Our data might suggest a novel interaction between exocrine and endocrine pancreas. Moreover, given the importance of pancreatic enzymes in digestion, these observations might suggest a mechanism by which digestive phenotypes, a secondary feature of Alström [1, 7], might arise.

In summary, our analysis of zebrafish models of two related but distinct syndromes offers novel insight into the mechanisms underlying their phenotypes. These data confirm previously proposed mechanisms of dysfunction for the common phenotypes, such as obesity, while at the same time offering new mechanisms for many features of both syndromes. Importantly, these findings distinguish the two disorders from each other and perhaps suggest novel disruptions of cell types and pathways in each. Finally, these results inform the understanding of how *BBS1*, *ALMS1* and cilia may function at the cellular level. These global gene expression data represent a starting point from which detailed mechanistic links can be explored.

## Methods

### Zebrafish lines

Experiments were carried out in wild type Tubingen (WT) zebrafish. Adult zebrafish were housed and naturally mated according to standard protocol. All zebrafish work was conducted in accordance with University of Maryland IACUC guidelines.

### Morpholinos

Morpholino antisense oligonucleotides (MOs) that block splicing of targeted mRNAs were injected into one- to two- cell stage embryos. We used previously validated MOs to target *bbs1* and *alms1* transcripts [9]. A control non-specific MO was used (5′-CCTCTTACCTCAGTTACAATTTATA-3′). The embryos were grown at 28.5 °C until 48 h post fertilization (hpf) for analyses.

### RNA extraction and sequencing

Groups of 20 embryos were pooled for each condition (control MO, *alms1* MO, *bbs1* MO) at 48hpf for RNA extraction. RNA was extracted using Isol-RNA Lysis Reagent (5 Prime) and purified using Qiagen RNeasy cleanup kit according to manufacturer’s protocol. The three RNA samples were sent in duplicates for sequencing and primary analysis. 2 ug of total RNA per sample was used. Illumina RNAseq libraries were prepared using the TruSeq RNA Sample Prep kit (Illumina, San Diego, CA). Libraries were barcoded and pooled, and each received 0.33 of a lane of sequencing on an Illumina HiSeq 2500 with a paired-end 100 base configuration. Library construction and sequencing was performed at the Institute for Genome Sciences of the University of Maryland, School of Medicine (IGS).

### RNA-sequencing data analysis

Sequenced reads were aligned to the zebrafish genomes (Zv10) using TopHat [33]. Gene expression levels (read counts) were calculated using HTseq [34] based on ENSEMBL gene annotations [35]. The zebrafish datasets were then normalized using reads per kilobase per million reads (RPKM). An average of 28,700 features were detected as expressed in each sample set. Replicate analysis and differentially expressed genes were determined using DEGseq [36]. Both *alms1* and *bbs1* knockdowns were compared to the control, cutoffs of false discovery rate (FDR) <0.05 and p value <0.05 were used to identify genes with significant changes in expression. Primary data analysis was performed at the Institute for Genome Sciences of the University of Maryland, School of Medicine (IGS). Sequence data for samples is publicly available through the NCBI Sequence Read Archive (SRA), Submission ID “University of Maryland BBS Alstrom Zebrafish RNA-Seq”.

### qRT-PCR

RNA was extracted from pools of 20 48hpf embryos using Isol-RNA Lysis Reagent (5 PRIME). The RNA was purified by 3 M sodium acetate and isopropanol precipitation, and spun for 10 min at 4° (12,500 rpm) following a 20 min incubation. The RNA pellet was then washed in 70 % ethanol and re-suspended in RNase free water. cDNA was transcribed using Fermentas First Strand cDNA transcription kit (Thermo Scientific) according to manufacturer’s protocol. cDNA was then diluted 1:3. qRT-PCR was performed using target specific primers and LightCycler 480 SybrGreen (Roche) according to manufacturer’s protocol on a LightCycler 480 machine (Roche). All samples were run in duplicate with CT value normalized to β-actin.

### Western blots

Western blotting was performed according to previously described protocol [9].

### Determination of enriched pathways

Pathways enriched among the genes exhibiting changes in expression were determined by uploading sets of the differentially expressed genes to ConsensusPathDB (http://cpdb.molgen.mpg.de). Genes were identified based on their gene symbol (HGNC symbol). Pathways from the KEGG and Reactome databases were used, along with a q-value cutoff of <0.05 and a minimum of 2 genes overlap with the input list.

### Generation of pathway networks

Pathway networks were generated using the ConsensusPathDB in the same manner as the enriched pathways. Once the enriched pathways were determined, the top 30 most highly significantly enriched pathways were selected for visualization. Pathways were shown as connected if there were at least 2 genes overlapping and the pathways shared at least 20 % of their genes.

### Determination of enriched gene ontologies

Gene ontologies enriched among the differentially expressed genes were determined using the GO Enrichment Analysis tool of the Gene Ontology Consortium (http://geneontology.org/page/go-enrichment-analysis). Gene lists were uploaded to the tool and queried against the biological process *Danio rerio* gene ontology reference database, a cutoff p-value of <0.05 was applied.

## Additional files

Additional file 1: Figure S1. Validation of Morpholinos (MOs) by qrt-PCR and Western blotting. Western blot analysis of 48 hpf zebrafish homogenates detecting the expression of Alms1 (**A**) or Bbs1 (**B**) proteins. (PDF 622 kb) Additional file 2: Table S1. Differentially expressed genes in Alström model. All genes exhibiting significant changes in *alms1*-depleted zebrafish larvae, relative to control. Columns represent: ENSEMBL transcript ID (Feature.ID), gene name (gene_symbol), fold change (FC), p-value relative to control (*p* < 0.05), false discovery rate (FDR; FDR < 0.05). (PDF 159 kb) Additional file 3: Table S2. Differentially expressed genes in BBS model. All genes exhibiting significant changes in *bbs1*-depleted zebrafish larvae, relative to control. Columns represent ENSEMBL transcript ID (Feature.ID), gene name (gene_symbol), fold change (FC), p-value relative to control (*p* < 0.05), false discovery rate (FDR; FDR < 0.05). (PDF 611 kb) Additional file 4: Figure S2. Top genes found in downregulated pathways. Top 15 genes found in the greatest percentage of downregulated pathways in the (**A**) BBS model or the (**B**) Alström model. (PDF 164 kb) Additional file 5: Figure S3. qRT-PCR validation of genes identified to be differentially expressed in opposing directions by RNA-Seq. Relative fold changes in expression of targeted genes in the Alström model (blue) and the BBS model (green) compared to control (red). *indicates *p* < 0.001; **indicates *p* < 0.05 (students *t*-test). (PDF 40 kb)
