# Supplementary figures and images for: Whole organism transcriptome analysis of zebrafish models of Bardet-Biedl Syndrome and Alström Syndrome provides mechanistic insight into shared and divergent phenotypes
Source: BMC Genomics. 2016 May 3;17:318. doi: 10.1186/s12864-016-2679-1 (PMC4855444; doi:10.1186/s12864-016-2679-1)

# Supplementary Figure 1.

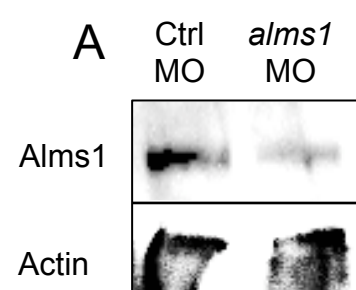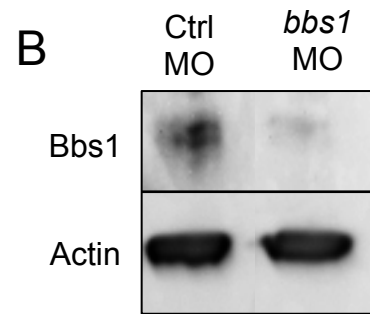

Supplement: Additional file 1: Figure S1. — Validation of Morpholinos (MOs) by qrt-PCR and Western blotting. Western blot analysis of 48 hpf zebrafish homogenates detecting the expression of Alms1 (A) or Bbs1 (B) proteins. (PDF 622 kb) [file 12864_2016_2679_MOESM1_ESM.pdf]

Supplementary Figure 2.

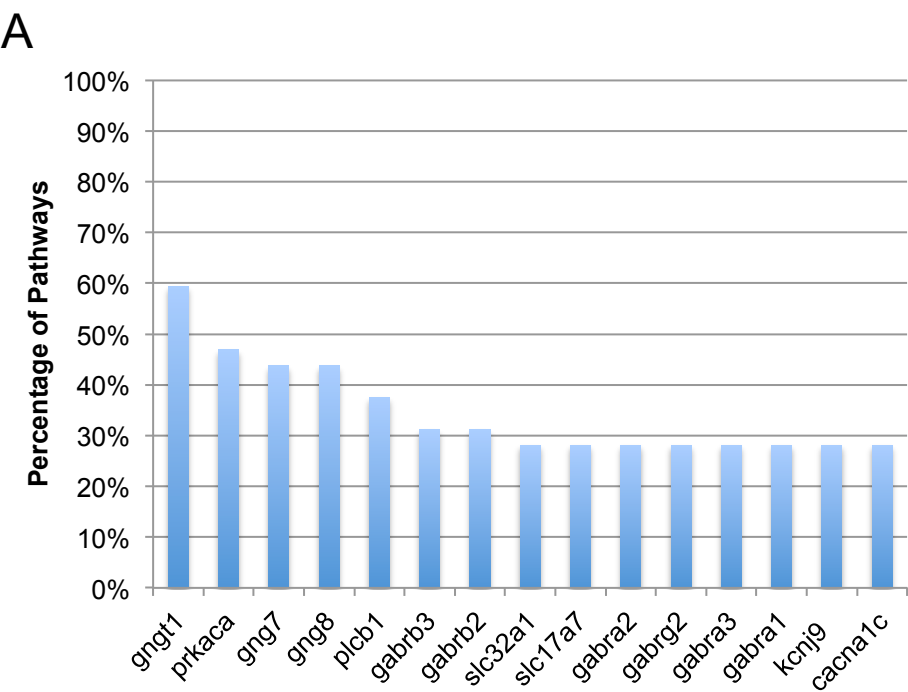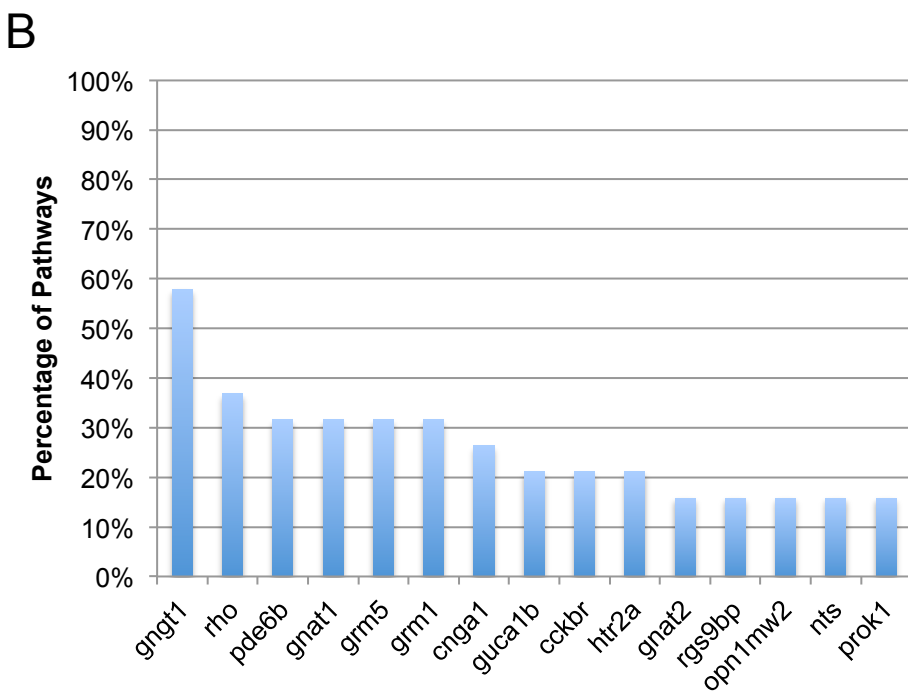

Supplement: Additional file 4: Figure S2. — Top genes found in downregulated pathways. Top 15 genes found in the greatest percentage of downregulated pathways in the (A) BBS model or the (B) Alström model. (PDF 164 kb) [file 12864_2016_2679_MOESM4_ESM.pdf]

Supplementary Figure 3.

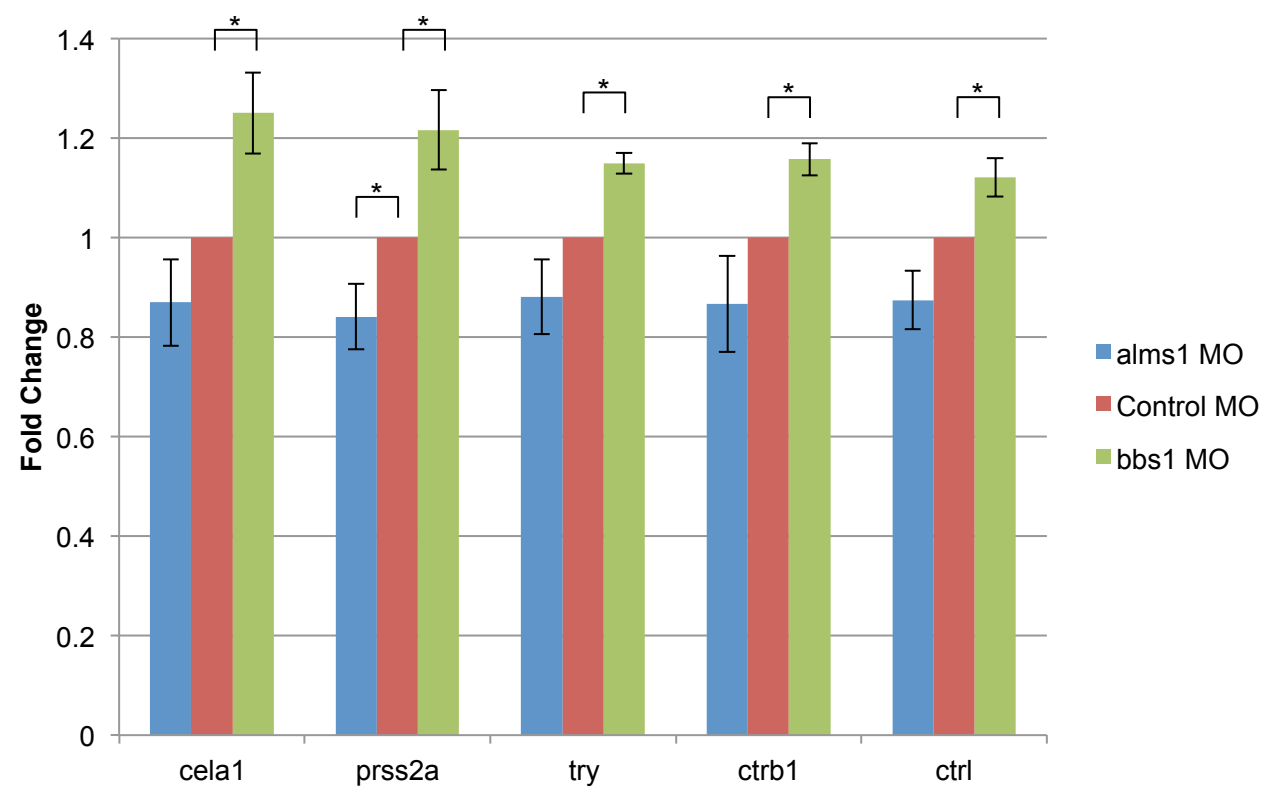

Supplement: Additional file 5: Figure S3. — qRT-PCR validation of genes identified to be differentially expressed in opposing directions by RNA-Seq. Relative fold changes in expression of targeted genes in the Alström model (blue) and the BBS model (green) compared to control (red). *indicates p < 0.001; **indicates p < 0.05 (students t-test). (PDF 40 kb) [file 12864_2016_2679_MOESM5_ESM.pdf]
