# Supplementary material for: Whole organism transcriptome analysis of zebrafish models of Bardet-Biedl Syndrome and Alström Syndrome provides mechanistic insight into shared and divergent phenotypes
Source: BMC Genomics. 2016 May 3;17:318. doi: 10.1186/s12864-016-2679-1 (PMC4855444; doi:10.1186/s12864-016-2679-1)
Supplement: Additional file 2: Table S1. — Differentially expressed genes in Alström model. All genes exhibiting significant changes in alms1-depleted zebrafish larvae, relative to control. Columns represent: ENSEMBL transcript ID (Feature.ID), gene name (gene_symbol), fold change (FC), p-value relative to control (p < 0.05), false discovery rate (FDR; FDR < 0.05). (PDF 159 kb) [file 12864_2016_2679_MOESM2_ESM.pdf]

Supplementary Table 1. Differentially expressed genes in Alstrom model

Feature.ID	gene_symbol	FC	LFC(Alms/Control)	p.Value	FDR
ENSDARG00000094048	si:dkey-224b4.5	115.7397159	6.854740198	2.45E-58	2.92E-56
ENSDARG00000090014	CAPN2 (3 of 4)	102.0821394	6.67358666	1.92E-224	2.09E-221
ENSDARG00000061496	si:dkeyp-98a7.1	42.64106232	5.414171475	2.87E-20	5.60E-19
ENSDARG00000091965	si:dkey-231p15.1	37.9795292	5.247150117	5.57E-27	1.61E-25
ENSDARG00000053512	crybb2	19.4984511	4.28528762	9.10E-09	6.90E-08
ENSDARG00000094508	si:dkey-21o19.8	13.4799287	3.75274096	9.12E-28	2.78E-26
ENSDARG00000052288	CR788231.1	10.57042369	3.4019613	5.75E-22	1.25E-20
ENSDARG00000088533	BX537263.8	10.17675814	3.347206152	8.92E-18	1.48E-16
ENSDARG00000009443	zgc:92137	9.260056803	3.211021043	3.55E-21	7.32E-20
ENSDARG00000094584	si:ch211-92l17.1	8.813295251	3.139681537	1.43E-14	1.87E-13
ENSDARG00000092662	dnaaf3	8.483340861	3.084632531	7.51E-09	5.76E-08
ENSDARG00000091085	lepa	8.142237928	3.02542538	3.26E-07	2.04E-06
ENSDARG00000069481	ghrh	8.065151873	3.011701701	4.58E-21	9.37E-20
ENSDARG00000051775		7.859414822	2.9744219	1.00E-06	5.85E-06
ENSDARG00000093639	si:dkey-90a24.1	7.621478155	2.93007083	2.10E-32	7.91E-31
ENSDARG00000079403	si:dkey-204l11.1	7.084454832	2.824656838	1.54E-35	6.92E-34
ENSDARG00000091735	si:ch211-244h4.1	6.81006154	2.767667835	9.64E-16	1.37E-14
ENSDARG00000073775	VDAC3 (3 of 4)	6.278046115	2.650315626	1.94E-08	1.42E-07
ENSDARG00000097054	si:ch73-65n21.2	6.204826685	2.633390913	5.60E-10	4.83E-09
ENSDARG00000046091	si:ch211-283g2.1	5.844420694	2.54706003	6.52E-12	6.78E-11
ENSDARG00000097929	si:dkey-117j14.6	5.625532493	2.491989663	1.11E-40	6.25E-39
ENSDARG00000088775	NLRP6 (56 of 145)	5.592500717	2.483493536	2.11E-16	3.15E-15
ENSDARG00000036628	cd74b	5.571518247	2.478070518	2.25E-10	2.01E-09
ENSDARG00000095893	si:dkey-85n7.7	5.534822265	2.46853699	2.09E-06	1.17E-05
ENSDARG00000096563	si:dkey-269i1.2	5.424981487	2.439618214	7.46E-07	4.44E-06
ENSDARG00000071662	si:rp71-36a1.3	5.360188857	2.422283832	2.80E-09	2.24E-08
ENSDARG00000091765	PLA2G4C (5 of 5)	5.324679667	2.412694735	4.55E-86	1.08E-83
ENSDARG00000074306	ctslb	5.216334268	2.383036322	8.82E-29	2.80E-27

ENSDARG00000089750	si:dkey-26g8.5	5.186226783	2.374685294	3.58E-24	8.88E-23
ENSDARG00000002295	si:dkey-21p1.3	5.131649904	2.359422749	2.78E-18	4.78E-17
ENSDARG00000089039		4.758108152	2.250388065	1.50E-05	7.43E-05
ENSDARG00000013855	slc12a3	4.732095383	2.242479154	3.14E-43	1.95E-41
ENSDARG00000093898	NLRP6 (116 of 145)	4.63657902	2.213060743	7.28E-17	1.12E-15
ENSDARG00000092002	DHPS (2 of 2)	4.628170252	2.210441936	2.50E-09	2.01E-08
ENSDARG00000079376	zgc:174153	4.589422171	2.198312523	1.90E-47	1.46E-45
ENSDARG00000094559	zgc:174855	4.57194461	2.192807925	1.23E-124	5.47E-122
ENSDARG00000077170	CR394546.2	4.570611047	2.192387053	2.46E-05	0.000117984
ENSDARG00000070212	zgc:158463	4.455931644	2.155727101	4.63E-19	8.35E-18
ENSDARG00000069223	pcdh1g26	4.360337076	2.124439667	1.25E-46	9.32E-45
ENSDARG00000097762	si:dkey-33c14.2	4.2628507	2.091818528	4.21E-08	2.94E-07
ENSDARG00000091566	CR361561.4	4.247681314	2.086675531	1.27E-05	6.35E-05
ENSDARG00000040118	zgc:113232	4.102266919	2.036421366	0	0
ENSDARG00000091768	CR932360.4	4.093591913	2.033367288	5.80E-05	0.000259735
ENSDARG00000095252	si:dkey-40h20.1	4.008668856	2.003123246	5.01E-15	6.78E-14
ENSDARG00000091351	cnp	3.954182475	1.983379449	3.93E-182	2.72E-179
ENSDARG00000090918	si:dkey-269i1.4	3.822894952	1.934665558	7.68E-48	6.02E-46
ENSDARG00000074897	hes2.1	3.808313518	1.929152253	3.89E-50	3.37E-48
ENSDARG00000075527	si:dkey-269i1.3	3.801909363	1.926724139	8.84E-25	2.28E-23
ENSDARG00000095072	si:dkey-26g8.4	3.741717485	1.903700633	8.47E-24	2.05E-22
ENSDARG00000039806	ntlb	3.675718771	1.878026391	1.83E-10	1.66E-09
ENSDARG00000086654	CT027762.3	3.663904433	1.873381874	6.00E-11	5.68E-10
ENSDARG00000088111	CU929417.2	3.663809208	1.873344377	4.62E-19	8.32E-18
ENSDARG00000078557	CU896645.1	3.659185263	1.871522461	8.19E-17	1.25E-15
ENSDARG00000026039	cyp1a	3.638569642	1.863371424	9.76E-194	7.70E-191
ENSDARG00000078237	ZCCHC3	3.560243436	1.831975891	3.36E-05	0.000157359
ENSDARG00000085168	AL935186.3	3.50263277	1.808439738	4.25E-43	2.61E-41
ENSDARG00000086605	si:dkey-8o9.5	3.492499821	1.804260042	9.39E-05	0.000405205
ENSDARG00000090070	CABZ01060490.2	3.472730962	1.796070647	2.34E-09	1.89E-08

ENSDARG00000057504		3.454658864	1.788543256	1.72E-76	3.39E-74
ENSDARG00000074745	CABZ01044297.1	3.452275778	1.787547716	0	0
ENSDARG00000091770	si:ch211-113a14.14	3.448535163	1.785983677	2.83E-06	1.56E-05
ENSDARG00000089397	wu:fe11b02	3.430491358	1.778415232	7.97E-05	0.000348311
ENSDARG00000090354	MLLT4 (2 of 2)	3.416154398	1.772373181	7.97E-05	0.000348326
ENSDARG00000002293	si:ch211-197g15.9	3.397699598	1.764558305	1.88E-10	1.70E-09
ENSDARG00000096603	bmb	3.344338695	1.741720963	2.78E-57	3.14E-55
ENSDARG00000044329	opa1	3.311692364	1.727568661	8.09E-29	2.57E-27
ENSDARG00000094744	zgc:171759	3.299696063	1.722333143	3.12E-06	1.70E-05
ENSDARG00000091205	si:ch211-113a14.19	3.281954844	1.714555389	0.000185435	0.00075896
ENSDARG00000069936	si:dkey-281i8.1	3.268164489	1.708480597	7.44E-51	6.66E-49
ENSDARG00000031483	col9a1	3.225651465	1.689590562	0	0
ENSDARG00000074069	zgc:171452	3.181672484	1.669785335	2.91E-10	2.58E-09
ENSDARG00000058891	nedd9	3.175846135	1.667141018	1.32E-27	3.95E-26
ENSDARG00000023656	he1a	3.168761773	1.663919202	1.77E-239	2.10E-236
ENSDARG00000078962	DUOXA1	3.166206757	1.662755468	5.09E-10	4.40E-09
ENSDARG00000095529	gusb	3.1623727	1.661007406	2.65E-24	6.67E-23
ENSDARG00000094439	tmem176l.3a	3.147271454	1.654101617	2.22E-18	3.84E-17
ENSDARG00000089021	si:dkey-7f16.3	3.14590962	1.653477224	4.04E-05	0.000186349
ENSDARG00000089806	si:dkey-239j18.3	3.132909263	1.647502986	1.10E-192	8.42E-190
ENSDARG00000068941	HIST2H3C	3.123542014	1.643182935	1.59E-06	9.06E-06
ENSDARG00000078299	COLGALT2 (2 of 2)	3.105058891	1.634620631	2.99E-20	5.82E-19
ENSDARG00000061564	nlrc3	3.098803144	1.631711108	1.60E-06	9.09E-06
ENSDARG00000088623	CR388079.2	3.089758672	1.62749416	5.02E-06	2.67E-05
ENSDARG00000058815	ihhb	3.08214762	1.623935962	1.05E-08	7.88E-08
ENSDARG00000070265		3.072481758	1.619404445	0.000769325	0.002779953
ENSDARG00000095744	si:dkey-269i1.4	3.066257173	1.616478704	7.40E-11	6.93E-10
ENSDARG00000088845	CABZ01085177.1	3.064053613	1.615441541	2.37E-06	1.32E-05
ENSDARG00000061480		3.034092611	1.601265122	7.47E-06	3.87E-05
ENSDARG00000033999	cyp26a1	3.005847591	1.58777186	8.48E-219	8.30E-216

ENSDARG00000071368	si:dkey-86k10.3	2.978998544	1.574827418	9.21E-06	4.71E-05
ENSDARG00000078674	hspb9	2.960703657	1.565940095	4.50E-114	1.85E-111
ENSDARG00000089456	men1	2.952328765	1.561853386	5.60E-41	3.18E-39
ENSDARG00000095866	fabp1b.2	2.948314232	1.559890295	0.000525529	0.001966075
ENSDARG00000087004	si:ch211-122l24.4	2.942425482	1.557005879	1.13E-08	8.47E-08
ENSDARG00000007344	tcap	2.931734115	1.551754268	1.62E-96	4.95E-94
ENSDARG00000089064	CR848728.2	2.926279303	1.549067476	7.22E-08	4.90E-07
ENSDARG00000098037	si:ch73-65n21.1	2.923768854	1.54782926	0.000241291	0.000964893
ENSDARG00000090572	si:busm1-180g5.7	2.91684047	1.544406483	0.000433442	0.001652716
ENSDARG00000092241	si:dkey-240n22.7	2.910877775	1.541454263	7.38E-23	1.70E-21
ENSDARG00000096322	si:ch73-379f7.4	2.894535607	1.533331904	5.16E-05	0.000233102
ENSDARG00000075538	zgc:163061	2.882502488	1.527321853	1.66E-11	1.66E-10
ENSDARG00000055527	cmn	2.876914815	1.524522504	0	0
ENSDARG00000089836	MRPS12 (2 of 2)	2.855994914	1.51399341	2.22E-57	2.53E-55
ENSDARG00000094311	SUSD1 (2 of 2)	2.847925014	1.509911161	1.03E-10	9.50E-10
ENSDARG00000019742	tlr4ba	2.841824491	1.506817458	2.08E-06	1.16E-05
ENSDARG00000087934	CABZ01080435.1	2.814514936	1.492886304	7.94E-17	1.22E-15
ENSDARG00000001937	CABZ01043017.1	2.807146108	1.489104156	6.54E-14	8.07E-13
ENSDARG00000086515	rad21a	2.803006523	1.486975101	3.87E-301	5.78E-298
ENSDARG00000086709	si:ch211-113a14.14	2.800403752	1.485634845	0.001150789	0.00399902
ENSDARG00000089031	larsa	2.792370379	1.481490313	0.000937434	0.003323938
ENSDARG00000077218	SLC22A17 (1 of 2)	2.79047269	1.480509527	3.26E-09	2.59E-08
ENSDARG00000032640	PDXK (1 of 3)	2.78812731	1.479296439	2.26E-35	1.01E-33
ENSDARG00000084432	5S_rRNA	2.770151828	1.469965051	0.002073212	0.006800654
ENSDARG00000088726		2.76865943	1.4691876	8.84E-08	5.95E-07
ENSDARG00000087315	CT956064.2	2.764236461	1.466881033	0.000938943	0.003328871
ENSDARG00000086076	HIST1H4D	2.747230182	1.457977794	0.000766257	0.002770981
ENSDARG00000088321		2.745602981	1.457123024	5.58E-09	4.34E-08
ENSDARG00000097785	hist2h3c	2.744255413	1.456414762	0.000766165	0.002770981
ENSDARG00000091905	si:dkey-108k21.21	2.742861021	1.455681524	1.64E-05	8.09E-05

ENSDARG00000097752	si:rp71-77l1.2	2.715561172	1.441250363	0.000133011	0.000559896
ENSDARG00000054321	ngs	2.715231889	1.441075414	1.16E-279	1.57E-276
ENSDARG00000075330	phox2ba	2.683641228	1.424191813	0.000627558	0.002308528
ENSDARG00000074491	zgc:171759	2.683134476	1.423919363	1.52E-09	1.25E-08
ENSDARG00000009905	ntla	2.671684061	1.417749413	2.70E-54	2.78E-52
ENSDARG00000097560	zgc:162999	2.654948754	1.408684015	0.000157039	0.00065119
ENSDARG00000045071	chad	2.653768314	1.408042423	2.20E-140	1.12E-137
ENSDARG00000078334	zgc:174938	2.639944826	1.400507778	1.92E-17	3.09E-16
ENSDARG00000092665	si:ch1073-382c16.2	2.622608134	1.391002259	9.18E-63	1.25E-60
ENSDARG00000079384	CCDC134 (2 of 2)	2.62207015	1.390706284	1.24E-09	1.03E-08
ENSDARG00000055160	chadla	2.61091853	1.384557441	3.26E-44	2.16E-42
ENSDARG00000091587	CABZ01076758.1	2.602855114	1.380095007	4.36E-09	3.44E-08
ENSDARG00000079002	dnai1	2.599085652	1.378004178	0.001306169	0.004474332
ENSDARG00000088906	CABZ01059404.1	2.582066741	1.368526292	2.46E-07	1.57E-06
ENSDARG00000038805	pigp	2.543048375	1.346558906	8.55E-26	2.33E-24
ENSDARG00000007329	tbx16	2.531366619	1.339916469	0.000141701	0.00059322
ENSDARG00000039547	zgc:171759	2.526730009	1.337271515	0.004123698	0.012608751
ENSDARG00000053799	BX855590.1	2.525360579	1.336489395	3.18E-06	1.74E-05
ENSDARG00000090531	ttc25	2.51763985	1.33207192	6.67E-05	0.000295885
ENSDARG00000008948	PLA2G3 (1 of 2)	2.513217076	1.329535287	5.45E-05	0.000245114
ENSDARG00000085949	AL935186.4	2.500505577	1.322219823	4.37E-50	3.77E-48
ENSDARG00000091491	BX511078.2	2.498224822	1.320903315	0.000374386	0.001444361
ENSDARG00000087157	cant1b	2.487453377	1.314669485	1.01E-14	1.35E-13
ENSDARG00000092215	BX901907.1	2.47089471	1.305033536	3.61E-16	5.31E-15
ENSDARG00000091707	CABZ01059421.1	2.470631168	1.304879652	3.46E-06	1.88E-05
ENSDARG00000046140	zgc:112038	2.470485934	1.304794842	0.000163289	0.000674936
ENSDARG00000093721	si:dkey-238o14.8	2.470258559	1.304662055	0.000806314	0.002899583
ENSDARG00000090745	NLRP6 (81 of 145)	2.46838561	1.303567789	0.000807586	0.002903789
ENSDARG00000010591	foxn4	2.463052172	1.300447187	2.23E-221	2.35E-218
ENSDARG00000087917	BX649341.1	2.446175663	1.290528009	0.000653718	0.002396994

ENSDARG00000091245	dnajc7	2.444758291	1.289691836	3.05E-101	1.02E-98
ENSDARG00000092148	GPBAR1 (2 of 2)	2.440224862	1.287014095	0.000348773	0.001354064
ENSDARG00000092910	si:dkey-264f17.4	2.431601301	1.281906696	0.005892884	0.017269132
ENSDARG00000004763	HHLA2 (1 of 4)	2.42158144	1.275949523	4.94E-11	4.70E-10
ENSDARG00000088053	PCDHGC5 (16 of 31)	2.421051791	1.275633942	4.78E-05	0.000217361
ENSDARG00000045634	lmod2a	2.418722032	1.274244979	0.001141179	0.003968539
ENSDARG00000086239	CR847893.1	2.417126652	1.273293069	0.004729149	0.014224028
ENSDARG00000097488	UNC80 (2 of 2)	2.411165455	1.269730652	3.90E-07	2.42E-06
ENSDARG00000085592	5S_rRNA	2.398143031	1.261917707	6.66E-05	0.00029547
ENSDARG00000018765	pknox1.1	2.394475598	1.259709733	2.64E-43	1.65E-41
ENSDARG00000090889	BX005069.4	2.393512263	1.259129198	0.002464879	0.007961212
ENSDARG00000088951	AL935186.6	2.384143373	1.253470996	0	0
ENSDARG00000086374	isg15	2.382846263	1.252685875	0.000261556	0.001039341
ENSDARG00000097036	si:dkey-86k10.5	2.377974824	1.249733441	0.001603525	0.005400386
ENSDARG00000056210	si:ch211-199o1.2	2.374957492	1.247901692	1.61E-26	4.51E-25
ENSDARG00000015512	aqp8b	2.373979445	1.247307443	1.50E-17	2.43E-16
ENSDARG00000045011	TAPBP (1 of 2)	2.369902983	1.244828001	0.000212402	0.000857952
ENSDARG00000088303	NLRP6 (49 of 145)	2.363994974	1.241226968	2.45E-07	1.56E-06
ENSDARG00000008333	znfl2a	2.346171072	1.230308212	1.49E-14	1.95E-13
ENSDARG00000089644	ints12	2.345092973	1.229645121	4.59E-13	5.28E-12
ENSDARG00000086785		2.332070568	1.221611445	3.32E-05	0.00015557
ENSDARG00000078878	METTL21C (2 of 2)	2.324317721	1.21680729	8.43E-11	7.85E-10
ENSDARG00000077017	AL935044.2	2.315721035	1.211461468	1.61E-08	1.18E-07
ENSDARG00000096489	si:dkey-227h16.2	2.315419057	1.211273324	1.39E-91	3.69E-89
ENSDARG00000019122	he1b	2.310907227	1.208459343	3.15E-80	6.78E-78
ENSDARG00000057010	si:dkey-154b15.1	2.301346667	1.202478323	1.78E-11	1.76E-10
ENSDARG00000088277	susd5	2.29200845	1.196612363	1.79E-13	2.14E-12
ENSDARG00000089812	CABZ01017733.1	2.278418454	1.188032737	0.000177948	0.000730742
ENSDARG00000058738	SLC26A6 (3 of 4)	2.264262917	1.179041488	3.30E-07	2.06E-06
ENSDARG00000068168	hes2.2	2.260672996	1.176752323	2.01E-68	3.20E-66

ENSDARG00000076237	CU302319.2	2.259122339	1.175762399	0.000458198	0.001735916
ENSDARG00000091667	si:dkey-234i14.3	2.257018182	1.174418041	4.68E-05	0.000213167
ENSDARG00000090977	ifit5	2.255104065	1.173194011	0.000458089	0.001735738
ENSDARG00000079486	fam20a	2.25161973	1.170963195	5.23E-19	9.40E-18
ENSDARG00000088436	CT956064.3	2.251451353	1.170855306	4.66E-207	4.01E-204
ENSDARG00000076461	pcdh1g18	2.241821243	1.164671246	0.005378736	0.015909757
ENSDARG00000060211	ankef1b	2.234182272	1.159746891	0.001820209	0.006053349
ENSDARG00000089263	CU915826.1	2.224023581	1.153172085	0.000510121	0.001914741
ENSDARG00000001767	slc29a2	2.219345607	1.150134349	1.47E-105	5.08E-103
ENSDARG00000086957	si:dkeyp-118a3.2	2.218837153	1.149803787	4.16E-25	1.08E-23
ENSDARG00000005959	echdc1	2.21516341	1.147413129	1.25E-05	6.24E-05
ENSDARG00000068589	CABZ01079764.1	2.214396274	1.14691342	4.83E-155	2.80E-152
ENSDARG00000095163	ZBED1 (10 of 10)	2.208316324	1.142946842	0.000179901	0.000738226
ENSDARG00000014594	anxa1b	2.206818939	1.141968267	2.02E-109	7.55E-107
ENSDARG00000089875	si:ch211-226o13.2	2.206079498	1.14148478	1.07E-22	2.44E-21
ENSDARG00000074191	zgc:172253	2.205435953	1.141063865	1.25E-05	6.25E-05
ENSDARG00000090297	ldlrad2	2.194184926	1.133685121	2.15E-16	3.21E-15
ENSDARG00000085067	AL935186.2	2.192620194	1.13265593	1.10E-197	8.90E-195
ENSDARG00000078579	zgc:175280	2.192319081	1.13245779	4.52E-12	4.79E-11
ENSDARG00000076521	MMP21	2.189522644	1.13061637	0.002496241	0.008048767
ENSDARG00000077570	CDH16	2.184333066	1.127192854	0.005952365	0.017424909
ENSDARG00000004328	CABZ01041200.1	2.18288472	1.126235942	3.44E-05	0.000160802
ENSDARG00000090557	MFAP4 (6 of 14)	2.179009849	1.123672717	1.29E-17	2.11E-16
ENSDARG00000010332	zgc:56231	2.178732229	1.123488897	0.00021916	0.000883148
ENSDARG00000043993	KCNIP2 (1 of 2)	2.177694308	1.122801451	8.72E-05	0.000379123
ENSDARG00000092583	si:dkey-16p19.5	2.170366052	1.117938387	1.87E-05	9.12E-05
ENSDARG00000090113	fabp11a	2.162257099	1.112538074	1.48E-83	3.45E-81
ENSDARG00000070788	DUPD1 (7 of 12)	2.152739318	1.10617363	0.000410444	0.001571569
ENSDARG00000092933	cbx8a	2.147132143	1.102410983	3.18E-09	2.53E-08
ENSDARG00000091988	si:dkey-164n2.1	2.146221886	1.101799236	7.02E-05	0.000309408

ENSDARG00000091063	FANCM (3 of 3)	2.146209791	1.101791106	0.000196	0.000798518
ENSDARG00000076554	cdkn1a	2.141027352	1.098303226	2.08E-34	8.86E-33
ENSDARG00000091219	CABZ01078767.1	2.140222742	1.097760952	0.000410972	0.001573167
ENSDARG00000079957	CABZ01066734.1	2.139502181	1.097275149	0.006534681	0.018922708
ENSDARG00000069252	bon	2.133867382	1.093470517	0.000241712	0.000966303
ENSDARG00000043518	si:dkey-239i20.2	2.131995965	1.092204708	8.27E-06	4.25E-05
ENSDARG00000086751	zgc:112234	2.131932899	1.092162031	0.002228226	0.007266312
ENSDARG00000070242	ENDOD1 (5 of 13)	2.125192501	1.087593527	0.000215288	0.000868497
ENSDARG00000091348	rtn3	2.123789391	1.086640706	6.68E-72	1.16E-69
ENSDARG00000054649	rac1l	2.122218176	1.085572981	8.08E-06	4.16E-05
ENSDARG00000097148	si:ch73-21b16.1	2.120806078	1.08461271	0.012663306	0.034063238
ENSDARG00000074260	si:ch211-113a14.19	2.119227128	1.083538216	0.012672198	0.034080696
ENSDARG00000042145	lhx8b	2.11741069	1.08230112	0.006554436	0.01897604
ENSDARG00000096310	TAF1C	2.11623741	1.081501485	4.31E-35	1.90E-33
ENSDARG00000092569	si:dkey-73n10.7p	2.113890008	1.079900311	3.04E-05	0.00014338
ENSDARG00000016227	CU571323.1	2.112650872	1.079054373	0.000493166	0.001855025
ENSDARG00000096063	si:dkey-191j3.1	2.108053762	1.075911661	0.012681991	0.034092208
ENSDARG00000073747	si:dkey-1j5.4	2.103201351	1.072586974	2.32E-18	3.99E-17
ENSDARG00000022020	FOCAD	2.101668006	1.071534789	3.55E-65	5.15E-63
ENSDARG00000068453	NLRP6 (4 of 145)	2.098309686	1.069227618	0.000846971	0.003031581
ENSDARG00000012609	hpx	2.09688592	1.068248374	0.000537292	0.002006379
ENSDARG00000095377	si:ch211-28e16.4	2.092226732	1.065039203	0.000191594	0.000782475
ENSDARG00000056374	noxo1b	2.090838938	1.064081932	7.26E-06	3.77E-05
ENSDARG00000095528	si:dkey-121n8.5	2.089072299	1.062862423	1.30E-07	8.58E-07
ENSDARG00000070770	her4.3	2.089052907	1.06284903	7.04E-56	7.57E-54
ENSDARG00000079603	si:ch211-256e16.6	2.088683981	1.062594228	0.000235337	0.000943077
ENSDARG00000091744	BX296557.7	2.082814991	1.058534695	7.81E-203	6.52E-200
ENSDARG00000078262	PCDHGC4 (8 of 9)	2.079458711	1.056208039	2.24E-50	1.96E-48
ENSDARG00000096128	si:dkey-233e3.5	2.078815862	1.055761972	0.000538682	0.002011043
ENSDARG00000014402	smc1b	2.078599392	1.055611734	2.15E-05	0.000103865

ENSDARG00000041141	cryba1a	2.073883244	1.052334675	2.87E-07	1.81E-06
ENSDARG00000078432	ZACN (1 of 2)	2.071122057	1.050412578	6.30E-07	3.79E-06
ENSDARG00000090248	BX649497.1	2.069928988	1.049581275	0.000315249	0.00123404
ENSDARG00000089831	NLRP6 (70 of 145)	2.062384957	1.044313646	2.64E-11	2.57E-10
ENSDARG00000020978	dhrs13a.2	2.052317348	1.037253831	5.17E-77	1.04E-74
ENSDARG00000075366	si:ch211-113a14.10	2.05046136	1.035948557	0.017253652	0.044789434
ENSDARG00000097721	si:dkey-234i14.21	2.046623227	1.033245534	0.004076869	0.01248864
ENSDARG00000097716	si:dkey-86k10.8	2.046479744	1.033144387	0.002886814	0.009155232
ENSDARG00000008022	kif18a	2.046137275	1.032902938	1.53E-72	2.69E-70
ENSDARG00000070945	zgc:163083	2.044860653	1.032002534	0.017256735	0.04479334
ENSDARG00000088717	C6H2orf40 (2 of 2)	2.044390886	1.031671065	3.42E-29	1.11E-27
ENSDARG00000077716	CMYA5 (2 of 2)	2.043813627	1.031263645	3.93E-16	5.78E-15
ENSDARG00000089863	CCDC57	2.043643705	1.031143695	1.98E-32	7.45E-31
ENSDARG00000086561	tk1	2.043219423	1.030844144	4.53E-59	5.54E-57
ENSDARG00000090419	CABZ01078499.2	2.041204557	1.029420768	0.003569173	0.011064592
ENSDARG00000089709	GFRAL	2.035952467	1.02570388	0.000200557	0.00081486
ENSDARG00000034423	sncga	2.035707475	1.025530266	1.40E-113	5.66E-111
ENSDARG00000046098	ebp	2.034529927	1.024695502	1.24E-19	2.33E-18
ENSDARG00000090491	Metazoa_SRP	2.03294572	1.023571696	0.009681859	0.026800477
ENSDARG00000044671	wfikkn1	2.025417821	1.018219551	0.012027923	0.032505054
ENSDARG00000088392	si:dkey-238o14.9	2.021187946	1.015203481	0.002212001	0.007219206
ENSDARG00000077129	notch1a	2.021027529	1.015088973	7.81E-79	1.64E-76
ENSDARG00000077862	si:dkey-169i5.4	2.011609337	1.008350155	0.000491159	0.001848457
ENSDARG00000090493	BX005380.2	2.011156286	1.008025197	0.007299506	0.020873381
ENSDARG00000035560	capga	2.006670576	1.004803797	0.012975019	0.034786337
ENSDARG00000086668	si:dkey-7j22.4	2.002636595	1.001900649	0.000205733	0.000833385
ENSDARG00000089347		2.001667954	1.001202673	3.17E-43	1.96E-41
ENSDARG00000087258	ypel2b	0.499650676	-1.001008289	0.002473764	0.007983549
ENSDARG00000075985	FP243385.1	0.499509736	-1.001415296	0.01500211	0.039563308
ENSDARG00000038185	gh1	0.499458696	-1.001562718	5.69E-10	4.90E-09

ENSDARG00000060051	slc47a2	0.499117504	-1.002548596	6.98E-06	3.63E-05
ENSDARG00000071735	prlh2	0.498423108	-1.004557139	0.000386532	0.001488848
ENSDARG00000015134	CAMKK1 (1 of 2)	0.498366452	-1.004721139	0.000446774	0.001698305
ENSDARG00000074839	impg1b	0.497847685	-1.006223674	1.17E-13	1.41E-12
ENSDARG00000097039	si:dkey-199l17.8	0.497266601	-1.00790856	8.96E-06	4.59E-05
ENSDARG00000054055	ostn	0.496778595	-1.009325081	0.001234617	0.004254386
ENSDARG00000091614	CR848032.2	0.496727655	-1.009473026	6.57E-05	0.000291856
ENSDARG00000092534	si:ch211-62a1.3	0.496174547	-1.011080366	3.59E-05	0.00016732
ENSDARG00000062606	si:dkey-26i13.8	0.495861805	-1.011989993	4.94E-06	2.63E-05
ENSDARG00000054184	btr06	0.495796072	-1.012181252	0.003461195	0.010761591
ENSDARG00000044632	myo7ab	0.495568476	-1.012843676	1.75E-24	4.44E-23
ENSDARG00000087801		0.495459446	-1.013161119	0.013030977	0.034919868
ENSDARG00000077128	galnt13	0.495373438	-1.013411582	3.19E-25	8.35E-24
ENSDARG00000088300	VSTM2A (2 of 2)	0.494934544	-1.014690355	0.009963569	0.027478231
ENSDARG00000036433	ERP27	0.493667133	-1.018389496	4.16E-05	0.000191425
ENSDARG00000098044	si:ch211-136a13.3	0.493128083	-1.019965681	3.42E-08	2.42E-07
ENSDARG00000039626	nrgna	0.49297229	-1.020421541	5.19E-21	1.06E-19
ENSDARG00000091459		0.492632173	-1.021417244	0.000137584	0.000577689
ENSDARG00000092920	si:ch211-106h4.12	0.492538738	-1.021690899	1.39E-06	7.98E-06
ENSDARG00000097157	si:ch211-207n23.2	0.491708359	-1.024125214	1.16E-18	2.04E-17
ENSDARG00000034667	CABZ01090041.1	0.491620184	-1.024383947	2.85E-06	1.57E-05
ENSDARG00000090126		0.490552167	-1.027521528	4.91E-05	0.000222887
ENSDARG00000094702	si:dkey-61n16.5	0.490463102	-1.02778349	3.15E-07	1.98E-06
ENSDARG00000010244	rpl22l1	0.4897806	-1.029792464	4.06E-208	3.60E-205
ENSDARG00000002644	rgs5a	0.489698047	-1.030035653	1.98E-57	2.26E-55
ENSDARG00000052700	si:dkey-162b23.4	0.489508487	-1.030594222	2.77E-96	8.35E-94
ENSDARG00000095045	ZC3H7B (3 of 3)	0.489183769	-1.031551557	0.008903419	0.0248637
ENSDARG00000037359	CDR2 (2 of 2)	0.488930298	-1.032299285	0.000389018	0.001497409
ENSDARG00000005332	lipi	0.488914843	-1.032344889	0.000958593	0.003392607
ENSDARG00000087295	ZFYVE9 (3 of 3)	0.488914843	-1.032344889	0.000958593	0.003392607

ENSDARG00000090560	mfap5	0.488633641	-1.033174904	0.000597833	0.002208916
ENSDARG00000080804	5S_rRNA	0.488584811	-1.03331908	0.002396231	0.007761569
ENSDARG00000097576	VAMP1 (2 of 2)	0.488446545	-1.033727412	1.38E-11	1.39E-10
ENSDARG00000063475	abcg1	0.488140181	-1.034632585	5.43E-11	5.16E-10
ENSDARG00000037588	bhlhe23	0.488128397	-1.034667411	1.96E-58	2.34E-56
ENSDARG00000074666	CA4 (4 of 5)	0.487057246	-1.037836745	0.000335251	0.001305319
ENSDARG00000062902	TULP2	0.486684502	-1.03894126	3.37E-22	7.40E-21
ENSDARG00000092142	si:ch211-136a13.2	0.486629594	-1.039104036	7.47E-06	3.87E-05
ENSDARG00000044276	spp1	0.486603063	-1.039182693	3.11E-05	0.000146555
ENSDARG00000056084	igsf21b	0.486479819	-1.039548136	1.18E-24	3.03E-23
ENSDARG00000027153	gabrr3a	0.486416776	-1.039735109	5.47E-09	4.26E-08
ENSDARG00000092694	si:ch211-106j21.2	0.486243117	-1.040250268	0.011199807	0.030508189
ENSDARG00000069117	kcnh5b	0.486013396	-1.040932015	1.28E-06	7.39E-06
ENSDARG00000009311	pvalb6	0.485966875	-1.041070116	3.86E-32	1.43E-30
ENSDARG00000040781	zgc:113317	0.485531054	-1.042364523	4.62E-32	1.71E-30
ENSDARG00000097855	si:ch211-3o3.9	0.485360679	-1.042870861	1.20E-34	5.18E-33
ENSDARG00000009466	rgs9bp	0.484947414	-1.04409978	3.02E-06	1.65E-05
ENSDARG00000087224	si:ch73-380n15.2	0.484618937	-1.045077315	0.018565678	0.047715339
ENSDARG00000091573	CU693477.1	0.484522322	-1.045364963	0.002398411	0.007766856
ENSDARG00000058462	zgc:158846	0.482939483	-1.050085678	9.73E-39	5.01E-37
ENSDARG00000057887	nts	0.482627399	-1.051018275	0.004349593	0.013205567
ENSDARG00000041073	si:dkey-12e7.4	0.481377091	-1.05476061	1.66E-09	1.36E-08
ENSDARG00000057498	habp2	0.481175867	-1.055363808	9.51E-23	2.18E-21
ENSDARG00000042526	sebox	0.480650317	-1.056940409	0.000289056	0.001138576
ENSDARG00000075513	ccdc136b	0.480430147	-1.057601412	5.64E-11	5.35E-10
ENSDARG00000056248	wu:fb15e04	0.480004675	-1.058879637	1.77E-276	2.29E-273
ENSDARG00000075067	AIPL1	0.479994009	-1.058911697	9.64E-08	6.45E-07
ENSDARG00000020084	tg	0.479806604	-1.05947508	3.88E-28	1.19E-26
ENSDARG00000041883	MPP7 (2 of 2)	0.47972691	-1.059714727	0.004871282	0.014591798
ENSDARG00000071364	si:ch73-329n5.1	0.479712389	-1.059758397	2.15E-07	1.38E-06

ENSDARG00000052289	CYGB (2 of 3)	0.479370223	-1.0607878	3.65E-05	0.000169654
ENSDARG00000069186	cyp27a1.2	0.4791135	-1.061560629	8.76E-06	4.49E-05
ENSDARG00000090686	SLC35F2 (2 of 2)	0.477876833	-1.065289265	0.003343598	0.010429087
ENSDARG00000039535	dapp1	0.477739595	-1.065703642	6.09E-07	3.67E-06
ENSDARG00000040732	elavl2	0.477419647	-1.066670156	0.015849374	0.041558303
ENSDARG00000095822	si:dkey-176g4.5	0.477166559	-1.067435157	0.000113285	0.000482395
ENSDARG00000097544	si:rp71-1h20.9	0.476613005	-1.069109777	0.000125545	0.000530071
ENSDARG00000062467	zgc:162946	0.476297086	-1.070066374	1.29E-06	7.42E-06
ENSDARG00000079986	si:ch211-250k18.8	0.475287107	-1.07312883	0.004170243	0.012726392
ENSDARG00000040928	CABZ01059391.1	0.474791148	-1.074635056	0.012616444	0.03396293
ENSDARG00000076848	LGALS3BP (3 of 3)	0.474746675	-1.0747702	3.65E-14	4.63E-13
ENSDARG00000056909		0.474633444	-1.075114336	7.89E-05	0.000344842
ENSDARG00000019063	FAT1 (2 of 2)	0.474051225	-1.076885133	1.42E-55	1.51E-53
ENSDARG00000051892	KCNG4 (1 of 2)	0.47241979	-1.081858693	0.000336896	0.001311181
ENSDARG00000084681	5S_rRNA	0.472351388	-1.082067597	0.004655028	0.014033808
ENSDARG00000089181	ADAD1	0.472207423	-1.082507373	0.009564715	0.026524097
ENSDARG00000074075	antxr1b	0.471785949	-1.083795643	5.49E-05	0.000247049
ENSDARG00000006341	tas1r3	0.471197963	-1.085594792	0.014155451	0.037581921
ENSDARG00000059399	CR318593.1	0.47102992	-1.086109393	0.005203313	0.015466629
ENSDARG00000075159	mfi2	0.470844221	-1.086678273	4.81E-20	9.25E-19
ENSDARG00000094908	lingo4a	0.469772728	-1.089965132	0.000875415	0.003125506
ENSDARG00000092276		0.469728379	-1.090101338	1.58E-12	1.74E-11
ENSDARG00000058103	glra4b	0.469311954	-1.091380888	2.25E-08	1.63E-07
ENSDARG00000088061	si:dkeyp-72e1.9	0.468143462	-1.094977386	1.43E-20	2.84E-19
ENSDARG00000010680	gngt2a	0.467272758	-1.097663165	5.37E-24	1.32E-22
ENSDARG00000067815	LINGO3 (2 of 2)	0.466609195	-1.099713358	3.81E-06	2.05E-05
ENSDARG00000032801	grk5	0.466436632	-1.100246999	1.49E-19	2.77E-18
ENSDARG00000089101	plrdgb	0.465816534	-1.102166248	3.46E-06	1.88E-05
ENSDARG00000095512	rca2.2	0.465522053	-1.10307858	0.002301883	0.007485868
ENSDARG00000074570	FP016199.1	0.464627117	-1.105854738	2.21E-07	1.42E-06

ENSDARG00000014233	sept8b	0.46433251	-1.1067698	2.00E-17	3.21E-16
ENSDARG00000022739	rxfp3.2a	0.463694885	-1.108752281	0.000233764	0.00093717
ENSDARG00000090366	zgc:171626	0.462735713	-1.111739648	0.002413195	0.007809386
ENSDARG00000057029	htr2a	0.462369319	-1.112882426	8.84E-07	5.21E-06
ENSDARG00000093753	si:ch211-133l5.5	0.461640413	-1.11515857	6.71E-10	5.74E-09
ENSDARG00000011929	plp1b	0.460791814	-1.117813008	1.29E-09	1.07E-08
ENSDARG00000088912	si:ch211-117l17.6	0.460080287	-1.120042451	3.26E-07	2.04E-06
ENSDARG00000069758	RBM44	0.459306824	-1.122469878	0.000145969	0.000609383
ENSDARG00000093224	si:ch211-157h7.1	0.458985485	-1.123479565	0.000197215	0.000802748
ENSDARG00000078970	il7r	0.45874141	-1.124246951	0.006485913	0.01879491
ENSDARG00000070078	abcb11b	0.458379509	-1.125385544	6.20E-07	3.73E-06
ENSDARG00000029112	gem	0.458207345	-1.125927511	5.98E-14	7.40E-13
ENSDARG00000021369	mc3r	0.457686923	-1.127567024	0.007238547	0.020724118
ENSDARG00000043847	tmem244	0.457311066	-1.128752264	2.29E-09	1.85E-08
ENSDARG00000087407	si:ch73-304f21.1	0.457280863	-1.128847549	5.91E-11	5.61E-10
ENSDARG00000078411	hspb15	0.457132438	-1.129315899	1.85E-12	2.03E-11
ENSDARG00000087873	si:ch211-170n20.3	0.455987857	-1.132932688	1.15E-57	1.33E-55
ENSDARG00000041086	CABZ01071177.1	0.455883201	-1.133263848	0.001646459	0.005532508
ENSDARG00000021573	slc16a7	0.455607522	-1.134136529	0.000149698	0.000623117
ENSDARG00000080531	SNORD31	0.453897004	-1.139563128	0.004404174	0.013355563
ENSDARG00000054543	samsn1a	0.453483689	-1.140877434	9.39E-54	9.45E-52
ENSDARG00000078276	COLGALT1 (3 of 3)	0.453428008	-1.141054586	2.51E-80	5.43E-78
ENSDARG00000070960	si:ch211-288g17.4	0.452966576	-1.142523495	0.005441785	0.016064442
ENSDARG00000075929		0.452558114	-1.143825032	0.00331201	0.010337376
ENSDARG00000087359	c3b	0.45246547	-1.144120398	0.001242756	0.004278795
ENSDARG00000074601	cbln2a	0.451838574	-1.146120655	4.05E-05	0.000186537
ENSDARG00000004358	gnb3a	0.450798179	-1.149446408	8.30E-189	6.04E-186
ENSDARG00000095217	MYH13 (10 of 11)	0.450730022	-1.149664547	4.00E-34	1.67E-32
ENSDARG00000090729	and4	0.45036407	-1.150836361	1.99E-67	3.02E-65
ENSDARG00000076824	CCKBR (2 of 2)	0.450332126	-1.150938693	0.002025843	0.00666221

ENSDARG00000016213	BX088711.1	0.450148852	-1.151525955	4.22E-08	2.94E-07
ENSDARG00000055053	CR558302.3	0.449698791	-1.15296909	2.40E-15	3.33E-14
ENSDARG00000023151	ucp1	0.449666735	-1.153071934	2.78E-177	1.80E-174
ENSDARG00000091607	ARHGEF28 (2 of 2)	0.449572449	-1.153374467	5.32E-05	0.000239747
ENSDARG00000019949	serpinh1b	0.449413456	-1.153884772	0	0
ENSDARG00000092419	vtg7	0.448588513	-1.156535417	4.48E-09	3.52E-08
ENSDARG00000062712	si:dkey-236e20.7	0.447946887	-1.158600411	1.91E-06	1.08E-05
ENSDARG00000030750	gabrr3b	0.446464039	-1.163384118	1.32E-08	9.79E-08
ENSDARG00000005966	slc4a5	0.44644021	-1.16346112	4.54E-12	4.80E-11
ENSDARG00000041414	bmf2	0.445159686	-1.167605149	2.29E-17	3.67E-16
ENSDARG00000092792	zgc:92658	0.445112759	-1.167757239	2.60E-09	2.10E-08
ENSDARG00000039964	fgfbp2a	0.444568748	-1.169521562	8.71E-20	1.65E-18
ENSDARG00000037646	rgs11	0.444480581	-1.169807705	2.46E-21	5.13E-20
ENSDARG00000088737	BX005423.3	0.44447275	-1.169833123	0.006051287	0.017681668
ENSDARG00000032553	egln3	0.443106289	-1.174275292	2.35E-43	1.48E-41
ENSDARG00000041382	si:dkey-283b15.2	0.443062658	-1.174417356	1.23E-48	9.96E-47
ENSDARG00000086603	FUT9 (15 of 16)	0.442974341	-1.174704962	0.002248834	0.007329303
ENSDARG00000087963	BX927130.1	0.442490818	-1.176280575	5.41E-09	4.21E-08
ENSDARG00000094095	si:ch211-149k23.10	0.442215212	-1.177179441	0.00225157	0.007336536
ENSDARG00000095831	si:ch211-175f12.2	0.441673233	-1.178948693	9.25E-09	7.01E-08
ENSDARG00000079302	and2	0.441143208	-1.180681023	0	0
ENSDARG00000092574	si:ch211-198d23.1	0.440442532	-1.182974303	0.000244657	0.000976428
ENSDARG00000079703	si:dkey-18p12.4	0.440209697	-1.183737171	3.23E-08	2.29E-07
ENSDARG00000094147	si:rp71-7l19.6	0.4400703	-1.184194085	0.007461317	0.021288906
ENSDARG00000058410	acbd5b	0.440064355	-1.184213577	5.94E-05	0.00026577
ENSDARG00000027799	ucmaa	0.439909775	-1.184720437	0.004521254	0.013679915
ENSDARG00000014805	fhl5	0.439762717	-1.185202796	4.87E-07	2.98E-06
ENSDARG00000068961	APEX2 (2 of 2)	0.438803963	-1.18835154	0.001686607	0.005654696
ENSDARG00000027495	elovl4b	0.438219531	-1.190274311	2.74E-110	1.07E-107
ENSDARG00000084796	5S_rRNA	0.43814802	-1.190509756	0.000296055	0.001163881

ENSDARG00000071657	si:dkey-19a16.4	0.436922792	-1.194549729	7.82E-05	0.000342218
ENSDARG00000092823	si:dkeyp-67a8.4	0.435856021	-1.198076457	0.00168821	0.0056594
ENSDARG00000012504	rlbp1a	0.434696881	-1.201918352	8.99E-89	2.26E-86
ENSDARG00000086969	CR774179.1	0.434418312	-1.202843178	7.72E-07	4.59E-06
ENSDARG00000074772	ccl-c11b	0.434349154	-1.203072866	3.74E-15	5.10E-14
ENSDARG00000076706	CABZ01038708.1	0.433963276	-1.204355136	0.004534715	0.013717717
ENSDARG00000090676		0.433803489	-1.20488644	0.007485725	0.021343528
ENSDARG00000017314	CELA1 (1 of 7)	0.433299328	-1.206564095	1.33E-06	7.64E-06
ENSDARG00000012126	zgc:109965	0.431676504	-1.211977525	2.24E-50	1.96E-48
ENSDARG00000087905	SLC39A8 (2 of 2)	0.430725398	-1.215159699	0.004540439	0.013732106
ENSDARG00000052582	si:ch211-131e11.14	0.430463562	-1.216036975	0.00125926	0.004330889
ENSDARG00000037507	kctd12b	0.430070707	-1.217354224	1.75E-10	1.59E-09
ENSDARG00000095002	TNNC2 (2 of 2)	0.42966348	-1.218720936	5.56E-17	8.63E-16
ENSDARG00000026855	cacna2d4a	0.429176447	-1.220357192	2.04E-18	3.53E-17
ENSDARG00000078647	samsn1b	0.427475103	-1.226087697	0.005012517	0.014974768
ENSDARG00000002576	cabp5a	0.426473993	-1.229470328	0.002057255	0.006756899
ENSDARG00000087551	BX323793.2	0.425797245	-1.231761481	3.33E-09	2.65E-08
ENSDARG00000086640		0.425744431	-1.231940437	0.000222478	0.00089572
ENSDARG00000075837	si:dkey-1d7.3	0.425686114	-1.232138066	1.62E-21	3.40E-20
ENSDARG00000045442	cpb1	0.425096678	-1.234137111	1.34E-21	2.84E-20
ENSDARG00000088491	CABZ01103847.1	0.424774458	-1.235231076	2.32E-05	0.000111633
ENSDARG00000056411	adipoqb	0.424021	-1.237792377	0.001388236	0.004730902
ENSDARG00000088432	si:dkey-162h11.3	0.423934686	-1.238086085	1.79E-17	2.90E-16
ENSDARG00000089319	CU464087.2	0.423103057	-1.240918984	0.000243462	0.000972476
ENSDARG00000056910	NFASC (1 of 2)	0.422875317	-1.241695741	6.15E-21	1.25E-19
ENSDARG00000090526	PLEKHS1 (3 of 4)	0.422797028	-1.241962859	1.13E-31	4.07E-30
ENSDARG00000029898	CNGA1 (2 of 2)	0.422341454	-1.243518237	3.27E-06	1.78E-05
ENSDARG00000076900	PROZ (2 of 2)	0.422055983	-1.244493718	0.000243627	0.000972999
ENSDARG00000088075	GRAMD3	0.421510188	-1.246360593	6.66E-09	5.14E-08
ENSDARG00000076852	CABZ01113374.2	0.421085319	-1.247815516	3.06E-15	4.20E-14

ENSDARG00000055463	lrit3a	0.420859595	-1.248589087	3.86E-06	2.08E-05
ENSDARG00000076820	xkr8.2	0.420385095	-1.250216577	1.54E-13	1.85E-12
ENSDARG00000036186	mbpa	0.420117159	-1.251136385	1.39E-27	4.15E-26
ENSDARG00000093518	si:ch73-186j5.4	0.419936967	-1.251755302	1.89E-07	1.22E-06
ENSDARG00000028343	zgc:101560	0.419653114	-1.252730809	2.67E-06	1.47E-05
ENSDARG00000026990	klhl6	0.419407131	-1.253576703	4.44E-05	0.000203206
ENSDARG00000092151	si:dkey-40i22.5	0.418301674	-1.257384323	0.000244456	0.000975762
ENSDARG00000091739	GPC6 (3 of 3)	0.416956397	-1.262031573	7.71E-05	0.000337862
ENSDARG00000052016	cabp2a	0.416751954	-1.26273913	1.54E-07	1.00E-06
ENSDARG00000091428		0.416659962	-1.263057619	2.43E-09	1.96E-08
ENSDARG00000079347	zgc:194659	0.415564449	-1.266855857	6.23E-17	9.63E-16
ENSDARG00000071709	CT971495.1	0.415234344	-1.26800232	6.78E-12	7.05E-11
ENSDARG00000090130	LRIF1 (2 of 2)	0.413911191	-1.27260684	3.37E-21	6.96E-20
ENSDARG00000071319	narf	0.413728267	-1.273244565	3.81E-43	2.35E-41
ENSDARG00000095595	si:ch73-272h24.1	0.413695162	-1.273360009	3.25E-07	2.03E-06
ENSDARG00000051981	STX3 (2 of 2)	0.412820213	-1.276414482	1.68E-51	1.54E-49
ENSDARG00000078241	MAP7 (2 of 2)	0.412597807	-1.277191943	1.27E-06	7.32E-06
ENSDARG00000062906	kcnv2b	0.411891784	-1.279662745	9.15E-11	8.50E-10
ENSDARG00000021065	camk2b2	0.41080244	-1.283483343	8.07E-09	6.16E-08
ENSDARG00000094533	si:dkey-246e1.3	0.410530557	-1.284438486	9.16E-05	0.000396108
ENSDARG00000087810	CABZ01088935.1	0.409644644	-1.287555144	0.000146301	0.000610499
ENSDARG00000023181	pcp4l1	0.409042494	-1.289677369	6.43E-40	3.50E-38
ENSDARG00000074683	CABZ01048960.1	0.408452451	-1.291759955	2.53E-06	1.40E-05
ENSDARG00000079190	sycp2l	0.407597705	-1.294782167	0.004497278	0.013613176
ENSDARG00000094132	igf1	0.407563327	-1.294903852	5.24E-14	6.51E-13
ENSDARG00000075270	si:ch211-233g6.8	0.407265277	-1.295959278	2.15E-104	7.25E-102
ENSDARG00000004445	GRM5 (1 of 2)	0.406309245	-1.299349905	1.04E-13	1.26E-12
ENSDARG00000053746	OSBPL1A (2 of 2)	0.4059925	-1.300475018	2.01E-12	2.19E-11
ENSDARG00000026248	slc1a7b	0.405877219	-1.300884729	2.54E-06	1.40E-05
ENSDARG00000057262		0.405437952	-1.302446951	1.12E-12	1.25E-11

ENSDARG00000086615	si:dkey-196n19.1	0.404745106	-1.304914458	0.000831737	0.002984571
ENSDARG00000024032	coch	0.403758739	-1.308434607	5.31E-12	5.57E-11
ENSDARG00000070511	CR933820.2	0.403427262	-1.309619516	3.24E-22	7.14E-21
ENSDARG00000002898	DGKA (1 of 2)	0.40293059	-1.311396757	1.44E-10	1.32E-09
ENSDARG00000042993	try	0.402833521	-1.311744355	8.80E-27	2.51E-25
ENSDARG00000073820	zgc:174917	0.401367325	-1.317004921	7.84E-15	1.05E-13
ENSDARG00000094766	CCBL2 (4 of 4)	0.40128635	-1.317296012	6.80E-05	0.000301249
ENSDARG00000052057	PCOLCE (1 of 2)	0.401263126	-1.317379508	4.47E-39	2.35E-37
ENSDARG00000014039	TDRD15	0.401112658	-1.317920599	1.51E-09	1.24E-08
ENSDARG00000043003	pcyt2	0.401090427	-1.31800056	3.84E-225	4.36E-222
ENSDARG00000037390	gsg1l	0.400993347	-1.318349794	1.30E-06	7.46E-06
ENSDARG00000091024	pparab	0.400826487	-1.318950249	0.000172764	0.000710583
ENSDARG00000042722	blnk	0.400470329	-1.320232739	0.000561018	0.002088111
ENSDARG00000057678	sfrp1b	0.400062118	-1.321704069	7.74E-30	2.59E-28
ENSDARG00000069827	crygm2d11	0.399712406	-1.322965744	8.36E-159	4.94E-156
ENSDARG00000086669	edn3b	0.397328967	-1.331594119	0.004939412	0.014779705
ENSDARG00000091492	LOXL2 (3 of 3)	0.397208361	-1.332032103	7.56E-52	7.08E-50
ENSDARG00000060350	APOD (3 of 3)	0.395825119	-1.337064925	1.10E-31	3.98E-30
ENSDARG00000086564	AL935184.1	0.395802404	-1.337147719	9.87E-17	1.50E-15
ENSDARG00000007024	uox	0.395777869	-1.337237154	3.69E-29	1.19E-27
ENSDARG00000010958	glmnb	0.395649819	-1.337703997	4.09E-31	1.44E-29
ENSDARG00000027852	plekhf1	0.395563468	-1.3380189	2.23E-45	1.56E-43
ENSDARG00000071076	ldhbb	0.394322836	-1.342550831	1.66E-26	4.63E-25
ENSDARG00000059416	EGFLAM	0.394153127	-1.343171873	1.72E-19	3.18E-18
ENSDARG00000032838	si:dkey-206f10.1	0.393504806	-1.345546839	5.10E-20	9.79E-19
ENSDARG00000030215	matn1	0.39220258	-1.350329067	0	0
ENSDARG00000091137	CABZ01079258.1	0.391614254	-1.352494817	5.84E-05	0.000261449
ENSDARG00000077708	snx8b	0.391140301	-1.354241904	6.30E-05	0.000280433
ENSDARG00000070724	chrna4b	0.390893358	-1.355153023	0.00018838	0.000769903
ENSDARG00000016480	slc17a7	0.390298978	-1.357348411	1.46E-20	2.89E-19

ENSDARG00000044973		0.390018015	-1.358387332	0.000446022	0.001696352
ENSDARG00000026017	xpnpep2	0.389451732	-1.360483561	7.92E-11	7.39E-10
ENSDARG00000069017	elnb	0.389404433	-1.360658786	2.43E-73	4.40E-71
ENSDARG00000097930	si:ch211-161c3.6	0.389369667	-1.360787596	1.17E-44	7.93E-43
ENSDARG00000093608	si:dkey-25o1.6	0.389307532	-1.361017839	8.76E-12	8.99E-11
ENSDARG00000078617	CABZ01048958.1	0.389152457	-1.361592627	1.09E-07	7.25E-07
ENSDARG00000087431	zgc:173962	0.388823196	-1.362813806	2.55E-06	1.41E-05
ENSDARG00000062077	acsbg1	0.388718487	-1.363202373	2.47E-07	1.57E-06
ENSDARG00000074696	zgc:194621	0.388157255	-1.365286842	5.63E-07	3.41E-06
ENSDARG00000070011	ASTL (9 of 9)	0.38753709	-1.367593701	4.12E-07	2.55E-06
ENSDARG00000079443		0.384750823	-1.378003682	4.19E-10	3.65E-09
ENSDARG00000093936	si:dkeyp-1h4.6	0.384323571	-1.379606633	3.55E-24	8.82E-23
ENSDARG00000088839	BX248120.1	0.38357143	-1.382432828	9.36E-07	5.49E-06
ENSDARG00000074887	CT583723.1	0.383230823	-1.383714495	8.79E-07	5.19E-06
ENSDARG00000094331	si:dkey-189n19.3	0.383021112	-1.384504181	6.70E-06	3.49E-05
ENSDARG00000053068	cyp8b1	0.381454605	-1.390416715	4.52E-18	7.62E-17
ENSDARG00000078912	si:ch73-24k9.2	0.38067334	-1.393374559	1.35E-05	6.74E-05
ENSDARG00000076055	RPGRIP1	0.378408999	-1.401981699	9.08E-52	8.48E-50
ENSDARG00000077187	impg1a	0.378323236	-1.402308709	1.22E-11	1.24E-10
ENSDARG00000040274	SCAMP5 (2 of 2)	0.378143046	-1.402996006	1.77E-32	6.68E-31
ENSDARG00000095328	si:ch211-191i18.4	0.377557701	-1.40523095	0.001729146	0.005781777
ENSDARG00000003326	cacng5a	0.377025589	-1.407265651	6.47E-07	3.89E-06
ENSDARG00000089111		0.375932959	-1.411452689	0.001862717	0.00617656
ENSDARG00000090320	MSLN (2 of 2)	0.375143932	-1.414483872	3.20E-46	2.33E-44
ENSDARG00000034007	prom1b	0.374449353	-1.417157497	4.29E-152	2.44E-149
ENSDARG00000056771	cfhl1	0.37395149	-1.419076962	4.05E-14	5.10E-13
ENSDARG00000058023	si:dkey-11o15.4	0.373834027	-1.419530202	5.28E-08	3.65E-07
ENSDARG00000076371	SHROOM2 (1 of 2)	0.373677798	-1.420133248	9.60E-69	1.54E-66
ENSDARG00000070858	si:busm1-105l16.2	0.373133864	-1.422234798	1.13E-05	5.67E-05
ENSDARG00000093893	CCBL2 (3 of 4)	0.372706833	-1.423886827	0.001244361	0.004283801

ENSDARG00000040004	PKHD1L1 (1 of 2)	0.371770951	-1.427514049	0.000521939	0.001954192
ENSDARG00000057209	lppr5a	0.371583737	-1.428240735	5.42E-13	6.19E-12
ENSDARG00000070602	CU457778.1	0.37115838	-1.429893152	1.66E-05	8.15E-05
ENSDARG00000059824	soat2	0.370608391	-1.432032551	1.49E-59	1.84E-57
ENSDARG00000007788	atp2b1b	0.370528566	-1.432343322	2.89E-147	1.52E-144
ENSDARG00000070492	fgf21	0.370457253	-1.432621016	0.000834028	0.002991104
ENSDARG00000089208	CABZ01055343.4	0.369403091	-1.436732155	4.20E-19	7.59E-18
ENSDARG00000034940	slc1a7a	0.369176753	-1.437616383	7.63E-15	1.02E-13
ENSDARG00000011886	pdca	0.368787672	-1.439137667	1.37E-17	2.24E-16
ENSDARG00000086260	CABZ01052573.1	0.368657733	-1.439646075	3.47E-19	6.31E-18
ENSDARG00000057338	gstp2	0.367296282	-1.444983802	3.69E-19	6.69E-18
ENSDARG00000036159	trhrb	0.367032525	-1.446020179	0.000893086	0.003181793
ENSDARG00000037337	cnrip1b	0.3668986	-1.446546696	9.33E-42	5.46E-40
ENSDARG00000079078	hbaa1	0.366401573	-1.448502397	0.001248901	0.004297868
ENSDARG00000039444	FAIM2	0.365920221	-1.450398955	5.24E-35	2.30E-33
ENSDARG00000068551	zgc:153394	0.365251913	-1.453036264	1.92E-11	1.90E-10
ENSDARG00000044684	rbp4l	0.365120735	-1.453554493	0.000893462	0.003182733
ENSDARG00000070116	nit1	0.363261475	-1.460919725	0.000402937	0.001545955
ENSDARG00000004721	mpp5b	0.363063061	-1.46170794	2.97E-07	1.87E-06
ENSDARG00000054667	adra2db	0.362873211	-1.462462542	1.77E-05	8.64E-05
ENSDARG00000071450	tdrd5	0.36196448	-1.466079965	9.14E-06	4.67E-05
ENSDARG00000037487	1-Mar	0.360647167	-1.471340005	5.82E-10	5.01E-09
ENSDARG00000015404	arl3l2	0.360579787	-1.471609569	9.36E-18	1.55E-16
ENSDARG00000038363	drd4a	0.359613864	-1.475479454	9.66E-08	6.47E-07
ENSDARG00000096207	SRR (2 of 2)	0.358416531	-1.480290915	1.77E-09	1.45E-08
ENSDARG00000090562	tmprss3a	0.357847277	-1.482584094	0.001338946	0.004576666
ENSDARG00000031013	C1QL1 (2 of 2)	0.357541447	-1.483817602	0.001338391	0.00457532
ENSDARG00000058803	grk1a	0.355462894	-1.492229126	1.51E-20	2.99E-19
ENSDARG00000086756	RGS9BP	0.354470075	-1.496264256	3.01E-27	8.86E-26
ENSDARG00000093342	si:ch211-13f8.1	0.353633414	-1.499673499	8.57E-12	8.81E-11

ENSDARG00000002585		0.351237725	-1.509480287	4.88E-13	5.61E-12
ENSDARG00000078132	si:dkey-7e14.7	0.350070687	-1.514281833	3.67E-19	6.66E-18
ENSDARG00000088638	si:dkey-188p4.1	0.349531327	-1.51650633	6.37E-05	0.000283302
ENSDARG00000007382	ubtd1a	0.349434903	-1.516904378	2.32E-25	6.13E-24
ENSDARG00000061945	cngb3	0.348519586	-1.520688362	0.000139167	0.000583645
ENSDARG00000037159	oprd1b	0.347551181	-1.524702647	1.47E-09	1.21E-08
ENSDARG00000077479	MSH5	0.347480422	-1.524996398	1.42E-05	7.06E-05
ENSDARG00000074011	FAM196A (1 of 3)	0.345855126	-1.531760257	0.001514603	0.005127682
ENSDARG00000074821	NLRP6 (12 of 145)	0.344742785	-1.536407736	0.000679242	0.002483204
ENSDARG00000019772	C2	0.344060166	-1.539267224	1.18E-07	7.80E-07
ENSDARG00000078272	CABP7 (2 of 2)	0.340214093	-1.55548519	2.05E-08	1.49E-07
ENSDARG00000014745	epd	0.340139914	-1.555799785	1.25E-46	9.29E-45
ENSDARG00000021336	pax4	0.339100852	-1.560213684	1.23E-07	8.14E-07
ENSDARG00000079227	plekhs1	0.336384137	-1.571818421	3.25E-05	0.000152376
ENSDARG00000087857	si:ch211-180a12.2	0.334958431	-1.577946029	3.48E-29	1.13E-27
ENSDARG00000062661	abca4b	0.333760199	-1.583116171	1.17E-87	2.84E-85
ENSDARG00000058841	ugt2b6	0.333545757	-1.584043404	2.76E-24	6.93E-23
ENSDARG00000062228	adipoqa	0.333275302	-1.585213687	7.58E-06	3.92E-05
ENSDARG00000094708	sftpba	0.332978375	-1.586499611	5.01E-09	3.92E-08
ENSDARG00000089063	lactbl1a	0.332897752	-1.586848965	2.10E-27	6.22E-26
ENSDARG00000018263	pdia2	0.332701573	-1.58769941	4.10E-32	1.52E-30
ENSDARG00000031534	TRIM35 (8 of 43)	0.33232002	-1.589354889	3.67E-06	1.98E-05
ENSDARG00000077542	si:dkeyp-75b4.8	0.332317808	-1.589364489	0.001070353	0.003744256
ENSDARG00000090391	ugt2b3	0.332150176	-1.590092416	3.06E-24	7.67E-23
ENSDARG00000030980	csrp1b	0.330612274	-1.596787807	2.47E-09	1.99E-08
ENSDARG00000056603	mcf2b	0.33021567	-1.598519514	1.56E-08	1.15E-07
ENSDARG00000089587	CR450785.2	0.329403065	-1.602074118	5.36E-06	2.84E-05
ENSDARG00000097079	C6H3orf18 (2 of 2)	0.328803966	-1.604700393	1.74E-15	2.42E-14
ENSDARG00000091504	CABZ01117756.1	0.328791858	-1.604753521	0.000502009	0.001886287
ENSDARG00000043466	kcnk3	0.328204531	-1.607332937	0.000338889	0.001318396

ENSDARG00000095553	lenep	0.32748472	-1.610500501	6.67E-08	4.54E-07
ENSDARG00000076717	prpf4	0.327059116	-1.612376666	6.16E-16	8.92E-15
ENSDARG00000040177	rgs16	0.326927572	-1.612957042	6.64E-21	1.34E-19
ENSDARG00000089439	TMEM132A	0.326743568	-1.613769257	2.57E-15	3.55E-14
ENSDARG00000075295	tulp1a	0.325970475	-1.617186798	5.68E-71	9.60E-69
ENSDARG00000070467	lactbl1b	0.324817833	-1.622297256	4.45E-16	6.50E-15
ENSDARG00000001993	myhb	0.324471877	-1.623834653	0	0
ENSDARG00000052575	arl13a	0.323596489	-1.627732142	1.89E-50	1.66E-48
ENSDARG00000096398	si:ch211-276a17.5	0.323499177	-1.628166052	1.86E-15	2.60E-14
ENSDARG00000078832	si:dkey-73p2.4	0.323412002	-1.628554876	3.71E-09	2.94E-08
ENSDARG00000038894	tmx3	0.321619979	-1.636571063	1.26E-59	1.56E-57
ENSDARG00000034107	actl6b	0.320358069	-1.642242764	3.07E-12	3.30E-11
ENSDARG00000094557	nupr1	0.31977228	-1.644883213	3.52E-107	1.27E-104
ENSDARG00000036304	CABZ01055343.1	0.319395151	-1.646585687	2.09E-07	1.34E-06
ENSDARG00000088475	pcdh1gb9	0.319120819	-1.647825362	1.15E-08	8.61E-08
ENSDARG00000067798	ppapdc1a	0.318668396	-1.649872148	1.73E-05	8.50E-05
ENSDARG00000059173	gdf2	0.31863299	-1.65003245	0.000165708	0.000683939
ENSDARG00000057790	ankrd6a	0.316982474	-1.657525018	1.66E-16	2.50E-15
ENSDARG00000089972	CR735107.1	0.315061085	-1.666296525	4.65E-14	5.82E-13
ENSDARG00000073948	prok1	0.314224576	-1.670132075	1.88E-06	1.06E-05
ENSDARG00000056876	si:dkey-43b14.9	0.314217484	-1.670164638	3.72E-05	0.000172781
ENSDARG00000052779	zgc:153932	0.313730131	-1.672404	1.36E-09	1.12E-08
ENSDARG00000055313	slc12a10.1	0.312289379	-1.679044592	0.000777305	0.00280593
ENSDARG00000036140		0.312022989	-1.68027577	1.06E-59	1.32E-57
ENSDARG00000094118	si:dkey-21o22.2	0.311996538	-1.680398076	9.25E-07	5.44E-06
ENSDARG00000061920	NPAS4 (2 of 3)	0.311950342	-1.680611702	0.000523834	0.001960252
ENSDARG00000053845	cbln14	0.311330226	-1.683482446	2.75E-06	1.51E-05
ENSDARG00000093467	MYH13 (9 of 11)	0.30998747	-1.689718194	3.50E-151	1.95E-148
ENSDARG00000078461	pde6c	0.309677017	-1.691163779	4.93E-41	2.81E-39
ENSDARG00000077945	HEPACAM (2 of 2)	0.308355106	-1.69733536	0.000363536	0.001407723

ENSDARG00000076698	si:dkey-83h2.2	0.308130818	-1.698385112	1.78E-05	8.71E-05
ENSDARG00000028846	arl3l1	0.308004734	-1.698975568	1.66E-51	1.52E-49
ENSDARG00000045164	nptx2b	0.306866375	-1.704317523	0.000251144	0.000999926
ENSDARG00000060017	CLEC3A	0.30668198	-1.705184696	2.60E-20	5.10E-19
ENSDARG00000018478	agxtb	0.305953856	-1.708614013	1.73E-79	3.67E-77
ENSDARG00000091028	si:ch211-210m1.2	0.304791205	-1.714106823	1.51E-07	9.83E-07
ENSDARG00000042293	ca4b	0.303458636	-1.720428215	9.68E-10	8.14E-09
ENSDARG00000090744	CABZ01089777.1	0.302665085	-1.724205838	1.94E-06	1.09E-05
ENSDARG00000068483	neto1	0.302426227	-1.725344837	5.90E-06	3.10E-05
ENSDARG00000034930	cx52.6	0.301944805	-1.727643243	3.54E-08	2.50E-07
ENSDARG00000056144	TMEM150B	0.300538869	-1.734376505	5.56E-05	0.000249679
ENSDARG00000011635	SCX (1 of 2)	0.300484061	-1.734639628	2.32E-10	2.07E-09
ENSDARG00000027381	CCDC171	0.300004574	-1.736943599	8.05E-05	0.000351359
ENSDARG00000011640	syt5b	0.298165681	-1.745813882	1.22E-292	1.73E-289
ENSDARG00000056805	PLCH2 (1 of 2)	0.298123689	-1.746017079	6.39E-23	1.49E-21
ENSDARG00000015752	zap70	0.297549007	-1.748800794	2.83E-06	1.56E-05
ENSDARG00000007080	rhcgl1	0.29506131	-1.760913334	1.94E-138	9.48E-136
ENSDARG00000057661	aldoca	0.290368886	-1.784041223	2.47E-08	1.77E-07
ENSDARG00000010933	CACNA1F (1 of 2)	0.289767473	-1.787032436	1.52E-75	2.96E-73
ENSDARG00000093471	si:ch211-224m9.4	0.288952995	-1.791093271	2.26E-12	2.46E-11
ENSDARG00000003317	si:dkeyp-35f12.3	0.288852979	-1.79159272	1.80E-24	4.55E-23
ENSDARG00000002771	SLC4A5 (1 of 2)	0.287527899	-1.798226148	3.64E-20	7.08E-19
ENSDARG00000094844	si:ch211-260b17.2	0.286843479	-1.801664372	1.35E-06	7.75E-06
ENSDARG00000019179	lrit1a	0.286163498	-1.805088434	1.11E-10	1.02E-09
ENSDARG00000052223	rcvrna	0.285366026	-1.809114509	5.78E-99	1.87E-96
ENSDARG00000075425	CR376723.1	0.283663487	-1.817747637	0.000375511	0.001448364
ENSDARG00000034201	ppa1a	0.28334508	-1.819367945	1.81E-05	8.82E-05
ENSDARG00000012125	cnga1	0.282816171	-1.822063481	1.80E-11	1.78E-10
ENSDARG00000075986	RIC3 (1 of 2)	0.282452618	-1.823919223	8.87E-14	1.08E-12
ENSDARG00000013134	CABZ01071407.1	0.281585015	-1.828357534	2.96E-19	5.41E-18

ENSDARG00000010296	KCNH6	0.280377664	-1.834556673	0.000118686	0.000503405
ENSDARG00000052896	lrit1b	0.280012587	-1.836436416	3.05E-35	1.35E-33
ENSDARG00000096324	si:ch211-155d24.7	0.278788266	-1.842758255	1.76E-13	2.10E-12
ENSDARG00000070972	si:ch211-81a5.8	0.276856339	-1.85279054	1.06E-16	1.61E-15
ENSDARG00000035798	gngt1	0.27673761	-1.853409367	9.80E-46	6.97E-44
ENSDARG00000063293	neto2b	0.276136718	-1.856545356	3.90E-09	3.08E-08
ENSDARG00000074519	si:dkey-224j12.3	0.276040986	-1.857045603	3.06E-07	1.92E-06
ENSDARG00000087082	CNGB3 (2 of 2)	0.275973503	-1.857398337	1.68E-24	4.27E-23
ENSDARG00000037805	lgals3bpa	0.275052368	-1.862221769	3.78E-05	0.000175246
ENSDARG00000068474	si:dkey-94e7.2	0.274847306	-1.863297756	1.91E-23	4.54E-22
ENSDARG00000076872	MPP4 (3 of 3)	0.271351101	-1.881767334	1.05E-26	2.98E-25
ENSDARG00000004643	cdhr1a	0.271340703	-1.881822617	1.30E-40	7.21E-39
ENSDARG00000021345	prph2l	0.269091411	-1.893831754	9.56E-34	3.88E-32
ENSDARG00000068088	tcnl	0.268023081	-1.899570848	2.19E-34	9.29E-33
ENSDARG00000078622	scpp5	0.267898798	-1.900239989	1.85E-09	1.51E-08
ENSDARG00000069615	ckmt2a	0.267037928	-1.904883431	4.19E-220	4.25E-217
ENSDARG00000009637		0.266065208	-1.910148228	3.69E-90	9.43E-88
ENSDARG00000091068	CABZ01063789.1	0.265942997	-1.910811047	8.61E-10	7.27E-09
ENSDARG00000070726	cnga3a	0.264882527	-1.916575417	6.91E-46	4.95E-44
ENSDARG00000078210	tulp1b	0.264414413	-1.919127277	4.25E-54	4.32E-52
ENSDARG00000053207	MYH13 (5 of 11)	0.263755439	-1.922727248	6.20E-178	4.09E-175
ENSDARG00000011113	chrna10a	0.263417706	-1.924575773	5.11E-09	3.99E-08
ENSDARG00000070931	si:ch211-232m10.6	0.261924112	-1.932779221	5.12E-09	4.00E-08
ENSDARG00000082099	dre-mir-137-3	0.261637846	-1.934356854	2.63E-06	1.45E-05
ENSDARG00000076448	serpinf2a	0.260821477	-1.938865424	1.24E-14	1.63E-13
ENSDARG00000038770	zgc:103625	0.260091807	-1.942907142	3.31E-44	2.18E-42
ENSDARG00000034628	slc1a8a	0.258762324	-1.950300521	5.45E-51	4.90E-49
ENSDARG00000011671	pde6b	0.257235833	-1.958836473	1.58E-26	4.43E-25
ENSDARG00000010555	pdha1b	0.257169778	-1.959206983	1.00E-08	7.57E-08
ENSDARG00000092243	si:ch211-163b2.2	0.255352934	-1.969435457	1.49E-11	1.49E-10

ENSDARG00000044475	ompa	0.255274248	-1.969880087	1.85E-23	4.40E-22
ENSDARG00000075058	gabra6a	0.254306998	-1.975356933	4.08E-07	2.53E-06
ENSDARG00000044199	gnat1	0.254135497	-1.976330196	1.33E-92	3.67E-90
ENSDARG00000091228	C12H17orf62 (2 of 2)	0.252983849	-1.982882812	6.99E-27	2.01E-25
ENSDARG00000086360	RP1 (2 of 2)	0.251866561	-1.9892685	6.96E-50	5.99E-48
ENSDARG00000021055	isl1l	0.248671244	-2.007688407	0.000112724	0.000480414
ENSDARG00000091745	CABZ01055591.1	0.247264457	-2.015873223	2.59E-07	1.65E-06
ENSDARG00000043953	BX119910.1	0.245279979	-2.027498617	6.21E-17	9.60E-16
ENSDARG00000012610	saga	0.244496154	-2.032116326	7.00E-107	2.48E-104
ENSDARG00000093549	sepp1a	0.243817027	-2.036129217	0	0
ENSDARG00000061481		0.243724762	-2.036675259	6.94E-129	3.18E-126
ENSDARG00000041735	tmem237a	0.243003102	-2.040953366	1.95E-23	4.64E-22
ENSDARG00000041205	slc6a1l	0.241886948	-2.047595169	8.40E-08	5.66E-07
ENSDARG00000063519	PLEKHB1 (1 of 2)	0.240332255	-2.056897808	2.34E-06	1.30E-05
ENSDARG00000076043	si:dkeyp-73d8.9	0.239379244	-2.062630033	6.59E-13	7.49E-12
ENSDARG00000054797	RDH13 (3 of 3)	0.238680981	-2.066844485	2.84E-70	4.74E-68
ENSDARG00000029321		0.238157889	-2.070009756	1.81E-08	1.33E-07
ENSDARG00000079544	si:ch1073-464p5.5	0.237444064	-2.074340407	5.83E-20	1.12E-18
ENSDARG00000013393	guca1b	0.237133179	-2.076230563	7.54E-05	0.000330866
ENSDARG00000090428	ctrb1	0.236354857	-2.080973581	1.13E-23	2.74E-22
ENSDARG00000088025		0.235849997	-2.08405851	7.43E-06	3.85E-05
ENSDARG00000013051	C5H11orf53 (1 of 2)	0.235289306	-2.087492344	5.00E-05	0.000226462
ENSDARG00000096756	si:ch211-196c10.13	0.234170357	-2.094369636	1.37E-28	4.30E-27
ENSDARG00000079569	CR385022.1	0.232803517	-2.102815243	2.11E-16	3.15E-15
ENSDARG00000045811		0.226512627	-2.142336619	9.76E-18	1.61E-16
ENSDARG00000018530	mapkapk2b	0.225475948	-2.148954552	1.48E-21	3.12E-20
ENSDARG00000093153	si:ch73-268e11.2	0.22314615	-2.163939182	1.44E-05	7.11E-05
ENSDARG00000014095	CT027835.1	0.222581815	-2.167592366	4.62E-05	0.00021078
ENSDARG00000041162	GRM1 (2 of 2)	0.221544776	-2.174329788	7.84E-10	6.65E-09
ENSDARG00000079413	PPEF2	0.221042294	-2.177605655	5.53E-11	5.25E-10

ENSDARG00000070652	si:ch211-134a4.1	0.220710133	-2.179775232	8.10E-15	1.08E-13
ENSDARG00000091797	ugt2b5	0.214389	-2.221697212	2.82E-09	2.26E-08
ENSDARG00000007086	aqp10a	0.211279205	-2.24277732	1.27E-06	7.32E-06
ENSDARG00000052620		0.211043721	-2.244386192	6.17E-07	3.72E-06
ENSDARG00000095802	C20H2orf71	0.210443615	-2.248494354	1.40E-44	9.44E-43
ENSDARG00000070666	rhol	0.208788738	-2.259884201	2.85E-09	2.28E-08
ENSDARG00000059978	cplx4a	0.208686138	-2.260593321	3.89E-58	4.60E-56
ENSDARG00000089270	FP102168.1	0.205698519	-2.281396689	1.29E-05	6.43E-05
ENSDARG00000019752	rom1a	0.204993454	-2.286350254	7.16E-44	4.62E-42
ENSDARG00000097311	si:dkey-201i2.1	0.202331262	-2.305208845	1.12E-07	7.42E-07
ENSDARG00000095820	si:dkey-6n3.3	0.20202986	-2.307359556	4.90E-17	7.65E-16
ENSDARG00000019902	rcv1	0.198591601	-2.33212349	0	0
ENSDARG00000026926	rom1b	0.196409644	-2.348062328	1.68E-46	1.24E-44
ENSDARG00000073742	si:ch73-103b2.3	0.196283947	-2.348985904	4.58E-27	1.33E-25
ENSDARG00000020814	faimb	0.19561382	-2.353919799	7.04E-27	2.02E-25
ENSDARG00000009386	MPP4 (1 of 3)	0.191917735	-2.381440062	2.29E-24	5.77E-23
ENSDARG00000037656	si:ch1073-417l11.1	0.191819659	-2.382177509	4.64E-39	2.43E-37
ENSDARG00000035163	tmem136b	0.191763453	-2.382600303	3.05E-08	2.17E-07
ENSDARG00000077687	rp1	0.191366804	-2.385587508	1.08E-15	1.53E-14
ENSDARG00000060792	si:dkey-97i18.6	0.18918023	-2.402166763	7.15E-07	4.27E-06
ENSDARG00000002193	rho	0.18794384	-2.411626461	0	0
ENSDARG00000061149	nrn1lb	0.186664246	-2.42148248	6.78E-24	1.66E-22
ENSDARG00000071592	aqp8a.2	0.185228626	-2.432621016	6.09E-08	4.16E-07
ENSDARG00000070439	pde6h	0.185045675	-2.434046682	1.37E-31	4.93E-30
ENSDARG00000088637	CR391924.1	0.182627479	-2.453024238	2.10E-27	6.22E-26
ENSDARG00000053480	aqp9b	0.180795597	-2.467568549	1.37E-27	4.10E-26
ENSDARG00000031588	si:dkey-239b22.1	0.180182218	-2.472471454	7.53E-164	4.65E-161
ENSDARG00000076015	PLBD1 (2 of 2)	0.17458816	-2.517972372	5.90E-07	3.57E-06
ENSDARG00000061101	snx19a	0.171853268	-2.540750807	3.10E-55	3.25E-53
ENSDARG00000057792	cx52.7	0.170227474	-2.554464195	1.85E-06	1.04E-05

ENSDARG00000092968		0.166086165	-2.589996197	2.00E-08	1.46E-07
ENSDARG00000027236	rs1a	0.161806313	-2.627660202	0	0
ENSDARG00000096216	si:ch211-162i8.7	0.159916796	-2.64460662	2.96E-07	1.86E-06
ENSDARG00000043820	nme2a	0.15705179	-2.670687708	1.86E-49	1.56E-47
ENSDARG00000027424	slc25a3a	0.15220496	-2.715912718	1.07E-56	1.18E-54
ENSDARG00000052664	MSLN (1 of 2)	0.149532878	-2.741465364	1.27E-29	4.19E-28
ENSDARG00000090805	CR385054.2	0.149190334	-2.744774029	1.03E-44	6.96E-43
ENSDARG00000030626	lrit2	0.146606634	-2.769977706	5.96E-15	8.02E-14
ENSDARG00000079274	zgc:66382	0.141310073	-2.823063789	1.23E-10	1.13E-09
ENSDARG00000023886	cacna2d4b	0.140475807	-2.831606407	5.52E-55	5.74E-53
ENSDARG00000045592	tnni2a.1	0.1404398	-2.831976251	1.75E-88	4.37E-86
ENSDARG00000035791	si:busm1-71b9.3	0.140244657	-2.833982282	9.18E-10	7.74E-09
ENSDARG00000097539	si:ch211-39f2.3	0.136932761	-2.868460443	1.92E-35	8.60E-34
ENSDARG00000017490	cel.1	0.129854005	-2.945037589	2.20E-22	4.89E-21
ENSDARG00000014840	prph2b	0.129312301	-2.951068573	8.11E-43	4.92E-41
ENSDARG00000038742	RBP1	0.126944187	-2.977733766	1.02E-09	8.54E-09
ENSDARG00000074119		0.126504059	-2.982744419	1.47E-13	1.77E-12
ENSDARG00000011184	grk1b	0.126080978	-2.987577467	1.17E-19	2.20E-18
ENSDARG00000005776	guk1b	0.124617387	-3.004422727	8.26E-116	3.45E-113
ENSDARG00000057427	sv2ba	0.124432681	-3.006562645	4.26E-265	5.26E-262
ENSDARG00000019492	shbg	0.121065824	-3.046136433	1.24E-11	1.26E-10
ENSDARG00000091135	RD3L	0.120882392	-3.048323984	1.24E-11	1.25E-10
ENSDARG00000089582	si:dkey-265e15.2	0.114492218	-3.126678549	6.36E-17	9.82E-16
ENSDARG00000076565	GPR151	0.111764171	-3.161470332	9.06E-18	1.50E-16
ENSDARG00000088751	IMPG2	0.110215219	-3.181604646	2.66E-07	1.69E-06
ENSDARG00000042529	gnat2	0.108705613	-3.20150166	3.51E-216	3.32E-213
ENSDARG00000056511	arr3a	0.10529059	-3.247551595	0	0
ENSDARG00000020602	grk7a	0.104351586	-3.260475565	2.65E-82	5.91E-80
ENSDARG00000019782		0.099069131	-3.335420589	1.79E-35	8.02E-34
ENSDARG00000017634	pdcb	0.09540884	-3.389733239	2.60E-97	8.20E-95

ENSDARG00000096659	NLRP6 (145 of 145)	0.089728571	-3.47828875	1.47E-28	4.62E-27
ENSDARG00000052138	slc1a2a	0.086344438	-3.53375295	1.12E-11	1.14E-10
ENSDARG00000044280	opn1mw2	0.084646248	-3.562410069	1.51E-35	6.83E-34
ENSDARG00000087311	zgc:171951	0.077833589	-3.6834633	2.02E-25	5.36E-24
ENSDARG00000037925	rgs9a	0.076644275	-3.705678153	3.55E-37	1.71E-35
ENSDARG00000044861	opn1lw2	0.076499111	-3.708413206	0	0
ENSDARG00000002696	gnb3b	0.073950558	-3.75729516	2.85E-181	1.93E-178
ENSDARG00000087583	si:ch211-226h8.13	0.067271833	-3.893853622	8.23E-10	6.96E-09
ENSDARG00000089997	gngt2b	0.06086204	-4.038313502	0	0
ENSDARG00000038018	prph2a	0.057215238	-4.127456761	2.91E-75	5.61E-73
ENSDARG00000017274	opn1sw2	0.05352218	-4.223719298	8.93180033685773e-312	0.00E+00
ENSDARG00000045677	opn1sw1	0.043409758	-4.525836818	0	0
