# Supplementary material for: Whole organism transcriptome analysis of zebrafish models of Bardet-Biedl Syndrome and Alström Syndrome provides mechanistic insight into shared and divergent phenotypes
Source: BMC Genomics. 2016 May 3;17:318. doi: 10.1186/s12864-016-2679-1 (PMC4855444; doi:10.1186/s12864-016-2679-1)
Supplement: Additional file 3: Table S2. — Differentially expressed genes in BBS model. All genes exhibiting significant changes in bbs1-depleted zebrafish larvae, relative to control. Columns represent ENSEMBL transcript ID (Feature.ID), gene name (gene_symbol), fold change (FC), p-value relative to control (p < 0.05), false discovery rate (FDR; FDR < 0.05). (PDF 611 kb) [file 12864_2016_2679_MOESM3_ESM.pdf]

Supplementary Table 2. Differentially expressed genes in BBS model

Feature.ID	gene_symbol	FC	LFC(BBS/Control)	p.Value	FDR
ENSDARG00000074210		117.0063206	6.870442655	3.52E-21	3.23E-20
ENSDARG00000094559	zgc:174855	98.16697308	6.617165826	0	0
ENSDARG00000094508	si:dkey-21o19.8	97.58691773	6.608615851	9.26E-202	4.18E-199
ENSDARG00000090014	CAPN2 (3 of 4)	88.17824696	6.46235089	1.73E-149	4.39E-147
ENSDARG00000096563	si:dkey-269i1.2	81.65940024	6.351547066	2.84E-138	6.12E-136
ENSDARG00000095072	si:dkey-26g8.4	74.62013284	6.221493023	0	0
ENSDARG00000075527	si:dkey-269i1.3	69.50396377	6.119023351	0	0
ENSDARG00000079376	zgc:174153	69.00902386	6.108713121	0	0
ENSDARG00000091085	lepa	67.79511512	6.083109421	1.17E-61	4.81E-60
ENSDARG00000089750	si:dkey-26g8.5	67.70008425	6.081085724	0	0
ENSDARG00000097527	si:ch73-56d11.3	59.86932657	5.903745137	5.63E-11	2.77E-10
ENSDARG00000078738	NAMPT (3 of 6)	51.04286411	5.673637378	3.64E-09	1.56E-08
ENSDARG00000094584	si:ch211-92l17.1	50.74383281	5.665160589	1.17E-90	9.67E-89
ENSDARG00000074306	ctslb	44.75563078	5.483997297	0	0
ENSDARG00000093639	si:dkey-90a24.1	44.58463426	5.478474677	3.43E-220	1.74E-217
ENSDARG00000090918	si:dkey-269i1.4	41.63876156	5.379855255	0	0
ENSDARG00000078557	CU896645.1	39.91871365	5.318993327	6.53648849447969e-321	6.19070677092478e-318
ENSDARG00000097929	si:dkey-117j14.6	37.51722262	5.229481125	0	0
ENSDARG00000069382	mxtx1	34.77412892	5.119942471	5.91E-17	4.30E-16
ENSDARG00000095893	si:dkey-85n7.7	33.76408144	5.077417404	1.31E-44	3.27E-43
ENSDARG00000095744	si:dkey-269i1.4	31.94302531	4.997429051	2.74E-236	1.56E-233
ENSDARG00000069481	ghrh	31.37070492	4.971346043	1.85E-88	1.46E-86
ENSDARG00000088938	BX548011.4	29.96726547	4.905315538	2.00E-09	8.72E-09
ENSDARG00000079403	si:dkey-204l11.1	27.82437634	4.798277446	1.94E-161	5.75E-159
ENSDARG00000095252	si:dkey-40h20.1	26.22467764	4.712853134	3.03E-158	8.77E-156
ENSDARG00000045548	lepb	24.07431241	4.589422689	2.05E-18	1.61E-17
ENSDARG00000093738	pth1a	22.8344365	4.513139283	7.92E-24	8.28E-23
ENSDARG00000023656	he1a	21.72308964	4.441157405	0	0

ENSDARG00000020738	zgc:153921	21.38451925	4.418494869	5.07E-07	1.81E-06
ENSDARG00000095866	fabp1b.2	21.13748453	4.401731794	2.65E-48	7.39E-47
ENSDARG00000092126	vtg5	19.60943399	4.293475989	5.41E-09	2.29E-08
ENSDARG00000019122	he1b	19.43703581	4.280736317	0	0
ENSDARG00000095254	si:dkey-239j18.2	18.39267025	4.201059041	4.86E-16	3.33E-15
ENSDARG00000037859	il11a	18.24879001	4.189728904	1.58E-18	1.25E-17
ENSDARG00000096603	bmb	17.78288417	4.152417426	0	0
ENSDARG00000070212	zgc:158463	17.75931821	4.150504292	9.64E-104	1.09E-101
ENSDARG00000056551	si:dkey-24c2.6	17.56329864	4.134491924	5.11E-08	1.99E-07
ENSDARG00000094983	si:ch211-122l24.5	17.45923949	4.125918813	3.09E-10	1.44E-09
ENSDARG00000051775		17.3340326	4.115535418	8.05E-12	4.20E-11
ENSDARG00000053563	si:ch73-56d11.4	15.13345327	3.919669326	1.78E-14	1.11E-13
ENSDARG00000084533	BX296557.1	14.45472517	3.853469274	1.92E-16	1.35E-15
ENSDARG00000091345	Metazoa_SRP	14.41360539	3.849359349	1.46E-09	6.49E-09
ENSDARG00000037262	CDKN2B	14.22595748	3.830453851	6.12E-21	5.55E-20
ENSDARG00000086233		13.8956172	3.79655801	1.13E-07	4.28E-07
ENSDARG00000040194	BX545917.1	13.66411145	3.772319743	4.26E-42	9.51E-41
ENSDARG00000091047	CABZ01045617.2	13.50177453	3.755077127	7.37E-53	2.39E-51
ENSDARG00000095489	BX296549.5	13.06507519	3.707643522	3.48E-12	1.86E-11
ENSDARG00000092419	vtg7	12.62977914	3.658757506	1.62E-270	1.28E-267
ENSDARG00000085168	AL935186.3	12.46754716	3.640105755	1.48E-216	7.23E-214
ENSDARG00000076930		12.29974379	3.620556359	1.88E-06	6.36E-06
ENSDARG00000007344	tcap	11.9924949	3.584059921	0	0
ENSDARG00000070289	zgc:171759	11.93975814	3.577701707	1.07E-07	4.06E-07
ENSDARG00000074937		11.86102557	3.568156854	1.53E-07	5.74E-07
ENSDARG00000092233	vtg1	11.71448218	3.550221279	1.35E-70	7.02E-69
ENSDARG00000090873	si:ch211-122l24.4	11.67378564	3.545200578	1.33E-20	1.18E-19
ENSDARG00000088485	CU651569.4	11.38790173	3.509430043	3.43E-07	1.25E-06
ENSDARG00000087472	HIST2H4B (2 of 2)	11.27608908	3.495194876	1.41E-10	6.73E-10
ENSDARG00000088726		11.17601717	3.482334238	1.74E-57	6.47E-56

ENSDARG00000094931		11.17573366	3.482297638	5.13E-07	1.83E-06
ENSDARG00000087934	CABZ01080435.1	11.14323467	3.478096175	7.68E-131	1.45E-128
ENSDARG00000089806	si:dkey-239j18.3	11.01187094	3.460987701	0	0
ENSDARG00000069685		10.59124832	3.404800736	1.09E-05	3.40E-05
ENSDARG00000091205	si:ch211-113a14.19	10.50902466	3.393556874	1.35E-16	9.63E-16
ENSDARG00000085949	AL935186.4	10.44667621	3.384972092	0	0
ENSDARG00000070297	zgc:112234	10.26648499	3.359870415	2.67E-06	8.89E-06
ENSDARG00000014024	ms4a17a.4	10.11887208	3.338976581	4.40E-30	6.04E-29
ENSDARG00000039547	zgc:171759	10.08691182	3.334412646	3.53E-18	2.74E-17
ENSDARG00000055424	CR392362.1	9.984917982	3.319750577	1.70E-05	5.20E-05
ENSDARG00000039730	zgc:112160	9.863852366	3.302151207	1.63E-19	1.37E-18
ENSDARG00000088951	AL935186.6	9.820525361	3.295800205	3.77E-18	2.92E-17
ENSDARG00000076554	cdkn1a	9.753151205	3.285868423	0	0
ENSDARG00000089617	RNaseP_nuc	9.577866199	3.259704282	8.02E-20	6.86E-19
ENSDARG00000094610	wu:fe11b02	9.473892914	3.243957365	1.23E-07	4.64E-07
ENSDARG00000070280		9.42932501	3.2371545	5.43E-07	1.94E-06
ENSDARG00000089719	si:dkey-108k21.11	9.24891371	3.20928393	1.29E-05	4.01E-05
ENSDARG00000074631	HIST4H4 (1 of 2)	9.029873817	3.174705828	1.94E-05	5.92E-05
ENSDARG00000013855	slc12a3	8.99417641	3.168991181	1.87E-89	1.50E-87
ENSDARG00000089044		8.941141756	3.160459071	7.59E-09	3.17E-08
ENSDARG00000078674	hspb9	8.922488169	3.157446083	0	0
ENSDARG00000093195	si:dkey-261m9.6	8.872887795	3.149403725	6.81E-08	2.61E-07
ENSDARG00000014277	vox	8.831846021	3.14271502	8.29E-66	3.81E-64
ENSDARG00000051734		8.805469155	3.138399873	2.82E-05	8.45E-05
ENSDARG00000078724	wu:fe11b02	8.79611035	3.136865703	8.07E-09	3.37E-08
ENSDARG00000094744	zgc:171759	8.65076885	3.11282836	4.79E-19	3.92E-18
ENSDARG00000093071		8.582962229	3.10147565	3.36E-10	1.56E-09
ENSDARG00000011701	ctsll	8.322397394	3.056999179	3.98E-53	1.30E-51
ENSDARG00000089437		8.318647707	3.056349021	1.07E-05	3.33E-05
ENSDARG00000086374	isg15	8.25042127	3.044467786	8.14E-26	9.30E-25

ENSDARG00000033355	PLA2G4C (1 of 5)	8.092526394	3.016590166	5.61E-09	2.37E-08
ENSDARG00000087670		8.076242335	3.013684201	8.85E-05	0.000251415
ENSDARG00000088381	Metazoa_SRP	8.07082651	3.012716423	1.19E-10	5.72E-10
ENSDARG00000089382	zgc:158463	7.971348847	2.994823866	5.47E-54	1.84E-52
ENSDARG00000085067	AL935186.2	7.944751039	2.990002011	3.01E-168	9.62E-166
ENSDARG00000071583	gtf3ab	7.938048097	2.988784304	2.08E-06	6.99E-06
ENSDARG00000039173	ctslb	7.859473729	2.974432713	5.73E-48	1.58E-46
ENSDARG00000001721	oc90	7.848226994	2.972366769	8.77E-156	2.44E-153
ENSDARG00000092662	dnaaf3	7.710798687	2.946880303	1.77E-05	5.41E-05
ENSDARG00000034203	zgc:171759	7.700210931	2.944897966	1.47E-06	5.02E-06
ENSDARG00000078962	DUOXA1	7.675827749	2.940322337	3.10E-32	4.64E-31
ENSDARG00000086552		7.602187346	2.926414579	3.43E-05	0.000101841
ENSDARG00000007602	si:dkey-79f11.4	7.592719784	2.924616765	2.62E-08	1.05E-07
ENSDARG00000074012	FP325123.1	7.591684523	2.924420042	8.57E-06	2.71E-05
ENSDARG00000012395	mmp13a	7.528215758	2.912307976	1.46E-113	2.04E-111
ENSDARG00000007276	ela3l	7.483211423	2.903657536	2.52E-12	1.36E-11
ENSDARG00000088533	BX537263.8	7.467953828	2.900713008	1.82E-07	6.78E-07
ENSDARG00000081218	5_8S_rRNA	7.453465715	2.897911406	8.02E-240	5.07E-237
ENSDARG00000082257	dre-mir-219-1	7.397853235	2.887106679	1.69E-06	5.75E-06
ENSDARG00000086555		7.277926831	2.863527547	6.50E-10	2.95E-09
ENSDARG00000002295	si:dkey-21p1.3	7.256590485	2.859291856	3.89E-22	3.73E-21
ENSDARG00000090426	CU457819.3	7.238963375	2.855783117	5.03E-08	1.96E-07
ENSDARG00000093052	c6	7.203271991	2.848652381	1.19E-41	2.62E-40
ENSDARG00000090297	ldlrad2	7.096344733	2.827076096	1.01E-121	1.66E-119
ENSDARG00000002204	hspb11	7.096165266	2.82703961	4.86E-220	2.42E-217
ENSDARG00000015355	fosl1a	7.026929674	2.81289446	2.09E-112	2.85E-110
ENSDARG00000095761	si:dkey-58b18.8	6.977975451	2.802808522	1.46E-05	4.51E-05
ENSDARG00000063223	arl14	6.960852954	2.799264099	2.24E-103	2.51E-101
ENSDARG00000053774	alpi.2	6.938630046	2.794650847	4.53E-15	2.93E-14
ENSDARG00000092395	CABZ01086354.1	6.814123161	2.768528024	2.99E-07	1.09E-06

ENSDARG00000043328	fip1l1a	6.803633918	2.766305516	8.20E-18	6.25E-17
ENSDARG00000025428	socs3a	6.765016434	2.758093439	1.43E-270	1.16E-267
ENSDARG00000052288	CR788231.1	6.679738559	2.739791638	3.28E-08	1.30E-07
ENSDARG00000088963	Metazoa_SRP	6.611088346	2.724887794	2.45E-06	8.17E-06
ENSDARG00000097785	hist2h3c	6.60661348	2.723910942	2.59E-10	1.21E-09
ENSDARG00000074260	si:ch211-113a14.19	6.59604467	2.721601169	3.82E-12	2.03E-11
ENSDARG00000080156	SNORD30	6.561133102	2.713944989	4.21E-05	0.000123856
ENSDARG00000085497	RNaseP_nuc	6.494986392	2.699326503	9.97E-06	3.13E-05
ENSDARG00000020332	nkx2.9	6.492957043	2.698875665	1.98E-38	3.81E-37
ENSDARG00000090182		6.488649333	2.6979182	0.000110717	0.000310421
ENSDARG00000097752	si:rp71-77l1.2	6.428862481	2.68456349	1.53E-13	8.93E-13
ENSDARG00000089039		6.376151745	2.672685964	1.44E-05	4.45E-05
ENSDARG00000031683	fos	6.368814276	2.671024801	1.13E-237	6.97E-235
ENSDARG00000068941	HIST2H3C	6.268326659	2.648080364	5.47E-14	3.29E-13
ENSDARG00000087425	SNORA62	6.240489582	2.641659216	8.32E-06	2.63E-05
ENSDARG00000009443	zgc:92137	6.173307516	2.626043659	3.31E-08	1.31E-07
ENSDARG00000089397	wu:fe11b02	6.173169605	2.626011429	6.12E-08	2.36E-07
ENSDARG00000075263	ankrd1a	6.044497531	2.595622416	3.97E-131	7.57E-129
ENSDARG00000092858	si:ch1073-126c3.2	6.035180451	2.593396908	5.94E-06	1.91E-05
ENSDARG00000068874	PTCHD3 (2 of 3)	6.005133816	2.586196395	1.66E-05	5.11E-05
ENSDARG00000087451		5.974955841	2.578928051	0.000316934	0.000836511
ENSDARG00000090893	HIST1H4A	5.96870672	2.577418367	4.88E-07	1.75E-06
ENSDARG00000093546	ms4a17a.2	5.956033624	2.574351898	4.28E-16	2.94E-15
ENSDARG00000086546		5.951028353	2.573138991	1.40E-06	4.78E-06
ENSDARG00000095340		5.933239221	2.568819952	0.000324288	0.000854652
ENSDARG00000033662	scd	5.933013565	2.568765081	5.98E-23	6.01E-22
ENSDARG00000094057	cyp2j20	5.897318834	2.560059195	3.55E-08	1.40E-07
ENSDARG00000091667	si:dkey-234i14.3	5.847697762	2.547868748	1.26E-21	1.18E-20
ENSDARG00000090079	Metazoa_SRP	5.823990594	2.542008026	2.13E-05	6.47E-05
ENSDARG00000035422	cyr61l1	5.804843941	2.537257282	2.14E-106	2.55E-104

ENSDARG00000097258	hist2h3c	5.802166774	2.536591764	0.000163455	0.000447854
ENSDARG00000095424	si:ch211-133n4.7	5.801047166	2.536313349	0.00024646	0.000658207
ENSDARG00000090186	rps27.2	5.800881845	2.536272234	3.36E-254	2.27E-251
ENSDARG00000032637	si:dkey-261m9.12	5.764850895	2.527283294	0.000167428	0.000457814
ENSDARG00000077239		5.732893765	2.519263545	1.22E-05	3.79E-05
ENSDARG00000097103	HIST1H4L	5.692309955	2.509014221	8.44E-05	0.000240397
ENSDARG00000040118	zgc:113232	5.674563025	2.504509301	5.83E-195	2.47E-192
ENSDARG00000083337	RNase_MRP	5.577279251	2.479561508	7.23E-31	1.02E-29
ENSDARG00000093910	FP015791.1	5.568686904	2.47733718	7.08E-57	2.58E-55
ENSDARG00000088291		5.543790301	2.470872687	4.35E-05	0.000127765
ENSDARG00000087564	NEK11	5.508401385	2.461633689	1.21E-14	7.65E-14
ENSDARG00000075508	si:dkey-108k21.10	5.455741054	2.447775171	8.66E-08	3.30E-07
ENSDARG00000039806	ntlb	5.426862611	2.440118385	5.46E-14	3.29E-13
ENSDARG00000053417	HTR3C	5.402343106	2.433585269	9.96E-18	7.55E-17
ENSDARG00000095161	si:rp71-10g3.1	5.399215266	2.432749738	6.38E-06	2.04E-05
ENSDARG00000088259	wu:fe11b02	5.382539191	2.428286919	1.09E-05	3.39E-05
ENSDARG00000056210	si:ch211-199o1.2	5.37844081	2.427188001	2.35E-101	2.53E-99
ENSDARG00000076129	CABZ01086354.1	5.375216567	2.426322882	5.44E-06	1.75E-05
ENSDARG00000017164	vent	5.374820816	2.426216659	1.28E-05	3.97E-05
ENSDARG00000077587	si:dkey-261m9.16	5.343146628	2.417689608	3.62E-09	1.55E-08
ENSDARG00000070265		5.339483959	2.416700318	5.46E-06	1.76E-05
ENSDARG00000019763	acp5a	5.306229639	2.407687113	1.84E-204	8.45E-202
ENSDARG00000091446		5.264683104	2.396346694	2.10E-05	6.37E-05
ENSDARG00000088479	Metazoa_SRP	5.264267232	2.396232727	0.000636186	0.001612483
ENSDARG00000088889	si:dkey-108k21.14	5.233773835	2.387851583	3.86E-07	1.39E-06
ENSDARG00000093477	CYP46A1 (5 of 5)	5.216131766	2.382980314	2.47E-05	7.44E-05
ENSDARG00000080823	SNORA73	5.213126918	2.382148984	0.000242635	0.00064903
ENSDARG00000042816	mmp9	5.211000319	2.381560343	2.63E-169	8.58E-167
ENSDARG00000088154	CABZ01072971.1	5.210609788	2.381452218	2.16E-13	1.25E-12
ENSDARG00000090909	CU651569.6	5.194996349	2.377122735	1.09E-05	3.40E-05

ENSDARG00000088371	junbb	5.190902545	2.375985403	1.87E-224	9.83E-222
ENSDARG00000086709	si:ch211-113a14.14	5.184900458	2.374316292	1.90E-06	6.42E-06
ENSDARG00000031483	col9a1	5.175817908	2.371786862	1.31E-64	5.85E-63
ENSDARG00000096310	TAF1C	5.152016507	2.365137216	4.29E-152	1.11E-149
ENSDARG00000087095	Metazoa_SRP	5.151435863	2.364974612	2.98E-05	8.90E-05
ENSDARG00000090491	Metazoa_SRP	5.151213018	2.364912201	1.59E-09	7.01E-09
ENSDARG00000057113	c6	5.142484007	2.362465402	7.82E-228	4.19E-225
ENSDARG00000077327	si:dkey-21o19.6	5.131747299	2.35945013	8.22E-107	9.93E-105
ENSDARG00000054321	ngs	5.108930209	2.353021228	3.04E-190	1.25E-187
ENSDARG00000096992	zgc:153405	5.078115304	2.344293153	1.52E-05	4.68E-05
ENSDARG00000038025	cbx7a	5.074758823	2.34333926	3.36E-112	4.55E-110
ENSDARG00000086076	HIST1H4D	5.071893782	2.342524532	4.94E-07	1.77E-06
ENSDARG00000091313	HIST4H4 (2 of 2)	5.044114506	2.334601026	0.000338094	0.000889386
ENSDARG00000033231	mcm6l	5.0137727	2.325896593	2.98E-07	1.09E-06
ENSDARG00000061547	zgc:153409	5.001831886	2.322456569	2.12E-05	6.44E-05
ENSDARG00000058815	ihhb	4.987424444	2.318294986	1.72E-14	1.07E-13
ENSDARG00000092743	zgc:171759	4.985788709	2.317821745	3.88E-06	1.27E-05
ENSDARG00000013856	amy2a	4.980812983	2.316381242	4.95E-05	0.00014458
ENSDARG00000095533	si:ch211-198c19.3	4.955065256	2.308904057	7.55E-29	9.81E-28
ENSDARG00000040442	prtgb	4.947341809	2.306653578	3.32E-79	2.16E-77
ENSDARG00000086666	HIST1H4K	4.93740933	2.303754255	0.000116836	0.000326418
ENSDARG00000051770		4.890861221	2.290088529	7.96E-05	0.000227374
ENSDARG00000091770	si:ch211-113a14.14	4.852798991	2.278817103	3.33E-07	1.21E-06
ENSDARG00000079783	isg20	4.851741238	2.278502608	1.63E-212	7.85E-210
ENSDARG00000087853	Metazoa_SRP	4.839622599	2.274894549	4.16E-51	1.27E-49
ENSDARG00000076667	ccng1	4.837464428	2.274251053	6.60E-06	2.11E-05
ENSDARG00000075366	si:ch211-113a14.10	4.827531741	2.271285745	2.14E-07	7.94E-07
ENSDARG00000090123	Metazoa_SRP	4.818453011	2.268570036	0.000109907	0.000308383
ENSDARG00000087506	BX548011.3	4.797159552	2.262180424	2.03E-05	6.18E-05
ENSDARG00000016918	ace2	4.789564838	2.259894584	1.29E-07	4.85E-07

ENSDARG00000089031	larsa	4.760223295	2.25102925	1.70E-06	5.76E-06
ENSDARG00000090149	HIST1H4I	4.744410229	2.246228759	1.38E-05	4.29E-05
ENSDARG00000087774	Metazoa_SRP	4.742740814	2.245721029	2.75E-30	3.81E-29
ENSDARG00000087004	si:ch211-122l24.4	4.714751448	2.237181717	1.79E-14	1.11E-13
ENSDARG00000096596	si:ch211-113a14.14	4.702101309	2.233305623	6.53E-10	2.96E-09
ENSDARG00000035559	tp53	4.691486115	2.230044996	6.27E-108	7.81E-106
ENSDARG00000007377	odc1	4.69121684	2.229962187	4.47E-142	1.00E-139
ENSDARG00000091744	BX296557.7	4.652814632	2.218103711	5.71E-105	6.54E-103
ENSDARG00000059369		4.639280416	2.213901051	5.53E-06	1.78E-05
ENSDARG00000089064	CR848728.2	4.591280501	2.198896575	2.02E-12	1.10E-11
ENSDARG00000092002	DHPS (2 of 2)	4.579931446	2.195326004	2.88E-06	9.56E-06
ENSDARG00000036359	minal	4.575651833	2.19397728	1.03E-176	3.52E-174
ENSDARG00000090531	ttc25	4.534910142	2.181073965	6.11E-10	2.78E-09
ENSDARG00000031051	il4r	4.514349043	2.17451797	1.24E-79	8.21E-78
ENSDARG00000086248	kcnj1a.3	4.511881617	2.173729215	1.40E-18	1.11E-17
ENSDARG00000014358	optc	4.485441114	2.165249872	4.30E-62	1.79E-60
ENSDARG00000090590	Metazoa_SRP	4.480367591	2.163617103	6.10E-06	1.96E-05
ENSDARG00000070945	zgc:163083	4.449037255	2.153493179	6.82E-07	2.41E-06
ENSDARG00000046012	slc47a1	4.449021126	2.153487949	2.19E-112	2.98E-110
ENSDARG00000059545	foxj1a	4.41229357	2.141528783	1.77E-141	3.93E-139
ENSDARG00000077960	si:ch211-186e20.7	4.39244343	2.135023706	2.21E-05	6.70E-05
ENSDARG00000092780	si:ch1073-170o4.1	4.387066883	2.133256701	0.000661287	0.001672226
ENSDARG00000068589	CABZ01079764.1	4.378106302	2.130306984	3.00E-177	1.04E-174
ENSDARG00000088158	Metazoa_SRP	4.33670398	2.116598969	0.000781892	0.001957866
ENSDARG00000060211	ankef1b	4.330502121	2.114534315	2.01E-08	8.10E-08
ENSDARG00000091234	CU019646.2	4.327116819	2.11340607	9.75E-49	2.76E-47
ENSDARG00000074253	olig3	4.322282316	2.111793307	1.56E-148	3.86E-146
ENSDARG00000009905	ntla	4.286285677	2.099728007	3.02E-97	3.02E-95
ENSDARG00000091768	CR932360.4	4.262742206	2.091781809	0.000911769	0.00226092
ENSDARG00000056795	serpine1	4.246684095	2.086336793	1.34E-108	1.68E-106

ENSDARG00000087235		4.237193927	2.083109159	1.35E-06	4.64E-06
ENSDARG00000091783	si:dkeyp-71f10.5	4.204426586	2.071909054	1.15E-49	3.35E-48
ENSDARG00000088775	NLRP6 (56 of 145)	4.201891934	2.071039059	4.16E-07	1.50E-06
ENSDARG00000057504		4.19091678	2.067265874	5.34E-78	3.36E-76
ENSDARG00000055527	cmn	4.188300046	2.066364799	2.06E-140	4.50E-138
ENSDARG00000075045	cxcl-c1c	4.171133422	2.06043946	2.44E-37	4.48E-36
ENSDARG00000067759	exd1	4.159104683	2.056272998	4.37E-06	1.42E-05
ENSDARG00000087543		4.147087301	2.052098418	2.92E-05	8.74E-05
ENSDARG00000074185	pcsk9	4.137040205	2.048598978	0.000149591	0.000412054
ENSDARG00000074745	CABZ01044297.1	4.135269309	2.047981288	2.93E-130	5.37E-128
ENSDARG00000074491	zgc:171759	4.130343517	2.046261774	1.88E-15	1.24E-14
ENSDARG00000091224	DUSP13 (2 of 3)	4.128245841	2.045528887	0.000436068	0.001130165
ENSDARG00000077739	col5a3a	4.116210707	2.041316835	5.44E-14	3.28E-13
ENSDARG00000076106	ftr43	4.094965898	2.033851438	0.00165469	0.003961801
ENSDARG00000088111	CU929417.2	4.094657447	2.033742763	9.53E-17	6.86E-16
ENSDARG00000009612	chia.3	4.074943731	2.026780138	0.001686411	0.004032655
ENSDARG00000093156	si:ch73-21g5.7	4.071028963	2.025393485	4.59E-173	1.52E-170
ENSDARG00000018566	flnca	4.07052276	2.025214086	7.03E-158	2.02E-155
ENSDARG00000092660	cyp27c1	4.0645666	2.023101528	2.16E-74	1.24E-72
ENSDARG00000089086	MUSTN1	4.062979933	2.022538239	4.15E-82	2.89E-80
ENSDARG00000087504	CABZ01045618.2	4.046277707	2.016595339	6.30E-16	4.28E-15
ENSDARG00000092665	si:ch1073-382c16.2	4.042482551	2.015241546	2.78E-102	3.03E-100
ENSDARG00000087854	ano9a	4.011715066	2.004219142	5.04E-08	1.96E-07
ENSDARG00000037804	phlda3	3.956578782	1.984253484	1.79E-125	3.05E-123
ENSDARG00000087264	Metazoa_SRP	3.955335854	1.983800201	1.17E-07	4.42E-07
ENSDARG00000078299	COLGALT2 (2 of 2)	3.949952357	1.981835252	7.38E-24	7.73E-23
ENSDARG00000059805	slc25a38a	3.943270942	1.979392841	5.98E-41	1.27E-39
ENSDARG00000071707	heatr1	3.943191301	1.979363703	7.37E-164	2.25E-161
ENSDARG00000089793	Metazoa_SRP	3.941717362	1.978824333	2.45E-05	7.38E-05
ENSDARG00000074378	junba	3.922687944	1.971842571	1.71E-122	2.83E-120

ENSDARG00000019741	si:ch211-152f23.5	3.917847987	1.970061422	8.17E-50	2.40E-48
ENSDARG00000010434	clu	3.900097866	1.963510326	1.82E-165	5.74E-163
ENSDARG00000069265	matn3b	3.882020682	1.956807804	1.84E-123	3.07E-121
ENSDARG00000087337	BX537263.2	3.874448734	1.953991055	0.001229479	0.002995214
ENSDARG00000086164	Metazoa_SRP	3.871124864	1.952752843	2.15E-07	7.98E-07
ENSDARG00000068680	ctrl	3.858290841	1.947961899	2.65E-07	9.75E-07
ENSDARG00000076241	txlnbb	3.845486706	1.943166205	4.02E-126	6.89E-124
ENSDARG00000018342	ggt1b	3.844859801	1.942930993	4.95E-23	4.99E-22
ENSDARG00000042982	cyp2p8	3.829936169	1.937320348	1.21E-10	5.83E-10
ENSDARG00000069726	glis3	3.82475331	1.935366699	2.11E-18	1.66E-17
ENSDARG00000083784	SNORD22	3.816541518	1.932265886	1.67E-11	8.54E-11
ENSDARG00000078294	si:ch211-113a14.14	3.801342464	1.926509004	3.99E-08	1.57E-07
ENSDARG00000075220	si:ch211-113a14.19	3.792683733	1.923219072	4.59E-07	1.65E-06
ENSDARG00000006409	mapk12b	3.789267045	1.921918816	2.44E-40	5.09E-39
ENSDARG00000055294	atoh1a	3.778949655	1.917985298	1.68E-30	2.34E-29
ENSDARG00000096496	si:dkey-261e22.5	3.778540913	1.917829244	5.64E-09	2.38E-08
ENSDARG00000090342	CCDC37 (2 of 2)	3.776182544	1.916928508	0.001427179	0.003446702
ENSDARG00000091265	wu:fe11b02	3.767584918	1.913640029	7.11E-06	2.27E-05
ENSDARG00000091685	Metazoa_SRP	3.76341126	1.912040953	8.01E-07	2.81E-06
ENSDARG00000027515	rrp1	3.739162501	1.902715171	3.73E-153	1.00E-150
ENSDARG00000091735	si:ch211-244h4.1	3.738407644	1.902423892	0.000300486	0.00079487
ENSDARG00000081931	SNORA16	3.738037775	1.902281148	6.72E-07	2.38E-06
ENSDARG00000075330	phox2ba	3.730095926	1.899212732	0.000106205	0.000298567
ENSDARG00000079002	dnai1	3.727915865	1.8983693	0.000104962	0.000295219
ENSDARG00000039957	rspo1	3.700928898	1.887887419	1.02E-43	2.46E-42
ENSDARG00000074897	hes2.1	3.691037011	1.884026204	2.62E-34	4.26E-33
ENSDARG00000094362	si:dkey-162b3.5	3.66402926	1.873431024	1.50E-05	4.64E-05
ENSDARG00000057648	dnttip2	3.663680078	1.873293529	1.73E-144	4.05E-142
ENSDARG00000088865	BX537263.9	3.642811131	1.865052198	1.56E-35	2.68E-34
ENSDARG00000055751	fosb	3.642738514	1.865023438	1.55E-29	2.07E-28

ENSDARG00000074904	DBNDD2	3.622929658	1.857156795	2.38E-43	5.65E-42
ENSDARG00000082753	AC024175.17	3.61882211	1.855520191	1.09E-74	6.30E-73
ENSDARG00000086751	zgc:112234	3.613930611	1.853568806	4.49E-06	1.46E-05
ENSDARG00000067831	tbata	3.612698549	1.853076878	1.53E-24	1.65E-23
ENSDARG00000074069	zgc:171452	3.604425061	1.849769155	3.89E-09	1.66E-08
ENSDARG00000079274	zgc:66382	3.596189773	1.846469155	1.89E-15	1.25E-14
ENSDARG00000063665	mat2al	3.595812544	1.846317813	8.04E-117	1.21E-114
ENSDARG00000075985	FP243385.1	3.587428737	1.842950173	1.17E-10	5.61E-10
ENSDARG00000043798	ms4a17a.1	3.580671719	1.840230256	3.07E-05	9.18E-05
ENSDARG00000097461	si:dkey-31b16.15	3.569011135	1.835524402	0.000626364	0.001588864
ENSDARG00000002259	ca15c	3.549830882	1.827750295	3.46E-17	2.55E-16
ENSDARG00000091525	si:ch211-113a14.14	3.546422969	1.826364611	3.08E-11	1.54E-10
ENSDARG00000042145	lhx8b	3.539630936	1.823598944	2.74E-05	8.23E-05
ENSDARG00000097592		3.538688768	1.82321488	0.000254505	0.00067899
ENSDARG00000031774	pus7	3.529859189	1.819610634	6.38E-144	1.49E-141
ENSDARG00000096905	si:ch73-23l24.1	3.520889656	1.815940014	1.47E-143	3.36E-141
ENSDARG00000088564	CABZ01045617.1	3.516528779	1.814152024	2.10E-39	4.23E-38
ENSDARG00000094292		3.514577273	1.813351175	0.000198663	0.000537632
ENSDARG00000015676	nmd3	3.508217361	1.810738136	1.89E-143	4.30E-141
ENSDARG00000044841	notch1a	3.500159388	1.80742062	5.92E-98	6.02E-96
ENSDARG00000058498	slc9a3.1	3.494412716	1.805050011	1.85E-15	1.22E-14
ENSDARG00000073720	RNF180 (2 of 2)	3.488643116	1.802666019	1.74E-82	1.22E-80
ENSDARG00000038131	SRF (1 of 2)	3.48637982	1.801729751	3.30E-106	3.91E-104
ENSDARG00000087053	DDX47 (2 of 2)	3.484677857	1.801025292	1.83E-68	8.98E-67
ENSDARG00000017835	brf1a	3.480221136	1.799178979	4.12E-82	2.87E-80
ENSDARG00000090352	si:dkey-97i18.5	3.462629946	1.791868214	7.49E-97	7.44E-95
ENSDARG00000078442	AL954696.1	3.456483349	1.789304976	3.65E-106	4.28E-104
ENSDARG00000008433	unc45b	3.453888937	1.788221692	2.75E-111	3.66E-109
ENSDARG00000081082	SNORA16	3.444840779	1.784437303	0.000232786	0.000623975
ENSDARG00000086605	si:dkey-8o9.5	3.444006967	1.784088061	0.002379708	0.005562703

ENSDARG00000077067	ddx47	3.427013562	1.776951901	2.23E-86	1.70E-84
ENSDARG00000083593	SNORA54	3.419762098	1.773895965	0.000106773	0.000300013
ENSDARG00000087359	c3b	3.419074527	1.77360587	3.37E-12	1.80E-11
ENSDARG00000086957	si:dkeyp-118a3.2	3.418871639	1.773520258	1.59E-50	4.78E-49
ENSDARG00000038147	hbbe3	3.414361748	1.771615918	3.38E-138	7.21E-136
ENSDARG00000086785		3.414360006	1.771615182	5.30E-08	2.06E-07
ENSDARG00000091005	si:dkeyp-80d11.11	3.41430632	1.771592498	0.002101352	0.004955655
ENSDARG00000091765	PLA2G4C (5 of 5)	3.401567932	1.766199901	5.59E-27	6.73E-26
ENSDARG00000076557	bicc2	3.390826169	1.761636826	1.32E-13	7.74E-13
ENSDARG00000087732	Metazoa_SRP	3.385052155	1.759178062	1.38E-105	1.60E-103
ENSDARG00000044488	pak1ip1	3.383870928	1.758674541	9.39E-131	1.76E-128
ENSDARG00000068850	fhit	3.38293296	1.758274587	7.97E-12	4.16E-11
ENSDARG00000036139	lctla	3.377527307	1.755967434	5.82E-132	1.14E-129
ENSDARG00000095925	si:dkey-258f14.7	3.373816901	1.75438168	2.65E-08	1.06E-07
ENSDARG00000093702	si:dkey-126g1.9	3.367804383	1.751808343	1.00E-105	1.16E-103
ENSDARG00000092833	si:dkeyp-1h4.8	3.36602957	1.751047851	7.80E-11	3.80E-10
ENSDARG00000062892	foxe3	3.365219433	1.750700581	3.50E-15	2.28E-14
ENSDARG00000095528	si:dkey-121n8.5	3.364063354	1.750204876	1.79E-16	1.26E-15
ENSDARG00000068934	cyp1b1	3.363176116	1.749824329	5.45E-69	2.71E-67
ENSDARG00000086654	CT027762.3	3.362550215	1.749555813	1.82E-06	6.15E-06
ENSDARG00000012215	umps	3.362107155	1.749365706	2.32E-131	4.48E-129
ENSDARG00000030700	ctps1a	3.361286113	1.74901335	6.25E-117	9.50E-115
ENSDARG00000069307	pomcb	3.35501778	1.746320412	2.40E-06	8.02E-06
ENSDARG00000057276	iqca1	3.354162211	1.745952461	4.10E-23	4.16E-22
ENSDARG00000086881	IER2	3.353501349	1.745668182	4.80E-91	4.02E-89
ENSDARG00000081050	SNORA21	3.352669694	1.745310356	0.00045684	0.001180555
ENSDARG00000007744	tsr1	3.350587959	1.744414281	3.95E-125	6.68E-123
ENSDARG00000096156		3.348844255	1.743663282	0.000456563	0.001180054
ENSDARG00000024511	bxdc2	3.345891567	1.742390692	9.05E-134	1.86E-131
ENSDARG00000097465	TRMT61B	3.338710875	1.739291165	2.37E-60	9.46E-59

ENSDARG00000017675	cirh1a	3.336236584	1.738221599	1.33E-130	2.46E-128
ENSDARG00000088881	si:dkeyp-69b9.3	3.332759491	1.736717209	1.49E-132	2.97E-130
ENSDARG00000001754	noc2l	3.327816216	1.734575761	3.09E-130	5.63E-128
ENSDARG00000051804	BX664721.1	3.325496486	1.733569747	0.000872143	0.002168755
ENSDARG00000004328	CABZ01041200.1	3.323623658	1.732757032	4.05E-09	1.73E-08
ENSDARG00000090581	FP017279.3	3.321575197	1.731867576	3.07E-17	2.26E-16
ENSDARG00000056029	cyp26c1	3.319711691	1.731057952	1.23E-128	2.21E-126
ENSDARG00000075991	COL5A3	3.309555527	1.726637476	2.94E-07	1.08E-06
ENSDARG00000045071	chad	3.305054894	1.724674234	2.05E-118	3.19E-116
ENSDARG00000079370	utp18	3.299350746	1.722182156	1.81E-131	3.52E-129
ENSDARG00000087694	wu:fe11b02	3.294871597	1.720222244	0.000982411	0.002427028
ENSDARG00000096716	HMX1	3.292889722	1.719354197	5.95E-05	0.000172035
ENSDARG00000078419	filip1a	3.289593764	1.717909435	1.12E-15	7.52E-15
ENSDARG00000052207	c3c	3.285742566	1.716219451	7.69E-129	1.39E-126
ENSDARG00000058549	smyd1b	3.280815599	1.714054508	2.04E-126	3.51E-124
ENSDARG00000091253	CU469500.4	3.279608318	1.713523525	2.54E-130	4.69E-128
ENSDARG00000007823	atf3	3.27840247	1.712992976	1.08E-113	1.51E-111
ENSDARG00000079105	mhc2dab	3.277489202	1.712591027	0.002471879	0.005768665
ENSDARG00000044853	rrp7a	3.277301587	1.71250844	3.61E-83	2.58E-81
ENSDARG00000096906	si:dkey-7i4.24	3.276734219	1.712258658	0.001916964	0.004551039
ENSDARG00000045372	ngdn	3.274985751	1.711488629	1.88E-106	2.25E-104
ENSDARG00000082634	SNORD72	3.268893819	1.708802516	5.17E-05	0.000150659
ENSDARG00000042839	dph6	3.262349289	1.705911255	1.40E-78	8.97E-77
ENSDARG00000019791	prmt3	3.257443995	1.703740375	2.56E-100	2.70E-98
ENSDARG00000088277	susd5	3.251503755	1.70110709	2.42E-22	2.35E-21
ENSDARG00000096322	si:ch73-379f7.4	3.243071414	1.697360793	0.000315433	0.000832704
ENSDARG00000089347		3.240351504	1.696150321	3.34E-91	2.84E-89
ENSDARG00000041895	cad	3.239264168	1.695666127	1.23E-50	3.69E-49
ENSDARG00000040479	cga	3.23792408	1.695069159	1.57E-08	6.39E-08
ENSDARG00000032584	thnsl2	3.236927772	1.694625174	8.66E-11	4.20E-10

ENSDARG00000058738	SLC26A6 (3 of 4)	3.227832617	1.690565768	1.84E-11	9.38E-11
ENSDARG00000043666	abt1	3.227784851	1.690544418	1.85E-91	1.60E-89
ENSDARG00000033443	mdm2	3.223400626	1.688583507	1.12E-92	9.98E-91
ENSDARG00000017354	epha2a	3.222563241	1.688208671	7.31E-127	1.28E-124
ENSDARG00000008852	elp4	3.210665575	1.682872401	4.30E-72	2.31E-70
ENSDARG00000043884	nup43	3.204975865	1.680313493	2.58E-97	2.60E-95
ENSDARG00000091156	cyp2aa12	3.196473968	1.676481344	5.95E-07	2.11E-06
ENSDARG00000056374	noxo1b	3.190951003	1.673986456	7.61E-11	3.71E-10
ENSDARG00000059390	slc12a1	3.186158647	1.671818104	1.36E-18	1.08E-17
ENSDARG00000088976	BX537263.10	3.186076597	1.671780951	5.61E-09	2.37E-08
ENSDARG00000029982	TM6SF2 (2 of 2)	3.177023907	1.667675946	3.95E-91	3.34E-89
ENSDARG00000007130	mrto4	3.175975849	1.667199942	4.05E-114	5.70E-112
ENSDARG00000089960	si:ch1073-288h17.2	3.17209512	1.665436033	2.92E-09	1.26E-08
ENSDARG00000017605	rpp40	3.160743081	1.660263771	8.37E-53	2.71E-51
ENSDARG00000040503	CABZ01070258.1	3.159863217	1.659862109	3.59E-110	4.66E-108
ENSDARG00000032768	irf1b	3.156463319	1.658308986	1.33E-24	1.43E-23
ENSDARG00000028323	mybbp1a	3.155772518	1.657993213	1.57E-111	2.11E-109
ENSDARG00000078081	BX957353.1	3.154351125	1.657343262	2.73E-30	3.78E-29
ENSDARG00000001937	CABZ01043017.1	3.154010224	1.657187337	8.29E-13	4.61E-12
ENSDARG00000091382	HIST1H4E	3.149452825	1.655101202	0.007816723	0.016877921
ENSDARG00000056130	neurog1	3.148546341	1.654685902	1.36E-109	1.75E-107
ENSDARG00000075953	ddx24	3.144784686	1.652961244	2.98E-122	4.90E-120
ENSDARG00000071652	RRP8	3.143359564	1.652307309	5.12E-84	3.70E-82
ENSDARG00000086515	rad21a	3.140614824	1.651047017	1.21E-115	1.77E-113
ENSDARG00000016789		3.133719876	1.647876223	6.72E-14	4.03E-13
ENSDARG00000086310	C12H10orf107	3.133689188	1.647862095	1.91E-08	7.71E-08
ENSDARG00000019547	aaas	3.133095684	1.64758883	3.13E-113	4.34E-111
ENSDARG00000094161	si:ch73-265d7.2	3.131743436	1.646966027	1.97E-33	3.10E-32
ENSDARG00000019659	foxi1	3.12949181	1.645928401	1.22E-32	1.85E-31
ENSDARG00000076766	zgc:163061	3.126004219	1.644319725	5.83E-06	1.87E-05

ENSDARG00000023526	ggt1a	3.122576875	1.64273709	1.19E-09	5.29E-09
ENSDARG00000094712	si:dkeyp-20e4.9	3.112487934	1.638068245	0.000223604	0.00060118
ENSDARG00000022410	rrp12	3.109423378	1.636647067	2.39E-83	1.71E-81
ENSDARG00000075693	NUFIP1	3.109252851	1.636567944	1.73E-60	6.90E-59
ENSDARG00000092285	cenpv	3.108443704	1.636192451	5.52E-22	5.25E-21
ENSDARG00000020298	btg2	3.107128735	1.635582017	9.44E-95	8.97E-93
ENSDARG00000054562	her15.1	3.106773476	1.635417054	1.82E-119	2.89E-117
ENSDARG00000090401	CABZ01020840.1	3.104408105	1.634318227	1.02E-55	3.64E-54
ENSDARG00000073901	CABZ01046997.2	3.101357395	1.632899789	0.007125568	0.015502203
ENSDARG00000010791	dla	3.099414966	1.631995923	2.47E-75	1.45E-73
ENSDARG00000033400	atoh1b	3.095580953	1.630210188	1.06E-45	2.72E-44
ENSDARG00000034423	sncga	3.089850408	1.627536993	3.91E-118	5.97E-116
ENSDARG00000012495	mphosph10	3.088174196	1.626754133	3.14E-118	4.85E-116
ENSDARG00000078643	VTCN1	3.074417417	1.620313055	4.45E-06	1.45E-05
ENSDARG00000071669	smyd5	3.071513599	1.618949771	4.06E-86	3.05E-84
ENSDARG00000091066	zgc:112234	3.070135186	1.618302182	0.000396814	0.001034468
ENSDARG00000078683	RNF14 (3 of 5)	3.066352358	1.616523488	2.80E-63	1.20E-61
ENSDARG00000088340	spag1b	3.064651299	1.615722931	4.59E-05	0.0001345
ENSDARG00000022232	ppan	3.064112087	1.615469073	1.44E-116	2.16E-114
ENSDARG00000058160	tnfaip2b	3.062215058	1.614575606	1.27E-22	1.25E-21
ENSDARG00000075482	zgc:112234	3.059259487	1.613182482	4.39E-07	1.58E-06
ENSDARG00000045254	C24H7orf50	3.057704797	1.61244913	2.08E-59	8.16E-58
ENSDARG00000002293	si:ch211-197g15.9	3.055222628	1.61127751	7.42E-06	2.36E-05
ENSDARG00000005772	tsr2	3.054744119	1.611051538	1.68E-114	2.38E-112
ENSDARG00000077559		3.044756731	1.606326965	1.17E-115	1.72E-113
ENSDARG00000020219	dld	3.044369737	1.606143584	9.10E-99	9.37E-97
ENSDARG00000008740	esf1	3.041709547	1.604882397	1.15E-115	1.71E-113
ENSDARG00000037158	rcc1	3.040421324	1.604271258	2.56E-115	3.74E-113
ENSDARG00000054560	ZGC:136520	3.037153587	1.602719867	2.92E-74	1.67E-72
ENSDARG00000055160	chadla	3.02966913	1.599160246	2.28E-44	5.64E-43

ENSDARG00000093381	tgm2l	3.024911822	1.596893088	2.69E-60	1.07E-58
ENSDARG00000077129	notch1a	3.019619471	1.594366754	3.11E-109	3.96E-107
ENSDARG00000015476	iqch	3.01550508	1.592399666	2.68E-07	9.84E-07
ENSDARG00000090203	ORC2	3.001403677	1.585637369	7.91E-57	2.87E-55
ENSDARG00000088420		2.99601449	1.583044601	0.000517983	0.00132937
ENSDARG00000088209	Metazoa_SRP	2.995962659	1.583019642	0.000653853	0.00165405
ENSDARG00000055365	si:dkey-25e12.3	2.991485113	1.580861884	1.14E-13	6.72E-13
ENSDARG00000086218	nkd3	2.990431573	1.580353706	0.003331133	0.00763294
ENSDARG00000091831	ddx27	2.989410936	1.579861229	7.80E-112	1.05E-109
ENSDARG00000091527	CABZ01068153.1	2.988487009	1.579415272	2.35E-10	1.10E-09
ENSDARG00000076815	eif3s10	2.985476123	1.577961031	6.41E-09	2.70E-08
ENSDARG00000079938	zgc:173594	2.984553874	1.577515296	2.72E-58	1.03E-56
ENSDARG00000053227	hamp2	2.983039309	1.576782989	1.15E-18	9.17E-18
ENSDARG00000045565	noc4l	2.980566602	1.575586611	1.06E-95	1.03E-93
ENSDARG00000097655	si:dkey-30e9.6	2.976238033	1.573489914	8.23E-11	4.00E-10
ENSDARG00000016484	dkc1	2.974859292	1.572821432	5.06E-87	3.90E-85
ENSDARG00000010478	hsp90aa1.1	2.974429637	1.57261305	5.76E-42	1.28E-40
ENSDARG00000073747	si:dkey-1j5.4	2.972693896	1.571770914	1.50E-32	2.27E-31
ENSDARG00000089060	kcnj1a.5	2.972484934	1.571669498	8.54E-25	9.34E-24
ENSDARG00000022020	FOCAD	2.969472563	1.570206702	3.03E-90	2.48E-88
ENSDARG00000052895	htra3a	2.968285628	1.569629924	0.00038681	0.001009409
ENSDARG00000051735	si:ch211-113a14.18	2.967967624	1.569475354	0.009075857	0.019374329
ENSDARG00000087775	Metazoa_SRP	2.967522551	1.569258994	0.007869596	0.01698563
ENSDARG00000077928	ccdc173	2.966549628	1.568785918	8.20E-27	9.81E-26
ENSDARG00000056744	ela2	2.959524526	1.565365413	3.30E-18	2.57E-17
ENSDARG00000059075	nip7	2.958450903	1.564841953	3.31E-85	2.44E-83
ENSDARG00000079497	C5H8orf4 (2 of 2)	2.945495288	1.558510245	4.20E-109	5.33E-107
ENSDARG00000033029	pkd1b	2.944875776	1.558206778	1.69E-65	7.66E-64
ENSDARG00000091471	ECT2L	2.943648322	1.557605323	1.07E-23	1.11E-22
ENSDARG00000079909	zgc:171759	2.94288804	1.557232657	0.000532317	0.001362832

ENSDARG00000087353	CABZ01057633.1	2.941367487	1.556487042	3.99E-07	1.44E-06
ENSDARG00000040730	wdr75	2.940633044	1.556126764	2.06E-108	2.58E-106
ENSDARG00000087779	im:7148292	2.93963072	1.555634933	8.05E-106	9.37E-104
ENSDARG00000041991	rrp9	2.935584332	1.553647702	2.05E-100	2.18E-98
ENSDARG00000025467	aatf	2.930843273	1.551315822	7.21E-103	7.94E-101
ENSDARG00000005001	grem2	2.928877627	1.550347917	5.53E-51	1.68E-49
ENSDARG00000087021	CABZ01085878.1	2.928294692	1.550060748	2.18E-67	1.05E-65
ENSDARG00000093423	swsap1	2.927618414	1.549727525	4.74E-05	0.00013889
ENSDARG00000026611	socs3b	2.925058232	1.548465346	2.39E-106	2.85E-104
ENSDARG00000077481	si:dkey-76k16.6	2.924226159	1.548054893	3.77E-25	4.18E-24
ENSDARG00000024561	nolc1	2.920845572	1.546386084	1.10E-77	6.87E-76
ENSDARG00000018902	pes	2.919888513	1.545913285	6.38E-100	6.64E-98
ENSDARG00000097269	si:ch211-222n22.1	2.91869618	1.545324042	0.007267494	0.015775942
ENSDARG00000056732	her4.1	2.917428477	1.544697288	3.78E-63	1.62E-61
ENSDARG00000088392	si:dkey-238o14.9	2.917387555	1.544677051	3.10E-05	9.25E-05
ENSDARG00000074160	PAQR4 (1 of 2)	2.916253985	1.544116374	4.45E-05	0.000130624
ENSDARG00000091749	fsip1	2.916219515	1.544099321	4.51E-11	2.23E-10
ENSDARG00000035696	fam203a	2.908293184	1.540172714	1.41E-43	3.38E-42
ENSDARG00000069245	matn3a	2.907875134	1.53996532	4.67E-94	4.37E-92
ENSDARG00000090635	kcnj1a.4	2.906193797	1.539130911	5.33E-11	2.62E-10
ENSDARG00000056043	lama1	2.901300094	1.536699527	1.20E-95	1.16E-93
ENSDARG00000068903	sdad1	2.900696615	1.536399411	3.38E-106	3.98E-104
ENSDARG00000078878	METTL21C (2 of 2)	2.898019455	1.53506728	2.97E-13	1.71E-12
ENSDARG00000090337	pprc1	2.897856474	1.534986143	1.42E-106	1.71E-104
ENSDARG00000086848	atad3b	2.896447676	1.534284603	2.42E-103	2.70E-101
ENSDARG00000042186	paplnb	2.895654618	1.533889534	0.001791501	0.004267789
ENSDARG00000037002	zgc:152968	2.894934689	1.533530801	0.007358252	0.015957108
ENSDARG00000077911	treh	2.891549508	1.531842803	0.006386219	0.014019289
ENSDARG00000090375	BX001033.2	2.890689651	1.531413727	2.72E-24	2.90E-23
ENSDARG00000058372	mcph1	2.889873188	1.531006187	6.78E-25	7.44E-24

ENSDARG00000009806	smfn	2.888492994	1.530316995	1.35E-78	8.62E-77
ENSDARG00000089570	ccdc169	2.884951941	1.528547286	1.09E-05	3.40E-05
ENSDARG00000059171	stpg1	2.884582492	1.528362522	6.79E-05	0.000195419
ENSDARG00000087437	crygn1	2.882673526	1.527407455	1.02E-10	4.90E-10
ENSDARG00000006434	polr1e	2.87353207	1.522825151	3.85E-89	3.07E-87
ENSDARG00000011931	dph5	2.872222565	1.522167546	9.71E-89	7.69E-87
ENSDARG00000093877	MFAP4 (10 of 14)	2.871559924	1.521834668	0.002290279	0.005368673
ENSDARG00000062152	chaf1a	2.870429976	1.521266862	2.02E-71	1.08E-69
ENSDARG00000052344	tbl3	2.870382156	1.521242827	1.39E-98	1.42E-96
ENSDARG00000023952	fam167ab	2.869775272	1.520937766	5.54E-47	1.48E-45
ENSDARG00000027428	rpp30	2.869662406	1.520881025	3.00E-41	6.46E-40
ENSDARG00000028688	exosc2	2.864311345	1.518188319	9.35E-94	8.65E-92
ENSDARG00000038969	zgc:113142	2.863283753	1.517670649	2.14E-10	1.01E-09
ENSDARG00000019001	ak7a	2.859874106	1.51595164	7.15E-43	1.67E-41
ENSDARG00000023299	nhp2l1b	2.859851539	1.515940256	5.12E-101	5.47E-99
ENSDARG00000081270	rn7sk	2.859816507	1.515922583	1.42E-53	4.71E-52
ENSDARG00000014329	npm1a	2.858832972	1.515426333	4.12E-49	1.18E-47
ENSDARG00000024416	twistnb	2.854205173	1.513089046	2.88E-70	1.49E-68
ENSDARG00000076330	dusp12	2.85247919	1.512216362	3.91E-31	5.61E-30
ENSDARG00000038538	ccdc37	2.851567816	1.511755343	1.13E-10	5.42E-10
ENSDARG00000070477	dnajc2	2.850908227	1.511421599	6.13E-103	6.80E-101
ENSDARG00000091075	HGH1 (2 of 2)	2.850178531	1.51105229	4.85E-34	7.80E-33
ENSDARG00000032963	her12	2.849133326	1.510523134	7.60E-91	6.31E-89
ENSDARG00000038312	rcl1	2.846348377	1.50911225	9.30E-86	6.90E-84
ENSDARG00000004232	dlb	2.842510474	1.507165665	6.15E-75	3.57E-73
ENSDARG00000043960	rpf2	2.842170065	1.506992882	1.13E-100	1.20E-98
ENSDARG00000061081	arpp21	2.838165454	1.504958695	1.28E-90	1.06E-88
ENSDARG00000088436	CT956064.3	2.837362533	1.504550497	1.69E-81	1.16E-79
ENSDARG00000004082	cx39.9	2.837169657	1.504452424	4.48E-91	3.77E-89
ENSDARG00000005616	bfb	2.836077018	1.503896712	6.27E-08	2.42E-07

ENSDARG00000059269	PRTG (2 of 2)	2.835463004	1.503584333	2.97E-13	1.71E-12
ENSDARG00000021579	orc5	2.83248785	1.502069768	2.87E-69	1.43E-67
ENSDARG00000077571	zgc:174862	2.831593563	1.501614201	5.76E-64	2.50E-62
ENSDARG00000091068	CABZ01063789.1	2.83107113	1.501347997	1.97E-13	1.14E-12
ENSDARG00000035427	surf6	2.8242934	1.49788997	3.44E-81	2.34E-79
ENSDARG00000008805	orc3	2.823763296	1.497619159	1.20E-89	9.65E-88
ENSDARG00000022728	rbm34	2.813379207	1.492304022	5.01E-92	4.42E-90
ENSDARG00000095115	zgc:171759	2.81098995	1.491078296	0.001138075	0.002785676
ENSDARG00000077346	CABZ01064799.1	2.807210423	1.48913721	0.008295466	0.017828976
ENSDARG00000069563		2.801270855	1.486081484	1.69E-10	8.02E-10
ENSDARG00000035257	akr1a1a	2.800211452	1.485535773	2.70E-58	1.03E-56
ENSDARG00000063627	bop1	2.795807462	1.48326501	6.77E-100	7.02E-98
ENSDARG00000097883	si:ch211-196c10.14	2.792865168	1.481745926	8.80E-17	6.34E-16
ENSDARG00000058337	nop58	2.79191505	1.481255045	2.72E-68	1.33E-66
ENSDARG00000077264	wdr43	2.789256832	1.479880782	4.68E-73	2.61E-71
ENSDARG00000040284	si:dkey-79d12.5	2.782828891	1.476552204	3.87E-48	1.07E-46
ENSDARG00000017360	col5a3a	2.782623441	1.476445689	9.74E-39	1.90E-37
ENSDARG00000007918	ttc27	2.776402949	1.473216967	5.19E-97	5.17E-95
ENSDARG00000091931	gpatch4	2.775620588	1.472810373	5.88E-56	2.12E-54
ENSDARG00000000516	ankar	2.773671301	1.471796828	8.33E-06	2.63E-05
ENSDARG00000006863	CCDC108	2.773358675	1.471634211	4.14E-17	3.04E-16
ENSDARG00000097044	si:dkey-14d8.24	2.771358903	1.470593558	1.11E-05	3.48E-05
ENSDARG00000038668	gbp1	2.770357439	1.470072129	4.58E-50	1.35E-48
ENSDARG00000008174	hoxb1a	2.769964238	1.46986735	3.48E-23	3.54E-22
ENSDARG00000078214	MYBBP1A (2 of 2)	2.765072292	1.4673172	4.77E-51	1.45E-49
ENSDARG00000087315	CT956064.2	2.764166071	1.466844295	0.008344416	0.017923337
ENSDARG00000036076	heatr3	2.762794305	1.466128155	8.32E-96	8.13E-94
ENSDARG00000026218	slc1a3a	2.756369127	1.462769104	8.07E-89	6.41E-87
ENSDARG00000038027	nol12	2.754159367	1.461612042	2.77E-63	1.19E-61
ENSDARG00000090419	CABZ01078499.2	2.752693842	1.46084416	0.000355386	0.000932133

ENSDARG00000075654	ccdc135	2.750127706	1.459498614	2.01E-28	2.55E-27
ENSDARG00000092272	si:dkey-248g17.3	2.749788836	1.459320834	3.69E-11	1.84E-10
ENSDARG00000070127	nfxl1	2.749774415	1.459313268	1.23E-95	1.18E-93
ENSDARG00000075157	bicc2	2.749349041	1.459090075	1.40E-31	2.05E-30
ENSDARG00000030893	zgc:154142	2.748269364	1.458523413	0.000412786	0.00107384
ENSDARG00000044676	GADD45G (3 of 3)	2.744692383	1.456644465	6.35E-52	1.99E-50
ENSDARG00000097984	si:ch211-277b21.2	2.737606775	1.452915236	0.001370523	0.003319184
ENSDARG00000053225	gnl2	2.737560931	1.452891076	1.22E-96	1.20E-94
ENSDARG00000041493	ipo4	2.734768136	1.451418521	1.22E-96	1.20E-94
ENSDARG00000079720	HPDL	2.733009551	1.450490501	1.46E-43	3.49E-42
ENSDARG00000037402	lim2.3	2.732885601	1.45042507	6.23E-91	5.21E-89
ENSDARG00000086810	msh3	2.731741536	1.449820989	3.59E-19	2.96E-18
ENSDARG00000091003	il34	2.731515333	1.449701521	0.006957803	0.015168577
ENSDARG00000016999	lin28a	2.725983218	1.44677668	1.12E-37	2.08E-36
ENSDARG00000010591	foxn4	2.723425651	1.445422482	1.51E-93	1.37E-91
ENSDARG00000061480		2.718066222	1.442580606	0.002483446	0.005791853
ENSDARG00000062612	dhx37	2.716569075	1.441785731	8.05E-92	7.02E-90
ENSDARG00000090840		2.712996957	1.43988743	2.84E-06	9.42E-06
ENSDARG00000030722	xirp1	2.711367213	1.439020517	8.03E-95	7.66E-93
ENSDARG00000045348	NUP54	2.710055094	1.438322181	1.33E-93	1.22E-91
ENSDARG00000056888	DNAH8	2.709980929	1.438282699	1.79E-41	3.90E-40
ENSDARG00000087309	CABZ01045618.1	2.709738081	1.438153409	1.88E-14	1.17E-13
ENSDARG00000041492	si:ch211-196f5.2	2.709285119	1.437912228	4.70E-09	2.00E-08
ENSDARG00000036070	znrd1	2.709103812	1.437815678	2.03E-35	3.47E-34
ENSDARG00000028976	pus3	2.707176603	1.436789005	1.20E-36	2.15E-35
ENSDARG00000057080	dnajc21	2.703432822	1.434792507	6.60E-83	4.69E-81
ENSDARG00000091983	si:ch73-249k16.1	2.701400735	1.43370767	9.15E-20	7.79E-19
ENSDARG00000076843	BX649262.1	2.700925396	1.433453791	2.40E-07	8.86E-07
ENSDARG00000016391	calcoco1b	2.700425316	1.433186649	2.96E-05	8.86E-05
ENSDARG00000029596	trim55a	2.697495675	1.431620647	5.77E-86	4.31E-84

ENSDARG00000008333	znfl2a	2.694575689	1.430058112	6.72E-15	4.30E-14
ENSDARG00000002487	noc3l	2.694560176	1.430049806	3.34E-93	2.98E-91
ENSDARG00000022560	clcnk	2.690312454	1.427773737	9.92E-11	4.79E-10
ENSDARG00000069675	her8.2	2.689959122	1.427584249	6.91E-55	2.40E-53
ENSDARG00000073876		2.689643328	1.427414871	2.14E-11	1.09E-10
ENSDARG00000092033	si:dkey-239h2.3	2.689478323	1.427326361	3.72E-37	6.79E-36
ENSDARG00000003564	dohh	2.689099928	1.427123367	2.88E-68	1.41E-66
ENSDARG00000039486	bag3	2.688492059	1.42679721	3.87E-83	2.76E-81
ENSDARG00000005574	vsx2	2.686833545	1.425906946	8.03E-92	7.02E-90
ENSDARG00000030789	ddx18	2.686362659	1.425654082	2.86E-82	2.01E-80
ENSDARG00000037178	zic2b	2.681098286	1.422824108	1.33E-83	9.58E-82
ENSDARG00000076761	ftsj	2.680327618	1.422409353	3.35E-89	2.68E-87
ENSDARG00000043323	lnx1	2.678658266	1.421510539	7.30E-92	6.42E-90
ENSDARG00000090904	RNF219	2.677733601	1.421012439	9.94E-20	8.44E-19
ENSDARG00000062262	ednrab	2.677384209	1.420824183	2.58E-08	1.03E-07
ENSDARG00000029722	hmgb2a	2.676224415	1.420199098	0.004910356	0.01096236
ENSDARG00000040557	exosc5	2.673855563	1.418921536	4.09E-58	1.54E-56
ENSDARG00000035253	npr3	2.671296195	1.417539952	3.79E-90	3.09E-88
ENSDARG00000089658	si:dkey-26i24.1	2.667946698	1.415729844	0.005264764	0.011703
ENSDARG00000045562	pus1	2.665676462	1.414501689	6.40E-57	2.34E-55
ENSDARG00000020133	jdp2	2.66237112	1.412711689	3.16E-39	6.32E-38
ENSDARG00000068657	irgq2	2.661764169	1.412382755	1.09E-05	3.41E-05
ENSDARG00000019742	tlr4ba	2.655970035	1.40923887	0.000584432	0.001488079
ENSDARG00000077567	angel1	2.655454809	1.408958977	9.60E-10	4.31E-09
ENSDARG00000078533	poc1a	2.653393138	1.407838447	3.23E-20	2.82E-19
ENSDARG00000037109	pwp2h	2.652531342	1.407369798	2.23E-90	1.83E-88
ENSDARG00000056617	rpgra	2.64679812	1.40424816	6.85E-05	0.000196913
ENSDARG00000069583	CNDP1	2.645605518	1.40359796	2.72E-64	1.19E-62
ENSDARG00000086647	chrng	2.645258241	1.403408571	4.88E-43	1.15E-41
ENSDARG00000063076	atrip	2.644154418	1.402806432	6.58E-75	3.82E-73

ENSDARG00000056729	her4.2	2.641798598	1.401520485	2.86E-74	1.64E-72
ENSDARG00000079462	PTGES3L	2.64086612	1.401011165	1.09E-60	4.38E-59
ENSDARG00000015921	pwp1	2.636684504	1.398724954	2.25E-87	1.74E-85
ENSDARG00000069977	C11H12orf10	2.632895902	1.396650482	2.64E-84	1.92E-82
ENSDARG00000086481	mogat3a	2.632388155	1.396372236	1.80E-51	5.55E-50
ENSDARG00000041617	nme5	2.627502926	1.393692371	1.09E-13	6.45E-13
ENSDARG00000077387	tcte1	2.624784677	1.392199077	0.000283095	0.000750825
ENSDARG00000096037	si:ch211-214j24.14	2.621461484	1.390371349	1.28E-69	6.47E-68
ENSDARG00000097058	si:ch211-60j9.3	2.620845572	1.390032348	2.29E-08	9.20E-08
ENSDARG00000038374	ccdc137	2.620433464	1.389805478	1.62E-72	8.85E-71
ENSDARG00000038664	pole2	2.617897359	1.388408534	1.69E-54	5.81E-53
ENSDARG00000020354	lmx1a	2.617415662	1.388143052	0.000554776	0.00141663
ENSDARG00000097762	si:dkey-33c14.2	2.6169239	1.387871972	0.014938391	0.030714561
ENSDARG00000094570	si:ch211-226h7.2	2.616275019	1.387514203	1.49E-07	5.59E-07
ENSDARG00000078347	DCSTAMP	2.615886849	1.387300138	0.010325682	0.021842139
ENSDARG00000043322	gsx2	2.615329682	1.38699282	2.00E-42	4.57E-41
ENSDARG00000053117	cpeb1a	2.607340519	1.382579011	0.017207261	0.034959593
ENSDARG00000045625	thumpd1	2.606481213	1.382103461	3.06E-74	1.74E-72
ENSDARG00000051762	DNAJA4	2.606366272	1.38203984	9.13E-87	7.00E-85
ENSDARG00000088132	HEATR2 (2 of 2)	2.603618255	1.380517935	1.01E-19	8.60E-19
ENSDARG00000042900	gtpbp1l	2.602346483	1.379813059	3.68E-79	2.39E-77
ENSDARG00000044325	zgc:193690	2.601215758	1.379186069	2.29E-11	1.16E-10
ENSDARG00000020642	gtdc1	2.600868705	1.378993572	1.71E-38	3.30E-37
ENSDARG00000077193	nags	2.600793053	1.378951607	5.44E-10	2.49E-09
ENSDARG00000043304	nop2	2.597169025	1.376939909	6.69E-86	4.97E-84
ENSDARG00000078579	zgc:175280	2.59615478	1.376376398	1.34E-13	7.86E-13
ENSDARG00000060127	adamts3	2.594288879	1.375339135	1.67E-60	6.71E-59
ENSDARG00000062502	CU571162.1	2.59371893	1.375022149	4.36E-11	2.16E-10
ENSDARG00000040478	lrp2bp	2.59003877	1.372973694	2.92E-05	8.74E-05
ENSDARG00000040623	fosl2	2.589337589	1.372583071	3.62E-85	2.67E-83

ENSDARG00000004806	grwd1	2.589067069	1.372432338	1.38E-80	9.31E-79
ENSDARG00000077760	ascc1	2.588549912	1.372144136	3.51E-22	3.38E-21
ENSDARG00000089922	DSN1	2.585711488	1.370561309	6.38E-69	3.17E-67
ENSDARG00000086850	CABZ01089385.1	2.584701231	1.369997527	1.57E-15	1.04E-14
ENSDARG00000037570	polr1d	2.579614833	1.36715567	4.58E-50	1.35E-48
ENSDARG00000068884	FBXO16 (2 of 2)	2.577712619	1.366091431	0.013045921	0.027126533
ENSDARG00000052610	olig4	2.576734638	1.365543971	1.18E-50	3.54E-49
ENSDARG00000007531	zcchc9	2.576190855	1.365239478	4.61E-51	1.41E-49
ENSDARG00000057105	nle1	2.574369197	1.364218969	1.06E-65	4.85E-64
ENSDARG00000094388	celsr2	2.573937665	1.363977115	5.54E-24	5.83E-23
ENSDARG00000088984	azi1	2.569795272	1.361653429	4.59E-80	3.06E-78
ENSDARG00000091905	si:dkey-108k21.21	2.569678114	1.361587654	0.002827992	0.006549167
ENSDARG00000078728	zgc:173710	2.566832985	1.359989428	1.69E-07	6.31E-07
ENSDARG00000009822	her4.4	2.566249926	1.35966168	9.71E-57	3.52E-55
ENSDARG00000034559	srpx2	2.564989756	1.358953064	6.39E-09	2.69E-08
ENSDARG00000052480	pdcd11	2.562020874	1.35728223	4.57E-67	2.18E-65
ENSDARG00000097914	si:dkey-25e11.13	2.56155329	1.357018905	0.004405152	0.009909237
ENSDARG00000036816	pou2f2a	2.560300804	1.356313319	2.86E-72	1.55E-70
ENSDARG00000013926	slc16a9a	2.560033297	1.356162575	2.65E-27	3.23E-26
ENSDARG00000044671	wfikkn1	2.559563279	1.355897674	0.005097316	0.011350317
ENSDARG00000005128	rnaseh2b	2.558911221	1.355530095	3.66E-74	2.07E-72
ENSDARG00000059386	dtwd1	2.558383232	1.355232388	8.28E-34	1.32E-32
ENSDARG00000075551	si:ch211-274p24.3	2.554256636	1.352903485	1.72E-51	5.32E-50
ENSDARG00000068288	LAMC2	2.553988887	1.352752247	2.60E-27	3.17E-26
ENSDARG00000021913	ak9	2.551937235	1.351592847	2.98E-19	2.47E-18
ENSDARG00000076927	DUSP27	2.551413938	1.351296979	8.78E-85	6.43E-83
ENSDARG00000035507	ddx31	2.548888172	1.349868079	3.15E-48	8.75E-47
ENSDARG00000001057	bysl	2.548334388	1.349554598	1.35E-69	6.81E-68
ENSDARG00000087815	pop1	2.548129138	1.349438394	1.73E-37	3.19E-36
ENSDARG00000075261	timp2b	2.548061452	1.349400072	4.35E-80	2.91E-78

ENSDARG00000090786	ybey	2.547944871	1.349334063	1.82E-15	1.20E-14
ENSDARG00000007217	wdr55	2.547653832	1.349169262	2.75E-40	5.73E-39
ENSDARG00000031795	abcf1	2.544166794	1.347193256	3.55E-37	6.50E-36
ENSDARG00000010978	trmt1	2.541256715	1.345542122	1.66E-55	5.88E-54
ENSDARG00000025017	prdm8	2.540523445	1.345125778	5.29E-66	2.44E-64
ENSDARG00000060634	azi1	2.540141572	1.344908906	1.05E-42	2.42E-41
ENSDARG00000006978	zak	2.536027245	1.342570245	3.42E-19	2.83E-18
ENSDARG00000089836	MRPS12 (2 of 2)	2.53510059	1.342042993	3.00E-30	4.12E-29
ENSDARG00000067701	myoz3a	2.534125214	1.341487811	3.60E-46	9.36E-45
ENSDARG00000054540	imp4	2.53354548	1.341157727	4.85E-66	2.24E-64
ENSDARG00000005619	nek2	2.531070044	1.339747433	1.98E-41	4.30E-40
ENSDARG00000011079	wdr3	2.529681556	1.338955785	1.04E-75	6.22E-74
ENSDARG00000086411	SRSF10 (2 of 2)	2.527989616	1.337990537	1.66E-80	1.12E-78
ENSDARG00000029172	polr1a	2.527408944	1.337659117	8.68E-81	5.90E-79
ENSDARG00000039400	polr1c	2.525947978	1.336824927	1.61E-62	6.83E-61
ENSDARG00000044002	cyp2x7	2.524903377	1.33622818	1.92E-58	7.32E-57
ENSDARG00000037946	prl	2.524882004	1.336215968	3.05E-31	4.41E-30
ENSDARG00000023002	dtl	2.523758855	1.335574068	1.10E-51	3.42E-50
ENSDARG00000021547	vrk2	2.523104036	1.335199695	8.33E-25	9.12E-24
ENSDARG00000035560	capga	2.521170125	1.334093473	0.004493847	0.010096764
ENSDARG00000020232	eif6	2.52014532	1.333506927	3.21E-80	2.15E-78
ENSDARG00000044400	wdr78	2.51945455	1.333111431	3.91E-10	1.81E-09
ENSDARG00000038205	her2	2.517255464	1.331851636	5.89E-50	1.74E-48
ENSDARG00000070546	msgn1	2.515570909	1.330885857	0.022091768	0.043870101
ENSDARG00000094614	si:dkeyp-69b9.2	2.515204575	1.330675747	3.67E-15	2.39E-14
ENSDARG00000074116		2.514950743	1.330530144	1.42E-08	5.82E-08
ENSDARG00000096282	notch1a	2.514559412	1.330305641	9.33E-25	1.02E-23
ENSDARG00000052331	BX322787.1	2.513488076	1.329690845	4.86E-44	1.19E-42
ENSDARG00000007277	myf5	2.512598648	1.32918024	5.71E-32	8.44E-31
ENSDARG00000097721	si:dkey-234i14.21	2.511814142	1.328729718	0.002007543	0.004753756

ENSDARG00000075714	C5H11orf88	2.511694259	1.32866086	7.33E-07	2.58E-06
ENSDARG00000091663		2.510943983	1.328229844	4.13E-11	2.05E-10
ENSDARG00000015599	xrcc5	2.509967074	1.327668439	1.97E-81	1.35E-79
ENSDARG00000056914	EMG1	2.508608805	1.326887513	2.18E-53	7.20E-52
ENSDARG00000037844	kdm8	2.50841135	1.326773953	3.27E-23	3.34E-22
ENSDARG00000020595	gnl3l	2.50812067	1.32660676	1.33E-78	8.55E-77
ENSDARG00000007955	iars	2.503891879	1.324172266	1.46E-40	3.07E-39
ENSDARG00000040569	tgm1l4	2.501394454	1.322732579	3.43E-29	4.52E-28
ENSDARG00000010347	acer1	2.50080017	1.322389781	7.42E-36	1.29E-34
ENSDARG00000016725	GADD45G (1 of 3)	2.498914914	1.32130178	3.88E-76	2.35E-74
ENSDARG00000040666	nifk	2.498244651	1.320914766	3.40E-80	2.28E-78
ENSDARG00000075700	ZSWIM2	2.49787724	1.320702577	0.000721909	0.001817115
ENSDARG00000097507	si:dkey-14d8.23	2.496057762	1.319651321	1.36E-09	6.03E-09
ENSDARG00000042835	tfdp2	2.49564016	1.319409931	6.13E-75	3.57E-73
ENSDARG00000006642	drg2	2.495369722	1.319253586	2.12E-79	1.40E-77
ENSDARG00000062632	duox	2.495252902	1.319186045	5.90E-68	2.87E-66
ENSDARG00000035984	mnx1	2.493960853	1.31843882	9.36E-25	1.02E-23
ENSDARG00000038000	crls1	2.493957615	1.318436947	1.07E-68	5.25E-67
ENSDARG00000052734	hmgcra	2.490789374	1.31660303	3.76E-42	8.42E-41
ENSDARG00000078054	SPDL1	2.490509954	1.316441177	5.97E-70	3.04E-68
ENSDARG00000091221	TTC34	2.487399908	1.314638473	5.33E-05	0.000155046
ENSDARG00000024631	NLRC5	2.486686279	1.314224508	0.000327308	0.000862051
ENSDARG00000063451	CCDC13	2.486316144	1.314009752	8.08E-07	2.83E-06
ENSDARG00000070560	nap1l4a	2.486093032	1.313880284	5.88E-62	2.44E-60
ENSDARG00000006394	mre11a	2.485446471	1.313505033	2.45E-47	6.64E-46
ENSDARG00000087529	ANKLE1	2.48525364	1.313393098	4.60E-80	3.06E-78
ENSDARG00000097848	si:ch73-111k20.3	2.48083989	1.310828628	3.06E-64	1.34E-62
ENSDARG00000077403	col8a1a	2.480661482	1.310724874	4.52E-78	2.85E-76
ENSDARG00000086621	CT583625.1	2.480349384	1.310543354	3.69E-05	0.000109173
ENSDARG00000073978	crabp2a	2.480245993	1.310483215	2.96E-46	7.71E-45

ENSDARG00000037182	SURF2	2.476836074	1.308498389	1.65E-53	5.46E-52
ENSDARG00000054154	bms1l	2.476585381	1.308352359	1.45E-70	7.52E-69
ENSDARG00000003961	parp3	2.475217023	1.307555024	1.37E-67	6.60E-66
ENSDARG00000055723	hsp70l	2.472351453	1.305883842	5.90E-18	4.52E-17
ENSDARG00000010437	fam46c	2.46920574	1.30404705	3.03E-70	1.56E-68
ENSDARG00000088930	Metazoa_SRP	2.467860976	1.303261124	0.014155828	0.029232469
ENSDARG00000040245	kpnb3	2.467728127	1.30318346	1.68E-24	1.81E-23
ENSDARG00000039069	slx4ip	2.467550225	1.30307945	5.53E-27	6.65E-26
ENSDARG00000007077	si:dkey-145p14.5	2.466822354	1.302653825	2.79E-75	1.63E-73
ENSDARG00000052471	taf1a	2.466679314	1.302570167	1.80E-45	4.60E-44
ENSDARG00000070461	C17H15orf41	2.46595687	1.302147567	2.70E-21	2.49E-20
ENSDARG00000032505	LYAR	2.465188988	1.301698251	1.54E-71	8.19E-70
ENSDARG00000012044	polr3gla	2.463394198	1.30064751	8.70E-34	1.39E-32
ENSDARG00000093977	si:ch211-220f16.1	2.461957743	1.299805999	0.023995386	0.04727695
ENSDARG00000016818	abcg2d	2.460068835	1.298698684	2.87E-31	4.15E-30
ENSDARG00000042990	cyp2p10	2.459528659	1.298381866	1.75E-20	1.55E-19
ENSDARG00000074446	zgc:173714	2.459451022	1.298336325	0.000995762	0.002458727
ENSDARG00000021924	hsp70.3	2.459224099	1.298203208	4.03E-63	1.72E-61
ENSDARG00000074691	mms22l	2.45910305	1.298132193	7.31E-64	3.17E-62
ENSDARG00000016015	ddx54	2.458929922	1.29803062	1.20E-77	7.49E-76
ENSDARG00000090386	cd3eap	2.458134613	1.297563923	5.31E-40	1.09E-38
ENSDARG00000015076	cx44.1	2.456764267	1.296759434	2.82E-45	7.18E-44
ENSDARG00000005846		2.45672722	1.296737678	0.004214538	0.009503029
ENSDARG00000079946	sqlea	2.456410595	1.296551731	3.96E-35	6.68E-34
ENSDARG00000074796	dbf4	2.454889419	1.29565804	2.28E-54	7.80E-53
ENSDARG00000033945	NOP14	2.454528689	1.29544603	6.85E-74	3.85E-72
ENSDARG00000095715	si:ch211-202e12.3	2.454206069	1.295256391	1.61E-69	8.05E-68
ENSDARG00000055868	RSL1D1	2.453500741	1.294841707	2.66E-77	1.64E-75
ENSDARG00000053318	lrrc6	2.453190348	1.29465918	1.41E-11	7.25E-11
ENSDARG00000083325	snoU85	2.452654284	1.294343892	0.008148492	0.0175383

ENSDARG00000058631	CABZ01030107.1	2.450208185	1.292904335	8.32E-11	4.04E-10
ENSDARG00000059857	blvra	2.450196273	1.292897321	4.57E-19	3.75E-18
ENSDARG00000063285	ube2t	2.449707624	1.292609572	1.41E-38	2.74E-37
ENSDARG00000079257	gemin5	2.449200107	1.292310651	1.45E-69	7.30E-68
ENSDARG00000058480	chtf18	2.448953341	1.292165287	1.26E-67	6.10E-66
ENSDARG00000097890	si:ch1073-100f3.2	2.448831416	1.292093458	3.70E-24	3.92E-23
ENSDARG00000057863	dnmt5	2.445420513	1.290082571	4.01E-52	1.27E-50
ENSDARG00000088321		2.445248866	1.289981303	5.81E-05	0.000168234
ENSDARG00000058030	hspa14	2.441935821	1.288025284	4.71E-77	2.90E-75
ENSDARG00000019726	celsr2	2.441072391	1.28751508	6.44E-76	3.87E-74
ENSDARG00000078462	CABZ01043311.1	2.439867777	1.286802966	2.55E-71	1.35E-69
ENSDARG00000044405	ttc4	2.43930986	1.286473032	2.12E-63	9.11E-62
ENSDARG00000042874	phlda2	2.439125056	1.286363728	1.26E-66	5.94E-65
ENSDARG00000079957	CABZ01066734.1	2.437318751	1.285294938	0.00812939	0.017498512
ENSDARG00000062924	CCDC176 (1 of 2)	2.435592132	1.284272557	0.002053022	0.004852147
ENSDARG00000090651	RFC4 (2 of 2)	2.434381226	1.283555113	1.55E-37	2.87E-36
ENSDARG00000003941	rrs1	2.432229391	1.2822793	4.76E-73	2.65E-71
ENSDARG00000061865	ddx10	2.432194604	1.282258666	1.18E-75	6.98E-74
ENSDARG00000079605	prmt5	2.430464489	1.281232056	7.38E-71	3.87E-69
ENSDARG00000026039	cyp1a	2.429638341	1.28074158	1.43E-49	4.18E-48
ENSDARG00000016753	ipo9	2.42938461	1.28059091	2.64E-70	1.37E-68
ENSDARG00000097742	si:ch73-204p21.5	2.428916546	1.280312922	0.020868557	0.041656479
ENSDARG00000097609	si:ch211-233f10.7	2.428177921	1.279874137	8.89E-07	3.10E-06
ENSDARG00000058325	casp8	2.427506928	1.279475413	4.98E-49	1.42E-47
ENSDARG00000021242	mvp	2.426234729	1.278719132	1.54E-69	7.76E-68
ENSDARG00000061509	BX927282.1	2.424276346	1.277554162	2.07E-09	9.02E-09
ENSDARG00000016485	CCDC39	2.422687635	1.276608405	8.63E-17	6.22E-16
ENSDARG00000082242	SNORA73	2.421235477	1.275743396	1.00E-06	3.48E-06
ENSDARG00000009280	smyd1a	2.420947804	1.275571975	1.29E-33	2.05E-32
ENSDARG00000076526	gar1	2.420738063	1.27544698	1.57E-69	7.84E-68

ENSDARG00000067838	C20H14orf169	2.420321976	1.275198983	6.97E-72	3.74E-70
ENSDARG00000003527	mak16	2.419288526	1.274582837	1.72E-74	9.92E-73
ENSDARG00000090379	CEP112	2.418364169	1.274031509	0.00024413	0.000652613
ENSDARG00000074656	ctssb.1	2.417237111	1.273358997	1.34E-33	2.12E-32
ENSDARG00000020494	znf330	2.416320029	1.272811545	6.68E-73	3.70E-71
ENSDARG00000074487	CR762483.1	2.416048697	1.272649534	9.68E-14	5.74E-13
ENSDARG00000091219	CABZ01078767.1	2.415877389	1.272547237	0.000802197	0.002004862
ENSDARG00000040925	wnt10b	2.415700643	1.272441685	9.46E-17	6.82E-16
ENSDARG00000016304	tfdp1b	2.415064582	1.272061769	1.55E-72	8.49E-71
ENSDARG00000091214	si:dkey-122c11.4	2.414611167	1.271790885	4.78E-06	1.55E-05
ENSDARG00000045683	ccdc87	2.414455302	1.271697756	5.42E-05	0.000157551
ENSDARG00000070657	pa2g4b	2.413618418	1.271197611	1.96E-36	3.48E-35
ENSDARG00000060298	nin	2.412379661	1.270456977	1.32E-74	7.64E-73
ENSDARG00000038386	ascl1a	2.411508972	1.269936178	1.15E-73	6.46E-72
ENSDARG00000038826	jph1b	2.411305846	1.269814652	5.48E-43	1.29E-41
ENSDARG00000021232	nkx2.7	2.410993515	1.26962777	1.57E-13	9.18E-13
ENSDARG00000031976	nos2b	2.410932919	1.26959151	2.38E-26	2.79E-25
ENSDARG00000007216	abce1	2.410457062	1.269306731	1.74E-36	3.10E-35
ENSDARG00000089405	zgc:112234	2.40989706	1.268971522	4.74E-09	2.01E-08
ENSDARG00000079122	tmx2a	2.408729063	1.268272126	1.27E-34	2.09E-33
ENSDARG00000092652		2.408504134	1.2681374	1.94E-05	5.91E-05
ENSDARG00000044512	ca4c	2.408262767	1.267992814	4.65E-05	0.000136352
ENSDARG00000030176	ITGB1BP2	2.406911056	1.26718283	2.51E-70	1.30E-68
ENSDARG00000054196	CR352249.1	2.406513827	1.266944713	3.53E-06	1.16E-05
ENSDARG00000095377	si:ch211-28e16.4	2.405046451	1.266064759	0.000246346	0.000657965
ENSDARG00000022399	ntmt1	2.402880375	1.264764827	3.23E-74	1.83E-72
ENSDARG00000087055	BX842614.1	2.401837607	1.264138611	5.83E-70	2.97E-68
ENSDARG00000036894	AIMP1 (1 of 2)	2.401487817	1.26392849	1.26E-70	6.61E-69
ENSDARG00000087418	VPRBP (2 of 2)	2.400340916	1.263239324	5.18E-54	1.74E-52
ENSDARG00000062147	otc	2.399237282	1.262575846	2.31E-32	3.48E-31

ENSDARG00000045552	si:dkey-202b22.6	2.398992937	1.26242891	0.006188091	0.013616964
ENSDARG00000094154	hist2h3c	2.398986862	1.262425257	9.46E-13	5.24E-12
ENSDARG00000090908	CABZ01099881.1	2.397669725	1.261632944	0.01018435	0.021575301
ENSDARG00000090371	BX664721.5	2.397482452	1.261520256	4.52E-44	1.11E-42
ENSDARG00000086662	MYO18B (1 of 4)	2.39555459	1.26035969	3.30E-45	8.36E-44
ENSDARG00000097189	si:ch1073-235i16.2	2.395329123	1.260223899	6.11E-17	4.45E-16
ENSDARG00000079907	ptger4c	2.394176343	1.259529418	0.002576456	0.005995974
ENSDARG00000039578	pa2g4a	2.391587018	1.257968285	5.84E-31	8.30E-30
ENSDARG00000088856	CABZ01078654.1	2.391244696	1.257761769	0.010174635	0.021561149
ENSDARG00000055554	wnt1	2.390223639	1.257145609	8.49E-21	7.66E-20
ENSDARG00000094210	zgc:109934	2.389879354	1.25693779	2.33E-15	1.53E-14
ENSDARG00000079031		2.387791397	1.255676805	6.62E-23	6.64E-22
ENSDARG00000020077	nol10	2.386527673	1.254913065	8.01E-73	4.41E-71
ENSDARG00000078068	abcg4b	2.385689745	1.254406435	2.91E-11	1.46E-10
ENSDARG00000089426	nop10	2.385602104	1.254353435	5.16E-64	2.25E-62
ENSDARG00000069292	si:dkeyp-89c11.1	2.382435783	1.252437328	4.43E-32	6.59E-31
ENSDARG00000046133	b3galnt2	2.381614261	1.251939765	2.63E-34	4.26E-33
ENSDARG00000089194	TACO1 (2 of 2)	2.380270288	1.251125406	7.19E-37	1.30E-35
ENSDARG00000003217	urb2	2.37916026	1.250452455	7.69E-70	3.90E-68
ENSDARG00000005419	il1b	2.377895851	1.249685528	3.72E-05	0.000109978
ENSDARG00000043877	mmachc	2.377650669	1.249536766	3.64E-13	2.08E-12
ENSDARG00000004774	wdr36	2.377365358	1.249363637	6.98E-73	3.85E-71
ENSDARG00000035852		2.375740363	1.248377177	1.45E-10	6.92E-10
ENSDARG00000023062	cyr61	2.375546206	1.248259268	1.50E-72	8.21E-71
ENSDARG00000095082	BX571811.2	2.375175857	1.248034334	0.022620257	0.04482872
ENSDARG00000029179	foxd1	2.373915182	1.24726839	1.34E-70	6.97E-69
ENSDARG00000056720	utp3	2.372034453	1.246124964	1.90E-66	8.94E-65
ENSDARG00000059908	CCDC86	2.371011759	1.245502818	2.95E-68	1.44E-66
ENSDARG00000097523	si:ch73-364h19.1	2.369390682	1.2445161	6.74E-11	3.30E-10
ENSDARG00000093412	sertad2a	2.369254599	1.244433238	3.92E-33	6.10E-32

ENSDARG00000042520	UTP14A	2.36883461	1.244177474	3.55E-72	1.91E-70
ENSDARG00000041321	wdr16	2.368562711	1.24401187	7.50E-13	4.19E-12
ENSDARG00000027744	gadd45ba	2.368155237	1.243763655	1.52E-71	8.11E-70
ENSDARG00000057973	dph1	2.367846831	1.24357576	9.68E-42	2.13E-40
ENSDARG00000036292	cdx4	2.367605874	1.243428941	0.00368158	0.008377762
ENSDARG00000017696	diexf	2.366642095	1.242841545	4.21E-71	2.21E-69
ENSDARG00000059711	nol6	2.364832169	1.2417378	2.35E-72	1.27E-70
ENSDARG00000016161	ccdc170	2.364703983	1.241659596	1.03E-06	3.58E-06
ENSDARG00000045669	si:dkey-39a18.1	2.363796112	1.241105602	3.24E-07	1.18E-06
ENSDARG00000007709	adamts8a	2.361946161	1.23997608	1.35E-25	1.53E-24
ENSDARG00000054150	cx23	2.361681416	1.239814363	3.49E-48	9.67E-47
ENSDARG00000094627	DYNLT1 (2 of 2)	2.361312591	1.239589038	0.000797478	0.001994257
ENSDARG00000030494	hfe2	2.360368753	1.239012265	5.42E-42	1.21E-40
ENSDARG00000077310	gpr144	2.359940712	1.238750616	2.58E-59	1.01E-57
ENSDARG00000029187	exosc3	2.359896736	1.238723732	3.05E-61	1.24E-59
ENSDARG00000008480	trmt61a	2.359324287	1.238373729	5.26E-52	1.66E-50
ENSDARG00000042737	slc52a3	2.356986361	1.23694341	1.40E-09	6.20E-09
ENSDARG00000062042	mthfsd	2.355501011	1.236033951	9.29E-07	3.23E-06
ENSDARG00000063341	C2H1orf123	2.355481538	1.236022025	1.13E-65	5.17E-64
ENSDARG00000043336	exosc7	2.355036134	1.235749196	5.70E-50	1.68E-48
ENSDARG00000063333	nup210	2.354440709	1.235384392	1.99E-54	6.83E-53
ENSDARG00000067639	prpf4	2.353909222	1.235058684	3.12E-55	1.10E-53
ENSDARG00000018605	tekt1	2.353884742	1.23504368	0.002059698	0.004865497
ENSDARG00000030267	gtf3aa	2.353239661	1.234648256	5.62E-43	1.32E-41
ENSDARG00000093108	si:ch211-69l10.2	2.352217249	1.234021313	3.55E-12	1.89E-11
ENSDARG00000056007	cyb5d1	2.351589907	1.233636491	9.07E-05	0.000257387
ENSDARG00000058968	haus1	2.350161953	1.232760178	2.55E-34	4.15E-33
ENSDARG00000094426	her4.2	2.349740574	1.232501483	3.60E-65	1.62E-63
ENSDARG00000090484	dnali1	2.349660798	1.232452501	4.39E-06	1.43E-05
ENSDARG00000092586	si:dkey-238o14.6	2.34945322	1.232325042	0.010383655	0.021959865

ENSDARG00000026395	rfx4	2.348233692	1.23157599	1.19E-67	5.75E-66
ENSDARG00000062978	ccdc151	2.347293178	1.230998046	1.40E-14	8.78E-14
ENSDARG00000090377	CABZ01003344.1	2.346266202	1.230366707	0.001143888	0.002797977
ENSDARG00000016343	zgc:92518	2.345864737	1.23011983	1.97E-50	5.90E-49
ENSDARG00000092362	hsp70.2	2.345491132	1.229890046	3.44E-38	6.54E-37
ENSDARG00000062898	cep128	2.345455463	1.229868106	3.74E-25	4.15E-24
ENSDARG00000055253	slc12a10.3	2.345060411	1.229625088	1.37E-05	4.25E-05
ENSDARG00000014385	chek1	2.34504522	1.229615743	5.41E-49	1.54E-47
ENSDARG00000058782	si:ch73-242m19.1	2.344785995	1.229456256	2.57E-07	9.47E-07
ENSDARG00000007278	ect2	2.342081971	1.22779157	2.74E-70	1.41E-68
ENSDARG00000075517	MPV17L	2.341274773	1.227294259	2.86E-08	1.14E-07
ENSDARG00000090403	cyp2aa8	2.34096274	1.227101972	3.36E-70	1.72E-68
ENSDARG00000096489	si:dkey-227h16.2	2.340713607	1.226948427	3.05E-54	1.03E-52
ENSDARG00000011262	traip	2.340178204	1.226618395	1.14E-39	2.30E-38
ENSDARG00000088319	CABZ01038329.1	2.339728522	1.226341144	1.38E-09	6.15E-09
ENSDARG00000004937	skp2	2.339114055	1.225962209	8.13E-60	3.21E-58
ENSDARG00000077607	dus2	2.338383845	1.225511767	3.74E-50	1.11E-48
ENSDARG00000096020	si:ch211-120c15.3	2.338139834	1.225361214	9.50E-07	3.30E-06
ENSDARG00000094484	si:dkeyp-4f2.5	2.337977838	1.225261255	0.003738566	0.00849927
ENSDARG00000071083	si:dkeyp-34c12.1	2.336432409	1.224307302	0.005055152	0.011265257
ENSDARG00000088845	CABZ01085177.1	2.335370186	1.223651253	0.009543927	0.020317045
ENSDARG00000036811	ifrd2	2.335310731	1.223614524	5.14E-63	2.19E-61
ENSDARG00000059294	marco	2.334545802	1.223141893	2.96E-20	2.59E-19
ENSDARG00000056324	zgc:123295	2.33384619	1.222709485	4.71E-09	2.00E-08
ENSDARG00000051855	gart	2.333654465	1.222590963	3.52E-53	1.16E-51
ENSDARG00000075538	zgc:163061	2.333559999	1.222532562	5.26E-05	0.000153032
ENSDARG00000026767	nol11	2.33353613	1.222517805	1.17E-69	5.92E-68
ENSDARG00000039887	c1qbp	2.333451658	1.22246558	4.23E-58	1.59E-56
ENSDARG00000023532	pinx1	2.333379241	1.222420806	2.57E-61	1.05E-59
ENSDARG00000032465	slc1a8b	2.33277767	1.222048815	0.018143358	0.036654382

ENSDARG00000058719	SMC6	2.33234861	1.221783441	1.21E-37	2.24E-36
ENSDARG00000006219	gnl3	2.331258242	1.221108826	3.29E-64	1.44E-62
ENSDARG00000055683	si:ch211-198e20.11	2.329606569	1.220086329	0.000159211	0.000437111
ENSDARG00000053535	lmo7b	2.328589744	1.219456485	1.55E-36	2.77E-35
ENSDARG00000087917	BX649341.1	2.327742469	1.218931454	0.012768071	0.026602567
ENSDARG00000090210		2.327245844	1.218623621	0.000452909	0.001171036
ENSDARG00000069945	p2ry12	2.326167186	1.21795479	0.000859853	0.002141005
ENSDARG00000004727	cmss1	2.324899675	1.217168462	2.24E-39	4.51E-38
ENSDARG00000094097	si:ch211-209a2.1	2.324162932	1.216711211	3.68E-38	6.97E-37
ENSDARG00000087923		2.32410411	1.216674697	9.83E-15	6.22E-14
ENSDARG00000042659	thyn1	2.323640751	1.216387037	1.40E-25	1.58E-24
ENSDARG00000097072	si:ch211-229g11.6	2.323553952	1.216333144	1.37E-34	2.25E-33
ENSDARG00000020289	pif1	2.323263431	1.216152748	2.31E-48	6.46E-47
ENSDARG00000032885	C11H1orf158	2.322878665	1.215913797	0.0001133	0.000317162
ENSDARG00000020527	nup62l	2.322825262	1.215880629	2.03E-63	8.75E-62
ENSDARG00000037617	ASCC1	2.322476623	1.215664075	4.77E-06	1.55E-05
ENSDARG00000086426	Metazoa_SRP	2.321279389	1.214920176	0.011223717	0.023616935
ENSDARG00000053912	fbl	2.320351578	1.214343418	1.25E-43	3.00E-42
ENSDARG00000058053	serping1	2.319455017	1.213785867	8.01E-35	1.33E-33
ENSDARG00000069630	tat	2.31912034	1.213577684	1.28E-19	1.08E-18
ENSDARG00000070770	her4.3	2.318911073	1.213447496	1.37E-46	3.61E-45
ENSDARG00000044220	kars	2.318464253	1.213169483	7.95E-26	9.09E-25
ENSDARG00000021462	daw1	2.318115568	1.212952492	9.48E-21	8.52E-20
ENSDARG00000069435	ccdc147	2.316644382	1.212036599	9.02E-11	4.37E-10
ENSDARG00000087144	FAM183B	2.316320297	1.211834761	0.000190266	0.000516138
ENSDARG00000070061	gfer	2.315414782	1.21127066	8.63E-45	2.17E-43
ENSDARG00000033376	chchd4	2.315374244	1.211245402	4.00E-59	1.56E-57
ENSDARG00000003287	wdr12	2.314171779	1.210495958	2.50E-62	1.05E-60
ENSDARG00000087373	dnah5	2.314008077	1.2103939	1.49E-23	1.54E-22
ENSDARG00000067859	scospondin	2.312896875	1.209700942	5.95E-26	6.86E-25

ENSDARG00000077469	polr1b	2.312136491	1.209226566	7.56E-59	2.92E-57
ENSDARG00000043976	etf1	2.308409213	1.206898994	2.10E-26	2.47E-25
ENSDARG00000070598	eno4	2.307272399	1.20618834	2.50E-11	1.26E-10
ENSDARG00000054980	ebna1bp2	2.306098808	1.205454329	9.09E-68	4.42E-66
ENSDARG00000086922	zgc:173702	2.305626387	1.205158752	0.007564821	0.016372582
ENSDARG00000053136	b2m	2.305384053	1.205007109	7.44E-13	4.15E-12
ENSDARG00000073899	tmem194a	2.304688505	1.204571773	2.03E-38	3.91E-37
ENSDARG00000076518	mettl1	2.304676811	1.204564453	3.83E-45	9.70E-44
ENSDARG00000021339	cpa5	2.304399812	1.204391045	8.30E-17	5.99E-16
ENSDARG00000054530	rars	2.30266121	1.203302164	4.77E-66	2.21E-64
ENSDARG00000042525	coe2	2.302070822	1.202932218	3.69E-66	1.72E-64
ENSDARG00000077265	WDR89	2.301845024	1.202790705	7.61E-31	1.08E-29
ENSDARG00000087013	cubn	2.301134662	1.202345412	3.62E-43	8.57E-42
ENSDARG00000091506	CABZ01073071.2	2.300165488	1.201737662	4.06E-11	2.02E-10
ENSDARG00000069841	CU596012.2	2.299737287	1.201469063	7.39E-22	6.99E-21
ENSDARG00000058479	zgc:110425	2.298639529	1.200780242	0.001061071	0.002610913
ENSDARG00000020169	fahd2a	2.298027112	1.200395819	1.26E-63	5.43E-62
ENSDARG00000031047	ptrh1	2.297696532	1.200188267	1.07E-34	1.76E-33
ENSDARG00000087832	bcl3	2.295167463	1.198599421	1.03E-08	4.27E-08
ENSDARG00000091947	znf106b	2.295125905	1.198573299	6.53E-19	5.30E-18
ENSDARG00000095529	gusb	2.293273809	1.197408618	2.16E-08	8.69E-08
ENSDARG00000076395	nme8	2.292616848	1.196995266	7.59E-09	3.17E-08
ENSDARG00000075169	bbs1	2.292233531	1.196754032	4.20E-37	7.64E-36
ENSDARG00000021566	OTOP1	2.291894899	1.196540887	3.91E-33	6.07E-32
ENSDARG00000040070	coil	2.291887685	1.196536346	1.40E-36	2.51E-35
ENSDARG00000002720	utp15	2.291216016	1.196113483	4.40E-61	1.79E-59
ENSDARG00000011404	fen1	2.288438726	1.194363663	7.91E-67	3.76E-65
ENSDARG00000017983	rtdr1	2.288114899	1.1941595	5.17E-05	0.00015085
ENSDARG00000076896	C2H10orf67	2.287935825	1.194046586	0.013770637	0.028517864
ENSDARG00000042458	rfc4	2.287741239	1.193923881	1.59E-55	5.63E-54

ENSDARG00000026454	MIS12	2.285810832	1.192706015	6.02E-43	1.41E-41
ENSDARG00000032263	zgc:110224	2.284528551	1.191896473	6.08E-19	4.95E-18
ENSDARG00000097163	si:dkey-182i1.1	2.283602736	1.191311696	9.51E-05	0.000269133
ENSDARG00000043640	cenpn	2.283576695	1.191295244	2.09E-38	4.00E-37
ENSDARG00000039412	dmrta2	2.28277061	1.190785894	3.93E-38	7.43E-37
ENSDARG00000057716	acrc	2.282228611	1.190443314	1.03E-41	2.26E-40
ENSDARG00000054128	im:7136021	2.281033582	1.189687686	6.51E-30	8.86E-29
ENSDARG00000033579	mtrf1	2.280248558	1.189191094	9.12E-15	5.78E-14
ENSDARG00000057911	ACTC1 (5 of 6)	2.277445855	1.187416755	1.03E-37	1.93E-36
ENSDARG00000087213	CABZ01053617.1	2.277334198	1.187346022	3.86E-05	0.000113969
ENSDARG00000013478	rrp15	2.275775302	1.18635812	1.55E-52	4.96E-51
ENSDARG00000089705	ABCF3	2.273058453	1.184634784	2.29E-54	7.84E-53
ENSDARG00000077407	C19H18orf21 (2 of 2)	2.272890913	1.184528444	1.62E-16	1.15E-15
ENSDARG00000097567	si:dkey-208c12.2	2.272446103	1.184246078	9.21E-05	0.000261073
ENSDARG00000040548	eed	2.271660675	1.183747351	3.43E-66	1.60E-64
ENSDARG00000044010	loxl2a	2.271425646	1.183598081	1.48E-58	5.68E-57
ENSDARG00000086021	si:dkey-20l4.2	2.270865633	1.183242344	1.14E-05	3.55E-05
ENSDARG00000012820	nop56	2.270484413	1.183000133	9.76E-35	1.61E-33
ENSDARG00000008022	kif18a	2.270408639	1.182951984	7.31E-55	2.54E-53
ENSDARG00000093019	si:dkey-83k24.5	2.269306339	1.182251375	7.35E-08	2.82E-07
ENSDARG00000079146	smarcal1	2.268431743	1.18169525	8.94E-43	2.08E-41
ENSDARG00000027907	CR318603.2	2.267244913	1.180940242	3.20E-06	1.05E-05
ENSDARG00000054259	nat10	2.266373377	1.18038556	5.83E-65	2.61E-63
ENSDARG00000007628	dusp1	2.26527199	1.179684284	2.45E-50	7.31E-49
ENSDARG00000079973	pus10	2.263282625	1.178416751	3.57E-42	8.01E-41
ENSDARG00000068021	si:ch211-224b1.4	2.262966533	1.178215249	1.30E-07	4.91E-07
ENSDARG00000062568	MRPL2	2.262847764	1.178139529	7.25E-60	2.86E-58
ENSDARG00000061515	tmtops2a	2.26227514	1.177774402	2.35E-27	2.87E-26
ENSDARG00000071500	fam207a	2.261350405	1.17718456	1.15E-25	1.30E-24
ENSDARG00000029859	C8H1orf228	2.260844135	1.176861535	4.36E-06	1.42E-05

ENSDARG00000078148	HEATR2 (1 of 2)	2.260533852	1.176663523	7.27E-15	4.64E-14
ENSDARG00000054414	spata17	2.260088275	1.176379123	0.002916213	0.006740287
ENSDARG00000045776	cnbpa	2.259570129	1.176048334	5.24E-37	9.50E-36
ENSDARG00000070216	FANCA	2.258767142	1.17553555	1.06E-37	1.98E-36
ENSDARG00000077704	FUT9 (13 of 16)	2.258723743	1.17550783	0.007046414	0.015344815
ENSDARG00000042172	c7a	2.256659269	1.174188604	9.77E-64	4.23E-62
ENSDARG00000097425	si:dkey-237p10.2	2.2556566	1.173547449	0.014639105	0.03015594
ENSDARG00000020028	cps1	2.255209529	1.173261479	3.97E-48	1.10E-46
ENSDARG00000025314	tcerg1a	2.254412501	1.172751516	9.46E-56	3.38E-54
ENSDARG00000075795	nol7	2.253997554	1.17248595	2.17E-62	9.13E-61
ENSDARG00000017355	rsph9	2.253719481	1.172307955	1.15E-08	4.75E-08
ENSDARG00000060992	rpusd2	2.253537768	1.172191629	1.99E-33	3.13E-32
ENSDARG00000091806	CABZ01072244.1	2.251783633	1.17106821	1.72E-57	6.39E-56
ENSDARG00000041295	lim2.5	2.250793821	1.170433908	7.82E-48	2.15E-46
ENSDARG00000016337	lctlb	2.250719023	1.170385964	5.48E-47	1.47E-45
ENSDARG00000088619	REXO4	2.250530048	1.170264827	7.46E-47	1.99E-45
ENSDARG00000052721	PRIM2	2.248316991	1.168845456	3.68E-59	1.43E-57
ENSDARG00000043209	nsun5	2.247634074	1.168407177	3.84E-32	5.72E-31
ENSDARG00000044754	gnrh2	2.246525732	1.167695587	0.000676749	0.00170935
ENSDARG00000020913	ddx56	2.246321177	1.167564217	9.22E-62	3.80E-60
ENSDARG00000045019	aamp	2.245371121	1.166953917	7.44E-61	3.00E-59
ENSDARG00000024681	pane1	2.244198578	1.166200339	3.11E-27	3.78E-26
ENSDARG00000018810	ZCCHC4	2.24403142	1.166092876	6.94E-21	6.28E-20
ENSDARG00000040890	fdps	2.243888928	1.166001264	4.34E-60	1.73E-58
ENSDARG00000011648	snrpd1	2.243390847	1.165680991	3.30E-58	1.25E-56
ENSDARG00000095564	CABZ01118769.1	2.243141175	1.165520421	0.00456302	0.010238419
ENSDARG00000092505	si:dkeyp-80d11.1	2.241810772	1.164664507	0.02277639	0.045116117
ENSDARG00000095738	BX649331.2	2.241804818	1.164660676	0.023917345	0.047149347
ENSDARG00000086337	si:dkey-102g19.3	2.241409201	1.164406058	0.016997485	0.034580377
ENSDARG00000027938	rad18	2.241385403	1.164390739	2.86E-40	5.94E-39

ENSDARG00000095801	si:dkey-119g10.4	2.239694258	1.163301803	1.16E-05	3.61E-05
ENSDARG00000042864	rpl7l1	2.239577507	1.163226596	3.07E-62	1.28E-60
ENSDARG00000097493	si:dkey-148h10.5	2.239267658	1.163026983	2.91E-07	1.07E-06
ENSDARG00000058389	ccl-c5a	2.236838407	1.161461038	0.004155759	0.009382405
ENSDARG00000093664	si:dkey-115d18.2	2.23583278	1.160812292	4.32E-08	1.69E-07
ENSDARG00000068374	si:ch211-132b12.7	2.235156842	1.160376069	9.84E-05	0.000277855
ENSDARG00000017314	CELA1 (1 of 7)	2.234709237	1.160087131	3.24E-09	1.39E-08
ENSDARG00000082894	snoR38	2.23456676	1.159995147	0.011033417	0.023250945
ENSDARG00000057770	plp1a	2.234021508	1.159643075	1.29E-60	5.16E-59
ENSDARG00000030262	si:ch211-220i18.4	2.233792225	1.159495	1.33E-14	8.35E-14
ENSDARG00000060115	lrrn1	2.233665258	1.159412997	2.57E-28	3.25E-27
ENSDARG00000062900	RNF115 (2 of 2)	2.233307177	1.159181698	2.05E-25	2.30E-24
ENSDARG00000077666	evi5a	2.232878099	1.158904491	0.001289917	0.003132245
ENSDARG00000037432	haus7	2.232699689	1.158789214	2.67E-42	6.04E-41
ENSDARG00000036152	GAS2 (2 of 2)	2.230521993	1.157381373	1.73E-08	7.03E-08
ENSDARG00000043518	si:dkey-239i20.2	2.230205486	1.157176643	0.000127869	0.000355633
ENSDARG00000014274	rfc2	2.230064183	1.157085233	4.67E-57	1.71E-55
ENSDARG00000093569	bckdhbl	2.229981868	1.15703198	4.91E-54	1.65E-52
ENSDARG00000016260	fxr2	2.229269518	1.156571048	2.66E-40	5.55E-39
ENSDARG00000051902	prmt7	2.228979508	1.156383354	2.88E-55	1.01E-53
ENSDARG00000023262	mapk15	2.228805502	1.156270725	0.002315947	0.005423026
ENSDARG00000021997	C20H6orf165	2.228725261	1.156218784	4.74E-07	1.70E-06
ENSDARG00000068755	exosc8	2.227576701	1.155475108	2.07E-42	4.71E-41
ENSDARG00000055739	ribc1	2.22653498	1.154800278	7.00E-08	2.69E-07
ENSDARG00000088160	CEP295	2.226414246	1.154722045	1.27E-18	1.01E-17
ENSDARG00000042725	cebpb	2.225863025	1.154364815	1.45E-58	5.56E-57
ENSDARG00000054224	IPO11	2.225126586	1.153887413	4.85E-61	1.97E-59
ENSDARG00000058212	ndnl2	2.225094353	1.153866513	2.51E-39	5.03E-38
ENSDARG00000054837	zgc:136870	2.224833018	1.153697061	0.000562628	0.001435262
ENSDARG00000029014	orc4	2.224327073	1.153368943	2.09E-50	6.22E-49

ENSDARG00000043157	dnajb13	2.224157263	1.1532588	0.000382383	0.000998773
ENSDARG00000026225	whsc1	2.22364157	1.152924258	2.09E-51	6.43E-50
ENSDARG00000095554	si:ch211-223a21.6	2.223532806	1.15285369	0.004825798	0.010789692
ENSDARG00000033726	tshb	2.223160522	1.152612121	0.000401808	0.001046433
ENSDARG00000037708	exosc10	2.223106313	1.152576942	3.78E-62	1.58E-60
ENSDARG00000022122	cdkal1	2.221744384	1.151692841	4.13E-55	1.45E-53
ENSDARG00000067517	CCDC15	2.221036018	1.151232789	7.02E-13	3.92E-12
ENSDARG00000055360	LLPH	2.218468165	1.149563851	7.56E-59	2.92E-57
ENSDARG00000051851	ctu2	2.218345397	1.149484011	9.91E-31	1.39E-29
ENSDARG00000090605	FILIP1L (2 of 2)	2.218050075	1.149291937	1.47E-09	6.51E-09
ENSDARG00000019765	C1H7orf55	2.216093766	1.148018925	9.34E-49	2.64E-47
ENSDARG00000016363	her8a	2.215755079	1.14779842	3.28E-61	1.33E-59
ENSDARG00000038847	gins3	2.215685749	1.147753278	1.06E-28	1.37E-27
ENSDARG00000040159	wnt4b	2.215331833	1.147522815	1.83E-40	3.81E-39
ENSDARG00000007971	cks1b	2.21484113	1.147203218	1.41E-37	2.61E-36
ENSDARG00000058419	gcn1l1	2.214094109	1.146716545	1.01E-23	1.05E-22
ENSDARG00000054446	ccdc43	2.21367394	1.146442738	1.74E-56	6.31E-55
ENSDARG00000077198	chst12b	2.213488296	1.146321745	0.021928214	0.043584914
ENSDARG00000026178	ube3d	2.212690983	1.145801983	1.22E-20	1.09E-19
ENSDARG00000005827	eif2s1a	2.211921847	1.145300412	1.00E-57	3.74E-56
ENSDARG00000068478	gpx4a	2.211904593	1.145289158	7.89E-38	1.48E-36
ENSDARG00000042182	drc1	2.210137733	1.144136279	1.80E-14	1.12E-13
ENSDARG00000087318	zgc:174688	2.209926628	1.143998471	7.81E-23	7.80E-22
ENSDARG00000042285	atp8b3	2.208913803	1.143337123	8.47E-62	3.49E-60
ENSDARG00000092204	ms4a17a.5	2.208077304	1.142790681	2.12E-06	7.11E-06
ENSDARG00000077461	dhx32a	2.207998667	1.142739301	2.17E-25	2.43E-24
ENSDARG00000061763	spata5l1	2.20796189	1.142715271	8.68E-31	1.22E-29
ENSDARG00000069916	SGOL1	2.205983694	1.141422127	5.67E-61	2.30E-59
ENSDARG00000032516	rrp36	2.205807979	1.141307206	2.23E-42	5.05E-41
ENSDARG00000074085	si:ch1073-188e1.1	2.20546046	1.141079896	8.49E-14	5.06E-13

ENSDARG00000010572	slc25a25a	2.205291035	1.140969063	9.32E-62	3.83E-60
ENSDARG00000036136	tipin	2.205277378	1.140960128	7.20E-30	9.77E-29
ENSDARG00000044125	txn	2.204832099	1.140668797	2.57E-59	1.01E-57
ENSDARG00000033134	ndufaf7	2.204557674	1.14048922	2.36E-42	5.34E-41
ENSDARG00000070311	rhbdl3	2.203972406	1.140106161	1.98E-33	3.12E-32
ENSDARG00000037046	rad51b	2.201605362	1.138555889	1.97E-08	7.97E-08
ENSDARG00000091063	FANCM (3 of 3)	2.200866521	1.138071651	0.001751075	0.004176037
ENSDARG00000068175	ing5b	2.200169021	1.137614358	7.35E-27	8.80E-26
ENSDARG00000042344	trmt6	2.200099181	1.137568562	4.24E-32	6.32E-31
ENSDARG00000071288	samhd1	2.199891763	1.137432543	2.08E-42	4.73E-41
ENSDARG00000005738	rpf1	2.19847034	1.136500069	1.74E-53	5.76E-52
ENSDARG00000071684	rx1	2.198276855	1.136373094	5.82E-54	1.95E-52
ENSDARG00000035458	atp2a1l	2.19793311	1.136147481	1.57E-12	8.58E-12
ENSDARG00000067548	fgf10b	2.197371356	1.135778706	0.024294432	0.047773389
ENSDARG00000056650	zgc:113276	2.196485617	1.135197052	1.11E-24	1.20E-23
ENSDARG00000074565	PUSL1	2.195794731	1.134743193	6.24E-08	2.40E-07
ENSDARG00000038583	abraa	2.193534695	1.133257526	1.82E-57	6.76E-56
ENSDARG00000032005	ccdc65	2.192987873	1.132897834	8.54E-11	4.14E-10
ENSDARG00000033757	ncaph2	2.192943416	1.132868587	2.03E-55	7.18E-54
ENSDARG00000015512	aqp8b	2.192688563	1.132700914	1.84E-10	8.69E-10
ENSDARG00000002830	trmt2a	2.192122739	1.132328579	2.01E-41	4.36E-40
ENSDARG00000001777	nup155	2.191953443	1.132217156	8.18E-53	2.65E-51
ENSDARG00000035730	utp6	2.191714158	1.132059655	2.98E-58	1.13E-56
ENSDARG00000003216	anxa2a	2.190717363	1.131403366	2.85E-38	5.43E-37
ENSDARG00000091946	si:ch211-139k8.2	2.19039354	1.131190097	1.32E-08	5.41E-08
ENSDARG00000056464	fitm1	2.190238027	1.131087665	1.17E-55	4.17E-54
ENSDARG00000025820	chek2	2.189823781	1.130814778	3.48E-22	3.35E-21
ENSDARG00000093389	CCHCR1 (2 of 2)	2.187930497	1.129566909	1.98E-09	8.67E-09
ENSDARG00000029747	mep1a.1	2.184447535	1.127268456	0.012322588	0.02575371
ENSDARG00000040584	n6amt2	2.184323248	1.12718637	5.03E-38	9.48E-37

ENSDARG00000022615	LAMA3 (1 of 2)	2.182484431	1.125971362	1.05E-49	3.07E-48
ENSDARG00000029406	h2afx	2.181748624	1.125484887	3.11E-49	8.95E-48
ENSDARG00000040036	tcf19l	2.180589206	1.124718011	2.48E-27	3.03E-26
ENSDARG00000002917	GLS2 (1 of 2)	2.18028547	1.124517043	0.00049871	0.001282224
ENSDARG00000089320	ccdc175	2.180186709	1.124451692	0.000322896	0.000851299
ENSDARG00000019874	hsph1	2.17984223	1.124223721	2.29E-15	1.51E-14
ENSDARG00000061377	efcab1	2.179090229	1.123725935	2.04E-06	6.85E-06
ENSDARG00000059679	zgc:158343	2.178605749	1.123405143	2.30E-10	1.08E-09
ENSDARG00000011133	ccne1	2.177877307	1.122922681	4.04E-30	5.55E-29
ENSDARG00000039934	hlcs	2.177664187	1.122781496	2.18E-51	6.71E-50
ENSDARG00000087466	DNAH9	2.176994918	1.12233804	5.88E-14	3.54E-13
ENSDARG00000079155	si:ch211-67f13.8	2.176591731	1.122070823	0.01913784	0.038474737
ENSDARG00000094376	NLRP6 (125 of 145)	2.176571194	1.12205721	0.019092784	0.03839801
ENSDARG00000039345	drg1	2.175885832	1.121602861	1.19E-58	4.57E-57
ENSDARG00000069681	pcgf6	2.174023098	1.120367269	9.50E-28	1.18E-26
ENSDARG00000012899	ddx49	2.172972948	1.119670214	3.05E-42	6.88E-41
ENSDARG00000063596	wbscr22	2.171908365	1.118963236	2.96E-41	6.38E-40
ENSDARG00000027658	irf10	2.170858377	1.11826561	0.001953187	0.004632765
ENSDARG00000002304	gins2	2.169481639	1.117350376	1.23E-23	1.28E-22
ENSDARG00000077179	ccdc83	2.168685345	1.116820747	0.002621761	0.00609442
ENSDARG00000088717	C6H2orf40 (2 of 2)	2.168370114	1.116611028	1.08E-24	1.18E-23
ENSDARG00000000349	C21H11orf70	2.168369789	1.116610812	0.000178188	0.000485228
ENSDARG00000038788	DNAI1 (1 of 2)	2.167847254	1.116263108	1.74E-13	1.01E-12
ENSDARG00000088231	CELSR2 (2 of 2)	2.166160407	1.115140081	3.34E-59	1.30E-57
ENSDARG00000018958	nrarpb	2.166041652	1.115060985	6.10E-59	2.36E-57
ENSDARG00000077923	zgc:174311	2.165048596	1.114399408	0.004472301	0.010050739
ENSDARG00000018061	neil1	2.165047503	1.11439868	9.59E-24	9.98E-23
ENSDARG00000073866	tdp1	2.164934543	1.114323405	7.96E-46	2.06E-44
ENSDARG00000039232	DUSP8 (2 of 2)	2.164785116	1.114223825	8.89E-39	1.74E-37
ENSDARG00000063626	ddx21	2.164764392	1.114210014	1.25E-37	2.32E-36

ENSDARG00000040178	havcr1	2.163605813	1.113437679	9.31E-18	7.07E-17
ENSDARG00000044143	nol8	2.162801556	1.1129013	1.06E-48	3.00E-47
ENSDARG00000092854	BX548173.1	2.160872772	1.111614132	0.004071921	0.009204829
ENSDARG00000021938	smad9	2.16070382	1.111501327	4.80E-45	1.21E-43
ENSDARG00000035221	zgc:110249	2.160583975	1.111421305	2.29E-06	7.66E-06
ENSDARG00000096986	si:dkey-14d8.22	2.159382911	1.110619091	0.000618329	0.001569464
ENSDARG00000037190	TRIM47 (16 of 86)	2.15850882	1.110034988	1.07E-19	9.10E-19
ENSDARG00000004736	pacrg	2.157361143	1.109267704	0.000226654	0.000608574
ENSDARG00000060507	depdc1b	2.157236612	1.109184424	8.66E-40	1.77E-38
ENSDARG00000073711	mmrn2b	2.15692154	1.108973698	2.39E-54	8.14E-53
ENSDARG00000053439	sec24a	2.156716999	1.108836881	5.64E-33	8.71E-32
ENSDARG00000040301	lrrc20	2.156198873	1.108490248	5.52E-58	2.07E-56
ENSDARG00000010316	qars	2.156145468	1.108454515	2.01E-49	5.82E-48
ENSDARG00000075737	A2ML1 (6 of 12)	2.156021689	1.108371691	0.008409938	0.018057249
ENSDARG00000053569	sox3	2.153304362	1.106552254	2.91E-47	7.85E-46
ENSDARG00000069823	proca1	2.153233135	1.106504532	7.97E-31	1.13E-29
ENSDARG00000060316	cish	2.152007053	1.105682807	2.58E-23	2.65E-22
ENSDARG00000076521	MMP21	2.150876695	1.10492482	0.019247733	0.038684717
ENSDARG00000091402	eif2b1	2.150493036	1.10466746	3.07E-42	6.91E-41
ENSDARG00000042727	EXO5	2.14545093	1.101280904	2.41E-24	2.58E-23
ENSDARG00000095879	wdr46	2.144546627	1.100672683	8.53E-32	1.26E-30
ENSDARG00000057529	itpa	2.143967709	1.100283177	2.64E-25	2.94E-24
ENSDARG00000034060	elac2	2.143838049	1.100195925	1.46E-51	4.54E-50
ENSDARG00000016212	nup214	2.14363644	1.100060246	1.65E-50	4.95E-49
ENSDARG00000010770	sox19a	2.143623068	1.100051246	5.61E-51	1.70E-49
ENSDARG00000055113	pom121	2.143017951	1.099643935	3.93E-56	1.42E-54
ENSDARG00000017820	polr3d	2.141641333	1.098716888	7.05E-32	1.04E-30
ENSDARG00000052550	nkx2.2b	2.14150124	1.098622513	2.50E-16	1.74E-15
ENSDARG00000091351	cnp	2.141158185	1.098391383	1.39E-29	1.86E-28
ENSDARG00000074622		2.13989531	1.097540217	3.08E-17	2.28E-16

ENSDARG00000012485	aurka	2.139759488	1.097448645	2.19E-55	7.75E-54
ENSDARG00000090714	gaa	2.139708399	1.097414198	3.56E-13	2.04E-12
ENSDARG00000093125	RGPD3	2.137808751	1.096132795	1.50E-38	2.91E-37
ENSDARG00000042533	gstm	2.13712138	1.09566885	3.48E-54	1.18E-52
ENSDARG00000008461	anapc5	2.13682982	1.095472014	3.29E-41	7.08E-40
ENSDARG00000086777	CABZ01055170.1	2.136656425	1.095354941	8.01E-41	1.70E-39
ENSDARG00000052617	raver1	2.135752858	1.094744713	5.17E-55	1.80E-53
ENSDARG00000017199	elp2	2.135701984	1.094710347	2.04E-42	4.66E-41
ENSDARG00000023950	znhit3	2.134796162	1.094098322	2.67E-09	1.15E-08
ENSDARG00000093713	si:dkey-256i11.2	2.133311237	1.093094461	1.83E-05	5.60E-05
ENSDARG00000098045	si:dkey-172f14.2	2.132762846	1.092723553	8.69E-10	3.92E-09
ENSDARG00000089858	COBLL1 (2 of 2)	2.131851582	1.092107002	7.14E-15	4.56E-14
ENSDARG00000097211	si:ch211-248g18.6	2.131678886	1.091990128	3.46E-10	1.60E-09
ENSDARG00000076237	CU302319.2	2.130862671	1.091437618	0.013717491	0.028418161
ENSDARG00000012929	eif2d	2.130448497	1.091157175	1.55E-53	5.14E-52
ENSDARG00000015070	pola2	2.130262334	1.091031104	2.44E-54	8.31E-53
ENSDARG00000095705	CDPF1	2.130256117	1.091026894	1.60E-32	2.43E-31
ENSDARG00000051756	pdhx	2.129828581	1.09073732	7.09E-57	2.58E-55
ENSDARG00000060304	map7d1a	2.129529117	1.090534456	1.50E-51	4.65E-50
ENSDARG00000031126	notum1a	2.127842131	1.089391118	1.51E-40	3.16E-39
ENSDARG00000076551	setd8b	2.127758212	1.089334219	1.33E-51	4.12E-50
ENSDARG00000055969	rfc3	2.126108399	1.088215154	1.78E-52	5.70E-51
ENSDARG00000025854	ddx51	2.126029133	1.088161366	1.35E-42	3.09E-41
ENSDARG00000067969	SPATS2 (1 of 2)	2.125489333	1.087795019	1.31E-40	2.75E-39
ENSDARG00000053301	insm1b	2.124054955	1.086821093	5.71E-52	1.80E-50
ENSDARG00000093283	CABZ01051641.2	2.123969855	1.08676329	0.013196451	0.027404674
ENSDARG00000004763	HHLA2 (1 of 4)	2.123773719	1.08663006	7.51E-06	2.39E-05
ENSDARG00000058656	desma	2.123706379	1.086584315	1.42E-16	1.01E-15
ENSDARG00000063276	msh3	2.123577401	1.086496694	1.71E-14	1.07E-13
ENSDARG00000041490	zgc:161979	2.12260184	1.085833774	2.49E-41	5.39E-40

ENSDARG00000017454	nup50	2.122001844	1.08542591	2.95E-45	7.49E-44
ENSDARG00000080531	SNORD31	2.121456997	1.085055435	0.001099947	0.002697695
ENSDARG00000038957	flvcr2a	2.120742464	1.084569435	1.23E-05	3.83E-05
ENSDARG00000056640	rpia	2.120713376	1.084549648	8.19E-55	2.84E-53
ENSDARG00000056111	lmod1	2.120546279	1.084435969	3.95E-21	3.62E-20
ENSDARG00000070109	ncapg	2.120540536	1.084432062	5.76E-42	1.28E-40
ENSDARG00000043105	qtrt1	2.120434987	1.08436025	4.72E-31	6.74E-30
ENSDARG00000006003	mycla	2.119377717	1.083640729	6.68E-56	2.40E-54
ENSDARG00000090936	ERICH2	2.118302883	1.082908886	9.62E-09	3.99E-08
ENSDARG00000079345	si:ch211-217k17.10	2.115182376	1.080782061	0.000329418	0.000867367
ENSDARG00000016496	cdk8	2.115167807	1.080772124	5.25E-54	1.76E-52
ENSDARG00000094558	si:dkeyp-53e4.1	2.113832947	1.079861368	0.000383068	0.001000378
ENSDARG00000054318	stk33	2.113201795	1.079430541	0.00019509	0.000528569
ENSDARG00000027857	cep97	2.112530585	1.078972229	5.65E-49	1.61E-47
ENSDARG00000089630	CABZ01087088.1	2.111199677	1.078063035	2.01E-21	1.87E-20
ENSDARG00000097714	si:ch1073-384e4.1	2.110880861	1.077845155	3.72E-06	1.22E-05
ENSDARG00000018574	sf3b4	2.109708777	1.077043864	6.57E-55	2.29E-53
ENSDARG00000073788	noa1	2.106383287	1.074767979	3.34E-26	3.90E-25
ENSDARG00000006392	exosc9	2.10458654	1.073536834	6.46E-40	1.32E-38
ENSDARG00000086639	siva1	2.104340423	1.073368111	1.44E-27	1.78E-26
ENSDARG00000083400	SNORA73	2.104310382	1.073347515	0.010084321	0.021382523
ENSDARG00000044402	nop16	2.104301702	1.073341564	1.15E-41	2.52E-40
ENSDARG00000096997	si:dkey-53i3.1	2.102983215	1.072437335	1.97E-12	1.07E-11
ENSDARG00000040252	atp1a1a.5	2.101268292	1.071260378	2.66E-22	2.58E-21
ENSDARG00000004455	p2rx5	2.100982353	1.071064044	1.48E-31	2.16E-30
ENSDARG00000096777	C12H16orf93	2.100885	1.070997193	0.014465723	0.029831222
ENSDARG00000067608	zswim7	2.100049521	1.070423348	7.16E-06	2.28E-05
ENSDARG00000021265	mybpc2b	2.099190969	1.069833418	2.08E-14	1.29E-13
ENSDARG00000075593	trim71	2.098084484	1.069072772	9.01E-46	2.32E-44
ENSDARG00000035634	rfc5	2.097595487	1.068736487	1.19E-52	3.85E-51

ENSDARG00000037378	qdprb1	2.096106831	1.067712248	1.02E-32	1.57E-31
ENSDARG00000096886	HEPHL1 (3 of 3)	2.095745056	1.067463226	2.88E-14	1.77E-13
ENSDARG00000071005	ppp1r3ca	2.095481741	1.067281951	4.11E-26	4.77E-25
ENSDARG00000033706	adora2aa	2.094853396	1.066849284	4.94E-08	1.93E-07
ENSDARG00000069388	tmem88b	2.093713299	1.066063901	1.08E-37	2.02E-36
ENSDARG00000023160	snrnp40	2.092936254	1.065528371	6.40E-53	2.08E-51
ENSDARG00000091383	CABZ01072242.1	2.09271351	1.065374822	3.86E-41	8.27E-40
ENSDARG00000086393	dbx1a	2.09243717	1.065184303	3.80E-42	8.51E-41
ENSDARG00000009743	efhc1	2.091821241	1.064759569	1.02E-15	6.89E-15
ENSDARG00000042370	PTAFR	2.091357801	1.064439907	7.06E-05	0.000202814
ENSDARG00000080337	AC024175.4	2.090654878	1.063954924	5.38E-12	2.83E-11
ENSDARG00000090070	CABZ01060490.2	2.090108568	1.063577883	0.016077927	0.032860174
ENSDARG00000023028	cdv3	2.089988237	1.063494822	4.96E-34	7.97E-33
ENSDARG00000078396	wdr46	2.089351421	1.063055169	4.76E-33	7.37E-32
ENSDARG00000016543	rnmtl1a	2.088581498	1.062523439	1.56E-16	1.11E-15
ENSDARG00000095698	si:dkey-228a15.1	2.087806653	1.061988113	7.63E-05	0.000218233
ENSDARG00000058891	nedd9	2.08701222	1.061439048	3.46E-07	1.26E-06
ENSDARG00000079748	ABHD15	2.086874454	1.061343811	0.010846502	0.022874018
ENSDARG00000008239	mocs3	2.086856644	1.061331499	1.89E-17	1.41E-16
ENSDARG00000075687	anapc1	2.086740837	1.061251436	9.18E-54	3.06E-52
ENSDARG00000053047	eif2s2	2.086325819	1.060964479	7.98E-22	7.52E-21
ENSDARG00000045695	myca	2.085953053	1.060706689	7.23E-52	2.26E-50
ENSDARG00000061850	CR847986.1	2.085427959	1.060343476	1.56E-16	1.10E-15
ENSDARG00000058488	adora2ab	2.085328203	1.060274462	7.08E-05	0.000203276
ENSDARG00000091350	CABZ01073112.1	2.085001534	1.060048445	6.24E-37	1.13E-35
ENSDARG00000069082	rnmtl1b	2.084848682	1.059942677	6.44E-22	6.11E-21
ENSDARG00000012222	nup35	2.084270809	1.059542739	2.43E-49	7.03E-48
ENSDARG00000090163	MYO18B (3 of 4)	2.083719298	1.059160942	0.000313973	0.000829157
ENSDARG00000070278	mettl14	2.083330713	1.058891875	7.10E-39	1.40E-37
ENSDARG00000044691	ppp1r3b	2.083008412	1.058668666	2.37E-24	2.53E-23

ENSDARG00000038754	plk3	2.082970776	1.058642599	4.02E-51	1.23E-49
ENSDARG00000087107	CFAP221	2.082928196	1.058613106	7.93E-05	0.000226398
ENSDARG00000016815	casc1	2.082495145	1.058313132	1.39E-06	4.75E-06
ENSDARG00000089307	pmaip1	2.082415056	1.058257648	8.50E-06	2.69E-05
ENSDARG00000092938	BX663505.2	2.081499816	1.057623431	0.002170758	0.005105775
ENSDARG00000089998	KIAA2012	2.081256697	1.057454914	2.16E-05	6.55E-05
ENSDARG00000089988	CCNA2	2.081078087	1.057331099	1.16E-48	3.25E-47
ENSDARG00000010732	xrcc4	2.080743217	1.057098934	1.14E-24	1.24E-23
ENSDARG00000016122	ptrhd1	2.080333101	1.05681455	3.18E-26	3.72E-25
ENSDARG00000031098	timm50	2.08007214	1.056633564	5.16E-49	1.47E-47
ENSDARG00000056438	her9	2.079927847	1.056533482	1.85E-49	5.38E-48
ENSDARG00000090568	MRPL52	2.079910295	1.056521307	2.44E-28	3.10E-27
ENSDARG00000077703	ehmt2	2.079580882	1.056292797	2.63E-53	8.67E-52
ENSDARG00000071164	alkbh1	2.079366631	1.056144155	7.89E-20	6.75E-19
ENSDARG00000056059	rangap1b	2.079159937	1.05600074	4.55E-53	1.49E-51
ENSDARG00000089697	nfe2l2b	2.078816828	1.055762643	3.36E-13	1.93E-12
ENSDARG00000070228	cdk6	2.078603785	1.055614784	3.38E-53	1.11E-51
ENSDARG00000088805	nes	2.077744258	1.055018089	5.95E-52	1.87E-50
ENSDARG00000091669	si:dkey-17e16.17	2.077706989	1.054992211	8.80E-44	2.13E-42
ENSDARG00000097705	dnah9	2.077139593	1.054598175	0.009430453	0.020092042
ENSDARG00000010130	smpdl3b	2.077137706	1.054596865	1.29E-33	2.05E-32
ENSDARG00000057062	wu:fc13c02	2.077032897	1.054524066	4.92E-51	1.50E-49
ENSDARG00000040727	tfb1m	2.076848962	1.054396301	1.30E-13	7.62E-13
ENSDARG00000006598	sgk2b	2.076840213	1.054390223	2.09E-42	4.76E-41
ENSDARG00000079457	C12H17orf70	2.07627181	1.053995323	9.01E-21	8.12E-20
ENSDARG00000089016	mapkapk3	2.075976508	1.053790118	6.38E-36	1.11E-34
ENSDARG00000068567	shha	2.074686017	1.052893015	4.78E-52	1.51E-50
ENSDARG00000034650	fabp7b	2.074394966	1.05269061	2.90E-24	3.09E-23
ENSDARG00000003519	aprt	2.074244661	1.052586073	2.05E-33	3.22E-32
ENSDARG00000017407	ttll12	2.072996128	1.051717422	4.93E-19	4.03E-18

ENSDARG00000002710	ncl	2.072526154	1.051390308	2.50E-08	1.00E-07
ENSDARG00000078800	slc26a10	2.072466729	1.051348941	1.03E-08	4.26E-08
ENSDARG00000040350	rpp38	2.07201978	1.051037775	6.43E-07	2.28E-06
ENSDARG00000057321	tut1	2.071613423	1.050754811	3.81E-37	6.94E-36
ENSDARG00000036227	npvf	2.071416719	1.050617818	4.99E-08	1.95E-07
ENSDARG00000037640	aurkb	2.070997386	1.050325732	1.22E-52	3.94E-51
ENSDARG00000021120	ndc1	2.070951505	1.050293771	1.31E-52	4.23E-51
ENSDARG00000037229	denr	2.070612472	1.050057569	1.77E-51	5.47E-50
ENSDARG00000097485	CFAP99	2.069865761	1.049537206	2.79E-17	2.07E-16
ENSDARG00000062806	ttll9	2.069849604	1.049525945	0.000768975	0.001927218
ENSDARG00000037910	FILIP1L (1 of 2)	2.069015362	1.048944357	6.14E-51	1.86E-49
ENSDARG00000077800	fancm	2.068623667	1.048671208	5.94E-18	4.55E-17
ENSDARG00000068916	PPP1R35	2.06859604	1.04865194	2.35E-10	1.11E-09
ENSDARG00000012274	eif4e1c	2.067970945	1.048215916	2.44E-48	6.82E-47
ENSDARG00000029764	mef2ca	2.067382693	1.04780547	3.00E-36	5.30E-35
ENSDARG00000017425	si:ch211-95g8.1	2.067187557	1.047669291	2.80E-20	2.45E-19
ENSDARG00000086657	si:dkey-29n10.3	2.065863131	1.046744675	0.013175438	0.02736904
ENSDARG00000087502		2.065701843	1.046632035	3.03E-09	1.31E-08
ENSDARG00000088764	lyplal1	2.06553809	1.046517665	2.89E-22	2.80E-21
ENSDARG00000094518	si:ch211-125e6.8	2.064914641	1.046082145	9.34E-27	1.12E-25
ENSDARG00000055443	zcchc7	2.063937402	1.045399215	7.60E-41	1.61E-39
ENSDARG00000030108	sec22ba	2.063467405	1.045070649	2.48E-44	6.13E-43
ENSDARG00000093792	BX469910.2	2.063459492	1.045065117	5.57E-05	0.000161707
ENSDARG00000076996	IPO13 (2 of 2)	2.063002093	1.044745285	7.08E-26	8.13E-25
ENSDARG00000061231	tinagl1	2.062061767	1.044087548	2.29E-52	7.30E-51
ENSDARG00000015352	lsg1	2.060809474	1.043211131	9.11E-52	2.84E-50
ENSDARG00000086998	zgc:64002	2.060668721	1.043112592	0.013191843	0.02739911
ENSDARG00000086305		2.0606511	1.043100255	8.81E-07	3.08E-06
ENSDARG00000071208	wnt4a	2.060472593	1.042975274	1.38E-27	1.71E-26
ENSDARG00000095863	zgc:161979	2.060119494	1.042728021	2.08E-17	1.55E-16

ENSDARG00000090806	GEMIN6	2.060055542	1.042683235	4.17E-17	3.06E-16
ENSDARG00000078606	C20H1orf131	2.059440444	1.042252406	4.50E-17	3.30E-16
ENSDARG00000054155	pcna	2.05936783	1.042201537	9.92E-17	7.13E-16
ENSDARG00000069723	zbbx	2.058946645	1.041906445	0.003369525	0.007713368
ENSDARG00000058606	SIK1	2.057864366	1.041147897	9.46E-42	2.09E-40
ENSDARG00000076411	ptrfa	2.057533781	1.040916117	5.54E-36	9.68E-35
ENSDARG00000015611	rasl11b	2.056984903	1.040531206	1.60E-30	2.23E-29
ENSDARG00000068993	TNFRSF14 (6 of 12)	2.056621553	1.040276342	2.68E-31	3.87E-30
ENSDARG00000030703	otx1a	2.056452912	1.040158038	2.77E-30	3.83E-29
ENSDARG00000034624	nuf2	2.055704542	1.039632927	2.29E-48	6.40E-47
ENSDARG00000088924	CENPU (2 of 2)	2.054957991	1.039108902	1.30E-19	1.10E-18
ENSDARG00000012674	rpusd4	2.054808485	1.039003936	1.05E-23	1.09E-22
ENSDARG00000008454	cpeb1b	2.054307163	1.038651912	0.000807335	0.002016776
ENSDARG00000076010	twist1b	2.05382801	1.038315374	8.89E-13	4.94E-12
ENSDARG00000008105	mettl2a	2.053043806	1.037764411	1.37E-43	3.29E-42
ENSDARG00000087710	SNORA3	2.052131496	1.037123179	0.002058514	0.004863104
ENSDARG00000094896	RBM33	2.051824907	1.036907624	0.003913748	0.008874099
ENSDARG00000011326	ankrd45	2.051072653	1.036378596	0.017635778	0.035738204
ENSDARG00000020114	slc20a1a	2.04983858	1.035510305	1.36E-37	2.52E-36
ENSDARG00000022013	lama2	2.049769517	1.035461697	9.45E-43	2.19E-41
ENSDARG00000040668	lrtomt	2.049633652	1.035366068	0.001246967	0.00303416
ENSDARG00000075944	nms	2.048435866	1.034522725	0.01762807	0.035727681
ENSDARG00000089591	WDR77	2.047510233	1.033870662	1.12E-42	2.58E-41
ENSDARG00000044329	opa1	2.047353034	1.033759894	1.35E-06	4.64E-06
ENSDARG00000046045	hoxc6b	2.046785102	1.033359638	1.85E-16	1.31E-15
ENSDARG00000078280	nkx3-1	2.045856582	1.032705013	0.001549548	0.00372513
ENSDARG00000040725	zgc:114130	2.045736655	1.032620441	1.02E-49	2.98E-48
ENSDARG00000012234	psme3	2.045401303	1.032383924	7.25E-51	2.20E-49
ENSDARG00000010246	prmt1	2.044824591	1.031977091	1.34E-24	1.45E-23
ENSDARG00000015930	rwdd1	2.044698059	1.031887816	1.32E-50	3.97E-49

ENSDARG00000018847	UTP20	2.044459918	1.031719779	2.84E-48	7.90E-47
ENSDARG00000068168	hes2.2	2.044097029	1.03146368	3.05E-32	4.57E-31
ENSDARG00000007885	fancl	2.043972476	1.031375769	2.31E-06	7.75E-06
ENSDARG00000029252	ssb	2.043919162	1.031338138	5.98E-41	1.27E-39
ENSDARG00000084628	SNORA27	2.042778134	1.030532522	0.010335824	0.021861964
ENSDARG00000022303	hig1	2.041960665	1.029955075	2.05E-50	6.13E-49
ENSDARG00000087954	rac1b	2.040842141	1.029164594	0.000269996	0.00071796
ENSDARG00000077900	si:ch211-141o9.10	2.04038612	1.028842192	2.45E-07	9.03E-07
ENSDARG00000075917	CFAP74	2.04009088	1.028633422	2.50E-12	1.35E-11
ENSDARG00000035750	ccnd1	2.039822439	1.028443575	1.10E-16	7.85E-16
ENSDARG00000087358	si:dkeyp-109b10.4	2.03958079	1.028272655	9.31E-12	4.84E-11
ENSDARG00000041904	ankzf1	2.037465196	1.026775416	1.56E-43	3.73E-42
ENSDARG00000091146		2.037430323	1.026750722	0.0080731	0.01739184
ENSDARG00000019503	zgc:103759	2.036269586	1.025928576	0.004107711	0.009277614
ENSDARG00000079672	EFTUD1	2.035022776	1.025044941	1.24E-19	1.05E-18
ENSDARG00000029445	EIF1B	2.034414806	1.024613867	2.57E-38	4.91E-37
ENSDARG00000088014	si:ch211-223a21.4	2.034379938	1.02458914	0.008632985	0.018502603
ENSDARG00000060256	alkbh8	2.034112099	1.024399187	4.38E-20	3.80E-19
ENSDARG00000030949	srprb	2.033651045	1.024072148	4.50E-49	1.29E-47
ENSDARG00000061548	si:ch211-145o7.3	2.033461915	1.023937971	1.33E-12	7.30E-12
ENSDARG00000032277	rars2	2.033087262	1.023672138	4.72E-19	3.86E-18
ENSDARG00000019156	haus5	2.031945602	1.02286178	2.76E-33	4.32E-32
ENSDARG00000059982	poc5	2.031167148	1.022308966	2.89E-20	2.53E-19
ENSDARG00000070868	CFAP126	2.031107792	1.022266806	1.56E-09	6.89E-09
ENSDARG00000089363	CABZ01002718.1	2.030697625	1.021975435	0.020492014	0.04095664
ENSDARG00000014976	lims2	2.030443557	1.021794923	2.95E-49	8.51E-48
ENSDARG00000016080	nob1	2.029842688	1.021367924	6.74E-50	1.98E-48
ENSDARG00000053021	grsf1	2.029755121	1.021305684	2.27E-46	5.94E-45
ENSDARG00000039863	lifrb	2.029093469	1.020835323	4.77E-15	3.08E-14
ENSDARG00000055252	SNAP23 (2 of 2)	2.028594063	1.0204802	3.33E-14	2.03E-13

ENSDARG00000012113	C19H1orf109	2.028374224	1.020323846	4.31E-12	2.29E-11
ENSDARG00000001859	dbx1b	2.027945737	1.02001905	5.20E-32	7.71E-31
ENSDARG00000010078	nup133	2.027735253	1.019869302	1.79E-46	4.70E-45
ENSDARG00000089083	itga7	2.027455019	1.019669907	3.32E-40	6.87E-39
ENSDARG00000020442	snx5	2.027110314	1.019424602	1.93E-45	4.93E-44
ENSDARG00000057010	si:dkey-154b15.1	2.026831071	1.019225851	5.37E-06	1.73E-05
ENSDARG00000052134	eri2	2.025356904	1.018176159	3.08E-16	2.13E-15
ENSDARG00000029866	slc6a14	2.02475574	1.017747876	0.000379319	0.000991226
ENSDARG00000087320	CABZ01017734.1	2.02361406	1.016934168	1.83E-08	7.40E-08
ENSDARG00000015222	cbll1	2.02183147	1.015662746	3.23E-34	5.23E-33
ENSDARG00000018264	trim101	2.020786546	1.014916939	1.33E-47	3.64E-46
ENSDARG00000079066	THBS1 (3 of 3)	2.019098182	1.013711066	7.59E-39	1.49E-37
ENSDARG00000033172	nr2f5	2.018703549	1.013429063	5.25E-43	1.23E-41
ENSDARG00000094191	nup37	2.01842489	1.013229902	1.39E-32	2.11E-31
ENSDARG00000042530	nup205	2.016929015	1.01216031	1.42E-35	2.45E-34
ENSDARG00000008247	vezf1a	2.015719765	1.011295082	2.34E-32	3.53E-31
ENSDARG00000074974	CEP192	2.015656566	1.011249849	1.35E-22	1.33E-21
ENSDARG00000006174	lrrc23	2.015122782	1.010867745	2.31E-05	6.97E-05
ENSDARG00000086976	USP30 (2 of 3)	2.015060957	1.010823482	0.016659695	0.033941769
ENSDARG00000091728	zgc:173587	2.014703975	1.010567876	1.17E-05	3.65E-05
ENSDARG00000068855	CCDC176 (2 of 2)	2.013664421	1.009823277	3.47E-05	0.000103158
ENSDARG00000046141	C8H12orf43	2.013486229	1.009695605	3.98E-14	2.42E-13
ENSDARG00000087285	TMEM182 (2 of 2)	2.013211155	1.009498496	2.50E-05	7.51E-05
ENSDARG00000058467	prpf40a	2.013183629	1.009478771	3.98E-34	6.42E-33
ENSDARG00000037309	pet112	2.013028015	1.00936725	1.78E-28	2.27E-27
ENSDARG00000077728	supv3l1	2.012987077	1.00933791	6.54E-42	1.45E-40
ENSDARG00000009461	hoxa9a	2.012699501	1.009131792	2.41E-18	1.89E-17
ENSDARG00000038399	polr3h	2.012206559	1.00877841	2.31E-41	5.00E-40
ENSDARG00000044479	eif2a	2.012032827	1.008653843	1.78E-48	5.00E-47
ENSDARG00000069775	FBXO40 (1 of 2)	2.011954212	1.008597472	1.53E-26	1.81E-25

ENSDARG00000093898	NLRP6 (116 of 145)	2.011149492	1.008020324	0.018530199	0.037364172
ENSDARG00000077698	rsad1	2.009028777	1.006498229	2.98E-06	9.85E-06
ENSDARG00000042345	si:dkeyp-55f12.3	2.008636338	1.006216389	3.01E-20	2.63E-19
ENSDARG00000016093	qtrtd1	2.008098311	1.005829902	9.31E-20	7.92E-19
ENSDARG00000089967	si:ch211-59o9.10	2.008025656	1.005777703	5.89E-40	1.21E-38
ENSDARG00000076913	eme1	2.007890711	1.005680746	2.00E-30	2.78E-29
ENSDARG00000060798	mrpl22	2.007860769	1.005659232	7.19E-39	1.42E-37
ENSDARG00000074007	wdr4	2.007814277	1.005625826	3.04E-20	2.66E-19
ENSDARG00000051984	zgc:113389	2.007548127	1.005434574	1.33E-10	6.38E-10
ENSDARG00000018797	crygmxl1	2.007261272	1.005228415	2.10E-11	1.06E-10
ENSDARG00000096049	si:ch211-120c15.1	2.006506734	1.004685998	0.00385712	0.008754082
ENSDARG00000039134	MTR	2.006035904	1.004347428	9.38E-48	2.57E-46
ENSDARG00000035665	CT027569.1	2.005367036	1.003866313	0.006593086	0.014425486
ENSDARG00000018891	rnaseh2a	2.005295264	1.003814678	4.30E-44	1.06E-42
ENSDARG00000094489	si:dkey-58f10.6	2.005173177	1.003726841	0.012585113	0.02626374
ENSDARG00000046098	ebp	2.005156185	1.003714615	1.41E-13	8.28E-13
ENSDARG00000093678	si:dkey-102m7.3	2.004856069	1.003498668	5.46E-22	5.20E-21
ENSDARG00000008490	ltv1	2.004798295	1.003457092	1.65E-48	4.64E-47
ENSDARG00000060394		2.003444449	1.002482508	7.93E-28	9.89E-27
ENSDARG00000069762	ccdc11	2.003419128	1.002464274	1.52E-08	6.19E-08
ENSDARG00000091754	CHCHD5	2.002851686	1.002055591	8.53E-10	3.85E-09
ENSDARG00000007065	mrps18b	2.002622507	1.0018905	8.41E-44	2.04E-42
ENSDARG00000045219	dkk1b	2.002399937	1.00173015	6.14E-21	5.56E-20
ENSDARG00000026281	lyrm1	2.001132543	1.000816726	2.46E-08	9.87E-08
ENSDARG00000078249	unc13d	2.001062749	1.000766408	4.69E-05	0.000137324
ENSDARG00000011683	prtfdc1	2.000864034	1.000623134	5.74E-39	1.14E-37
ENSDARG00000076221	zgc:198419	2.000745738	1.000537836	7.36E-36	1.28E-34
ENSDARG00000076904	loxl5b	2.000507166	1.000365797	2.12E-08	8.54E-08
ENSDARG00000061196	emilin2a	2.00025622	1.000184812	4.19E-13	2.39E-12
ENSDARG00000076068	crtc1a	0.499917927	-1.000236833	1.64E-12	8.97E-12

ENSDARG00000004648	kctd16a	0.499902569	-1.000281154	3.44E-20	3.00E-19
ENSDARG00000090614	BX005294.4	0.499881784	-1.00034114	1.53E-05	4.71E-05
ENSDARG00000077231	vwf	0.499881399	-1.000342249	5.38E-06	1.73E-05
ENSDARG00000095421	si:dkey-106l3.6	0.499841663	-1.000456935	2.83E-17	2.09E-16
ENSDARG00000058154	znf385c	0.499730795	-1.000776971	1.84E-43	4.38E-42
ENSDARG00000005786	ppm1f	0.499669216	-1.000954758	4.59E-06	1.49E-05
ENSDARG00000085803	5S_rRNA	0.499624614	-1.001083543	0.003481381	0.007954043
ENSDARG00000053110	pkib	0.49962151	-1.001092506	1.17E-19	9.93E-19
ENSDARG00000052769	glrbb	0.499614331	-1.001113235	2.16E-42	4.90E-41
ENSDARG00000040332	crfb2	0.499588692	-1.001187271	9.07E-10	4.08E-09
ENSDARG00000079024	rps6kl1	0.499470393	-1.001528932	7.60E-23	7.59E-22
ENSDARG00000037794	nptx2a	0.499458331	-1.001563773	0.004314191	0.009714623
ENSDARG00000080018	kif16bb	0.499377843	-1.001796284	3.33E-06	1.10E-05
ENSDARG00000068580	glis1a	0.499293275	-1.002040621	0.000186879	0.000507531
ENSDARG00000055585	c1galt1a	0.499259953	-1.002136907	2.78E-14	1.71E-13
ENSDARG00000053663	hmp19	0.499088671	-1.002631938	2.65E-39	5.32E-38
ENSDARG00000041797	cx28.9	0.499034036	-1.002789878	7.75E-06	2.46E-05
ENSDARG00000042565	MAP3K12	0.499029677	-1.002802482	4.15E-23	4.20E-22
ENSDARG00000097299	flrt2	0.498940699	-1.003059738	3.27E-10	1.52E-09
ENSDARG00000024708	SLC25A41 (2 of 2)	0.498849578	-1.00332324	2.14E-06	7.18E-06
ENSDARG00000074488	crfb13	0.498833052	-1.003371036	4.12E-15	2.68E-14
ENSDARG00000075823	ZNF608	0.498788132	-1.003500955	1.18E-38	2.30E-37
ENSDARG00000074546	si:ch211-213a13.11	0.498458743	-1.004453996	0.001178355	0.002878567
ENSDARG00000031598	calb1	0.49841729	-1.004573978	3.44E-18	2.67E-17
ENSDARG00000086101	CABZ01071530.1	0.497891244	-1.006097452	0.012644707	0.026372609
ENSDARG00000076501	asxl2	0.49781955	-1.006305207	2.05E-25	2.29E-24
ENSDARG00000090951	C19H1orf216	0.497730312	-1.006563845	1.09E-10	5.27E-10
ENSDARG00000071648	zgc:113298	0.497725756	-1.006577049	1.19E-06	4.11E-06
ENSDARG00000063357	GPR45	0.497473274	-1.007309073	0.000208598	0.00056275
ENSDARG00000028053	bmp1b	0.497468322	-1.007323435	6.88E-12	3.60E-11

ENSDARG00000040547	b3gat1a	0.497436241	-1.007416474	1.14E-13	6.71E-13
ENSDARG00000045033	bnip3	0.497353366	-1.007656853	2.93E-41	6.32E-40
ENSDARG00000040151	esrrb	0.497337724	-1.007702227	3.94E-14	2.40E-13
ENSDARG00000094377	samd10b	0.497219416	-1.00804546	5.64E-08	2.18E-07
ENSDARG00000096713	si:ch211-170d12.2	0.497082412	-1.008443037	2.53E-06	8.43E-06
ENSDARG00000035994	rims2b	0.496808562	-1.009238057	4.95E-30	6.77E-29
ENSDARG00000063704	gpr1	0.496760065	-1.009378896	9.91E-16	6.68E-15
ENSDARG00000055999	BRD8 (2 of 2)	0.496377056	-1.010491663	1.82E-12	9.89E-12
ENSDARG00000067760		0.496375714	-1.010495564	9.70E-30	1.31E-28
ENSDARG00000062562	egln2	0.496354927	-1.01055598	3.26E-29	4.31E-28
ENSDARG00000095301	si:dkey-253a1.2	0.496338237	-1.010604492	0.007877288	0.016998358
ENSDARG00000062865	MRAS	0.496261117	-1.010828674	1.78E-19	1.49E-18
ENSDARG00000007578	lppr2a	0.495653892	-1.012595035	2.57E-08	1.03E-07
ENSDARG00000091317	hunk	0.495489692	-1.013073051	1.95E-36	3.47E-35
ENSDARG00000088825	CIT (1 of 2)	0.495385849	-1.013375437	1.06E-05	3.31E-05
ENSDARG00000041145	paqr5a	0.495346227	-1.013490831	4.45E-05	0.000130654
ENSDARG00000078284	phkb	0.495265314	-1.013726509	1.89E-47	5.13E-46
ENSDARG00000058039	bhlhe22	0.49513769	-1.014098324	5.90E-49	1.68E-47
ENSDARG00000079908	tmem151a	0.495134027	-1.014108997	1.23E-18	9.83E-18
ENSDARG00000020798	necap1	0.495083985	-1.014254812	1.23E-43	2.96E-42
ENSDARG00000028541	si:ch211-121a2.4	0.494631468	-1.01557407	7.99E-05	0.000228209
ENSDARG00000087121	PCDHGC5 (10 of 31)	0.494606682	-1.015646364	6.68E-05	0.000192308
ENSDARG00000078238	NACC1	0.494579921	-1.015724424	0.005952651	0.013131419
ENSDARG00000015891	ptprea	0.49448017	-1.016015428	1.37E-32	2.09E-31
ENSDARG00000095665	si:ch211-191a16.2	0.494457792	-1.016080719	5.24E-10	2.40E-09
ENSDARG00000076591	snap91	0.494407829	-1.016226505	3.24E-49	9.34E-48
ENSDARG00000086342	zgc:101566	0.494369468	-1.01633845	2.37E-43	5.62E-42
ENSDARG00000086290	znf804b	0.494265413	-1.01664214	5.46E-14	3.29E-13
ENSDARG00000031343	rab6bb	0.494099891	-1.017125357	3.29E-49	9.47E-48
ENSDARG00000023082	krt1-19d	0.494000152	-1.01741661	4.56E-42	1.02E-40

ENSDARG00000071657	si:dkey-19a16.4	0.493967476	-1.017512041	0.006589041	0.014418755
ENSDARG00000045265	si:ch211-138k19.2	0.493928471	-1.017625964	3.43E-07	1.25E-06
ENSDARG00000059699	DNAJC16 (1 of 2)	0.493917879	-1.017656901	1.27E-10	6.10E-10
ENSDARG00000097528	si:dkey-7j14.5	0.493906975	-1.017688751	2.35E-39	4.72E-38
ENSDARG00000089061	CR853287.1	0.493858677	-1.017829837	0.008712726	0.018663653
ENSDARG00000094000	si:dkey-153k10.6	0.493828599	-1.017917706	3.97E-07	1.44E-06
ENSDARG00000061532	C25H15orf27	0.493722878	-1.018226597	3.53E-23	3.59E-22
ENSDARG00000044815	SERINC4	0.493711811	-1.018258935	6.96E-09	2.92E-08
ENSDARG00000052447	cryabb	0.493678837	-1.018355293	0.01754161	0.035575286
ENSDARG00000004415	tcf7l2	0.493673962	-1.018369541	1.31E-41	2.87E-40
ENSDARG00000035649	ext1c	0.493642468	-1.018461581	2.69E-30	3.72E-29
ENSDARG00000002600	pcsk1	0.493479341	-1.018938406	1.83E-17	1.37E-16
ENSDARG00000086575	CU929113.1	0.493281902	-1.019515738	0.001481962	0.003570506
ENSDARG00000015849	zgc:56628	0.493259644	-1.019580837	5.85E-05	0.000169493
ENSDARG00000005943	htra4	0.493195001	-1.019769918	6.67E-07	2.36E-06
ENSDARG00000028521	c1ql3b	0.493009692	-1.020312086	1.45E-09	6.42E-09
ENSDARG00000003989	crhr1	0.492993609	-1.02035915	1.86E-11	9.45E-11
ENSDARG00000059534	JPH3 (2 of 2)	0.492913398	-1.0205939	5.64E-44	1.38E-42
ENSDARG00000088505	fam217b	0.492876181	-1.020702833	3.46E-35	5.85E-34
ENSDARG00000061473	tbkbp1	0.492753401	-1.021062267	1.59E-18	1.26E-17
ENSDARG00000075956	FAXC (1 of 2)	0.492735187	-1.021115594	6.13E-14	3.68E-13
ENSDARG00000045886	slc38a2	0.492486023	-1.021845314	1.74E-41	3.80E-40
ENSDARG00000042861	ltk	0.492414366	-1.022055243	3.71E-28	4.68E-27
ENSDARG00000095833	alk	0.492339593	-1.022274332	6.70E-15	4.29E-14
ENSDARG00000060124	timm17b	0.492225591	-1.022608428	2.24E-05	6.77E-05
ENSDARG00000058162	pcyt1ba	0.492081715	-1.023030185	1.33E-15	8.87E-15
ENSDARG00000060532	adam22	0.491991372	-1.02329508	3.71E-49	1.07E-47
ENSDARG00000002241	KCNA2 (1 of 2)	0.491807093	-1.023835554	0.00483112	0.010799041
ENSDARG00000002607		0.491786229	-1.023896757	5.21E-37	9.46E-36
ENSDARG00000032737	gria3a	0.491353689	-1.025166207	1.88E-41	4.09E-40

ENSDARG00000079446		0.491193192	-1.02563753	6.32E-13	3.55E-12
ENSDARG00000022251	znf536	0.491125319	-1.025836895	2.71E-49	7.84E-48
ENSDARG00000013000	ppfia2	0.491115679	-1.025865213	7.02E-39	1.39E-37
ENSDARG00000069385	RGL4	0.491101711	-1.025906245	2.58E-47	6.97E-46
ENSDARG00000095522	si:dkey-71b5.3	0.490911314	-1.026465678	0.00576187	0.01273528
ENSDARG00000092825	EFCAB10	0.490876924	-1.026566746	0.001955215	0.004636802
ENSDARG00000086027	si:ch211-168d23.3	0.49086685	-1.026596355	3.94E-15	2.56E-14
ENSDARG00000035158	MCAM (2 of 3)	0.490824219	-1.026721656	9.62E-31	1.35E-29
ENSDARG00000090650		0.490761491	-1.026906047	1.96E-15	1.29E-14
ENSDARG00000095022	thsd7ba	0.490490909	-1.027701698	1.04E-22	1.03E-21
ENSDARG00000051730	slc7a10b	0.490483898	-1.027722319	2.48E-05	7.46E-05
ENSDARG00000078874		0.49047138	-1.02775914	0.01009204	0.021397294
ENSDARG00000071890	pcdh2g7	0.490389697	-1.027999426	0.016293414	0.033276651
ENSDARG00000075393	spock2	0.49037525	-1.028041929	3.96E-42	8.85E-41
ENSDARG00000070567	cadpsb	0.490225656	-1.028482106	9.87E-48	2.70E-46
ENSDARG00000095608	si:dkey-57k2.6	0.490210926	-1.028525455	0.006190553	0.013621326
ENSDARG00000060372	PLXNA2 (1 of 3)	0.489973933	-1.029223097	1.52E-49	4.41E-48
ENSDARG00000077357	LRRC61	0.489881355	-1.029495713	1.34E-08	5.52E-08
ENSDARG00000016348	DIP2B (2 of 2)	0.489846867	-1.02959728	1.20E-38	2.35E-37
ENSDARG00000069669	adra2c	0.489833248	-1.029637393	1.46E-07	5.48E-07
ENSDARG00000006093	cdk15	0.489716643	-1.029980867	2.27E-07	8.40E-07
ENSDARG00000095170	lrrfip1b	0.489711923	-1.029994774	1.19E-32	1.82E-31
ENSDARG00000025162	asic4a	0.489660483	-1.030146324	1.14E-10	5.47E-10
ENSDARG00000076299	EMILIN3 (1 of 2)	0.48958613	-1.030365408	5.70E-37	1.03E-35
ENSDARG00000007678	ttyh3b	0.48956734	-1.030420779	5.29E-35	8.87E-34
ENSDARG00000086650	si:dkeyp-27e10.6	0.48955004	-1.030471762	0.000732152	0.001841267
ENSDARG00000076768	REPS2	0.48939592	-1.030926022	3.84E-47	1.03E-45
ENSDARG00000073963	C4H12orf56	0.489358658	-1.031035872	7.73E-09	3.23E-08
ENSDARG00000077872	si:dkey-52d15.1	0.489297973	-1.031214789	3.78E-41	8.11E-40
ENSDARG00000035907	fam49a	0.489167568	-1.031599338	2.97E-46	7.73E-45

ENSDARG00000000189	sema6e	0.488972687	-1.032174214	2.63E-46	6.85E-45
ENSDARG00000076244	uo:ion006	0.488847161	-1.03254462	0.000162544	0.000445617
ENSDARG00000089829	CYTH3 (3 of 3)	0.488790353	-1.032712282	5.74E-05	0.000166299
ENSDARG00000097047	zgc:56112	0.488789615	-1.032714461	7.60E-05	0.000217456
ENSDARG00000088567	VPS72 (2 of 2)	0.488722272	-1.032913241	6.16E-08	2.37E-07
ENSDARG00000089605	CABZ01079081.1	0.488653297	-1.033116869	5.56E-05	0.00016142
ENSDARG00000089795	SERTAD4	0.488640891	-1.033153498	1.25E-33	1.99E-32
ENSDARG00000091306	kcnn1a	0.488593978	-1.033292012	9.16E-49	2.60E-47
ENSDARG00000095152	si:ch211-255i3.4	0.48843215	-1.033769928	0.000131897	0.000366119
ENSDARG00000074505	mturn	0.488417962	-1.033811838	0.000233339	0.000625342
ENSDARG00000086331	si:ch1073-351l9.1	0.488224176	-1.034384357	0.013408045	0.027811562
ENSDARG00000091269	si:ch73-27e22.8	0.488155904	-1.034586116	0.000532719	0.001363617
ENSDARG00000087273	IFT43	0.4881307	-1.034660605	0.002595879	0.006038702
ENSDARG00000012833	foxn3	0.488095104	-1.034765814	1.65E-29	2.20E-28
ENSDARG00000053272	RAP1GAP (1 of 3)	0.487905777	-1.035325528	6.67E-17	4.84E-16
ENSDARG00000007824	CR847998.1	0.487884223	-1.035389263	1.80E-50	5.38E-49
ENSDARG00000081800	CU929237.1	0.487794597	-1.035654316	0.013690894	0.028367196
ENSDARG00000078603	myo1hb	0.487777183	-1.035705821	0.00154619	0.003717371
ENSDARG00000034504	lmo1	0.487715092	-1.035889477	2.14E-48	6.00E-47
ENSDARG00000093774	rbp2b	0.487557128	-1.036356823	1.98E-40	4.12E-39
ENSDARG00000079687	rnf10	0.487550149	-1.036377473	2.10E-22	2.05E-21
ENSDARG00000060222	scn1ba	0.487473693	-1.03660373	7.81E-34	1.25E-32
ENSDARG00000092968		0.487451876	-1.0366683	0.024186226	0.047594447
ENSDARG00000069765	SYNGAP1 (2 of 2)	0.487173785	-1.037491592	2.13E-19	1.78E-18
ENSDARG00000094526	ksr2	0.48715114	-1.037558653	2.80E-09	1.21E-08
ENSDARG00000078250	zgc:194398	0.487105095	-1.03769502	4.53E-22	4.33E-21
ENSDARG00000092260	WBP1	0.48701526	-1.037961116	2.20E-19	1.83E-18
ENSDARG00000092997	si:ch211-89o5.1	0.486825853	-1.038522311	0.000176308	0.000480694
ENSDARG00000061255	dusp3a	0.48668553	-1.038938215	6.18E-34	9.88E-33
ENSDARG00000062884	MAP3K2	0.486617028	-1.03914129	1.38E-14	8.66E-14

ENSDARG00000061636	KCNJ9	0.486597119	-1.039200317	5.55E-07	1.98E-06
ENSDARG00000027512	asic1b	0.486513211	-1.039449114	1.50E-15	1.00E-14
ENSDARG00000020901	gabrp	0.486471198	-1.039573704	2.89E-18	2.26E-17
ENSDARG00000010023	CU856539.1	0.486255175	-1.040214492	2.62E-47	7.07E-46
ENSDARG00000068516	hapln1b	0.486183405	-1.040427446	2.48E-51	7.61E-50
ENSDARG00000092155	apoc2	0.486067149	-1.040772463	8.67E-21	7.82E-20
ENSDARG00000090443	CABZ01081904.1	0.486052011	-1.040817393	5.71E-21	5.19E-20
ENSDARG00000027852	plekhf1	0.486044669	-1.040839187	1.30E-21	1.22E-20
ENSDARG00000070844	gamt	0.485960044	-1.041090397	8.36E-15	5.32E-14
ENSDARG00000073824	RASGRF1	0.485787932	-1.041601445	1.81E-22	1.78E-21
ENSDARG00000051879	abcc8	0.485736218	-1.041755033	2.43E-34	3.96E-33
ENSDARG00000089549	BAALC	0.48565702	-1.04199028	8.31E-12	4.33E-11
ENSDARG00000039435	rgs17	0.485624445	-1.04208705	5.31E-39	1.05E-37
ENSDARG00000025747	mapk10	0.485607765	-1.042136604	4.09E-41	8.76E-40
ENSDARG00000079068	adam12	0.485519218	-1.042399693	3.75E-12	2.00E-11
ENSDARG00000042270	arsa	0.485397909	-1.042760201	1.56E-24	1.68E-23
ENSDARG00000044894	zgc:113307	0.485328556	-1.042966347	2.38E-29	3.16E-28
ENSDARG00000036308	nod1	0.485155484	-1.043480915	2.54E-05	7.64E-05
ENSDARG00000086333	CABZ01084952.1	0.485063783	-1.043753629	0.000213031	0.000573783
ENSDARG00000045556	OTUD7A	0.48493419	-1.044139122	1.13E-16	8.07E-16
ENSDARG00000051915	pde5ab	0.484759548	-1.04465878	1.86E-10	8.82E-10
ENSDARG00000074959	pcdh2g3	0.484714688	-1.044792295	0.008236174	0.017710922
ENSDARG00000071823	adarb2	0.484696757	-1.044845664	1.47E-09	6.50E-09
ENSDARG00000035791	si:busm1-71b9.3	0.484691717	-1.044860667	0.015822469	0.032382324
ENSDARG00000026406	anxa5a	0.484687211	-1.044874078	6.82E-09	2.86E-08
ENSDARG00000063159	si:dkey-32e23.4	0.484645738	-1.04499753	6.32E-26	7.28E-25
ENSDARG00000079151	CU459012.3	0.484507706	-1.045408483	3.40E-17	2.50E-16
ENSDARG00000055698	pcnxl2	0.484466184	-1.045532125	2.21E-47	5.98E-46
ENSDARG00000009568	CA10 (1 of 2)	0.484405297	-1.045713455	1.60E-07	5.99E-07
ENSDARG00000074145	BX119910.4	0.484387693	-1.045765884	0.004690764	0.010513425

ENSDARG00000014402	smc1b	0.483920093	-1.047159252	0.021619504	0.043025493
ENSDARG00000007561	cdh23	0.483695398	-1.047829282	0.000205199	0.000554002
ENSDARG00000056101	kcnd3	0.48335133	-1.048855882	2.10E-31	3.05E-30
ENSDARG00000025875	kctd2	0.483333392	-1.048909425	3.14E-16	2.17E-15
ENSDARG00000060415	arhgef28	0.483151597	-1.049452165	1.11E-48	3.13E-47
ENSDARG00000055302	grid2	0.483117915	-1.049552742	1.97E-26	2.32E-25
ENSDARG00000016531	klhl4	0.483024728	-1.049831046	1.09E-21	1.02E-20
ENSDARG00000020795	rac3b	0.482979333	-1.049966638	4.10E-50	1.22E-48
ENSDARG00000004964	cyp4t8	0.482970624	-1.049992654	5.23E-07	1.87E-06
ENSDARG00000058252	adamts15b	0.482862533	-1.050315572	0.007877002	0.016998358
ENSDARG00000077470	FAM163A (1 of 2)	0.482682132	-1.050854672	4.41E-08	1.73E-07
ENSDARG00000075817	METTL24	0.482584066	-1.051147815	6.85E-09	2.87E-08
ENSDARG00000015931		0.482194623	-1.052312532	6.45E-52	2.02E-50
ENSDARG00000063411	LRRC73	0.482114379	-1.052552636	1.72E-16	1.21E-15
ENSDARG00000087491	efr3a	0.481997309	-1.052903004	0.001189693	0.002903768
ENSDARG00000010042	dnm1a	0.481883872	-1.053242579	2.54E-47	6.86E-46
ENSDARG00000077430	CU695076.1	0.481783857	-1.053542041	2.25E-07	8.34E-07
ENSDARG00000063433	atp2b2	0.481692076	-1.053816904	1.98E-42	4.54E-41
ENSDARG00000019304	phactr3b	0.48166674	-1.053892789	2.94E-38	5.61E-37
ENSDARG00000070929	sox14	0.481521653	-1.05432742	3.09E-42	6.94E-41
ENSDARG00000078123	SLITRK3 (2 of 2)	0.481454733	-1.054527935	3.10E-22	3.00E-21
ENSDARG00000092659	ppp3r1a	0.481284341	-1.055038611	1.61E-52	5.15E-51
ENSDARG00000063695	FARP1 (1 of 2)	0.481205979	-1.055273528	1.98E-16	1.39E-15
ENSDARG00000058585	chst2b	0.481162398	-1.055404193	2.89E-19	2.39E-18
ENSDARG00000086300	FAM107A	0.480883656	-1.056240201	6.73E-29	8.77E-28
ENSDARG00000079227	plekhs1	0.480830689	-1.056399116	0.017815226	0.036065836
ENSDARG00000014675	efna3a	0.480782087	-1.05654495	1.34E-28	1.72E-27
ENSDARG00000078940	pcdh2g12	0.48076684	-1.056590702	4.12E-07	1.49E-06
ENSDARG00000079008	si:dkey-7f3.9	0.480750735	-1.056639031	1.08E-40	2.27E-39
ENSDARG00000004158	TMEM63C	0.480702837	-1.056782776	1.18E-30	1.66E-29

ENSDARG00000077115	si:ch73-44m9.1	0.480614182	-1.057048873	0.00269902	0.006266322
ENSDARG00000016213	BX088711.1	0.48057174	-1.057176282	2.29E-05	6.91E-05
ENSDARG00000089957	lgi2a	0.480545405	-1.057255343	2.65E-09	1.15E-08
ENSDARG00000052713	lrrtm1	0.48049102	-1.057418626	1.76E-43	4.19E-42
ENSDARG00000063144	shisa7	0.480479642	-1.057452791	3.94E-53	1.29E-51
ENSDARG00000058649	trim46b	0.480469479	-1.057483306	5.66E-29	7.40E-28
ENSDARG00000074519	si:dkey-224j12.3	0.480325912	-1.057914457	0.010574937	0.022329495
ENSDARG00000008788	camk1gb	0.480190104	-1.058322423	2.87E-11	1.44E-10
ENSDARG00000053205	ppfia4	0.480031885	-1.058797857	2.42E-23	2.49E-22
ENSDARG00000026335	susd4	0.480015238	-1.058847891	4.65E-14	2.82E-13
ENSDARG00000023933	skila	0.479958737	-1.059017714	4.58E-42	1.02E-40
ENSDARG00000014792	lrrc4bb	0.47993852	-1.059078485	4.10E-52	1.30E-50
ENSDARG00000056108	ndufa4	0.479884666	-1.059240381	1.04E-48	2.95E-47
ENSDARG00000017213	prss35	0.479864849	-1.059299959	9.45E-51	2.86E-49
ENSDARG00000087698	PCDHGC5 (12 of 31)	0.479758596	-1.059619439	0.000392754	0.001024351
ENSDARG00000078461	pde6c	0.479723469	-1.059725073	2.94E-15	1.93E-14
ENSDARG00000019525	tbcelb	0.479584373	-1.060143446	1.62E-28	2.08E-27
ENSDARG00000004577	zgc:194665	0.479550885	-1.060244188	6.46E-24	6.78E-23
ENSDARG00000038693	gng7	0.479514583	-1.060353404	3.05E-37	5.58E-36
ENSDARG00000038359	enosf1	0.479492644	-1.060419412	7.35E-48	2.02E-46
ENSDARG00000058255		0.479413742	-1.06065683	3.83E-22	3.67E-21
ENSDARG00000078740	il1rapl1b	0.479408029	-1.060674024	1.06E-09	4.76E-09
ENSDARG00000027903	ADAMTSL3	0.479403256	-1.060688389	4.54E-15	2.94E-14
ENSDARG00000091600	CR352229.1	0.479360811	-1.060816126	3.59E-47	9.66E-46
ENSDARG00000051908	opn4xb	0.479269815	-1.061090016	3.35E-06	1.10E-05
ENSDARG00000062889	FAM78B (1 of 2)	0.479194479	-1.061316808	0.001720589	0.004107469
ENSDARG00000059425	sntg2	0.479078463	-1.061666137	8.65E-05	0.00024603
ENSDARG00000086663	UNC79 (1 of 2)	0.478757327	-1.062633528	9.16E-13	5.08E-12
ENSDARG00000012204	CDK18	0.478631656	-1.063012278	1.88E-13	1.09E-12
ENSDARG00000020145	zgc:73340	0.478630726	-1.06301508	3.78E-11	1.89E-10

ENSDARG00000055874	cpe	0.478526935	-1.063327962	1.60E-30	2.23E-29
ENSDARG00000042859	SLC5A6 (2 of 2)	0.478311482	-1.063977669	4.91E-11	2.43E-10
ENSDARG00000024890		0.47822615	-1.064235076	5.85E-09	2.47E-08
ENSDARG00000086946	TOX2 (2 of 2)	0.478180487	-1.064372835	6.57E-06	2.10E-05
ENSDARG00000061481		0.477950315	-1.065067445	7.39E-32	1.09E-30
ENSDARG00000005670	ttbk2	0.477870857	-1.065307308	6.40E-26	7.37E-25
ENSDARG00000097783	si:ch211-278d18.5	0.477814765	-1.06547666	8.12E-06	2.57E-05
ENSDARG00000015880	slc6a5	0.477804743	-1.06550692	2.32E-27	2.83E-26
ENSDARG00000086825	CU861664.1	0.477779762	-1.06558235	6.41E-05	0.00018482
ENSDARG00000002510	dmbx1b	0.477762027	-1.065635902	2.33E-10	1.09E-09
ENSDARG00000062208	prrt1	0.477706285	-1.065804238	1.23E-50	3.70E-49
ENSDARG00000096611	taok2b	0.47764949	-1.065975769	0.002384049	0.005571016
ENSDARG00000060053	KCNH2 (2 of 2)	0.477632032	-1.066028501	1.64E-06	5.57E-06
ENSDARG00000091623	TMEM74 (2 of 2)	0.477574142	-1.066203371	1.44E-12	7.90E-12
ENSDARG00000074528	PRTFDC1 (2 of 2)	0.477486597	-1.066467856	1.20E-12	6.60E-12
ENSDARG00000042974	sypa	0.477372175	-1.066813617	4.68E-52	1.48E-50
ENSDARG00000000503	stx1b	0.477162466	-1.06744753	4.39E-21	4.02E-20
ENSDARG00000078581	crebrf	0.477161583	-1.067450202	1.01E-15	6.80E-15
ENSDARG00000039335	st6gal2a	0.477140621	-1.06751358	3.30E-47	8.90E-46
ENSDARG00000093112	espnl	0.47708642	-1.067677474	9.33E-06	2.94E-05
ENSDARG00000096420	si:dkey-13n23.4	0.476957644	-1.068066942	8.57E-07	3.00E-06
ENSDARG00000044015	gfra2b	0.476952232	-1.068083312	5.89E-11	2.89E-10
ENSDARG00000044577	chst8	0.476785857	-1.068586652	0.013078613	0.027189772
ENSDARG00000056957	GABRG1	0.476637564	-1.069035438	3.71E-09	1.59E-08
ENSDARG00000017437	adcy3b	0.476590536	-1.069177792	0.000100024	0.000282194
ENSDARG00000034808	kcnip1b	0.476540881	-1.069328111	4.08E-33	6.33E-32
ENSDARG00000013227	glulc	0.476534852	-1.069346362	0.003470227	0.007930471
ENSDARG00000040703	si:ch211-242n18.1	0.47651097	-1.069418667	6.30E-05	0.000181916
ENSDARG00000092281	flnb	0.475969888	-1.07105779	6.67E-21	6.04E-20
ENSDARG00000051852	kcnc1a	0.475777216	-1.071641909	2.52E-29	3.33E-28

ENSDARG00000002564	syngr1a	0.475650767	-1.072025392	2.16E-46	5.66E-45
ENSDARG00000071021	papss2a	0.475604692	-1.072165147	7.68E-38	1.44E-36
ENSDARG00000077399	UBASH3B (2 of 2)	0.475492305	-1.072506102	1.20E-10	5.75E-10
ENSDARG00000004322	USH1G (1 of 2)	0.475460902	-1.072601384	0.009587331	0.0204018
ENSDARG00000095312	si:ch211-74m13.1	0.475303502	-1.073079064	9.03E-07	3.15E-06
ENSDARG00000055158	prox1a	0.474872388	-1.074388223	2.37E-54	8.09E-53
ENSDARG00000097930	si:ch211-161c3.6	0.474812916	-1.074568915	5.75E-22	5.46E-21
ENSDARG00000090387	ONECUT2	0.474688175	-1.074947985	1.29E-11	6.65E-11
ENSDARG00000014215	cdh13	0.474593637	-1.075235335	3.06E-42	6.89E-41
ENSDARG00000087724	PCDHGC5 (13 of 31)	0.474274357	-1.076206229	0.000509834	0.001309586
ENSDARG00000052648	hs3st4	0.474115617	-1.076689181	0.000545035	0.001393636
ENSDARG00000060854	kctd3	0.473954374	-1.077179911	1.48E-28	1.90E-27
ENSDARG00000042418	wbscr17	0.473891103	-1.07737252	3.96E-17	2.91E-16
ENSDARG00000092903	C18H15orf48	0.473860039	-1.077467093	0.002743352	0.006364048
ENSDARG00000037607	NOL4L (1 of 2)	0.47376097	-1.077768746	8.79E-30	1.19E-28
ENSDARG00000097358	si:dkey-151m15.8	0.473681451	-1.078010917	7.20E-07	2.54E-06
ENSDARG00000062688	gpnmb	0.473669289	-1.078047958	1.63E-49	4.74E-48
ENSDARG00000058005	hgd	0.473265653	-1.079277871	5.99E-41	1.27E-39
ENSDARG00000087736	si:dkey-118k5.3	0.473212823	-1.079438926	2.41E-13	1.39E-12
ENSDARG00000061265	lmln	0.473088664	-1.079817504	7.72E-42	1.71E-40
ENSDARG00000091359	CAMSAP3 (2 of 2)	0.473056311	-1.079916168	6.23E-49	1.77E-47
ENSDARG00000070078	abcb11b	0.472923497	-1.080321271	8.83E-05	0.000251042
ENSDARG00000078548	GLP2R	0.47271659	-1.080952598	1.62E-05	4.97E-05
ENSDARG00000094417	si:dkey-13n15.2	0.472592863	-1.081330253	0.002910155	0.006727927
ENSDARG00000078302	BRINP1	0.472538805	-1.081495286	1.45E-28	1.87E-27
ENSDARG00000086475	SHISA6 (1 of 2)	0.472511108	-1.081579848	0.001340282	0.003248993
ENSDARG00000004305	vangl1	0.472276836	-1.082295318	3.39E-38	6.44E-37
ENSDARG00000069048	serpinf1	0.472072457	-1.082919783	1.51E-54	5.20E-53
ENSDARG00000037613	LGALS8 (2 of 2)	0.472049291	-1.082990582	1.06E-07	4.01E-07
ENSDARG00000060637	clstn2	0.472036264	-1.083030398	5.30E-36	9.27E-35

ENSDARG00000022437	CD81 (1 of 2)	0.472028601	-1.083053818	4.78E-35	8.02E-34
ENSDARG00000018066	ptchd1	0.471901574	-1.08344211	1.72E-16	1.21E-15
ENSDARG00000089877	C7H4orf48	0.471869231	-1.083540995	7.53E-55	2.61E-53
ENSDARG00000014891	robo2	0.471677437	-1.084127505	2.45E-51	7.54E-50
ENSDARG00000078588	CABZ01081821.1	0.47159385	-1.084383192	0.002129701	0.005017927
ENSDARG00000067824	CNTNAP3	0.471551896	-1.084511542	3.11E-36	5.48E-35
ENSDARG00000088669	PCDHGC5 (22 of 31)	0.471481627	-1.084726544	4.02E-07	1.45E-06
ENSDARG00000053383	rprma	0.471464305	-1.084779548	0.00049298	0.001268525
ENSDARG00000039497	ccdc85a	0.471456187	-1.08480439	1.54E-12	8.40E-12
ENSDARG00000026820	gc3	0.471094845	-1.085910551	6.29E-12	3.31E-11
ENSDARG00000031540	tmem200a	0.471065827	-1.085999419	2.16E-10	1.02E-09
ENSDARG00000075139	hdac5	0.471041612	-1.08607358	2.06E-33	3.24E-32
ENSDARG00000074564	fam57bb	0.47102788	-1.086115639	3.31E-18	2.58E-17
ENSDARG00000079396	LRFN5 (2 of 2)	0.471001262	-1.086197169	2.62E-41	5.66E-40
ENSDARG00000087102	si:ch1073-164k15.3	0.470984288	-1.086249162	4.95E-08	1.93E-07
ENSDARG00000037815	b4galt5	0.470912028	-1.086470522	0.000834259	0.00208074
ENSDARG00000090179	si:ch211-117c9.1	0.470893545	-1.086527147	0.003139343	0.007231305
ENSDARG00000077465	SORCS2	0.470475974	-1.087807045	6.21E-49	1.77E-47
ENSDARG00000068247	luzp2	0.4703252	-1.088269462	8.70E-39	1.71E-37
ENSDARG00000069297	upf3a	0.470270509	-1.088437232	2.84E-43	6.72E-42
ENSDARG00000002197	pygl	0.470179292	-1.088717093	4.38E-51	1.33E-49
ENSDARG00000060865	scai	0.470041524	-1.089139882	1.90E-18	1.50E-17
ENSDARG00000071709	CT971495.1	0.470030625	-1.089173336	6.08E-07	2.16E-06
ENSDARG00000088096	CABZ01043829.1	0.469982251	-1.089321822	0.010323965	0.021840132
ENSDARG00000074723	myo10l1	0.469975201	-1.089343461	3.32E-39	6.63E-38
ENSDARG00000070586	ctgfb	0.469850321	-1.089726859	9.23E-11	4.46E-10
ENSDARG00000089394	CABZ01081333.1	0.469785183	-1.089926884	0.000181116	0.000492539
ENSDARG00000009922	dmbx1a	0.469477058	-1.090873435	5.77E-45	1.45E-43
ENSDARG00000035309	entpd3	0.469420279	-1.091047927	1.26E-51	3.91E-50
ENSDARG00000042948	dnajc5aa	0.469379945	-1.091171894	1.24E-40	2.60E-39

ENSDARG00000005332	lipi	0.469315139	-1.091371096	0.006276748	0.013800298
ENSDARG00000097328	si:ch73-305a18.1	0.469262535	-1.091532811	0.000530468	0.001358712
ENSDARG00000056478	ACAP2 (1 of 2)	0.469192493	-1.091748164	2.01E-45	5.13E-44
ENSDARG00000075046	RNF151	0.468949245	-1.092496309	0.000175379	0.000478403
ENSDARG00000091408	MTCL1	0.468921262	-1.0925824	2.53E-27	3.08E-26
ENSDARG00000071055	ZNF512B	0.468872079	-1.092733725	5.03E-07	1.80E-06
ENSDARG00000060609	CD109	0.468866029	-1.092752341	1.49E-14	9.33E-14
ENSDARG00000056105	syt11b	0.468714547	-1.093218524	4.60E-46	1.19E-44
ENSDARG00000014652	ankrd13b	0.468591919	-1.093596021	2.40E-32	3.61E-31
ENSDARG00000089588	BX324216.2	0.468574163	-1.093650689	0.001472929	0.003549947
ENSDARG00000097262	si:ch211-255f14.2	0.468391392	-1.094213531	1.15E-10	5.52E-10
ENSDARG00000001154	rimbp2	0.468299328	-1.094497128	9.87E-17	7.10E-16
ENSDARG00000088736	si:ch211-250n8.1	0.468276379	-1.094567829	0.000443469	0.001148089
ENSDARG00000058492	si:dkey-30c15.17	0.468164861	-1.09491144	2.79E-08	1.11E-07
ENSDARG00000062314	rasl10a	0.467443726	-1.0971354	0.000260206	0.000693324
ENSDARG00000031855	mst1	0.467324052	-1.097504805	1.00E-42	2.32E-41
ENSDARG00000086183	vkorc1	0.467276407	-1.097651899	1.73E-15	1.15E-14
ENSDARG00000091916	ugt5b4	0.466928349	-1.098726912	1.15E-35	1.98E-34
ENSDARG00000089817	NPTN (2 of 2)	0.466891354	-1.098841223	2.43E-21	2.25E-20
ENSDARG00000045420	epdr1	0.466837393	-1.099007971	1.17E-25	1.33E-24
ENSDARG00000056768	rprml	0.466806951	-1.099102053	7.87E-09	3.28E-08
ENSDARG00000093730	si:dkey-253d23.1	0.466755062	-1.099262426	0.000155251	0.000427106
ENSDARG00000097235	si:dkeyp-34b8.3	0.466703014	-1.09942331	4.84E-05	0.000141669
ENSDARG00000091116	pkhd1l1	0.466689582	-1.099464832	2.72E-55	9.59E-54
ENSDARG00000063307	SGSM2	0.466472767	-1.100135236	7.57E-22	7.15E-21
ENSDARG00000087364	si:ch73-140j24.4	0.46646942	-1.100145589	4.51E-43	1.06E-41
ENSDARG00000053358	basp1	0.466427746	-1.100274482	4.46E-38	8.43E-37
ENSDARG00000014280	dlgap1a	0.466398304	-1.100365552	3.02E-07	1.10E-06
ENSDARG00000061409	bivm	0.466250577	-1.100822585	1.08E-25	1.23E-24
ENSDARG00000041323	csdc2a	0.466122609	-1.101218604	3.60E-36	6.34E-35

ENSDARG00000087040	rhbdl1	0.465857582	-1.102039121	1.33E-07	5.02E-07
ENSDARG00000094172	si:ch73-263f13.1	0.465803986	-1.10220511	5.86E-05	0.000169784
ENSDARG00000077656	si:ch211-71k24.5	0.465433177	-1.103354041	3.36E-21	3.09E-20
ENSDARG00000077539	BX119910.7	0.465212852	-1.104037142	2.49E-07	9.17E-07
ENSDARG00000095034	C24H8orf34	0.465151141	-1.104228529	6.49E-09	2.73E-08
ENSDARG00000092455	si:ch211-246e12.3	0.464979774	-1.104760133	0.020927339	0.041765013
ENSDARG00000044605	stx12l	0.464977701	-1.104766564	7.19E-42	1.59E-40
ENSDARG00000075792	iqsec3a	0.464837893	-1.105200413	6.12E-19	4.98E-18
ENSDARG00000011487	gnaq	0.464705338	-1.105611877	2.50E-22	2.43E-21
ENSDARG00000035508	barhl1a	0.464703031	-1.105619039	2.53E-32	3.80E-31
ENSDARG00000068274	ANKRD65	0.464573744	-1.106020475	4.80E-08	1.88E-07
ENSDARG00000009930	cadm2a	0.464125332	-1.107413654	4.84E-18	3.72E-17
ENSDARG00000061692	yjefn3	0.464015833	-1.107754063	5.96E-20	5.13E-19
ENSDARG00000079583	ppp2r2bb	0.463944577	-1.107975624	1.38E-22	1.36E-21
ENSDARG00000038855	chmp5a	0.463724472	-1.108660232	1.44E-22	1.42E-21
ENSDARG00000060545	TMC8	0.46361344	-1.109005704	0.012636327	0.026359692
ENSDARG00000026484	rab15	0.463424519	-1.109593717	4.10E-36	7.20E-35
ENSDARG00000094677		0.463362996	-1.109785258	0.005615138	0.012432238
ENSDARG00000077468	si:ch73-206p6.1	0.463313236	-1.109940196	9.57E-23	9.50E-22
ENSDARG00000089847		0.463272356	-1.110067497	7.19E-05	0.000206363
ENSDARG00000057159	ankrd29	0.46297253	-1.111001501	2.61E-12	1.40E-11
ENSDARG00000097706	si:dkey-11a7.3	0.462959229	-1.111042949	1.99E-25	2.23E-24
ENSDARG00000074652	smtna	0.462839816	-1.111415117	0.000909833	0.002256357
ENSDARG00000035578	hs3st1l2	0.462544915	-1.112334632	3.26E-23	3.33E-22
ENSDARG00000078416	zeb2b	0.462507126	-1.112452501	2.98E-53	9.80E-52
ENSDARG00000076679	RAI1	0.462337447	-1.112981876	3.05E-54	1.03E-52
ENSDARG00000059881	fam69b	0.462241053	-1.1132827	1.02E-34	1.68E-33
ENSDARG00000038185	gh1	0.462173431	-1.113493771	1.32E-08	5.43E-08
ENSDARG00000002816	rasgrf2b	0.462017423	-1.113980837	3.14E-06	1.04E-05
ENSDARG00000096979	NPC2 (2 of 2)	0.462001104	-1.114031797	1.33E-32	2.02E-31

ENSDARG00000061446	smap2	0.46187515	-1.114425169	9.32E-09	3.87E-08
ENSDARG00000062519	abcc13	0.461485485	-1.115642822	1.37E-24	1.48E-23
ENSDARG00000067683	PDE1C (2 of 2)	0.461419447	-1.115849286	0.01125437	0.023672668
ENSDARG00000089252	CABZ01058048.1	0.461362916	-1.11602605	1.67E-24	1.80E-23
ENSDARG00000004291	hecw1b	0.46133927	-1.116099992	1.22E-40	2.57E-39
ENSDARG00000095412	si:dkey-183p4.9	0.461115865	-1.116798791	0.018580916	0.037453147
ENSDARG00000008720	lmo3	0.461003823	-1.11714938	5.27E-57	1.93E-55
ENSDARG00000079014	sema4f	0.460840888	-1.117659369	8.36E-18	6.36E-17
ENSDARG00000063412	cers1	0.460815426	-1.117739081	6.69E-32	9.87E-31
ENSDARG00000088432	si:dkey-162h11.3	0.460793344	-1.117808217	4.34E-11	2.15E-10
ENSDARG00000056262	slc35g2a	0.460628332	-1.118324945	1.24E-32	1.88E-31
ENSDARG00000079348	GRIN2B (2 of 2)	0.460596062	-1.11842602	6.41E-29	8.35E-28
ENSDARG00000038366	cyp2k18	0.460563786	-1.118527116	0.000426215	0.001106143
ENSDARG00000053499	isl2b	0.460491789	-1.118752663	6.15E-37	1.11E-35
ENSDARG00000094118	si:dkey-21o22.2	0.460457061	-1.118861469	0.004710299	0.010553045
ENSDARG00000074739	slitrk3b	0.460223378	-1.119593826	2.34E-14	1.44E-13
ENSDARG00000079343	si:ch211-241b2.4	0.460212715	-1.119627252	0.000858988	0.002139038
ENSDARG00000053673		0.460138872	-1.119858757	1.52E-16	1.07E-15
ENSDARG00000055791	SLC3A2 (3 of 3)	0.460034697	-1.120185419	6.46E-44	1.57E-42
ENSDARG00000087096	CR847847.1	0.459997245	-1.120302874	0.007784939	0.016815682
ENSDARG00000075592	tnfaip8l2a	0.459924543	-1.120530908	0.000140945	0.000389521
ENSDARG00000059348	rxfp3.3b	0.459842478	-1.120788352	0.000521787	0.001338528
ENSDARG00000061436	col6a2	0.459766472	-1.121026831	8.47E-36	1.47E-34
ENSDARG00000006983	celf3b	0.459723439	-1.121161871	6.02E-52	1.89E-50
ENSDARG00000075724	pcdh2g1	0.459672511	-1.1213217	0.012031834	0.025203516
ENSDARG00000061051	BRINP3 (1 of 3)	0.459596634	-1.121559862	1.53E-11	7.85E-11
ENSDARG00000059535	LRRTM4 (1 of 4)	0.459193738	-1.122825128	0.00026881	0.000715074
ENSDARG00000077940	VSTM2B	0.459002376	-1.123426475	1.86E-33	2.94E-32
ENSDARG00000056092		0.458666499	-1.12448256	1.60E-57	5.96E-56
ENSDARG00000053454	CABZ01012080.1	0.458530152	-1.12491149	1.25E-09	5.59E-09

ENSDARG00000074921	FAM117B (2 of 2)	0.458523725	-1.12493171	2.25E-55	7.93E-54
ENSDARG00000069095	gls2	0.458302576	-1.1256277	3.32E-10	1.54E-09
ENSDARG00000077514	SLITRK1	0.458289635	-1.125668437	2.96E-40	6.14E-39
ENSDARG00000076757	EPHB1	0.458178442	-1.126018515	6.00E-15	3.85E-14
ENSDARG00000079302	and2	0.458164832	-1.126061371	4.83E-13	2.73E-12
ENSDARG00000067692	CDH18 (2 of 2)	0.458040256	-1.126453695	1.22E-12	6.70E-12
ENSDARG00000056797	ppp2r2c	0.458025745	-1.126499404	1.05E-54	3.64E-53
ENSDARG00000006832	GALNT9	0.45792703	-1.12681037	6.75E-37	1.22E-35
ENSDARG00000052155	LRRC8A (2 of 2)	0.457450246	-1.128313257	8.04E-36	1.40E-34
ENSDARG00000059775	slc32a1	0.457343936	-1.128648573	1.41E-59	5.53E-58
ENSDARG00000069038	si:ch211-242e8.1	0.457284815	-1.128835083	0.00110974	0.002720305
ENSDARG00000003403	tenm1	0.457204143	-1.129089618	3.32E-57	1.23E-55
ENSDARG00000093349	si:ch211-141e20.2	0.457173828	-1.129185281	7.15E-07	2.52E-06
ENSDARG00000042107	CNGB1 (1 of 2)	0.456771916	-1.130454143	3.21E-05	9.57E-05
ENSDARG00000086902	DIRAS2	0.45642356	-1.131554833	0.001509065	0.00363211
ENSDARG00000014953	cnih3	0.456378579	-1.131697017	6.13E-17	4.46E-16
ENSDARG00000086207	grin2da	0.456281098	-1.132005207	8.59E-27	1.03E-25
ENSDARG00000069101	NAPB (2 of 2)	0.455784787	-1.133575325	1.98E-31	2.88E-30
ENSDARG00000092680	si:dkey-58f10.12	0.455649551	-1.13400345	1.11E-09	4.96E-09
ENSDARG00000042477	nr2c2	0.455582911	-1.134214463	5.74E-45	1.44E-43
ENSDARG00000036878	sh3bgrl2	0.455299543	-1.135112082	2.67E-27	3.25E-26
ENSDARG00000008674	chrnb2a	0.455117728	-1.13568831	0.003920573	0.008887445
ENSDARG00000025108	magixa	0.454875922	-1.136455023	5.47E-42	1.22E-40
ENSDARG00000096920	SRRM4	0.454871163	-1.136470119	1.12E-14	7.09E-14
ENSDARG00000077687	rp1	0.454819022	-1.1366355	0.000163587	0.00044813
ENSDARG00000079559	FAM196A (3 of 3)	0.454798929	-1.136699239	0.003438264	0.00786312
ENSDARG00000076076	crtc1b	0.454780282	-1.136758391	5.22E-08	2.03E-07
ENSDARG00000026084	KCNS1	0.454706718	-1.136991776	0.001049738	0.002584817
ENSDARG00000013647	cntn3a.2	0.454649915	-1.137172013	0.000274881	0.000730402
ENSDARG00000073704	si:dkeyp-72g9.4	0.454630578	-1.137233374	3.71E-54	1.25E-52

ENSDARG00000087955	si:ch211-127b16.4	0.454535686	-1.137534528	1.49E-07	5.60E-07
ENSDARG00000038981		0.454522452	-1.137576533	2.31E-41	5.00E-40
ENSDARG00000078645	CAMKK2	0.454417655	-1.137909207	8.87E-54	2.96E-52
ENSDARG00000009477	prkar2ab	0.454353778	-1.138112018	5.89E-10	2.68E-09
ENSDARG00000020292		0.454328887	-1.138191056	7.34E-16	4.97E-15
ENSDARG00000030311	TMC2 (1 of 2)	0.454275597	-1.138360287	0.002510489	0.00585108
ENSDARG00000092730	si:dkey-22i16.3	0.4542311	-1.138501608	1.88E-30	2.61E-29
ENSDARG00000035532	zgc:110329	0.454162661	-1.138718994	2.17E-42	4.94E-41
ENSDARG00000091498	BX571715.2	0.454155409	-1.138742032	3.65E-19	3.01E-18
ENSDARG00000079204	adam11	0.453986775	-1.139277825	3.98E-37	7.25E-36
ENSDARG00000079249	OGDHL	0.453921115	-1.139486495	1.98E-06	6.66E-06
ENSDARG00000044551	atg9a	0.453894097	-1.139572369	4.31E-47	1.16E-45
ENSDARG00000089271	si:dkey-114c15.7	0.453644942	-1.140364522	3.28E-59	1.28E-57
ENSDARG00000043012	napg	0.453598591	-1.140511935	2.15E-44	5.32E-43
ENSDARG00000018809	abhd3	0.453487427	-1.140865542	6.56E-31	9.32E-30
ENSDARG00000087599	TENM3 (2 of 2)	0.453395883	-1.141156802	4.10E-31	5.88E-30
ENSDARG00000089465	CABZ01048956.1	0.453341564	-1.141329657	2.84E-30	3.92E-29
ENSDARG00000092813	FNDC7 (8 of 8)	0.453233714	-1.141672913	1.57E-14	9.79E-14
ENSDARG00000057394	rab33a	0.453101499	-1.142093832	4.29E-15	2.78E-14
ENSDARG00000089696		0.453089101	-1.142133308	1.08E-10	5.18E-10
ENSDARG00000096327	cd164l2	0.453003286	-1.142406581	1.18E-07	4.45E-07
ENSDARG00000007654	nsfa	0.452843239	-1.142916377	3.08E-58	1.16E-56
ENSDARG00000036567	aanat1	0.452817893	-1.142997126	0.00415357	0.00937821
ENSDARG00000011929	plp1b	0.452762692	-1.143173011	2.27E-07	8.38E-07
ENSDARG00000062106	klf5b	0.452414911	-1.144281614	0.004190612	0.009452081
ENSDARG00000079540	CACNA2D2 (3 of 3)	0.452370379	-1.144423631	1.15E-11	5.93E-11
ENSDARG00000087431	zgc:173962	0.452297294	-1.144656731	0.001126743	0.002759366
ENSDARG00000005780	npy8br	0.451986549	-1.145648256	2.71E-05	8.14E-05
ENSDARG00000089933	rasal3	0.451874384	-1.146006318	0.000154563	0.000425253
ENSDARG00000020450	cacng8a	0.451850819	-1.146081556	1.18E-24	1.28E-23

ENSDARG00000097474	si:ch211-158e19.1	0.451801137	-1.146240194	0.000731262	0.00183919
ENSDARG00000007663	amph	0.45178119	-1.14630389	8.14E-61	3.28E-59
ENSDARG00000059677	mamdc2a	0.451772616	-1.146331269	8.89E-15	5.64E-14
ENSDARG00000023903	atf2	0.451761517	-1.146366715	8.93E-12	4.65E-11
ENSDARG00000074308	fam211ba	0.451624649	-1.146803867	1.63E-34	2.67E-33
ENSDARG00000063345	creg1	0.451575064	-1.146962273	7.61E-50	2.24E-48
ENSDARG00000097406	si:dkey-81e3.2	0.451397564	-1.147529462	3.72E-15	2.42E-14
ENSDARG00000041609	adarb1a	0.451160767	-1.14828648	1.29E-48	3.62E-47
ENSDARG00000071091	chrm3a	0.450985737	-1.148846288	7.27E-09	3.04E-08
ENSDARG00000040781	zgc:113317	0.4508994	-1.149122504	3.50E-26	4.08E-25
ENSDARG00000068008	arhgap44	0.450876492	-1.149195804	4.27E-27	5.14E-26
ENSDARG00000089449	KAZN (3 of 3)	0.450851537	-1.149275656	0.003302234	0.007571306
ENSDARG00000057921	ccdc136b	0.450647845	-1.149927604	1.31E-55	4.67E-54
ENSDARG00000097730	si:ch1073-443n13.3	0.450620628	-1.150014739	4.21E-12	2.23E-11
ENSDARG00000011693	ttc36	0.450604299	-1.150067019	1.97E-10	9.30E-10
ENSDARG00000061918	cplx2	0.450507828	-1.150375921	4.75E-57	1.74E-55
ENSDARG00000017266	scml4	0.450419289	-1.150659485	2.05E-44	5.09E-43
ENSDARG00000074089	BX119910.2	0.450209438	-1.151331794	1.42E-05	4.38E-05
ENSDARG00000058601	gdap1	0.450106112	-1.151662939	4.81E-28	6.03E-27
ENSDARG00000074310	rbfox3l	0.44973662	-1.152847735	1.71E-05	5.25E-05
ENSDARG00000010158	nbeab	0.449715693	-1.152914867	7.27E-25	7.97E-24
ENSDARG00000062812	glsa	0.449548341	-1.153451834	3.16E-43	7.47E-42
ENSDARG00000076480	mcf2lb	0.449407948	-1.153902454	9.52E-31	1.34E-29
ENSDARG00000060393	fibcd1	0.449406156	-1.153908209	2.13E-17	1.58E-16
ENSDARG00000076972	si:dkey-208k22.3	0.449404734	-1.153912774	2.29E-07	8.47E-07
ENSDARG00000058445	PDILT	0.449328106	-1.154158787	1.17E-08	4.83E-08
ENSDARG00000055183	etv1	0.449173217	-1.154656188	2.90E-14	1.78E-13
ENSDARG00000087295	ZFYVE9 (3 of 3)	0.449080441	-1.154954207	0.004778117	0.010689486
ENSDARG00000070467	lactbl1b	0.448986423	-1.155256276	2.03E-07	7.53E-07
ENSDARG00000057649	satb1a	0.448978772	-1.155280859	8.79E-45	2.20E-43

ENSDARG00000061421	BRSK2 (1 of 2)	0.448890995	-1.155562939	4.66E-08	1.82E-07
ENSDARG00000077986	TMEM151A (1 of 2)	0.448829996	-1.155759	2.16E-08	8.69E-08
ENSDARG00000025106	ppp3cb	0.44881235	-1.15581572	3.49E-47	9.39E-46
ENSDARG00000093851	si:ch73-332k15.1	0.448663639	-1.156293828	1.09E-13	6.43E-13
ENSDARG00000098024	si:dkey-262k9.2	0.448656782	-1.156315876	3.70E-35	6.24E-34
ENSDARG00000075830	syt3	0.448408858	-1.157113318	2.06E-38	3.97E-37
ENSDARG00000074521	map6a	0.448214406	-1.157739077	1.34E-15	8.93E-15
ENSDARG00000007856	rtn4rl1a	0.448181334	-1.157845532	0.001137399	0.002784261
ENSDARG00000026095	FP102191.1	0.448066709	-1.158214557	0.0017453	0.004162962
ENSDARG00000060085	kcnq3	0.447991659	-1.158456222	9.59E-15	6.07E-14
ENSDARG00000031647	stat2	0.447974588	-1.158511199	5.49E-35	9.20E-34
ENSDARG00000056019	TTBK1 (1 of 2)	0.447844252	-1.158931005	1.75E-40	3.65E-39
ENSDARG00000030761	syt16	0.447557354	-1.15985552	3.22E-25	3.58E-24
ENSDARG00000058966	zgc:112332	0.4472291	-1.160914032	1.56E-19	1.31E-18
ENSDARG00000062634	kat2b	0.447001175	-1.161649471	6.98E-24	7.32E-23
ENSDARG00000056833	svopa	0.446841866	-1.162163733	5.42E-18	4.17E-17
ENSDARG00000092456	si:ch211-188i4.4	0.446780747	-1.162361077	1.18E-08	4.87E-08
ENSDARG00000058892	cep170b	0.446496098	-1.163280528	5.64E-23	5.68E-22
ENSDARG00000096307	si:ch73-379f7.5	0.446374075	-1.163674855	3.22E-06	1.06E-05
ENSDARG00000055839	MARCH4 (1 of 2)	0.446346392	-1.163764329	1.09E-10	5.23E-10
ENSDARG00000096853	si:dkey-103g5.7	0.446339394	-1.163786952	0.015515778	0.031818824
ENSDARG00000077847	olfm2a	0.446320084	-1.163849368	2.18E-53	7.20E-52
ENSDARG00000078981	faxcb	0.446141096	-1.164428048	9.24E-20	7.86E-19
ENSDARG00000095819	AMER3	0.44580503	-1.165515199	2.81E-09	1.21E-08
ENSDARG00000076373	vopp1	0.445733495	-1.165746716	1.03E-50	3.12E-49
ENSDARG00000020581	OTOF (1 of 2)	0.445710686	-1.165820544	1.17E-46	3.09E-45
ENSDARG00000074543	unc5a	0.445606974	-1.166156282	7.12E-07	2.51E-06
ENSDARG00000076028	sez6l	0.445480374	-1.166566221	6.55E-32	9.67E-31
ENSDARG00000078542	inpp5ja	0.445344052	-1.167007771	1.03E-05	3.23E-05
ENSDARG00000039502	eef1a1a	0.445275145	-1.16723101	7.22E-50	2.13E-48

ENSDARG00000043406	slc8a1b	0.445050643	-1.167958582	2.28E-42	5.17E-41
ENSDARG00000058392	adcy2a	0.445013114	-1.168080242	9.83E-14	5.83E-13
ENSDARG00000008275	klhl24b	0.445010485	-1.168088768	1.40E-58	5.38E-57
ENSDARG00000044591	CT025910.1	0.444965955	-1.168233137	2.41E-39	4.84E-38
ENSDARG00000040261	runx2a	0.444940881	-1.168314435	0.006102186	0.013440853
ENSDARG00000097526	si:ch73-112l6.6	0.444819344	-1.168708566	0.001124887	0.002755295
ENSDARG00000059988	gpr153	0.444743404	-1.168954886	1.44E-05	4.45E-05
ENSDARG00000043673	BEGAIN (1 of 2)	0.444573807	-1.169505142	5.98E-28	7.48E-27
ENSDARG00000088925	ATP9A	0.4445163	-1.169691774	1.89E-23	1.95E-22
ENSDARG00000078466	cetn2	0.444499884	-1.169745052	7.75E-22	7.32E-21
ENSDARG00000059523	RIMS4	0.444330162	-1.170296017	1.80E-09	7.90E-09
ENSDARG00000092722	UNC5D (3 of 3)	0.444275192	-1.17047451	6.39E-07	2.26E-06
ENSDARG00000091411	si:ch211-214p13.9	0.443945911	-1.171544181	0.015566462	0.031911246
ENSDARG00000079251	nlgn2b	0.443717197	-1.172287627	4.07E-28	5.12E-27
ENSDARG00000030687	PHKA2	0.443713582	-1.17229938	4.78E-54	1.61E-52
ENSDARG00000018619	CERS4 (1 of 2)	0.443599023	-1.172671908	9.63E-16	6.49E-15
ENSDARG00000076401	cacng3b	0.443469287	-1.173093901	4.99E-40	1.03E-38
ENSDARG00000079891	dnajc6	0.44340679	-1.173297234	1.60E-64	7.07E-63
ENSDARG00000089195	pcdh2g20	0.443385044	-1.173367988	0.000698314	0.001760532
ENSDARG00000053761	si:dkey-187j14.4	0.443280192	-1.173709199	2.36E-06	7.90E-06
ENSDARG00000077771	pcdh2g28	0.443265479	-1.173757083	3.15E-05	9.41E-05
ENSDARG00000059272	KBTBD11	0.443263196	-1.173764513	3.51E-28	4.42E-27
ENSDARG00000004869	penka	0.443222255	-1.173897771	7.18E-08	2.75E-07
ENSDARG00000075048	lonrf1	0.443099474	-1.174297481	1.50E-34	2.47E-33
ENSDARG00000077755	PCDHGC4 (7 of 9)	0.443047107	-1.174467993	2.25E-18	1.77E-17
ENSDARG00000093064	si:dkey-92j12.6	0.443033632	-1.174511873	2.20E-07	8.15E-07
ENSDARG00000035544	etnppl	0.442971049	-1.174715682	2.08E-38	3.98E-37
ENSDARG00000076639	esamb	0.442930509	-1.174847723	1.82E-07	6.77E-07
ENSDARG00000063361	si:ch211-1e14.1	0.442915783	-1.174895687	6.70E-26	7.70E-25
ENSDARG00000040048	GALNTL6	0.442706117	-1.175578788	2.16E-36	3.83E-35

ENSDARG00000040430	nptxra	0.442647669	-1.175769269	4.59E-30	6.28E-29
ENSDARG00000076460	bai1a	0.442421911	-1.176505258	5.19E-57	1.90E-55
ENSDARG00000088444	CU929133.1	0.44235822	-1.176712964	5.50E-22	5.23E-21
ENSDARG00000039884	CABZ01068274.1	0.44235245	-1.176731783	1.38E-52	4.44E-51
ENSDARG00000053448	si:ch211-251f6.6	0.442119698	-1.177491081	2.03E-22	1.98E-21
ENSDARG00000008861	tfap2e	0.441973464	-1.177968342	1.63E-46	4.30E-45
ENSDARG00000079231	slc7a14b	0.441905466	-1.17819032	0.001676463	0.004010555
ENSDARG00000075713	shox2	0.441746094	-1.178710717	9.81E-48	2.69E-46
ENSDARG00000018259	atp1a3a	0.44165455	-1.179009721	1.15E-25	1.30E-24
ENSDARG00000041179	crygm5	0.441652866	-1.179015221	1.04E-11	5.37E-11
ENSDARG00000039352	pald1b	0.441570598	-1.179283981	8.99E-27	1.07E-25
ENSDARG00000015002	cdh4	0.441429048	-1.179746527	7.68E-52	2.40E-50
ENSDARG00000037933	kcnc3b	0.441315537	-1.180117556	2.17E-11	1.10E-10
ENSDARG00000077994	b4galt2	0.44127714	-1.180243084	6.49E-19	5.27E-18
ENSDARG00000019579	ldb2a	0.441150506	-1.180657155	2.21E-26	2.60E-25
ENSDARG00000033845	igsf9ba	0.44108904	-1.18085818	3.75E-64	1.64E-62
ENSDARG00000021820	SLC45A4 (1 of 2)	0.441034227	-1.181037471	5.55E-09	2.35E-08
ENSDARG00000034321	palmdb	0.441011737	-1.181111044	0.000140438	0.000388195
ENSDARG00000007247	ric8a	0.440966065	-1.181260457	1.46E-37	2.71E-36
ENSDARG00000077124	BRSK1 (1 of 2)	0.440781914	-1.181863065	1.99E-15	1.31E-14
ENSDARG00000015964	zgc:153311	0.44073977	-1.182001013	5.69E-05	0.000164887
ENSDARG00000056618	xk	0.440682883	-1.182187235	1.81E-20	1.61E-19
ENSDARG00000006849	asic2	0.44031187	-1.183402357	3.08E-30	4.23E-29
ENSDARG00000080015	lrrtm4l1	0.44023759	-1.18364576	0.000100743	0.000284027
ENSDARG00000038731	slc25a36a	0.440189993	-1.183801746	2.94E-64	1.29E-62
ENSDARG00000007195	GRM2 (2 of 2)	0.440158332	-1.183905518	1.02E-47	2.78E-46
ENSDARG00000060322	zgc:153654	0.440142801	-1.183956425	0.005360391	0.011903461
ENSDARG00000069291	CU184872.1	0.440057273	-1.184236794	1.10E-07	4.16E-07
ENSDARG00000078998	lhfpl4a	0.440051235	-1.184256589	6.81E-60	2.70E-58
ENSDARG00000092970	si:ch211-11c15.3	0.439963163	-1.184545359	9.68E-53	3.13E-51

ENSDARG00000062976	iffo1a	0.439527703	-1.185973994	6.28E-10	2.86E-09
ENSDARG00000044748	or115-12	0.439382619	-1.186450293	0.012913424	0.026879788
ENSDARG00000031387	cers4a	0.439329896	-1.186623419	7.69E-28	9.60E-27
ENSDARG00000067841	ccdc177	0.439304515	-1.186706768	2.88E-35	4.89E-34
ENSDARG00000037145	slc8a4b	0.43925285	-1.186876447	1.47E-10	7.01E-10
ENSDARG00000017490	cel.1	0.439230242	-1.186950705	2.01E-05	6.12E-05
ENSDARG00000094522	si:ch211-14c7.2	0.439214601	-1.18700208	1.39E-38	2.70E-37
ENSDARG00000069600	zgc:109889	0.439087027	-1.187421184	7.28E-66	3.35E-64
ENSDARG00000042613	crp3	0.438816758	-1.188309473	0.010583434	0.022345023
ENSDARG00000081299	si:ch211-152f6.6	0.438630052	-1.188923436	4.10E-66	1.91E-64
ENSDARG00000078125	rusc1	0.438575811	-1.18910185	2.32E-61	9.52E-60
ENSDARG00000005095	glcea	0.438570155	-1.189120457	2.22E-26	2.61E-25
ENSDARG00000096639	si:ch211-101l18.8	0.438367148	-1.189788409	1.07E-06	3.70E-06
ENSDARG00000054003	rargb	0.43829648	-1.190021001	7.69E-17	5.57E-16
ENSDARG00000079468	RHOBTB1	0.438187636	-1.190379316	9.49E-09	3.93E-08
ENSDARG00000023822	zgc:73349	0.438164546	-1.190455343	2.62E-05	7.88E-05
ENSDARG00000041569	ces2	0.438115577	-1.190616586	7.60E-25	8.32E-24
ENSDARG00000077608	GPR137B (1 of 2)	0.438115077	-1.190618232	5.00E-08	1.95E-07
ENSDARG00000027082	hmbox1a	0.437891387	-1.191355021	2.36E-10	1.11E-09
ENSDARG00000006607	gpr27	0.43785078	-1.191488815	7.58E-35	1.26E-33
ENSDARG00000076103	grid2ipa	0.437838763	-1.191528408	1.14E-05	3.55E-05
ENSDARG00000075271	rapgef5a	0.437816134	-1.191602974	6.41E-58	2.40E-56
ENSDARG00000053323	zgc:112285	0.437777563	-1.191730081	4.20E-53	1.37E-51
ENSDARG00000005775	scn8aa	0.437733763	-1.191874431	1.28E-61	5.24E-60
ENSDARG00000059067	jupb	0.437552698	-1.192471312	2.78E-13	1.60E-12
ENSDARG00000071192	cx40.8	0.437545272	-1.192495796	4.55E-06	1.48E-05
ENSDARG00000091792	akap12a	0.437447778	-1.192817295	5.83E-38	1.10E-36
ENSDARG00000032623	rcan3	0.437282288	-1.193363182	8.31E-54	2.78E-52
ENSDARG00000061647	nrxn1a	0.437101709	-1.193959075	2.46E-60	9.82E-59
ENSDARG00000025648	fibpb	0.437036749	-1.194173497	0.017541168	0.035575286

ENSDARG00000063158	SLC24A2 (2 of 2)	0.437007332	-1.194270609	6.10E-13	3.43E-12
ENSDARG00000011042	pgbd5	0.436665256	-1.19540035	1.98E-16	1.39E-15
ENSDARG00000025974	MAGI3 (1 of 2)	0.43656651	-1.195726634	4.99E-10	2.29E-09
ENSDARG00000088900	si:dkey-21o19.2	0.436564801	-1.195732281	2.39E-36	4.23E-35
ENSDARG00000016771	tfa	0.436369743	-1.196377023	2.32E-07	8.57E-07
ENSDARG00000036584	st8sia5	0.436276254	-1.196686144	1.58E-64	7.00E-63
ENSDARG00000075161	defbl1	0.436021071	-1.19753024	7.35E-62	3.04E-60
ENSDARG00000060711	SV2B (2 of 3)	0.435802418	-1.198253892	6.12E-52	1.92E-50
ENSDARG00000004282	zgc:77375	0.435801054	-1.198258411	2.08E-53	6.88E-52
ENSDARG00000077112	sc:d0413	0.435763127	-1.19838397	9.98E-27	1.19E-25
ENSDARG00000078151	GRIK5 (2 of 2)	0.43575077	-1.19842488	7.91E-26	9.05E-25
ENSDARG00000091715	si:dkey-162h11.2	0.435536077	-1.199135867	6.15E-35	1.03E-33
ENSDARG00000045904	nr2e3	0.435518839	-1.199192969	3.88E-48	1.07E-46
ENSDARG00000071011	cdk5r2a	0.43536781	-1.199693354	1.26E-64	5.62E-63
ENSDARG00000034229	kcnip3a	0.435355158	-1.199735279	0.00905004	0.019322122
ENSDARG00000058876	mmp16b	0.435223434	-1.200171856	1.62E-25	1.82E-24
ENSDARG00000028354	stxbp6l	0.435220437	-1.200181791	1.13E-09	5.04E-09
ENSDARG00000069438	neurl1aa	0.435114825	-1.200531921	4.65E-31	6.63E-30
ENSDARG00000059885	frmd3	0.435058648	-1.200718197	8.54E-13	4.75E-12
ENSDARG00000076878	fam196ab	0.435044856	-1.200763934	4.42E-05	0.000129841
ENSDARG00000043858	cdk19	0.435006564	-1.200890925	1.12E-13	6.60E-13
ENSDARG00000086084	CU571170.2	0.434908513	-1.201216147	0.016607206	0.033842111
ENSDARG00000054378	pcbp3	0.43484298	-1.201433551	1.28E-31	1.87E-30
ENSDARG00000045945	syn2a	0.434536427	-1.202450971	3.01E-67	1.45E-65
ENSDARG00000093232	si:dkey-202p8.1	0.434477013	-1.202648246	1.60E-10	7.60E-10
ENSDARG00000075803		0.434454163	-1.202724121	1.56E-22	1.53E-21
ENSDARG00000097322	si:dkey-81l17.6	0.434108193	-1.203873444	3.18E-19	2.63E-18
ENSDARG00000008127	pcdh15b	0.433966932	-1.20434298	1.19E-17	8.97E-17
ENSDARG00000063354	ABTB1	0.433894908	-1.204582441	1.09E-27	1.35E-26
ENSDARG00000061110	zbtb44	0.433620864	-1.20549392	1.21E-15	8.09E-15

ENSDARG00000086739	CU929219.1	0.433466601	-1.206007258	7.83E-23	7.81E-22
ENSDARG00000090615	BX957297.2	0.433434991	-1.206112468	2.00E-08	8.09E-08
ENSDARG00000036577	atp6v0cb	0.433358296	-1.206367773	8.32E-43	1.94E-41
ENSDARG00000091641	CABZ01007222.1	0.433060455	-1.207359656	8.03E-35	1.33E-33
ENSDARG00000089334	ednrb1a	0.43303105	-1.207457618	4.22E-50	1.25E-48
ENSDARG00000037122	TENM2 (2 of 2)	0.432808135	-1.208200478	7.81E-65	3.49E-63
ENSDARG00000077596	si:ch211-269m17.1	0.432796343	-1.208239787	3.38E-35	5.72E-34
ENSDARG00000036428	EMP3 (1 of 2)	0.432713253	-1.208516786	9.30E-56	3.33E-54
ENSDARG00000092671	si:ch1073-345a8.1	0.432437794	-1.209435478	0.001451403	0.003501038
ENSDARG00000079618	sik2a	0.432350677	-1.209726147	8.46E-07	2.96E-06
ENSDARG00000016584	rgs7	0.432180216	-1.210295065	2.11E-36	3.74E-35
ENSDARG00000060656	si:ch211-10a23.2	0.431747667	-1.211739714	2.58E-48	7.19E-47
ENSDARG00000027497	mast1a	0.431712244	-1.211858085	2.08E-21	1.93E-20
ENSDARG00000039256	speg	0.431697745	-1.211906539	2.72E-62	1.14E-60
ENSDARG00000086991	pbx1a	0.43162636	-1.21214512	1.51E-32	2.29E-31
ENSDARG00000088934	BX322612.1	0.431532415	-1.212459162	0.013011146	0.027063374
ENSDARG00000074902	NOL4L (2 of 2)	0.431308256	-1.213208762	1.45E-29	1.94E-28
ENSDARG00000026979	krt1-c5	0.431225976	-1.213484011	3.09E-41	6.65E-40
ENSDARG00000087586	pcdh2ab2	0.4312245	-1.213488949	0.000123877	0.000345036
ENSDARG00000097170	pcdh1a3	0.431071058	-1.21400239	1.37E-54	4.73E-53
ENSDARG00000037805	lgals3bpa	0.431069905	-1.214006251	0.022759241	0.045085291
ENSDARG00000088913	CABZ01006980.1	0.430888793	-1.214612521	0.02274777	0.04506885
ENSDARG00000055754	smc1a	0.430600557	-1.215577909	2.86E-32	4.29E-31
ENSDARG00000037373	ephb2a	0.430581856	-1.215640566	8.31E-34	1.33E-32
ENSDARG00000087974	pcdh2ab12	0.43035425	-1.216403377	2.71E-07	9.95E-07
ENSDARG00000087681	DRAXIN (2 of 2)	0.430304391	-1.216570532	7.63E-09	3.19E-08
ENSDARG00000076601	arhgef9b	0.430291799	-1.216612751	9.98E-10	4.48E-09
ENSDARG00000059616	hs3st2	0.430097401	-1.217264681	5.53E-10	2.52E-09
ENSDARG00000012460	rassf4	0.430032081	-1.217483802	1.86E-24	1.99E-23
ENSDARG00000035555	gbgt1l3	0.429949202	-1.217761878	2.40E-13	1.39E-12

ENSDARG00000062400	LRRN2	0.429284303	-1.219994675	3.53E-06	1.16E-05
ENSDARG00000087131	ifnlr1	0.429273442	-1.220031174	0.002300588	0.005390177
ENSDARG00000077572	si:ch211-193k19.2	0.429235165	-1.22015982	0.000114516	0.000320315
ENSDARG00000052657	si:dkey-90l8.3	0.42918626	-1.220324204	2.01E-09	8.79E-09
ENSDARG00000062325	shank2	0.428964416	-1.221070119	1.71E-31	2.49E-30
ENSDARG00000045143	hbbe2	0.428830796	-1.221519581	5.17E-69	2.57E-67
ENSDARG00000043806	postna	0.428604836	-1.222279968	4.60E-66	2.13E-64
ENSDARG00000019861	fgl2	0.428493075	-1.222656205	4.94E-10	2.26E-09
ENSDARG00000058243	phactr3a	0.428445042	-1.222817939	1.27E-43	3.05E-42
ENSDARG00000055479		0.42843554	-1.222849933	0.007883377	0.017006332
ENSDARG00000068214	ccni	0.428367816	-1.223078002	3.27E-31	4.70E-30
ENSDARG00000053547	jakmip2	0.428329338	-1.223207599	1.64E-58	6.25E-57
ENSDARG00000090808		0.428310132	-1.22327229	1.03E-05	3.23E-05
ENSDARG00000067798	ppapdc1a	0.428295412	-1.223321872	0.007879135	0.016998472
ENSDARG00000019686		0.428172756	-1.223735092	0.015312383	0.031431205
ENSDARG00000061835	zpld1a	0.427943779	-1.224506819	6.24E-08	2.40E-07
ENSDARG00000076804	ttyh1	0.427818339	-1.224929766	6.52E-68	3.17E-66
ENSDARG00000069748	capn5b	0.427759827	-1.225127097	8.55E-07	2.99E-06
ENSDARG00000061644	ensab	0.427722468	-1.225253102	2.31E-29	3.06E-28
ENSDARG00000076218	b3galt4	0.427425229	-1.226256027	9.17E-08	3.49E-07
ENSDARG00000077606	ARSI (2 of 2)	0.427412701	-1.226298316	1.44E-13	8.43E-13
ENSDARG00000079153	CABZ01066573.1	0.427354173	-1.226495885	1.20E-06	4.11E-06
ENSDARG00000043609	epor	0.427327918	-1.22658452	4.46E-07	1.60E-06
ENSDARG00000055589	s100t	0.427104121	-1.227340277	8.36E-48	2.30E-46
ENSDARG00000033426		0.427079159	-1.227424598	5.14E-08	2.00E-07
ENSDARG00000063475	abcg1	0.427065505	-1.227470723	3.29E-10	1.53E-09
ENSDARG00000044278	synpr	0.427009164	-1.227661062	3.02E-08	1.20E-07
ENSDARG00000025595	agmo	0.426970393	-1.22779206	1.91E-13	1.11E-12
ENSDARG00000017780	rorcb	0.426953321	-1.227849746	4.12E-31	5.90E-30
ENSDARG00000077162	AL935029.1	0.42689303	-1.228053488	8.42E-64	3.65E-62

ENSDARG00000045685	cntn1b	0.426876611	-1.228108976	4.58E-49	1.31E-47
ENSDARG00000070960	si:ch211-288g17.4	0.426798526	-1.2283729	0.024130523	0.047500384
ENSDARG00000007869	ehd3	0.426719601	-1.228639715	1.98E-44	4.91E-43
ENSDARG00000074419	hcn4l	0.42668889	-1.228743548	2.28E-08	9.18E-08
ENSDARG00000033411	cabp1b	0.426611587	-1.229004945	0.024118214	0.047479443
ENSDARG00000078339	CT573264.3	0.426420861	-1.229650077	0.005829485	0.012876564
ENSDARG00000088810	prox1b	0.426392197	-1.229747058	1.15E-34	1.90E-33
ENSDARG00000060350	APOD (3 of 3)	0.426151801	-1.230560667	2.29E-20	2.02E-19
ENSDARG00000032098	c8g	0.426004491	-1.231059455	1.07E-32	1.63E-31
ENSDARG00000090275	CDHR5 (2 of 2)	0.425883204	-1.23147026	5.76E-05	0.000166821
ENSDARG00000015404	arl3l2	0.425780474	-1.231818304	3.20E-10	1.49E-09
ENSDARG00000091659	LPL (2 of 2)	0.425776986	-1.231830122	7.36E-30	9.99E-29
ENSDARG00000096834	si:ch211-155k24.9	0.425735623	-1.231970285	6.55E-22	6.21E-21
ENSDARG00000020866	APOA4 (1 of 4)	0.425650291	-1.23225948	8.90E-53	2.88E-51
ENSDARG00000084796	5S_rRNA	0.425549995	-1.232599459	0.003723119	0.008465508
ENSDARG00000028118	dscamb	0.425503861	-1.232755873	3.99E-70	2.04E-68
ENSDARG00000027070	acbd4	0.425491647	-1.232797286	6.12E-05	0.000176762
ENSDARG00000059026	zgc:123217	0.425478808	-1.232840818	1.10E-07	4.17E-07
ENSDARG00000091264	FRMPD1 (4 of 5)	0.425457872	-1.232911807	3.75E-05	0.000110954
ENSDARG00000087426	C1H20orf27 (2 of 2)	0.425356952	-1.233254062	8.15E-24	8.51E-23
ENSDARG00000024017	mamdc1	0.425237434	-1.233659491	7.91E-33	1.22E-31
ENSDARG00000079102	PRRG3	0.425181028	-1.233850871	1.11E-06	3.84E-06
ENSDARG00000094124	si:dkey-259n11.3	0.425176289	-1.233866951	0.0064534	0.014152092
ENSDARG00000089367	PRRT2	0.42497659	-1.234544722	1.53E-70	7.95E-69
ENSDARG00000091444	tubb4b	0.424897589	-1.234812937	1.33E-09	5.92E-09
ENSDARG00000079617	zgc:101560	0.424877902	-1.234879784	6.32E-06	2.03E-05
ENSDARG00000020178	nova1	0.424866127	-1.234919768	4.74E-70	2.42E-68
ENSDARG00000031465	dgat1a	0.424864644	-1.234924802	5.25E-23	5.29E-22
ENSDARG00000031712	grm3	0.424841291	-1.235004103	2.41E-17	1.79E-16
ENSDARG00000036944	ensaa	0.424476284	-1.236244146	1.47E-16	1.04E-15

ENSDARG00000097492	si:dkey-89b17.7	0.424449888	-1.236333859	5.76E-25	6.34E-24
ENSDARG00000077134	gpr158a	0.424429395	-1.236403519	2.92E-35	4.96E-34
ENSDARG00000075712	CTBP2	0.424281747	-1.236905481	3.17E-15	2.07E-14
ENSDARG00000079858	tmem163a	0.424026826	-1.237772556	7.67E-16	5.19E-15
ENSDARG00000079945	dfnb59	0.423815328	-1.238492328	0.013931495	0.02881109
ENSDARG00000013687	cilp2	0.423766289	-1.238659269	1.50E-49	4.38E-48
ENSDARG00000019990	cabp1a	0.423746013	-1.2387283	1.02E-05	3.19E-05
ENSDARG00000076431	PPP1R9B (1 of 2)	0.423723909	-1.238803557	4.26E-67	2.04E-65
ENSDARG00000059832	bai3	0.423681421	-1.23894823	9.40E-66	4.31E-64
ENSDARG00000086775	top1	0.423629255	-1.239125871	2.68E-08	1.07E-07
ENSDARG00000008249	ptchd4	0.423621775	-1.239151348	5.98E-13	3.37E-12
ENSDARG00000054036	rnf34b	0.423603671	-1.239213003	6.00E-06	1.93E-05
ENSDARG00000053709	CU694453.2	0.423158656	-1.240729418	4.95E-17	3.62E-16
ENSDARG00000043396	fndc4a	0.422933369	-1.241497704	0.002740488	0.006358441
ENSDARG00000020982	PRICKLE2 (1 of 2)	0.422666799	-1.242407304	7.00E-25	7.68E-24
ENSDARG00000001127	slc17a6a	0.422567581	-1.242746005	4.52E-57	1.66E-55
ENSDARG00000060345	apod	0.422378426	-1.243391948	1.82E-52	5.80E-51
ENSDARG00000077228	ntrk3a	0.422267712	-1.243770159	3.07E-11	1.54E-10
ENSDARG00000074211	si:dkeyp-72e1.7	0.422255064	-1.243813369	3.92E-10	1.81E-09
ENSDARG00000062165	TUB	0.422184353	-1.244054984	8.49E-47	2.26E-45
ENSDARG00000090145	TMEM240 (2 of 2)	0.422048079	-1.244520737	1.87E-19	1.56E-18
ENSDARG00000070694	NXPH3	0.422029165	-1.244585393	0.000135681	0.000375887
ENSDARG00000041433	si:dkey-7c18.24	0.421950035	-1.244855923	6.22E-37	1.12E-35
ENSDARG00000092801	si:dkey-71b5.6	0.421879825	-1.245095999	0.019920447	0.039935064
ENSDARG00000062346	cacna1r	0.421783773	-1.245424501	2.18E-35	3.72E-34
ENSDARG00000074443	gas7a	0.421344486	-1.246927849	8.07E-28	1.01E-26
ENSDARG00000075978	pcdh1gc5	0.421329374	-1.246979595	1.49E-36	2.65E-35
ENSDARG00000087745	LRRC38 (2 of 2)	0.421321023	-1.247008188	1.43E-06	4.89E-06
ENSDARG00000079878	si:ch73-387g8.1	0.421318908	-1.247015433	1.53E-09	6.77E-09
ENSDARG00000043746	nrxn3a	0.420880688	-1.24851678	1.23E-69	6.24E-68

ENSDARG00000070045	celf4	0.420880201	-1.248518452	1.60E-64	7.06E-63
ENSDARG00000079698	asb13a.1	0.420838574	-1.248661146	8.39E-12	4.37E-11
ENSDARG00000045765	asb13a.2	0.42078146	-1.248856954	0.002245723	0.005270752
ENSDARG00000097763	si:dkey-286f11.2	0.420760816	-1.248927738	4.04E-15	2.63E-14
ENSDARG00000076691	dlg2	0.420606419	-1.249457228	2.89E-18	2.26E-17
ENSDARG00000086762	DFNA5 (2 of 2)	0.420595066	-1.249496168	6.10E-16	4.15E-15
ENSDARG00000016835	tcirg1	0.420571177	-1.249578115	1.35E-43	3.25E-42
ENSDARG00000086990	LRRC16B	0.420393075	-1.250189189	5.96E-44	1.45E-42
ENSDARG00000089269	FP102887.1	0.420384497	-1.25021863	0.002037857	0.004819111
ENSDARG00000076959	si:ch211-38m6.6	0.420174934	-1.250937995	5.84E-07	2.07E-06
ENSDARG00000033498	rorb	0.42016741	-1.250963829	3.95E-57	1.45E-55
ENSDARG00000054002	hn1a	0.419953	-1.251700221	1.95E-16	1.37E-15
ENSDARG00000063186	BEND7	0.419912276	-1.251840128	1.16E-05	3.61E-05
ENSDARG00000089123	si:dkey-79d12.2	0.419814423	-1.252176362	8.11E-10	3.66E-09
ENSDARG00000079873	si:ch211-160d14.6	0.419690118	-1.252603602	3.32E-16	2.29E-15
ENSDARG00000097477	si:dkey-4c2.11	0.419660405	-1.252705742	0.014578846	0.0300427
ENSDARG00000060459	LRFN2 (1 of 2)	0.419551456	-1.253080333	5.25E-12	2.77E-11
ENSDARG00000012340	ptpn11b	0.41942759	-1.253506329	4.48E-32	6.65E-31
ENSDARG00000062347	mtus2	0.419350351	-1.25377203	6.93E-69	3.43E-67
ENSDARG00000086541	CABZ01100278.1	0.419232392	-1.254177902	1.29E-07	4.84E-07
ENSDARG00000031463	SYT6 (1 of 2)	0.419197741	-1.254297154	2.00E-12	1.09E-11
ENSDARG00000070625	enpp5	0.419081855	-1.254696037	9.47E-42	2.09E-40
ENSDARG00000056450	MANEAL	0.419075628	-1.254717472	2.57E-55	9.05E-54
ENSDARG00000077867	CT573264.3	0.418932914	-1.255208857	2.38E-05	7.18E-05
ENSDARG00000023914	mark4a	0.41884755	-1.255502862	2.14E-39	4.31E-38
ENSDARG00000045789	srgap2b	0.418558173	-1.256499948	3.21E-61	1.30E-59
ENSDARG00000053480	aqp9b	0.418296998	-1.257400452	2.33E-08	9.38E-08
ENSDARG00000070126	si:dkey-22l11.6	0.418093289	-1.258103207	0.000780682	0.001955352
ENSDARG00000088952	CU694197.1	0.417950127	-1.258597296	1.35E-18	1.07E-17
ENSDARG00000079491	KCNA3	0.417916762	-1.258712472	1.33E-33	2.11E-32

ENSDARG00000093604	si:dkey-242k1.6	0.417893222	-1.258793737	4.11E-16	2.82E-15
ENSDARG00000014105	apba1a	0.417854177	-1.258928536	8.94E-28	1.11E-26
ENSDARG00000058771	NAV1 (1 of 3)	0.417497812	-1.26015946	1.19E-35	2.05E-34
ENSDARG00000070100	BX511131.1	0.417432333	-1.260385742	0.009330241	0.019890466
ENSDARG00000018743	scamp5a	0.417349561	-1.260671841	4.28E-59	1.66E-57
ENSDARG00000054026	mustn1a	0.417180931	-1.261254881	0.005356978	0.011896812
ENSDARG00000089412		0.417041143	-1.261738375	1.09E-24	1.18E-23
ENSDARG00000055132	lrfn4a	0.416851458	-1.262394713	9.36E-35	1.55E-33
ENSDARG00000069867		0.416756043	-1.262724975	0.00889665	0.019024649
ENSDARG00000067889	gltpd2	0.416694984	-1.262936363	5.46E-10	2.50E-09
ENSDARG00000079455	nlgn4a	0.416643257	-1.263115462	4.29E-50	1.27E-48
ENSDARG00000089298		0.416609223	-1.263233316	0.000255676	0.000681858
ENSDARG00000023940	si:ch73-362m14.4	0.416575907	-1.263348693	4.41E-39	8.77E-38
ENSDARG00000054253	sobpa	0.416397847	-1.263965487	2.23E-39	4.49E-38
ENSDARG00000087669	UNC13C (2 of 3)	0.416304374	-1.264289378	5.11E-13	2.89E-12
ENSDARG00000088342	tmem145	0.416075784	-1.26508177	2.58E-19	2.15E-18
ENSDARG00000058015	ano3	0.415920126	-1.265621599	2.08E-12	1.12E-11
ENSDARG00000075376	SGSM1 (2 of 2)	0.415584231	-1.266787183	1.16E-48	3.25E-47
ENSDARG00000020212	slc1a1	0.415522441	-1.267001702	1.64E-45	4.18E-44
ENSDARG00000074738	pcdh2g9	0.415348162	-1.267606923	8.43E-05	0.000239976
ENSDARG00000090629	tmtops3b	0.415138049	-1.26833693	0.022077726	0.043845282
ENSDARG00000055317	C1QL4 (2 of 2)	0.414908151	-1.269136095	6.29E-19	5.11E-18
ENSDARG00000073755	pcdh1g22	0.414773037	-1.269605981	8.94E-20	7.62E-19
ENSDARG00000062720	pcdh1a	0.4147405	-1.269719158	1.84E-72	9.99E-71
ENSDARG00000061203	trpc1	0.414519063	-1.270489646	1.24E-34	2.04E-33
ENSDARG00000074866	ptpn5	0.414402319	-1.270896019	3.99E-71	2.10E-69
ENSDARG00000068624	CLMN (2 of 3)	0.414400872	-1.270901056	8.40E-50	2.46E-48
ENSDARG00000057519	CT737184.1	0.414360512	-1.271041573	0.000474278	0.001222948
ENSDARG00000074690	ppm1j	0.414300031	-1.271252168	1.27E-16	9.06E-16
ENSDARG00000061832	SNTG1	0.414239672	-1.271462368	0.002283381	0.005353389

ENSDARG00000068745	MAP4 (1 of 3)	0.414147271	-1.271784211	5.79E-74	3.27E-72
ENSDARG00000042677	cadm1b	0.414126033	-1.271858197	2.59E-13	1.49E-12
ENSDARG00000077367	ntng2a	0.414076136	-1.272032035	3.42E-35	5.79E-34
ENSDARG00000088064	CABZ01064069.1	0.414044659	-1.27214171	0.012113444	0.025355776
ENSDARG00000046014	kcna6	0.413958528	-1.272441854	6.09E-08	2.35E-07
ENSDARG00000033382	grifin	0.413921215	-1.272571901	8.17E-12	4.26E-11
ENSDARG00000079791	si:ch211-176g6.2	0.413864475	-1.272769677	0.003981143	0.009011809
ENSDARG00000095592	si:dkey-11o15.5	0.413742322	-1.273195555	0.000160765	0.000441036
ENSDARG00000022132	drp2	0.413638883	-1.273556287	0.003244214	0.007452328
ENSDARG00000054931	PPP2R5B	0.413474858	-1.274128488	2.65E-57	9.81E-56
ENSDARG00000083588	CABZ01039424.1	0.413379134	-1.274462526	3.59E-05	0.000106455
ENSDARG00000079847	ENTHD1	0.413152724	-1.275252916	5.97E-73	3.30E-71
ENSDARG00000097804	si:ch73-156o22.2	0.413100306	-1.275435966	5.20E-31	7.41E-30
ENSDARG00000074153	slitrk5b	0.412978496	-1.275861433	0.001506738	0.003627123
ENSDARG00000079500	kif3cb	0.412963564	-1.275913598	6.32E-62	2.62E-60
ENSDARG00000054137	gpr126	0.412776342	-1.276567807	7.33E-63	3.12E-61
ENSDARG00000044447	CDHR1 (2 of 2)	0.412763707	-1.27661197	1.53E-17	1.15E-16
ENSDARG00000088584	si:ch73-352p18.4	0.412753751	-1.276646768	0.001021227	0.002517887
ENSDARG00000018773		0.412631369	-1.277074595	1.70E-22	1.67E-21
ENSDARG00000088323		0.412577868	-1.277261661	3.36E-22	3.24E-21
ENSDARG00000090880	CABZ01074423.1	0.412444446	-1.277728286	1.04E-06	3.61E-06
ENSDARG00000053869	ankrd55	0.41232615	-1.278142135	9.53E-28	1.18E-26
ENSDARG00000043122	gpr88	0.412261822	-1.278367229	3.42E-05	0.000101824
ENSDARG00000045014	tuba2	0.412209873	-1.278549033	1.26E-42	2.89E-41
ENSDARG00000025325	CCSAP (1 of 2)	0.412168659	-1.278693288	3.06E-53	1.01E-51
ENSDARG00000075141	gprc5bb	0.412139807	-1.27879428	5.23E-07	1.87E-06
ENSDARG00000070951	hmga1b	0.412125782	-1.278843376	1.52E-75	8.97E-74
ENSDARG00000075159	mfi2	0.412054219	-1.279093913	2.16E-18	1.70E-17
ENSDARG00000078241	MAP7 (2 of 2)	0.412019354	-1.279215988	9.84E-05	0.000277811
ENSDARG00000091683	cnrip1a	0.411933354	-1.27951715	1.24E-71	6.61E-70

ENSDARG00000091043	pcdh2ab6	0.411927339	-1.279538217	7.26E-08	2.78E-07
ENSDARG00000024299	c1qtnf4	0.411840192	-1.279843463	4.07E-65	1.83E-63
ENSDARG00000079264	baiap3	0.411726054	-1.280243349	6.95E-24	7.28E-23
ENSDARG00000008398	cacna1c	0.411528848	-1.280934528	1.15E-46	3.05E-45
ENSDARG00000062725	unc5da	0.411522442	-1.280956987	1.14E-12	6.28E-12
ENSDARG00000077190	HCN1	0.411078231	-1.282515122	1.24E-10	5.96E-10
ENSDARG00000078671	cdk5r2b	0.410992336	-1.282816605	5.16E-12	2.72E-11
ENSDARG00000067964	slc6a5	0.410922799	-1.283060717	2.14E-32	3.24E-31
ENSDARG00000095512	rca2.2	0.410753008	-1.283656954	0.005660245	0.012527227
ENSDARG00000062840	DTX1	0.41072245	-1.283764287	0.000404444	0.001053103
ENSDARG00000078366	robo2	0.410659905	-1.283983999	9.72E-35	1.61E-33
ENSDARG00000016667	gabbr1b	0.410223261	-1.285518794	4.42E-48	1.22E-46
ENSDARG00000027602	mb21d2b	0.410193913	-1.285622013	7.69E-35	1.28E-33
ENSDARG00000070430	chtopb	0.410134365	-1.285831464	6.79E-06	2.17E-05
ENSDARG00000094297	NLRP6 (122 of 145)	0.410114477	-1.285901422	0.001236638	0.003011105
ENSDARG00000079742	MCF2L2	0.410107264	-1.285926798	1.16E-30	1.62E-29
ENSDARG00000091332		0.410078622	-1.286027559	0.016435726	0.033531147
ENSDARG00000090696	CABZ01068272.1	0.41000357	-1.286291623	0.013349946	0.02769914
ENSDARG00000007889	csf1ra	0.409990298	-1.286338326	1.03E-58	3.96E-57
ENSDARG00000059134	CABZ01087549.1	0.409868295	-1.286767698	7.17E-20	6.15E-19
ENSDARG00000044161	grid1b	0.409798903	-1.287011971	7.82E-13	4.36E-12
ENSDARG00000015174	atp6v0a1b	0.409716431	-1.287302344	5.80E-75	3.38E-73
ENSDARG00000087214		0.409453164	-1.288229659	0.000275088	0.000730815
ENSDARG00000089742	FAM161A	0.409300093	-1.288769099	4.49E-12	2.38E-11
ENSDARG00000089438	FP236162.1	0.409248463	-1.288951096	9.58E-25	1.04E-23
ENSDARG00000019341	gpc1a	0.409226316	-1.289029173	2.09E-54	7.15E-53
ENSDARG00000090170	rab11fip4a	0.40917896	-1.289196132	0.007313362	0.01587066
ENSDARG00000061454	nrxn2a	0.409098962	-1.289478217	5.40E-76	3.26E-74
ENSDARG00000034757	zdhhc2	0.409089123	-1.289512916	6.04E-13	3.39E-12
ENSDARG00000089314	mapta	0.409070489	-1.289578633	3.33E-40	6.89E-39

ENSDARG00000070495	FRMD7 (2 of 2)	0.409032194	-1.289713696	2.81E-05	8.41E-05
ENSDARG00000058875	pcdh2ac	0.40892266	-1.290100085	2.22E-76	1.35E-74
ENSDARG00000045760	prmt8b	0.408880094	-1.290250268	3.36E-17	2.48E-16
ENSDARG00000056745	necab2	0.408811056	-1.29049388	2.57E-71	1.36E-69
ENSDARG00000074821	NLRP6 (12 of 145)	0.408592642	-1.291264869	0.017990458	0.036373934
ENSDARG00000034215	rab42a	0.40858114	-1.291305484	1.06E-41	2.33E-40
ENSDARG00000052905	zgc:165423	0.408558449	-1.291385609	1.15E-16	8.24E-16
ENSDARG00000076376	tmem175	0.408549155	-1.291418428	9.64E-17	6.94E-16
ENSDARG00000054836	slc1a9	0.408052236	-1.293174248	0.0002352	0.000630091
ENSDARG00000079700	si:dkey-276l13.6	0.408012707	-1.293314011	3.37E-13	1.93E-12
ENSDARG00000060330	si:ch211-284e13.4	0.407952789	-1.293525892	2.18E-37	4.01E-36
ENSDARG00000062687	KCNH7 (1 of 2)	0.407794785	-1.294084767	2.79E-39	5.59E-38
ENSDARG00000030265	scdb	0.407727376	-1.294323267	1.90E-58	7.23E-57
ENSDARG00000015530	si:dkey-169l5.3	0.407605477	-1.294754659	7.94E-24	8.30E-23
ENSDARG00000078141	ELFN1 (2 of 2)	0.407567741	-1.294888228	1.01E-24	1.10E-23
ENSDARG00000060237	kcnma1b	0.407517174	-1.295067234	0.000329679	0.000867973
ENSDARG00000033140	desi1a	0.407484706	-1.295182182	5.38E-64	2.34E-62
ENSDARG00000094577	CDC42SE2	0.407474393	-1.295218697	4.76E-36	8.33E-35
ENSDARG00000043864	nptnb	0.4074301	-1.295375528	4.98E-59	1.93E-57
ENSDARG00000003998	phyhipla	0.407342548	-1.295685581	1.93E-22	1.89E-21
ENSDARG00000045156	rgs9b	0.407130192	-1.29643788	5.68E-26	6.56E-25
ENSDARG00000041033	or119-2	0.406792083	-1.297636493	0.002689226	0.006245115
ENSDARG00000059751	BX511065.1	0.406767446	-1.29772387	1.83E-24	1.97E-23
ENSDARG00000060269	WSCD1 (1 of 3)	0.406748175	-1.297792222	5.88E-06	1.89E-05
ENSDARG00000079412	ftr02	0.406674083	-1.298055041	3.57E-10	1.65E-09
ENSDARG00000079986	si:ch211-250k18.8	0.406279708	-1.299454787	0.008082986	0.017409177
ENSDARG00000055644	zgc:101788	0.406198423	-1.299743455	6.13E-11	3.00E-10
ENSDARG00000068716	cuedc1a	0.406094651	-1.30011207	1.26E-46	3.32E-45
ENSDARG00000075858	fam155a	0.406018029	-1.300384302	1.41E-08	5.79E-08
ENSDARG00000091130	KCNQ2 (2 of 2)	0.405723279	-1.301432015	7.05E-36	1.23E-34

ENSDARG00000044387	cldn2	0.405710815	-1.301476336	2.44E-08	9.77E-08
ENSDARG00000034522	rab6ba	0.405524701	-1.302138303	1.57E-64	6.95E-63
ENSDARG00000017429	sphkap	0.40532867	-1.30283587	3.94E-50	1.17E-48
ENSDARG00000043448	itm2ca	0.405309482	-1.302904169	1.38E-77	8.60E-76
ENSDARG00000051712	si:ch211-147g22.2	0.405267749	-1.303052725	0.000192086	0.000520777
ENSDARG00000003991	fhl2b	0.405056033	-1.3038066	1.20E-29	1.62E-28
ENSDARG00000032039	mxd1	0.405038172	-1.303870218	2.40E-37	4.41E-36
ENSDARG00000085981	5S_rRNA	0.404726378	-1.304981216	5.90E-05	0.000170776
ENSDARG00000079119	si:ch211-229d2.5	0.40442735	-1.306047529	9.88E-43	2.29E-41
ENSDARG00000007151	kcnk2b	0.404399433	-1.306147122	1.06E-12	5.88E-12
ENSDARG00000078744	tmem63bb	0.404273384	-1.306596869	1.82E-12	9.88E-12
ENSDARG00000060400	si:ch73-362m14.3	0.404052989	-1.307383588	3.53E-05	0.000104868
ENSDARG00000068323	astn1	0.403988284	-1.30761464	1.04E-75	6.21E-74
ENSDARG00000045275	klhl14	0.40392551	-1.307838831	4.15E-40	8.56E-39
ENSDARG00000033544	adamts15b	0.403818211	-1.308222122	4.52E-33	6.99E-32
ENSDARG00000088023	si:dkey-26c10.5	0.403735772	-1.308516675	1.44E-08	5.89E-08
ENSDARG00000038367	cyp2k19	0.403674328	-1.308736256	4.85E-10	2.23E-09
ENSDARG00000091912	NLRP6 (97 of 145)	0.403636082	-1.308872948	0.013403232	0.02780361
ENSDARG00000014746	rbfox1	0.403605316	-1.308982916	7.11E-67	3.38E-65
ENSDARG00000076351	brinp3a	0.403569414	-1.309111258	5.31E-44	1.30E-42
ENSDARG00000031600	rd3	0.403541043	-1.309212682	3.75E-44	9.21E-43
ENSDARG00000086231	PCDHGC5 (7 of 31)	0.403523022	-1.30927711	0.000277559	0.000736762
ENSDARG00000069171	gramd4	0.403418505	-1.309650831	5.02E-08	1.95E-07
ENSDARG00000015240	cdkl5	0.403363013	-1.309849296	1.89E-45	4.83E-44
ENSDARG00000068138	ch25hl1.2	0.403104513	-1.310774161	0.002831406	0.006556539
ENSDARG00000078247	vip	0.40309166	-1.31082016	2.36E-09	1.02E-08
ENSDARG00000029989	adcyap1r1a	0.402945841	-1.311342154	2.53E-78	1.61E-76
ENSDARG00000012482	pclob	0.402913455	-1.31145811	1.10E-66	5.21E-65
ENSDARG00000079665	GPR158 (2 of 2)	0.402912812	-1.311460413	1.03E-20	9.24E-20
ENSDARG00000056244		0.402881368	-1.311573007	1.14E-29	1.54E-28

ENSDARG00000053220		0.402865047	-1.311631453	4.11E-55	1.44E-53
ENSDARG00000030078	glipr1b	0.402559406	-1.312726395	5.24E-21	4.77E-20
ENSDARG00000089130	CABZ01084618.1	0.402236416	-1.313884396	6.16E-11	3.02E-10
ENSDARG00000088593	chst1	0.402046869	-1.314564402	8.97E-50	2.62E-48
ENSDARG00000088735	RAP1GAP (3 of 3)	0.401928626	-1.314988765	0.002934251	0.006779773
ENSDARG00000090642	si:dkey-54n8.4	0.401790972	-1.315482948	9.92E-29	1.28E-27
ENSDARG00000061718	znf704	0.40177558	-1.315538216	2.16E-21	2.00E-20
ENSDARG00000090064	CABZ01084273.1	0.401715246	-1.31575488	4.00E-22	3.83E-21
ENSDARG00000062293	qpctlb	0.40120815	-1.317577181	0.007795291	0.016835482
ENSDARG00000068245	asic1c	0.400868702	-1.318798313	3.29E-07	1.20E-06
ENSDARG00000045145	shisa9a	0.400755042	-1.319207423	4.22E-16	2.90E-15
ENSDARG00000074320	KCNAB3	0.400601336	-1.319760861	4.21E-07	1.52E-06
ENSDARG00000071048	wisp3	0.400560523	-1.319907851	1.14E-26	1.36E-25
ENSDARG00000033508	CR396586.1	0.400475789	-1.32021307	0.002199105	0.005169025
ENSDARG00000004861	esrrga	0.400296921	-1.320857576	5.18E-79	3.35E-77
ENSDARG00000028628	gdap1l1	0.399986892	-1.321975374	1.69E-18	1.34E-17
ENSDARG00000074563	GPR37 (2 of 2)	0.399892121	-1.32231724	7.88E-26	9.02E-25
ENSDARG00000042940	nab1a	0.399830433	-1.322539809	2.67E-17	1.98E-16
ENSDARG00000095915	col6a3	0.399681365	-1.323077785	3.89E-73	2.17E-71
ENSDARG00000025847	sox12	0.399628951	-1.323266992	2.59E-79	1.70E-77
ENSDARG00000040982	fgf14	0.399624926	-1.323281524	2.82E-25	3.14E-24
ENSDARG00000091094	CABZ01111604.1	0.399605488	-1.3233517	6.33E-11	3.10E-10
ENSDARG00000052242	aff2	0.399469979	-1.323841008	2.60E-62	1.09E-60
ENSDARG00000053771	APBB3	0.399404449	-1.324077693	1.73E-26	2.04E-25
ENSDARG00000062024	kif1ab	0.399397931	-1.324101236	3.13E-79	2.05E-77
ENSDARG00000006923	cacna1ab	0.399397749	-1.324101891	4.35E-41	9.32E-40
ENSDARG00000087981	CABZ01088330.1	0.398811168	-1.326222285	4.12E-71	2.17E-69
ENSDARG00000063056	tulp4b	0.398644448	-1.326825518	8.62E-57	3.13E-55
ENSDARG00000062236	ndrg4	0.398318484	-1.328005666	2.83E-49	8.17E-48
ENSDARG00000042794	CABZ01079045.1	0.398202793	-1.328424753	6.41E-08	2.47E-07

ENSDARG00000035899	lingo1b	0.398161971	-1.328572661	1.21E-45	3.10E-44
ENSDARG00000079161	BSN (1 of 3)	0.398099381	-1.328799467	1.60E-79	1.06E-77
ENSDARG00000055792	FOXO4	0.398063931	-1.328927943	5.76E-73	3.20E-71
ENSDARG00000036383	si:ch73-335m24.5	0.397637497	-1.330474287	3.31E-32	4.95E-31
ENSDARG00000020718	SLC25A22 (1 of 2)	0.397599607	-1.330611764	4.42E-39	8.78E-38
ENSDARG00000075924	RAPGEF4	0.39739174	-1.331366209	2.35E-34	3.84E-33
ENSDARG00000074073	MAP6 (1 of 2)	0.397090041	-1.332461918	1.01E-44	2.53E-43
ENSDARG00000097648	C15H11orf87	0.396987949	-1.332832881	3.99E-66	1.86E-64
ENSDARG00000009081	sh2d3cb	0.39677978	-1.333589586	6.14E-08	2.37E-07
ENSDARG00000055672	map3k10	0.396740771	-1.333731432	3.22E-30	4.43E-29
ENSDARG00000004141	zgc:92630	0.396633202	-1.334122646	1.08E-15	7.28E-15
ENSDARG00000023600	sh3gl2	0.396255535	-1.335497008	1.57E-73	8.80E-72
ENSDARG00000078536	MEGF9 (2 of 2)	0.396111754	-1.336020583	5.72E-30	7.81E-29
ENSDARG00000032959	kcnc1b	0.395980749	-1.336497802	2.01E-35	3.45E-34
ENSDARG00000033556	PCDH11Y	0.395855915	-1.336952687	7.82E-76	4.69E-74
ENSDARG00000056163	zgc:73228	0.395791375	-1.337187923	1.85E-16	1.30E-15
ENSDARG00000089771	KCTD5	0.39567596	-1.337608681	2.89E-26	3.38E-25
ENSDARG00000052558	porcn	0.395615449	-1.33782933	8.33E-50	2.44E-48
ENSDARG00000036446	PRKAR2B	0.395365129	-1.338742462	1.26E-43	3.02E-42
ENSDARG00000059960	plch2a	0.395347733	-1.338805945	4.92E-77	3.02E-75
ENSDARG00000068083	RAB37	0.395229868	-1.339236117	1.73E-05	5.29E-05
ENSDARG00000002898	DGKA (1 of 2)	0.395178867	-1.339422296	9.67E-08	3.67E-07
ENSDARG00000088739	si:ch211-235m3.9	0.39509813	-1.339717076	0.008154632	0.017550186
ENSDARG00000086790	gucy1b3	0.395062799	-1.339846093	1.52E-38	2.95E-37
ENSDARG00000076697	SIPA1	0.394973999	-1.340170411	2.00E-57	7.41E-56
ENSDARG00000089193	si:ch211-287i21.1	0.394929591	-1.340332626	1.62E-32	2.45E-31
ENSDARG00000090760	FAM160A1 (2 of 2)	0.394913319	-1.340392071	1.33E-25	1.50E-24
ENSDARG00000073883	clstn3	0.394897015	-1.340451634	1.76E-76	1.07E-74
ENSDARG00000077428	camta1a	0.394804769	-1.340788679	4.49E-78	2.84E-76
ENSDARG00000006383	stxbp5l	0.394704016	-1.341156897	9.74E-45	2.44E-43

ENSDARG00000076790	crygm2d16	0.394646949	-1.3413655	3.04E-81	2.08E-79
ENSDARG00000036952	XKR7 (2 of 2)	0.394642078	-1.341383305	8.84E-23	8.79E-22
ENSDARG00000037390	gsg1l	0.394573165	-1.341635253	0.00010142	0.000285838
ENSDARG00000037498	gria3b	0.394477539	-1.341984938	4.05E-46	1.05E-44
ENSDARG00000032199	gpc3	0.394462096	-1.342041418	2.07E-07	7.69E-07
ENSDARG00000045639	elavl4	0.394442728	-1.342112255	1.91E-57	7.08E-56
ENSDARG00000043553	ches1	0.394435714	-1.342137907	3.36E-17	2.47E-16
ENSDARG00000073920	cntnap5a	0.394002782	-1.34372228	1.27E-32	1.93E-31
ENSDARG00000010433	ARHGAP44 (1 of 2)	0.39399702	-1.343743377	1.54E-18	1.22E-17
ENSDARG00000084627	5S_rRNA	0.393734661	-1.344704375	0.008568405	0.018373893
ENSDARG00000052910	wnt8a	0.393302645	-1.346288205	0.019031046	0.038284417
ENSDARG00000073699	COL9A1 (2 of 2)	0.393249944	-1.346481535	8.42E-41	1.78E-39
ENSDARG00000006747	tmem178b	0.392962624	-1.347535995	7.82E-17	5.66E-16
ENSDARG00000079671	lppr4a	0.392939529	-1.347620788	1.13E-33	1.80E-32
ENSDARG00000075154	B3GALT1	0.39281062	-1.348094161	1.15E-08	4.74E-08
ENSDARG00000093411	gad1a	0.392709249	-1.348466519	4.19E-16	2.88E-15
ENSDARG00000057155	XRG5	0.392676913	-1.348585316	4.68E-09	1.99E-08
ENSDARG00000086716	NXPH4	0.392652926	-1.348673447	0.000360973	0.000946417
ENSDARG00000076640	NFATC2 (1 of 2)	0.392547127	-1.349062229	2.27E-09	9.85E-09
ENSDARG00000041627	sncb	0.392512632	-1.34918901	1.76E-51	5.45E-50
ENSDARG00000058222	mpp4a	0.392289531	-1.350009259	1.01E-09	4.52E-09
ENSDARG00000075133	BAI1 (1 of 3)	0.392196033	-1.350353154	7.09E-30	9.63E-29
ENSDARG00000031537	wu:fl09a04	0.391988745	-1.351115864	8.50E-10	3.84E-09
ENSDARG00000090930	si:ch211-120g10.1	0.391899877	-1.351442975	1.14E-11	5.91E-11
ENSDARG00000093359	inpp5jb	0.391736583	-1.352044232	3.19E-12	1.71E-11
ENSDARG00000089883	si:dkey-210j14.3	0.391611347	-1.352505528	7.58E-17	5.49E-16
ENSDARG00000060939	KIRREL3 (1 of 3)	0.391595946	-1.352562267	4.44E-20	3.84E-19
ENSDARG00000012388	CU855779.1	0.391519948	-1.35284228	1.34E-26	1.59E-25
ENSDARG00000093089	tac3a	0.391102077	-1.354382896	0.000177325	0.0004832
ENSDARG00000087010	ATG2A	0.390955743	-1.354922794	5.15E-08	2.00E-07

ENSDARG00000089486	cplx3b	0.390955513	-1.354923644	3.57E-11	1.78E-10
ENSDARG00000009941	erc1a	0.39075687	-1.355656859	4.88E-53	1.59E-51
ENSDARG00000074724		0.390607303	-1.356209173	7.56E-05	0.000216533
ENSDARG00000030791	mafgb	0.390574031	-1.356332067	2.01E-32	3.04E-31
ENSDARG00000027360	si:ch211-106h11.3	0.390516567	-1.35654434	1.62E-52	5.19E-51
ENSDARG00000008186	syk	0.390450002	-1.356790277	3.55E-23	3.61E-22
ENSDARG00000069994	elna	0.390289529	-1.357383337	5.23E-30	7.15E-29
ENSDARG00000077888	slain1b	0.390252162	-1.357521469	6.33E-10	2.88E-09
ENSDARG00000077652	LRAT	0.390235593	-1.357582723	5.20E-06	1.68E-05
ENSDARG00000075694	adora1	0.390222222	-1.357632159	3.75E-15	2.44E-14
ENSDARG00000089082	CU041398.1	0.389881716	-1.358891596	2.02E-39	4.08E-38
ENSDARG00000056047	noxa1	0.389822666	-1.359110118	0.0002015	0.000544695
ENSDARG00000096261	CELF6	0.389817684	-1.359128557	0.006993715	0.015240161
ENSDARG00000076309	mxra5b	0.389692109	-1.359593375	1.99E-55	7.05E-54
ENSDARG00000075043	GPRIN3	0.389103045	-1.361775824	1.41E-54	4.86E-53
ENSDARG00000014053	olfm1b	0.389027596	-1.362055598	4.86E-84	3.52E-82
ENSDARG00000093091	iqsec3b	0.388986763	-1.362207033	1.79E-08	7.24E-08
ENSDARG00000090963	ATP6AP1L (1 of 2)	0.388774992	-1.362992675	7.55E-44	1.84E-42
ENSDARG00000013491	map3k9	0.388750655	-1.363082989	3.40E-29	4.49E-28
ENSDARG00000097374	si:dkey-108k21.7	0.388672191	-1.363374208	6.61E-07	2.34E-06
ENSDARG00000096508	si:ch211-265g21.1	0.388411562	-1.364341949	1.01E-42	2.34E-41
ENSDARG00000057408	CABZ01067232.1	0.388376965	-1.364470457	3.42E-29	4.51E-28
ENSDARG00000026137	sfxn5b	0.388171007	-1.365235731	1.27E-65	5.77E-64
ENSDARG00000075281	tbc1d30	0.388157768	-1.365284935	6.94E-62	2.88E-60
ENSDARG00000071892	CU915772.1	0.388157588	-1.365285603	4.05E-05	0.00011931
ENSDARG00000058357	ankrd33ba	0.388095695	-1.365515664	6.21E-13	3.49E-12
ENSDARG00000061497	THSD7B (1 of 2)	0.388017341	-1.365806965	1.25E-09	5.56E-09
ENSDARG00000062420	nfia	0.387715183	-1.366930863	7.16E-85	5.26E-83
ENSDARG00000005453	foxp2	0.387618358	-1.367291197	7.39E-83	5.24E-81
ENSDARG00000097226	pde4ba	0.3875886	-1.367401955	0.001475557	0.003555677

ENSDARG00000086049	FAM196B	0.387563618	-1.367494947	0.005231777	0.011635135
ENSDARG00000068421	ttc9b	0.387530499	-1.367618239	6.18E-31	8.79E-30
ENSDARG00000091509	ATP6AP1L (2 of 2)	0.387368949	-1.368219779	4.21E-20	3.65E-19
ENSDARG00000022971	epha6	0.386971894	-1.36969931	5.28E-36	9.24E-35
ENSDARG00000062849	kcnk10a	0.386623693	-1.370998042	1.01E-09	4.52E-09
ENSDARG00000071889	pcdh2g8	0.386556251	-1.371249726	2.67E-05	8.03E-05
ENSDARG00000057056	si:dkey-202l22.3	0.386410719	-1.371792981	0.00092513	0.002292293
ENSDARG00000013615	pbx3b	0.386360829	-1.371979259	8.77E-82	6.08E-80
ENSDARG00000069139	grik1a	0.386324424	-1.372115207	2.75E-14	1.70E-13
ENSDARG00000055852	MTNR1A (2 of 2)	0.386125954	-1.372856566	0.00527226	0.011717828
ENSDARG00000032799	kcnd2	0.386108118	-1.372923208	2.63E-33	4.11E-32
ENSDARG00000062122	klhl5	0.386046387	-1.373153883	1.08E-15	7.24E-15
ENSDARG00000035655	rad21b	0.385965287	-1.373456996	7.44E-62	3.07E-60
ENSDARG00000037682	BX005294.1	0.385946142	-1.373528557	2.69E-10	1.26E-09
ENSDARG00000021241	zgc:165604	0.385893084	-1.373726906	3.14E-07	1.14E-06
ENSDARG00000077189	INTS5 (1 of 2)	0.385600025	-1.374822949	9.93E-07	3.45E-06
ENSDARG00000040535	CSGALNACT1 (1 of 2)	0.385578281	-1.374904305	1.11E-55	3.95E-54
ENSDARG00000054288	zgc:63972	0.385391975	-1.375601565	9.15E-13	5.08E-12
ENSDARG00000069767	pvrl1a	0.385219917	-1.376245797	8.47E-12	4.41E-11
ENSDARG00000031640	si:dkey-109j17.5	0.385160829	-1.376467108	1.98E-08	8.00E-08
ENSDARG00000052775	opn3	0.385081221	-1.376765326	1.06E-05	3.33E-05
ENSDARG00000037660	TRPC7 (2 of 3)	0.385010248	-1.377031246	8.34E-05	0.000237551
ENSDARG00000012367	TRIM46 (1 of 2)	0.3849089	-1.377411064	5.89E-43	1.38E-41
ENSDARG00000094512		0.38483374	-1.377692803	3.64E-07	1.32E-06
ENSDARG00000092174	si:ch211-59d15.4	0.384771532	-1.377926033	0.002463036	0.005748501
ENSDARG00000022518	pex5la	0.384607104	-1.378542686	4.13E-11	2.05E-10
ENSDARG00000012667	tfap2b	0.384593618	-1.378593271	2.02E-84	1.47E-82
ENSDARG00000070810	cck	0.384183773	-1.380131512	4.33E-07	1.56E-06
ENSDARG00000079899	FAM135B	0.384050597	-1.380631703	4.41E-13	2.50E-12
ENSDARG00000055463	lrit3a	0.383953722	-1.38099566	7.33E-05	0.000210066

ENSDARG00000022817	pvalb3	0.383889258	-1.381237902	1.82E-53	6.02E-52
ENSDARG00000094256	si:dkey-46m10.2	0.383818863	-1.381502478	0.001608307	0.003857248
ENSDARG00000040246	rap2ab	0.383271094	-1.383562899	1.60E-10	7.60E-10
ENSDARG00000062477	kiaa1549la	0.383159916	-1.383981452	1.02E-78	6.53E-77
ENSDARG00000045353		0.382993349	-1.384608757	6.64E-06	2.12E-05
ENSDARG00000089297	CR847851.8	0.382975875	-1.384674578	1.70E-06	5.77E-06
ENSDARG00000086485	PCDHGC5 (9 of 31)	0.382416066	-1.38678496	0.000255362	0.000681084
ENSDARG00000086451	CR318624.1	0.382302109	-1.387214935	7.63E-05	0.000218241
ENSDARG00000040625	slc8a2b	0.382266338	-1.387349933	3.21E-23	3.28E-22
ENSDARG00000039951	si:ch211-87j1.4	0.381832262	-1.388989089	2.47E-10	1.16E-09
ENSDARG00000076809	si:ch211-141h20.6	0.381488454	-1.3902887	0.001610964	0.003863294
ENSDARG00000086731	FSTL4 (2 of 2)	0.381267043	-1.391126266	0.000589079	0.001499238
ENSDARG00000033655	stmn1b	0.381024874	-1.392042914	3.44E-39	6.86E-38
ENSDARG00000088020	cdkn1d	0.380970522	-1.392248721	6.55E-65	2.93E-63
ENSDARG00000029114	LMTK3	0.380849443	-1.392707308	8.85E-78	5.56E-76
ENSDARG00000059781	atcayb	0.380693574	-1.393297876	1.69E-05	5.19E-05
ENSDARG00000078088	CR339051.1	0.380356165	-1.394577106	8.19E-33	1.26E-31
ENSDARG00000070005	GABRA2 (2 of 2)	0.38022896	-1.395059677	0.000710169	0.001788487
ENSDARG00000079656	FAM171A2 (2 of 2)	0.380074503	-1.395645849	4.32E-88	3.37E-86
ENSDARG00000075539	snphb	0.379919842	-1.396233034	2.60E-15	1.70E-14
ENSDARG00000087088	si:ch211-274p24.4	0.379835429	-1.396553617	9.02E-15	5.72E-14
ENSDARG00000079741	CSRNP3	0.379801724	-1.396681643	4.26E-33	6.61E-32
ENSDARG00000076820	xkr8.2	0.379766801	-1.396814303	1.62E-11	8.29E-11
ENSDARG00000009965	mag	0.379397598	-1.398217551	2.60E-25	2.90E-24
ENSDARG00000059682	slc43a3a	0.379092544	-1.399378014	1.02E-20	9.19E-20
ENSDARG00000074781	ptprt	0.379020091	-1.399653771	1.08E-65	4.95E-64
ENSDARG00000068787	slc6a17	0.378788858	-1.4005342	8.43E-38	1.58E-36
ENSDARG00000036058	gnao1b	0.378709294	-1.400837267	3.22E-88	2.52E-86
ENSDARG00000077343	CT573264.2	0.378604133	-1.401237934	0.000288124	0.000763164
ENSDARG00000061082	CR936337.1	0.378457954	-1.401795068	2.05E-21	1.91E-20

ENSDARG00000034493	grin2aa	0.378339639	-1.402246158	1.19E-54	4.13E-53
ENSDARG00000010031	ssh1b	0.378246094	-1.402602909	1.04E-43	2.52E-42
ENSDARG00000078258	GGT5 (2 of 2)	0.37811735	-1.403094045	9.85E-08	3.74E-07
ENSDARG00000003250	pik3cd	0.378065562	-1.403291655	9.48E-15	6.01E-14
ENSDARG00000003290	dab1b	0.377856989	-1.404087786	4.07E-13	2.32E-12
ENSDARG00000074524	CNTNAP1	0.37782267	-1.404218826	1.09E-21	1.03E-20
ENSDARG00000042608	CABZ01078431.1	0.377821397	-1.404223689	9.61E-19	7.72E-18
ENSDARG00000033473		0.377726283	-1.404586921	0.011768562	0.024692081
ENSDARG00000061885	satb2	0.377719093	-1.404614382	2.40E-39	4.82E-38
ENSDARG00000076701	B4GALNT4 (2 of 2)	0.377717933	-1.404618815	6.79E-19	5.51E-18
ENSDARG00000056690	mtmr1a	0.377595588	-1.405086187	3.36E-67	1.61E-65
ENSDARG00000088522	CR847851.5	0.377564137	-1.40520636	0.000129451	0.000359681
ENSDARG00000092082	si:ch1073-44m10.1	0.377250638	-1.406404756	9.45E-09	3.92E-08
ENSDARG00000087127	TCERG1L	0.377194572	-1.40661918	2.11E-09	9.20E-09
ENSDARG00000060877	edil3b	0.377166698	-1.406725796	4.02E-08	1.58E-07
ENSDARG00000043662	cnih2	0.377139397	-1.406830228	2.68E-54	9.13E-53
ENSDARG00000031049	igsf21a	0.37689499	-1.407765476	2.46E-37	4.51E-36
ENSDARG00000091605	U6	0.376724055	-1.40841994	0.020078353	0.040219198
ENSDARG00000029374	dennd1b	0.37640231	-1.409652614	8.07E-13	4.49E-12
ENSDARG00000068232	cbln12	0.376398539	-1.409667065	0.000774578	0.001940748
ENSDARG00000044319	fstl4	0.376353374	-1.409840191	1.21E-11	6.22E-11
ENSDARG00000022487	DYNC1I1 (1 of 2)	0.37629652	-1.410058146	1.73E-19	1.45E-18
ENSDARG00000022109	kcnip1a	0.376257097	-1.410209301	8.95E-45	2.24E-43
ENSDARG00000021595	lhfpl3	0.37624538	-1.410254228	3.42E-76	2.07E-74
ENSDARG00000079308	tor3a	0.37559375	-1.412755038	9.46E-44	2.29E-42
ENSDARG00000060354	samd7	0.375383114	-1.413564339	1.09E-37	2.03E-36
ENSDARG00000086973	GPRIN1	0.375355608	-1.413670059	1.99E-08	8.02E-08
ENSDARG00000054575	waif2	0.375337444	-1.413739875	1.47E-05	4.53E-05
ENSDARG00000043904	pnrc1	0.375228712	-1.41415787	1.66E-12	9.04E-12
ENSDARG00000059773	SRCIN1 (2 of 2)	0.375046177	-1.41485986	1.89E-72	1.03E-70

ENSDARG00000096449	NLRP6 (140 of 145)	0.374837168	-1.415664082	0.007595362	0.016432424
ENSDARG00000078528		0.374743009	-1.416026532	2.76E-86	2.09E-84
ENSDARG00000088117	FUT8 (2 of 2)	0.374739129	-1.416041468	8.84E-13	4.91E-12
ENSDARG00000076712	RPH3A (2 of 2)	0.374351051	-1.417536289	1.83E-34	2.99E-33
ENSDARG00000062887	steap2	0.374013406	-1.418838114	7.33E-29	9.53E-28
ENSDARG00000033832	ampd1	0.37391631	-1.419212694	1.23E-46	3.25E-45
ENSDARG00000074601	cbln2a	0.373802449	-1.419652074	0.000136784	0.000378831
ENSDARG00000068477	csdc2b	0.373748969	-1.419858494	9.37E-09	3.89E-08
ENSDARG00000052437	MIA	0.373454983	-1.420993745	2.75E-35	4.69E-34
ENSDARG00000057575	pnp4a	0.373330271	-1.421475601	1.03E-90	8.53E-89
ENSDARG00000095192	si:dkey-150k17.2	0.3732613	-1.421742159	8.93E-30	1.21E-28
ENSDARG00000005853	slc25a6	0.372861421	-1.423288564	4.41E-33	6.83E-32
ENSDARG00000045230	cox6b1	0.372823094	-1.423436867	2.70E-90	2.21E-88
ENSDARG00000097781	si:ch73-374e19.2	0.372773731	-1.423627896	1.18E-11	6.11E-11
ENSDARG00000062621	SATB1	0.372542357	-1.424523629	1.43E-68	7.04E-67
ENSDARG00000080021	BX548160.2	0.372466579	-1.424817114	1.54E-06	5.24E-06
ENSDARG00000043852	CABZ01092941.1	0.372321539	-1.425379017	1.23E-10	5.91E-10
ENSDARG00000033447	nxph1	0.372264471	-1.425600165	1.50E-71	8.02E-70
ENSDARG00000030547	rnd1	0.372006496	-1.426600282	3.12E-74	1.77E-72
ENSDARG00000034373	col27a1a	0.371981142	-1.42669861	2.92E-91	2.50E-89
ENSDARG00000086255	si:rp71-1c10.10	0.371338961	-1.429191402	0.007848659	0.016943013
ENSDARG00000078327	CEP170B (2 of 3)	0.371197377	-1.429741579	1.30E-65	5.91E-64
ENSDARG00000075209	P4HTM	0.371189698	-1.429771424	9.14E-26	1.04E-24
ENSDARG00000013005	opcml	0.371119603	-1.430043888	4.33E-16	2.97E-15
ENSDARG00000078832	si:dkey-73p2.4	0.370944906	-1.430723166	1.33E-05	4.12E-05
ENSDARG00000068701	gpr85	0.370890393	-1.430935196	1.33E-82	9.40E-81
ENSDARG00000017162	CABZ01076801.1	0.370766192	-1.431418397	1.18E-58	4.53E-57
ENSDARG00000018436	CABZ01096573.1	0.370682057	-1.431745813	1.39E-27	1.72E-26
ENSDARG00000079347	zgc:194659	0.370638485	-1.431915406	1.13E-14	7.13E-14
ENSDARG00000089986	TP53INP2	0.370561944	-1.432213368	1.78E-78	1.13E-76

ENSDARG00000061990	kcnh4	0.370555562	-1.432238217	3.83E-08	1.51E-07
ENSDARG00000070173	gria2a	0.37025483	-1.433409538	3.07E-91	2.62E-89
ENSDARG00000092466	thbs2a	0.370130442	-1.433894297	2.04E-15	1.35E-14
ENSDARG00000020544	rem1	0.370078839	-1.43409545	7.80E-30	1.06E-28
ENSDARG00000044091	pitpnab	0.370049301	-1.434210604	9.73E-67	4.61E-65
ENSDARG00000063007	apc2	0.36946223	-1.43650121	1.00E-77	6.28E-76
ENSDARG00000014273	camk2d2	0.369429723	-1.436628151	2.94E-93	2.63E-91
ENSDARG00000092151	si:dkey-40i22.5	0.36927752	-1.437222654	0.001104973	0.002709553
ENSDARG00000089593	pcdh2ab11	0.369248959	-1.43733424	6.50E-11	3.18E-10
ENSDARG00000097012	si:dkey-225f23.5	0.36915941	-1.437684161	6.76E-25	7.42E-24
ENSDARG00000056087	C9H2orf40 (1 of 2)	0.368979512	-1.438387382	9.58E-25	1.04E-23
ENSDARG00000059939	dab1a	0.368910428	-1.438657526	2.57E-44	6.33E-43
ENSDARG00000079933	SLC46A3 (2 of 2)	0.368749602	-1.439286602	3.09E-48	8.60E-47
ENSDARG00000009738	cacnb4a	0.368645129	-1.4396954	3.54E-14	2.16E-13
ENSDARG00000090744	CABZ01089777.1	0.368592654	-1.439900778	0.001062899	0.002614278
ENSDARG00000078650	KCNA4	0.368541712	-1.440100182	1.81E-07	6.74E-07
ENSDARG00000096238	si:dkey-156n14.10	0.368527135	-1.440157243	0.00011129	0.000311935
ENSDARG00000059173	gdf2	0.368451623	-1.440452887	0.00787685	0.016998358
ENSDARG00000087438	si:dkey-257i7.5	0.368307278	-1.441018188	0.012598188	0.026289096
ENSDARG00000076777	BX119910.5	0.36815175	-1.441627536	2.00E-14	1.24E-13
ENSDARG00000007398	LRRK2 (1 of 2)	0.368079797	-1.441909529	2.10E-16	1.47E-15
ENSDARG00000061042	gabbr2	0.367911006	-1.442571258	4.12E-25	4.57E-24
ENSDARG00000070473	parp6b	0.367865677	-1.442749019	2.81E-39	5.63E-38
ENSDARG00000039434	oprm1	0.367662425	-1.443546352	1.04E-06	3.60E-06
ENSDARG00000059311	dpysl5b	0.367535779	-1.444043393	4.28E-91	3.61E-89
ENSDARG00000061117	agap2	0.367516313	-1.444119807	2.05E-65	9.28E-64
ENSDARG00000023373	lppr5b	0.367409812	-1.444537939	3.08E-06	1.02E-05
ENSDARG00000020489	CASKIN1 (1 of 2)	0.367183035	-1.445428691	1.17E-62	4.95E-61
ENSDARG00000086307	PCDHGC5 (8 of 31)	0.367080772	-1.44583055	6.68E-05	0.000192154
ENSDARG00000022509	cox4i2	0.367041013	-1.445986817	6.04E-55	2.11E-53

ENSDARG00000017466	ncam2	0.367038959	-1.445994892	1.64E-73	9.16E-72
ENSDARG00000068407	six9	0.366602899	-1.4477099	0.000863727	0.002149897
ENSDARG00000061829	UNC13A (1 of 2)	0.366044524	-1.449908954	1.06E-77	6.62E-76
ENSDARG00000030097	sdcbp	0.366016302	-1.45002019	8.37E-69	4.14E-67
ENSDARG00000039979	tecrl2	0.36593101	-1.450356415	1.10E-09	4.91E-09
ENSDARG00000055172	si:ch211-256m1.8	0.365870341	-1.450595627	2.22E-94	2.09E-92
ENSDARG00000060259	zgc:162160	0.365724582	-1.451170494	1.84E-09	8.07E-09
ENSDARG00000076044	TRHDE (2 of 2)	0.36556965	-1.451781793	8.87E-63	3.77E-61
ENSDARG00000075697	si:ch73-380l3.1	0.365504144	-1.452040332	2.26E-20	1.99E-19
ENSDARG00000019945	ptprdb	0.365497701	-1.452065765	2.77E-74	1.58E-72
ENSDARG00000070831	rftn1a	0.365261522	-1.452998313	1.08E-06	3.74E-06
ENSDARG00000079962	doc2d	0.365011282	-1.453987039	2.98E-17	2.20E-16
ENSDARG00000078190	zgc:193751	0.364948011	-1.454237136	4.93E-10	2.26E-09
ENSDARG00000087946	UNC13C (3 of 3)	0.364870953	-1.454541789	4.64E-07	1.66E-06
ENSDARG00000075676	FNDC7 (2 of 8)	0.364821322	-1.454738046	1.87E-38	3.62E-37
ENSDARG00000062013	TRHDE (1 of 2)	0.364499057	-1.456013012	1.27E-15	8.47E-15
ENSDARG00000090391	ugt2b3	0.364397788	-1.456413893	2.05E-15	1.35E-14
ENSDARG00000069254	chrm4a	0.364378613	-1.456489813	5.66E-18	4.34E-17
ENSDARG00000059818	kif5ab	0.364027348	-1.457881256	2.68E-18	2.10E-17
ENSDARG00000051823	hyal4	0.36400482	-1.457970543	2.91E-37	5.34E-36
ENSDARG00000057481	RNF157	0.363963735	-1.458133388	2.80E-86	2.11E-84
ENSDARG00000074372	elfn1b	0.363797401	-1.45879286	1.03E-18	8.28E-18
ENSDARG00000088307	mkl2a	0.36358479	-1.459636248	4.07E-06	1.33E-05
ENSDARG00000076443	CR388231.1	0.363572019	-1.459686921	2.54E-34	4.12E-33
ENSDARG00000074794	NECAB3	0.363411806	-1.460322807	1.14E-45	2.93E-44
ENSDARG00000061753	CABZ01083827.1	0.363391083	-1.460405078	0.000253726	0.000677039
ENSDARG00000036344	calb2b	0.363331148	-1.460643042	1.45E-93	1.33E-91
ENSDARG00000071375	celf5a	0.363261286	-1.460920475	8.38E-39	1.64E-37
ENSDARG00000028066	diras1a	0.363031823	-1.461832075	1.92E-88	1.51E-86
ENSDARG00000052099	agxta	0.36296017	-1.462116853	3.65E-43	8.62E-42

ENSDARG00000076248	ppp1r3db	0.362709133	-1.463115022	8.71E-15	5.53E-14
ENSDARG00000063215	lrrc3b	0.362640275	-1.463388934	3.82E-07	1.38E-06
ENSDARG00000079414	sez6b	0.362612649	-1.463498842	6.43E-74	3.63E-72
ENSDARG00000028857	SGSM1 (1 of 2)	0.362521627	-1.46386103	3.22E-29	4.25E-28
ENSDARG00000077715	GRIK3	0.362437415	-1.464196202	5.27E-28	6.60E-27
ENSDARG00000071205	tmem170b	0.36243599	-1.464201871	0.01041357	0.022016575
ENSDARG00000039145	plaub	0.362296245	-1.464758243	4.63E-05	0.000135588
ENSDARG00000044053	vsnl1b	0.362102558	-1.465529727	1.35E-93	1.24E-91
ENSDARG00000060539	SHANK1 (1 of 2)	0.362043487	-1.465765096	2.27E-79	1.49E-77
ENSDARG00000062538	TMEM132B	0.362016905	-1.465871028	6.78E-24	7.11E-23
ENSDARG00000052766	EVI5L	0.361898034	-1.466344824	9.36E-33	1.43E-31
ENSDARG00000057728	slc17a8	0.361745593	-1.466952655	6.16E-08	2.38E-07
ENSDARG00000039385	dlg4b	0.361497435	-1.467942686	5.91E-47	1.58E-45
ENSDARG00000088315	si:ch211-103f14.3	0.361234293	-1.468993236	1.67E-22	1.64E-21
ENSDARG00000045979		0.361080847	-1.469606197	4.48E-35	7.53E-34
ENSDARG00000036031	sept5b	0.361027162	-1.469820714	3.36E-94	3.15E-92
ENSDARG00000016739	LRRC4C	0.360983488	-1.469995246	1.63E-23	1.68E-22
ENSDARG00000043410	xkr6	0.360712856	-1.471077252	3.75E-20	3.26E-19
ENSDARG00000091573	CU693477.1	0.360687541	-1.471178504	0.001477937	0.003561112
ENSDARG00000057751	zbtb47a	0.360657993	-1.471296697	8.20E-12	4.28E-11
ENSDARG00000061819	wscd2	0.36063913	-1.471372154	4.94E-13	2.79E-12
ENSDARG00000061517	lrp1bb	0.360595734	-1.471545766	9.24E-80	6.13E-78
ENSDARG00000020979	FAM65C	0.36038895	-1.472373317	8.00E-44	1.95E-42
ENSDARG00000013020	dtnbb	0.360163768	-1.473275039	1.95E-38	3.76E-37
ENSDARG00000059763	gabrd	0.360133818	-1.473395014	1.91E-46	5.02E-45
ENSDARG00000033635	NPFFR2.1	0.359529553	-1.475817731	3.63E-05	0.000107574
ENSDARG00000098051	si:busm1-234g15.3	0.359284335	-1.47680206	1.88E-08	7.63E-08
ENSDARG00000079670	lrrc7	0.358958972	-1.478109139	3.11E-52	9.88E-51
ENSDARG00000056876	si:dkey-43b14.9	0.35875355	-1.478934988	0.00310564	0.007159477
ENSDARG00000090084	PLCB1	0.358706751	-1.479123199	1.17E-12	6.46E-12

ENSDARG00000088741	si:dkey-19n13.5	0.358490207	-1.479994387	3.80E-58	1.43E-56
ENSDARG00000039626	nrgna	0.358472581	-1.480065323	2.15E-26	2.53E-25
ENSDARG00000075170	ZDHHC22	0.358449583	-1.480157883	1.04E-32	1.59E-31
ENSDARG00000057678	sfrp1b	0.358304637	-1.480741382	1.97E-25	2.21E-24
ENSDARG00000070575	fam131bb	0.358290512	-1.480798254	6.21E-23	6.23E-22
ENSDARG00000036560	si:dkey-24g18.3	0.358215416	-1.481100671	2.13E-09	9.28E-09
ENSDARG00000091977	si:dkey-195m1.4	0.358193925	-1.481187224	0.000903167	0.002240805
ENSDARG00000028552	lppr3b	0.35818257	-1.48123296	5.67E-45	1.43E-43
ENSDARG00000097472	si:dkey-45l12.1	0.35810743	-1.481535643	5.91E-05	0.00017103
ENSDARG00000063157	mapk8ip2	0.357973092	-1.482076947	2.10E-91	1.81E-89
ENSDARG00000006341	tas1r3	0.357929472	-1.482252755	0.010647372	0.022474094
ENSDARG00000089562	BX957322.2	0.357876073	-1.482468003	6.65E-53	2.16E-51
ENSDARG00000052667	snn	0.357816137	-1.482709644	2.61E-20	2.30E-19
ENSDARG00000079977	NHS (2 of 2)	0.357783707	-1.482840404	6.21E-60	2.46E-58
ENSDARG00000056037	itih6	0.357688682	-1.483223625	2.66E-28	3.37E-27
ENSDARG00000094197	si:ch211-196c10.11	0.357539726	-1.483824546	1.36E-17	1.02E-16
ENSDARG00000058117	snap25b	0.357351815	-1.48458298	2.82E-99	2.92E-97
ENSDARG00000093123		0.357327819	-1.484679857	5.49E-11	2.70E-10
ENSDARG00000018270	olfm1a	0.357242946	-1.485022569	1.99E-81	1.36E-79
ENSDARG00000042531	RYR3 (1 of 3)	0.35703103	-1.48587863	0.000342865	0.000901187
ENSDARG00000061162	ephx4	0.357010421	-1.48596191	6.69E-15	4.28E-14
ENSDARG00000063282		0.35692862	-1.486292506	8.38E-08	3.20E-07
ENSDARG00000014287	eno2	0.356766228	-1.486949041	5.93E-98	6.02E-96
ENSDARG00000074376	MDGA1	0.356730026	-1.487095444	1.63E-15	1.08E-14
ENSDARG00000002394	ugt5d1	0.356601097	-1.487616953	2.56E-06	8.54E-06
ENSDARG00000056753	dctn1b	0.356546881	-1.487836312	9.75E-85	7.12E-83
ENSDARG00000011941	bmpr2a	0.356441864	-1.488261305	2.70E-93	2.43E-91
ENSDARG00000087877		0.356309912	-1.488795477	0.001411744	0.003411165
ENSDARG00000034862	f7	0.356202887	-1.489228886	8.26E-46	2.13E-44
ENSDARG00000023609	epha8	0.356166096	-1.489377903	4.46E-39	8.87E-38

ENSDARG00000069026		0.356105945	-1.489621574	0.006427484	0.014102248
ENSDARG00000086272	si:dkey-4p15.5	0.356085641	-1.489703832	9.85E-36	1.71E-34
ENSDARG00000095963	si:ch73-361h17.1	0.355956925	-1.490225425	1.23E-06	4.23E-06
ENSDARG00000086462	PTPRR	0.355740439	-1.491103114	5.53E-23	5.57E-22
ENSDARG00000008100	slc7a10a	0.355673041	-1.491376468	8.93E-94	8.30E-92
ENSDARG00000037359	CDR2 (2 of 2)	0.355242382	-1.493124383	0.000128531	0.000357333
ENSDARG00000039500	LRFN3	0.355209236	-1.493259001	8.81E-18	6.71E-17
ENSDARG00000055622	zgc:153345	0.355017629	-1.49403743	0.000440663	0.001141451
ENSDARG00000097576	VAMP1 (2 of 2)	0.354677122	-1.495421823	4.49E-14	2.72E-13
ENSDARG00000036913	st6galnac6	0.354662133	-1.495482793	3.93E-10	1.81E-09
ENSDARG00000088852	CABZ01051991.1	0.35457164	-1.495850947	7.05E-27	8.45E-26
ENSDARG00000063713	SYNGAP1 (1 of 2)	0.354524912	-1.496041086	3.92E-65	1.76E-63
ENSDARG00000029590	jph3	0.354457887	-1.496313863	3.77E-62	1.57E-60
ENSDARG00000052764	chrnb3a	0.354152132	-1.497558869	3.36E-51	1.03E-49
ENSDARG00000078908	fbxo41	0.353922034	-1.498496513	3.68E-25	4.08E-24
ENSDARG00000076213	DOCK4 (2 of 2)	0.35366585	-1.499541175	5.77E-44	1.41E-42
ENSDARG00000079971	CABZ01080134.1	0.353657881	-1.499573686	1.40E-05	4.34E-05
ENSDARG00000091823		0.353376179	-1.500723305	1.05E-13	6.19E-13
ENSDARG00000059057	atpv0e2	0.352983266	-1.502328306	2.09E-62	8.82E-61
ENSDARG00000004187		0.352681617	-1.503561716	3.49E-29	4.59E-28
ENSDARG00000044850	AL627305.1	0.352659306	-1.503652984	8.24E-05	0.000234996
ENSDARG00000088405	svilb	0.352481026	-1.504382497	1.59E-17	1.19E-16
ENSDARG00000078078	lrfn4b	0.352265009	-1.505266917	2.88E-56	1.04E-54
ENSDARG00000063169	UBN1	0.352239512	-1.505371343	7.63E-08	2.92E-07
ENSDARG00000062462	SHISA7 (1 of 2)	0.352088775	-1.505988864	3.87E-44	9.51E-43
ENSDARG00000090883	gabra3	0.352028312	-1.50623663	1.21E-08	4.98E-08
ENSDARG00000074317	FAM20C (1 of 2)	0.351991835	-1.50638613	1.19E-08	4.88E-08
ENSDARG00000089874	pcdh2ab8	0.35193281	-1.506628076	1.07E-09	4.81E-09
ENSDARG00000069296	moxd1l	0.351729944	-1.507459935	4.86E-11	2.40E-10
ENSDARG00000070038	rbp2a	0.351626469	-1.507884419	2.01E-101	2.17E-99

ENSDARG00000060875	pde1a	0.351545859	-1.508215194	3.93E-23	3.99E-22
ENSDARG00000041431	slc24a1	0.351290364	-1.509264089	6.08E-07	2.16E-06
ENSDARG00000032714	gria1b	0.351108176	-1.510012504	5.61E-26	6.49E-25
ENSDARG00000062618		0.3509881	-1.510505976	2.18E-42	4.95E-41
ENSDARG00000045799	acanb	0.350837909	-1.511123452	1.57E-80	1.06E-78
ENSDARG00000042922	gpr173	0.350733858	-1.511551387	1.93E-48	5.41E-47
ENSDARG00000079620	amigo1	0.350503839	-1.512497849	1.53E-39	3.11E-38
ENSDARG00000040490	dusp19a	0.350389543	-1.512968375	0.000172503	0.000471011
ENSDARG00000079182	NPAS3	0.350375139	-1.513027683	2.69E-102	2.95E-100
ENSDARG00000017391	unc13ba	0.350263858	-1.513485963	1.64E-12	8.95E-12
ENSDARG00000037553	il1rapl2	0.350151277	-1.513949744	8.98E-06	2.83E-05
ENSDARG00000018065	ntm	0.35011546	-1.514097328	1.77E-11	9.05E-11
ENSDARG00000089380	RASAL1 (2 of 2)	0.350045796	-1.514384414	0.003208374	0.007375963
ENSDARG00000062723	ZFYVE9 (2 of 3)	0.349892823	-1.515015023	6.17E-05	0.000178243
ENSDARG00000041791	mgat4c	0.349414538	-1.516988458	4.07E-22	3.90E-21
ENSDARG00000090923	CABZ01053592.1	0.349304111	-1.517444471	3.36E-19	2.78E-18
ENSDARG00000055722	bco2a	0.349187304	-1.517926989	3.29E-14	2.01E-13
ENSDARG00000090721	prdm2a	0.34911738	-1.518215917	1.42E-64	6.29E-63
ENSDARG00000043483	otx5	0.349020827	-1.518614966	1.69E-93	1.53E-91
ENSDARG00000062744	scn1lab	0.348907148	-1.519084941	7.64E-92	6.70E-90
ENSDARG00000095603	grid2ipb	0.348745185	-1.519754797	5.24E-41	1.12E-39
ENSDARG00000056877	vamp2	0.348663879	-1.520091185	1.37E-93	1.25E-91
ENSDARG00000060482	mid1	0.348451926	-1.520968468	3.21E-22	3.10E-21
ENSDARG00000037738	FBXL21	0.348266704	-1.521735543	8.01E-07	2.81E-06
ENSDARG00000068026	PRKACA	0.348191014	-1.522049125	0.000169893	0.000464284
ENSDARG00000059719	fam169aa	0.348090634	-1.522465098	2.55E-20	2.24E-19
ENSDARG00000062926	prkar1b	0.347728267	-1.523967746	2.43E-72	1.32E-70
ENSDARG00000017446	camk1db	0.347515023	-1.524852748	2.02E-22	1.97E-21
ENSDARG00000059601	map1aa	0.347470731	-1.525036635	2.44E-64	1.07E-62
ENSDARG00000070037	rgs8	0.347040597	-1.526823654	8.07E-37	1.45E-35

ENSDARG00000052615	tnmd	0.346830476	-1.527697422	6.39E-88	4.96E-86
ENSDARG00000061541	unc5db	0.346818851	-1.527745778	7.48E-24	7.83E-23
ENSDARG00000038859	rgs20	0.346399161	-1.529492659	3.11E-66	1.46E-64
ENSDARG00000009311	pvalb6	0.346050774	-1.530944363	1.23E-40	2.59E-39
ENSDARG00000043835	rab3ab	0.345976764	-1.531252945	1.60E-52	5.15E-51
ENSDARG00000044034	tmem86a	0.345822688	-1.531895573	2.96E-37	5.42E-36
ENSDARG00000059368	gria4b	0.34559701	-1.532837361	1.34E-91	1.17E-89
ENSDARG00000086919	CABZ01073963.1	0.345328662	-1.533958012	1.07E-06	3.72E-06
ENSDARG00000038918	drd1b	0.344864742	-1.535897457	6.88E-11	3.36E-10
ENSDARG00000037954	tnnt1	0.344549683	-1.537216064	2.90E-22	2.81E-21
ENSDARG00000013669	napba	0.34449382	-1.537449994	1.03E-72	5.66E-71
ENSDARG00000063011	antxr2b	0.344327162	-1.538148104	1.07E-12	5.89E-12
ENSDARG00000093027	BX119910.12	0.344229492	-1.538557391	6.03E-10	2.74E-09
ENSDARG00000042846	disp2	0.34420695	-1.538651867	8.83E-81	5.99E-79
ENSDARG00000063008	si:dkeyp-27e10.3	0.344127894	-1.538983257	3.18E-88	2.49E-86
ENSDARG00000041086	CABZ01071177.1	0.344116038	-1.539032962	0.001994339	0.004725247
ENSDARG00000061749	htr3b	0.343719954	-1.540694486	1.78E-09	7.80E-09
ENSDARG00000079295	cacna1bb	0.343415543	-1.541972758	4.03E-51	1.23E-49
ENSDARG00000077489	LRFN2 (2 of 2)	0.34332768	-1.542341918	8.92E-29	1.16E-27
ENSDARG00000052642	shisa9b	0.343326028	-1.542348861	0.000108626	0.000304919
ENSDARG00000061019	SHANK2 (1 of 2)	0.343223627	-1.542779225	9.53E-12	4.95E-11
ENSDARG00000086787	CABZ01084877.1	0.342968248	-1.543853079	1.01E-08	4.20E-08
ENSDARG00000087644	PCMTD2 (2 of 2)	0.342885382	-1.544201697	2.54E-46	6.63E-45
ENSDARG00000077022	fam131a	0.34242014	-1.546160538	5.82E-47	1.56E-45
ENSDARG00000045301	st8sia3	0.342381192	-1.546324641	7.54E-11	3.67E-10
ENSDARG00000056603	mcf2b	0.342356339	-1.546429368	1.22E-05	3.80E-05
ENSDARG00000057498	habp2	0.341948574	-1.548148721	9.33E-29	1.21E-27
ENSDARG00000015623	cecr1b	0.341672776	-1.549312793	1.76E-10	8.34E-10
ENSDARG00000062077	acsbg1	0.341599155	-1.549623688	4.61E-06	1.50E-05
ENSDARG00000076371	SHROOM2 (1 of 2)	0.341516395	-1.549973255	1.17E-52	3.79E-51

ENSDARG00000037904	cacna1db	0.341424571	-1.550361209	1.28E-09	5.69E-09
ENSDARG00000045383	GPR22 (2 of 2)	0.341289301	-1.550932908	2.66E-12	1.43E-11
ENSDARG00000093460	GPR52	0.341226641	-1.551197806	7.62E-11	3.71E-10
ENSDARG00000043589	ca4a	0.341167562	-1.551447614	3.47E-26	4.05E-25
ENSDARG00000052765	gria2b	0.340820174	-1.552917362	3.68E-105	4.24E-103
ENSDARG00000038398	PMM1	0.34060233	-1.55383979	4.70E-11	2.32E-10
ENSDARG00000062471	fam19a4b	0.340504845	-1.554252767	4.83E-11	2.39E-10
ENSDARG00000076824	CCKBR (2 of 2)	0.34049639	-1.55428859	0.00205455	0.004855322
ENSDARG00000009281	dnm1b	0.340143912	-1.555782827	1.34E-107	1.65E-105
ENSDARG00000088075	GRAMD3	0.33998502	-1.556456912	1.50E-08	6.10E-08
ENSDARG00000021573	slc16a7	0.339594428	-1.558115308	0.000141905	0.000392021
ENSDARG00000087762	RAB27B	0.339478772	-1.558606731	4.98E-27	6.00E-26
ENSDARG00000086314	CU633991.1	0.339450279	-1.558727826	0.005349041	0.011880115
ENSDARG00000025189	cpne8	0.339025954	-1.560532372	1.41E-06	4.83E-06
ENSDARG00000071592	aqp8a.2	0.339013241	-1.560586472	0.001619201	0.003881084
ENSDARG00000076013	mab21l3	0.33890221	-1.561059051	7.42E-06	2.36E-05
ENSDARG00000076264	zgc:195170	0.338682285	-1.561995568	0.003324935	0.007621118
ENSDARG00000035606	aldh3b2	0.338664268	-1.562072315	0.001615514	0.003873226
ENSDARG00000077609	elfn2	0.338604142	-1.562328472	2.64E-24	2.82E-23
ENSDARG00000060837	trpc5b	0.33840368	-1.563182837	8.33E-11	4.05E-10
ENSDARG00000093745	htr1aa	0.338250209	-1.56383727	0.001284907	0.003120614
ENSDARG00000011088	si:ch211-288d18.1	0.337981166	-1.564985242	3.14E-45	7.96E-44
ENSDARG00000040528	lgals3bpb	0.337929833	-1.565204377	4.08E-33	6.33E-32
ENSDARG00000016460	fut9a	0.337905719	-1.565307328	1.13E-60	4.54E-59
ENSDARG00000055192	zgc:136930	0.337900326	-1.565330351	7.15E-30	9.71E-29
ENSDARG00000090748	pcdh2ab9	0.337781396	-1.565838222	0.001275982	0.003100529
ENSDARG00000019930	tal1	0.337778314	-1.565851387	1.17E-82	8.29E-81
ENSDARG00000093323	NLRP6 (110 of 145)	0.337697105	-1.566198283	1.34E-15	8.98E-15
ENSDARG00000021065	camk2b2	0.337544075	-1.566852199	5.43E-08	2.11E-07
ENSDARG00000086133	SERINC3	0.337535487	-1.566888906	1.98E-06	6.68E-06

ENSDARG00000055559	chrna6	0.337190526	-1.568364095	8.83E-19	7.10E-18
ENSDARG00000015537	gad2	0.336854998	-1.569800392	6.62E-103	7.32E-101
ENSDARG00000053487	osbp2	0.336600805	-1.570889466	2.05E-39	4.15E-38
ENSDARG00000030376	grin2bb	0.336262475	-1.572340304	1.87E-85	1.38E-83
ENSDARG00000063207	erbb4a	0.336205803	-1.57258347	8.40E-19	6.77E-18
ENSDARG00000086992	BX005106.1	0.335846098	-1.574127826	1.93E-06	6.51E-06
ENSDARG00000074066	tas2r200.2	0.335745673	-1.574559287	0.000619979	0.001573511
ENSDARG00000043918	TACR3 (2 of 3)	0.335369682	-1.576175823	0.00128541	0.003121569
ENSDARG00000053746	OSBPL1A (2 of 2)	0.335027865	-1.577647002	6.67E-12	3.50E-11
ENSDARG00000043907	fgf11b	0.334862229	-1.578360441	1.55E-34	2.54E-33
ENSDARG00000096687	RGS7 (2 of 2)	0.334830679	-1.578496375	1.19E-08	4.90E-08
ENSDARG00000074640		0.334604548	-1.579471042	7.11E-05	0.000204197
ENSDARG00000036156	fnbp1	0.334458873	-1.580099277	1.14E-47	3.13E-46
ENSDARG00000029112	gem	0.33435444	-1.580549821	2.25E-16	1.58E-15
ENSDARG00000071637	ASIC1 (2 of 2)	0.334189905	-1.58125994	3.40E-07	1.24E-06
ENSDARG00000009442	pip5k1cb	0.334033994	-1.581933165	7.94E-17	5.75E-16
ENSDARG00000035765	CU694757.1	0.333895991	-1.582529322	2.68E-07	9.83E-07
ENSDARG00000089156	egr3	0.333576091	-1.583912209	4.49E-32	6.66E-31
ENSDARG00000058695	ddr2b	0.333553909	-1.584008145	4.45E-49	1.28E-47
ENSDARG00000018263	pdia2	0.333541968	-1.584059793	3.79E-23	3.85E-22
ENSDARG00000077905	CSMD3	0.333507914	-1.584207099	6.51E-16	4.42E-15
ENSDARG00000042703	parp6a	0.333338453	-1.584940342	7.24E-46	1.87E-44
ENSDARG00000096922	si:ch211-147k10.6	0.333233896	-1.585392936	8.28E-13	4.61E-12
ENSDARG00000062156	ABI3BP (1 of 2)	0.333176274	-1.585642426	0.001027682	0.002533362
ENSDARG00000062510	bcl11ba	0.333017468	-1.58633024	2.26E-110	2.96E-108
ENSDARG00000044212	CR385063.1	0.332377383	-1.589105879	3.42E-51	1.05E-49
ENSDARG00000025891	shox	0.332187393	-1.589930774	1.59E-109	2.04E-107
ENSDARG00000093768	prr18	0.331874238	-1.591291452	1.90E-107	2.33E-105
ENSDARG00000036999	PTCHD2	0.331724887	-1.591940843	1.32E-30	1.85E-29
ENSDARG00000075189	cntnap5b	0.331647039	-1.592279449	1.77E-58	6.76E-57

ENSDARG00000076652	zgc:193807	0.331376621	-1.593456274	0.000116477	0.000325574
ENSDARG00000088837	CR631124.1	0.331202404	-1.594214952	0.001300513	0.003156627
ENSDARG00000036767		0.331051366	-1.594873011	5.46E-33	8.45E-32
ENSDARG00000020191	ppef1	0.331021127	-1.595004797	7.54E-09	3.15E-08
ENSDARG00000037084	CPNE7	0.330919631	-1.595447215	1.93E-25	2.17E-24
ENSDARG00000090496	THSD7A (2 of 2)	0.330790975	-1.596008219	2.97E-13	1.71E-12
ENSDARG00000034165	lingo1a	0.330515962	-1.597208149	2.77E-44	6.82E-43
ENSDARG00000035652	sat1a.1	0.330290082	-1.598194445	1.31E-87	1.01E-85
ENSDARG00000056080	si:dkey-191g9.5	0.33026649	-1.598297498	1.25E-19	1.06E-18
ENSDARG00000092552	FNDC7 (7 of 8)	0.330194675	-1.59861124	0.000634102	0.001607489
ENSDARG00000086169	sgk494b	0.329990889	-1.599501903	6.74E-13	3.77E-12
ENSDARG00000053624	csf1rb	0.32944345	-1.601897249	1.67E-07	6.22E-07
ENSDARG00000074761	ccdc64	0.32933372	-1.602377859	5.11E-13	2.89E-12
ENSDARG00000004015	adcyap1a	0.329245438	-1.602764644	4.86E-06	1.57E-05
ENSDARG00000029916	KCNQ2 (1 of 2)	0.329133024	-1.603257307	9.73E-07	3.38E-06
ENSDARG00000086960	GPC6 (2 of 3)	0.328999107	-1.603844426	9.18E-05	0.000260445
ENSDARG00000083909	5S_rRNA	0.328548929	-1.605819857	0.008835296	0.018907321
ENSDARG00000043951	pcdh1g9	0.328530946	-1.605898823	0.000313153	0.000827069
ENSDARG00000078647	samsn1b	0.328384447	-1.606542297	0.006894388	0.015038404
ENSDARG00000060018	C14H5orf24 (1 of 2)	0.328091926	-1.607828004	9.69E-76	5.80E-74
ENSDARG00000091395	CABZ01067945.1	0.328061404	-1.607962222	2.82E-114	3.98E-112
ENSDARG00000092945	si:ch211-250g4.3	0.328023511	-1.60812887	0.000191703	0.000519839
ENSDARG00000095253	CU571315.2	0.328018818	-1.608149512	1.03E-05	3.21E-05
ENSDARG00000037905	cacna1aa	0.327922407	-1.60857361	6.54E-108	8.11E-106
ENSDARG00000088061	si:dkeyp-72e1.9	0.327602136	-1.60998333	3.44E-26	4.02E-25
ENSDARG00000077389	PCDHGC4 (6 of 9)	0.32748943	-1.610479753	3.63E-66	1.69E-64
ENSDARG00000038814	myrip	0.32738038	-1.61096023	3.79E-91	3.21E-89
ENSDARG00000039218	GPR101	0.32737345	-1.610990772	7.00E-13	3.91E-12
ENSDARG00000086802		0.327238946	-1.611583637	7.84E-09	3.27E-08
ENSDARG00000015531	SLC4A8	0.326978414	-1.612732698	6.14E-39	1.22E-37

ENSDARG00000088418	PCDHGC5 (19 of 31)	0.326848334	-1.613306751	7.29E-05	0.000209029
ENSDARG00000054184	btr06	0.326750726	-1.613737653	0.000811047	0.002025337
ENSDARG00000071894	pcdh2g3	0.326727157	-1.613841721	2.25E-08	9.05E-08
ENSDARG00000045164	nptx2b	0.32672014	-1.613872707	0.006972264	0.015197771
ENSDARG00000071076	ldhbb	0.326510958	-1.614796686	4.37E-25	4.83E-24
ENSDARG00000058803	grk1a	0.326492451	-1.614878461	9.48E-17	6.83E-16
ENSDARG00000025667	bai2	0.326390816	-1.615327629	1.21E-23	1.26E-22
ENSDARG00000021660	bsk146	0.326190226	-1.616214543	6.38E-111	8.44E-109
ENSDARG00000090424	camk2n2	0.326109268	-1.61657265	6.85E-31	9.71E-30
ENSDARG00000054690	si:dkey-166k12.1	0.325826661	-1.617823435	1.01E-29	1.37E-28
ENSDARG00000063299	pcloa	0.325825312	-1.617829412	5.21E-115	7.55E-113
ENSDARG00000078034	zgc:112001	0.325807641	-1.617907658	2.68E-34	4.34E-33
ENSDARG00000076459	pcdhb	0.325786626	-1.618000716	7.89E-08	3.02E-07
ENSDARG00000005966	slc4a5	0.325242283	-1.620413268	1.15E-13	6.79E-13
ENSDARG00000078504	CU459081.1	0.325241483	-1.620416818	6.49E-38	1.22E-36
ENSDARG00000087342	CABZ01117094.1	0.32519018	-1.620644405	3.85E-15	2.50E-14
ENSDARG00000062086	kiaa1549lb	0.325184046	-1.620671618	1.22E-12	6.73E-12
ENSDARG00000037916	cdk5r1a	0.325138152	-1.620875241	9.51E-33	1.46E-31
ENSDARG00000032838	si:dkey-206f10.1	0.324925813	-1.621817737	1.11E-18	8.90E-18
ENSDARG00000074018	btbd17a	0.324725327	-1.622708183	2.60E-17	1.93E-16
ENSDARG00000087061	si:ch211-71k14.1	0.324634641	-1.623111142	0.005429634	0.012049691
ENSDARG00000060597		0.324633078	-1.623118087	4.54E-67	2.17E-65
ENSDARG00000058701	nos1apb	0.324099761	-1.625490137	1.64E-29	2.18E-28
ENSDARG00000093937	cnn1b	0.324072918	-1.625609634	1.05E-07	3.96E-07
ENSDARG00000037747	si:ch211-160d14.1	0.323798538	-1.626831622	1.16E-35	2.00E-34
ENSDARG00000087517	btbd3b	0.32353359	-1.62801259	4.78E-48	1.32E-46
ENSDARG00000027419	gad1b	0.323343942	-1.628858514	1.45E-116	2.16E-114
ENSDARG00000042041	tal2	0.323221314	-1.629405761	1.36E-53	4.54E-52
ENSDARG00000076987	SLITRK5 (2 of 2)	0.323167371	-1.629646554	3.24E-54	1.10E-52
ENSDARG00000063180	dock3	0.323156858	-1.629693485	2.18E-75	1.28E-73

ENSDARG00000089257	EYS	0.323108702	-1.62990849	5.80E-05	0.00016794
ENSDARG00000091799	FAM217B (2 of 2)	0.322967363	-1.630539712	4.96E-27	5.98E-26
ENSDARG00000061362	FAM69A (1 of 2)	0.322913454	-1.630780542	1.36E-09	6.03E-09
ENSDARG00000070148	cygb2	0.322896764	-1.63085511	5.29E-19	4.32E-18
ENSDARG00000038363	drd4a	0.322886188	-1.630902368	5.10E-06	1.65E-05
ENSDARG00000023771	gabrb3	0.322644249	-1.631983786	4.53E-55	1.58E-53
ENSDARG00000055400	ELMO1 (2 of 2)	0.322601944	-1.632172963	3.13E-76	1.90E-74
ENSDARG00000077552	CABZ01081650.1	0.322342924	-1.633331779	4.76E-26	5.52E-25
ENSDARG00000097160	si:ch211-121j5.4	0.32227672	-1.633628119	1.46E-14	9.13E-14
ENSDARG00000036433	ERP27	0.322129119	-1.634289014	1.51E-06	5.15E-06
ENSDARG00000070504	trpc5a	0.322119987	-1.634329915	9.36E-05	0.000265233
ENSDARG00000055377	gnb5b	0.321409143	-1.637517128	4.72E-49	1.35E-47
ENSDARG00000056294	SBK1	0.321311136	-1.637957113	1.39E-41	3.03E-40
ENSDARG00000090164	NHSL2 (2 of 2)	0.321296283	-1.638023803	8.58E-31	1.21E-29
ENSDARG00000060116	CAMK1 (2 of 2)	0.321265091	-1.638163869	2.19E-44	5.42E-43
ENSDARG00000040435	SLC6A11	0.321138944	-1.638730468	6.95E-91	5.79E-89
ENSDARG00000086319	BSN (3 of 3)	0.321120025	-1.638815461	1.68E-12	9.15E-12
ENSDARG00000079586	gabrb2	0.320787478	-1.640310268	2.72E-38	5.19E-37
ENSDARG00000097610	si:ch211-136a14.2	0.320679258	-1.640797055	0.004276295	0.009634635
ENSDARG00000014871	syngr3a	0.320592741	-1.641186337	4.56E-119	7.19E-117
ENSDARG00000063264	PCDH9	0.320470707	-1.641735602	4.32E-90	3.50E-88
ENSDARG00000042826	egr2b	0.320434482	-1.641898691	3.81E-16	2.63E-15
ENSDARG00000096641	si:ch73-115f13.1	0.320326847	-1.642383377	2.27E-11	1.15E-10
ENSDARG00000079245	si:dkey-73p2.2	0.31973068	-1.64507091	1.10E-15	7.37E-15
ENSDARG00000074056	kctd1	0.319214958	-1.647399839	2.55E-28	3.24E-27
ENSDARG00000005586	zbtb20	0.319016313	-1.648297895	6.44E-08	2.48E-07
ENSDARG00000070781	cx35b	0.318977168	-1.648474935	7.57E-18	5.78E-17
ENSDARG00000056774	gpr37l1b	0.318864904	-1.648982781	2.86E-56	1.03E-54
ENSDARG00000018383	frzb	0.318746226	-1.649519836	5.78E-52	1.82E-50
ENSDARG00000079822	BSN (2 of 3)	0.318479109	-1.650729353	1.20E-112	1.65E-110

ENSDARG00000060638	CLSTN2 (2 of 2)	0.317774535	-1.653924578	2.65E-14	1.64E-13
ENSDARG00000002635	FAM184A (1 of 2)	0.317556121	-1.654916515	6.14E-82	4.26E-80
ENSDARG00000025478	GIPR	0.317550175	-1.654943528	4.08E-06	1.33E-05
ENSDARG00000027618	grip2b	0.317286151	-1.656143542	1.80E-07	6.71E-07
ENSDARG00000042090	PLA2G4C (3 of 5)	0.317078348	-1.657088731	0.002739672	0.006357067
ENSDARG00000022739	rxfp3.2a	0.317071665	-1.657119135	5.93E-05	0.000171681
ENSDARG00000037478	CDH8	0.316250234	-1.660861546	4.50E-86	3.37E-84
ENSDARG00000010244	rpl22l1	0.31623922	-1.660911792	8.18E-94	7.62E-92
ENSDARG00000041176	scn2b	0.315529143	-1.664154834	2.03E-58	7.73E-57
ENSDARG00000088587	PCDHGC5 (21 of 31)	0.315059679	-1.666302964	4.69E-05	0.000137364
ENSDARG00000086877	mhc1zaa	0.314974657	-1.666692341	3.86E-29	5.08E-28
ENSDARG00000063524	ano2	0.314846246	-1.667280629	2.11E-34	3.44E-33
ENSDARG00000076702	PSD (1 of 3)	0.31466848	-1.668095421	8.50E-17	6.14E-16
ENSDARG00000068683	chmp2ba	0.314603104	-1.66839519	2.35E-12	1.27E-11
ENSDARG00000074033	adamtsl2	0.314589022	-1.668459768	1.55E-15	1.03E-14
ENSDARG00000061909	PCDH10 (2 of 2)	0.31458249	-1.668489723	3.16E-66	1.48E-64
ENSDARG00000030614	syt1a	0.31455901	-1.668597408	9.42E-109	1.19E-106
ENSDARG00000027963	camkva	0.314522663	-1.668764121	7.92E-39	1.56E-37
ENSDARG00000093342	si:ch211-13f8.1	0.314152928	-1.670461069	1.49E-09	6.57E-09
ENSDARG00000076730	syt6a	0.313944791	-1.671417219	2.30E-32	3.47E-31
ENSDARG00000027564		0.31387302	-1.671747072	3.23E-16	2.23E-15
ENSDARG00000093317	si:ch211-209j10.6	0.313813389	-1.672021186	6.48E-18	4.96E-17
ENSDARG00000091614	CR848032.2	0.3134633	-1.673631549	2.02E-06	6.79E-06
ENSDARG00000041787	cx32.3	0.3132003	-1.6748425	1.99E-15	1.32E-14
ENSDARG00000086375	DGKQ	0.313184	-1.674917583	0.000250893	0.00066971
ENSDARG00000096324	si:ch211-155d24.7	0.313064831	-1.675466647	1.91E-08	7.74E-08
ENSDARG00000086799	MDGA2 (2 of 2)	0.312574097	-1.677729868	0.00065022	0.001645706
ENSDARG00000089429	si:dkey-205h13.2	0.311749334	-1.681541617	2.37E-110	3.09E-108
ENSDARG00000069590	tmem150c	0.311728514	-1.681637972	1.24E-29	1.66E-28
ENSDARG00000087343	hs3st1l1	0.311594865	-1.682256638	7.46E-20	6.40E-19

ENSDARG00000063014	dbpa	0.311378645	-1.683258088	1.78E-41	3.87E-40
ENSDARG00000094003	serp2	0.311071889	-1.684680068	3.10E-24	3.30E-23
ENSDARG00000055578	rtn4rl1b	0.311066337	-1.684705816	5.03E-13	2.84E-12
ENSDARG00000062934	gpr12	0.311019835	-1.684921504	4.09E-26	4.75E-25
ENSDARG00000088143	SEMA4G (2 of 2)	0.31075382	-1.68615597	1.25E-06	4.29E-06
ENSDARG00000036998	arf3b	0.310677786	-1.686509003	8.76E-63	3.73E-61
ENSDARG00000041926	DLG4 (2 of 2)	0.310419717	-1.687707898	5.23E-24	5.51E-23
ENSDARG00000011635	SCX (1 of 2)	0.310177954	-1.688831944	7.65E-07	2.69E-06
ENSDARG00000057468	kcnc2	0.31016024	-1.688914338	4.55E-20	3.94E-19
ENSDARG00000088268	OTOS	0.309964155	-1.689826706	1.70E-34	2.77E-33
ENSDARG00000038655	ajap1	0.309548854	-1.691760979	1.29E-54	4.46E-53
ENSDARG00000010690	CU138503.2	0.309411406	-1.692401716	0.000416474	0.001082544
ENSDARG00000079805	tagln3a	0.309175805	-1.693500669	9.47E-16	6.39E-15
ENSDARG00000017254	kcnk1b	0.308997232	-1.694334182	6.68E-13	3.74E-12
ENSDARG00000040633	CABZ01016567.1	0.30892137	-1.694688422	2.05E-38	3.94E-37
ENSDARG00000077708	snx8b	0.30884677	-1.695036854	0.000258248	0.00068859
ENSDARG00000077349	SORCS3 (2 of 2)	0.308320717	-1.697496265	7.44E-21	6.72E-20
ENSDARG00000021607	negr1	0.307734397	-1.700242386	1.22E-114	1.74E-112
ENSDARG00000090727	celf5b	0.307649593	-1.700640013	3.30E-09	1.41E-08
ENSDARG00000079285	pcdh1g32	0.307623593	-1.700761943	1.70E-72	9.27E-71
ENSDARG00000044638	map1lc3c	0.307589766	-1.700920593	0.000158665	0.000435823
ENSDARG00000077920	CR352286.1	0.30698096	-1.703778915	6.99E-12	3.66E-11
ENSDARG00000055525	lgalslb	0.306728672	-1.704965063	1.58E-34	2.59E-33
ENSDARG00000087583	si:ch211-226h8.13	0.306581677	-1.705656616	0.002115775	0.004985942
ENSDARG00000025672	antxr1a	0.30618261	-1.707535748	4.29E-10	1.98E-09
ENSDARG00000015349	mfge8a	0.30615391	-1.707670989	4.35E-88	3.38E-86
ENSDARG00000088380	pcdh2ab12	0.30593656	-1.708695571	0.005594425	0.012389274
ENSDARG00000011519	myl4	0.305711718	-1.709756243	1.72E-77	1.07E-75
ENSDARG00000011671	pde6b	0.305668576	-1.709959852	4.57E-16	3.13E-15
ENSDARG00000061031	dner	0.305655506	-1.710021541	2.76E-52	8.78E-51

ENSDARG00000053201	zgc:172323	0.305457073	-1.710958446	5.41E-08	2.10E-07
ENSDARG00000058799	mybpha	0.305243837	-1.711965929	3.62E-42	8.12E-41
ENSDARG00000083622	dre-mir-124-6	0.305070455	-1.712785629	8.87E-06	2.80E-05
ENSDARG00000094660	si:ch211-218d20.15	0.304988397	-1.713173738	2.98E-23	3.05E-22
ENSDARG00000087624	LRRC2	0.304664493	-1.714706722	1.77E-14	1.10E-13
ENSDARG00000031161	nr1d4a	0.304563373	-1.71518564	6.93E-06	2.21E-05
ENSDARG00000062693	nrxn3b	0.30453934	-1.715299489	4.55E-98	4.65E-96
ENSDARG00000097196	si:ch211-145n14.3	0.304529567	-1.715345789	2.29E-71	1.21E-69
ENSDARG00000055843	cdh10	0.304522326	-1.715380093	3.85E-57	1.42E-55
ENSDARG00000076707	pcdh2g29	0.304319683	-1.716340448	1.97E-12	1.07E-11
ENSDARG00000056572		0.304164317	-1.717077181	1.14E-25	1.29E-24
ENSDARG00000086155		0.303868331	-1.718481771	4.20E-06	1.37E-05
ENSDARG00000096721	si:rp71-1c10.11	0.303631289	-1.719607627	2.52E-23	2.58E-22
ENSDARG00000071462	lama3	0.303069903	-1.722277507	1.29E-30	1.81E-29
ENSDARG00000045568	bcat1	0.302843764	-1.723354391	6.11E-70	3.10E-68
ENSDARG00000052787	zdhhc12b	0.302650137	-1.724277092	5.12E-25	5.65E-24
ENSDARG00000070907	lcor	0.302546837	-1.724769593	3.86E-55	1.36E-53
ENSDARG00000055669	NCAM2 (2 of 2)	0.30252144	-1.724890703	7.50E-21	6.77E-20
ENSDARG00000002732	spon2b	0.302367153	-1.72562667	2.97E-54	1.01E-52
ENSDARG00000086479	khdrbs3	0.302358327	-1.725668783	8.22E-119	1.29E-116
ENSDARG00000087260	MTSS1L (2 of 2)	0.302271326	-1.726083968	1.20E-24	1.29E-23
ENSDARG00000093608	si:dkey-25o1.6	0.301997129	-1.727393263	3.44E-11	1.72E-10
ENSDARG00000088300	VSTM2A (2 of 2)	0.301994687	-1.727404924	0.002746072	0.006369317
ENSDARG00000054055	ostn	0.301984211	-1.727454974	9.91E-05	0.000279836
ENSDARG00000090106	SLC17A7 (2 of 2)	0.301953386	-1.727602245	2.46E-29	3.26E-28
ENSDARG00000070229	C11H1orf116 (1 of 2)	0.301904359	-1.727836508	0.001710954	0.004086094
ENSDARG00000077996		0.301696528	-1.728830003	9.19E-39	1.80E-37
ENSDARG00000028048	rdh8a	0.301634171	-1.729128218	1.02E-81	7.05E-80
ENSDARG00000002644	rgs5a	0.301603322	-1.729275774	9.25E-82	6.39E-80
ENSDARG00000002230	sypb	0.301181165	-1.731296544	7.00E-131	1.33E-128

ENSDARG00000017444	hal	0.301004289	-1.732144052	4.79E-53	1.56E-51
ENSDARG00000087406	CABZ01060681.1	0.300920199	-1.732547146	1.65E-06	5.59E-06
ENSDARG00000052896	lrit1b	0.300751392	-1.733356681	6.76E-24	7.09E-23
ENSDARG00000089538	RNF166 (2 of 2)	0.300501083	-1.734557904	2.09E-10	9.86E-10
ENSDARG00000057439	PHF1	0.300458346	-1.734763098	1.28E-06	4.39E-06
ENSDARG00000091607	ARHGEF28 (2 of 2)	0.300417223	-1.734960571	8.66E-06	2.73E-05
ENSDARG00000089101	plrdgb	0.299865081	-1.737614563	1.54E-07	5.76E-07
ENSDARG00000058045	tlr21	0.299858922	-1.737644194	0.000528909	0.001355207
ENSDARG00000090348	pcdh2ab5	0.299738639	-1.738223022	7.76E-05	0.00022176
ENSDARG00000053665	gabrg2	0.299708829	-1.738366511	2.98E-94	2.80E-92
ENSDARG00000042390	syndig1l	0.29946433	-1.739543923	4.45E-32	6.62E-31
ENSDARG00000056347	rab3aa	0.299462732	-1.739551623	1.91E-92	1.69E-90
ENSDARG00000071230	LRFN5 (1 of 2)	0.299457051	-1.739578995	3.59E-38	6.81E-37
ENSDARG00000069495	tmem179b	0.299446409	-1.739630263	1.21E-17	9.14E-17
ENSDARG00000020231	mapre3a	0.299315843	-1.740259451	2.29E-64	1.01E-62
ENSDARG00000011257	enpp2	0.298943881	-1.742053415	2.55E-08	1.02E-07
ENSDARG00000062659	CABZ01053721.1	0.298628323	-1.743577093	0.00215434	0.005071756
ENSDARG00000092469	FNDC7 (6 of 8)	0.298519042	-1.744105133	4.39E-09	1.87E-08
ENSDARG00000002760	gdf5	0.298307389	-1.74512838	5.67E-45	1.43E-43
ENSDARG00000091919	radil	0.297847264	-1.747355389	1.22E-10	5.85E-10
ENSDARG00000087190	MUC4	0.297666009	-1.748233608	4.40E-09	1.87E-08
ENSDARG00000057527	MEF2A (2 of 2)	0.297061029	-1.751168743	3.34E-12	1.78E-11
ENSDARG00000040177	rgs16	0.29667312	-1.753053875	5.85E-17	4.26E-16
ENSDARG00000038862	kcnb2	0.296506567	-1.753864039	6.92E-38	1.30E-36
ENSDARG00000045087	cdk5r1b	0.296064334	-1.756017393	1.01E-133	2.07E-131
ENSDARG00000062633	cadm2b	0.295913189	-1.756754095	1.13E-25	1.28E-24
ENSDARG00000054667	adra2db	0.295376128	-1.759374863	9.84E-05	0.000277828
ENSDARG00000046021	PI16	0.295269862	-1.759893987	0.002157071	0.005076102
ENSDARG00000079719	abhd8a	0.295112274	-1.760664171	2.23E-16	1.56E-15
ENSDARG00000062610	pcdh1g32	0.294700914	-1.762676562	2.10E-12	1.13E-11

ENSDARG00000089734	CABZ01065246.2	0.294411917	-1.764092026	1.56E-09	6.89E-09
ENSDARG00000025206	syt2a	0.2941358	-1.765445705	4.75E-67	2.26E-65
ENSDARG00000063483	RNF122	0.293948073	-1.766366775	1.13E-50	3.40E-49
ENSDARG00000044514	st6gal1	0.293742666	-1.767375265	3.64E-82	2.55E-80
ENSDARG00000089358	CABZ01035189.1	0.293552843	-1.768307865	9.55E-37	1.71E-35
ENSDARG00000034424	atp1b2b	0.293418646	-1.768967542	3.29E-79	2.15E-77
ENSDARG00000088181	MPPED1	0.293166232	-1.770209156	2.02E-13	1.17E-12
ENSDARG00000001559	csmd2	0.29293249	-1.771359879	8.33E-84	6.00E-82
ENSDARG00000070680	cdh4	0.292701287	-1.772499004	1.10E-14	6.93E-14
ENSDARG00000076997	stxbp4	0.292589529	-1.773049953	3.01E-15	1.97E-14
ENSDARG00000018351	hpda	0.292545514	-1.773267001	1.03E-44	2.57E-43
ENSDARG00000086531	CABZ01071530.2	0.29250326	-1.773475393	1.55E-07	5.79E-07
ENSDARG00000015752	zap70	0.292067624	-1.77562565	0.000155788	0.000428499
ENSDARG00000097187	si:ch211-153l6.6	0.292049445	-1.775715452	1.18E-23	1.22E-22
ENSDARG00000096242	si:ch211-76l23.4	0.292036502	-1.77577939	5.89E-86	4.39E-84
ENSDARG00000042383	zbtb25	0.292025925	-1.775831643	3.79E-59	1.48E-57
ENSDARG00000069973	samd12	0.291504761	-1.778408648	0.00019541	0.000529288
ENSDARG00000059891		0.291478962	-1.778536335	2.90E-07	1.06E-06
ENSDARG00000077710	nlgn1	0.291445514	-1.778701899	2.38E-20	2.10E-19
ENSDARG00000003259	lox	0.291415034	-1.778852787	4.80E-26	5.56E-25
ENSDARG00000077187	impg1a	0.291291908	-1.779462468	3.56E-11	1.78E-10
ENSDARG00000069196	ebf1b	0.291222477	-1.779806385	2.36E-27	2.89E-26
ENSDARG00000087652	tcf4	0.291164846	-1.780091912	3.00E-32	4.49E-31
ENSDARG00000042397	zgc:158340	0.291080632	-1.780509249	7.59E-72	4.07E-70
ENSDARG00000095509	si:dkeyp-91h9.1	0.2909404	-1.781204454	2.97E-05	8.87E-05
ENSDARG00000038372	cldn15lb	0.290651849	-1.782636006	5.32E-19	4.34E-18
ENSDARG00000069804	RBBP6 (6 of 71)	0.290569974	-1.783042466	0.003380512	0.007736648
ENSDARG00000094766	CCBL2 (4 of 4)	0.290075347	-1.785500404	7.80E-05	0.000223045
ENSDARG00000087086	MGAT5B	0.28886566	-1.791529385	4.40E-06	1.43E-05
ENSDARG00000060458	HRH4 (2 of 2)	0.288838066	-1.791667205	0.000158734	0.000435927

ENSDARG00000044129	si:ch211-214p16.1	0.28876117	-1.79205134	7.57E-07	2.66E-06
ENSDARG00000068019	sox8a	0.288457514	-1.793569251	1.22E-21	1.14E-20
ENSDARG00000040198	fstl5	0.287909486	-1.79631277	9.69E-127	1.68E-124
ENSDARG00000038116	gnrhr4	0.287844425	-1.796638826	0.004378043	0.009851377
ENSDARG00000002585		0.287790283	-1.796910215	1.66E-11	8.47E-11
ENSDARG00000053559	tspan3b	0.287113134	-1.800308765	1.31E-59	5.16E-58
ENSDARG00000093753	si:ch211-133l5.5	0.287056396	-1.800593894	1.18E-13	6.97E-13
ENSDARG00000062343	NETO2 (1 of 2)	0.286729413	-1.80223819	3.88E-41	8.31E-40
ENSDARG00000055014	CR558302.2	0.286674864	-1.80251268	4.34E-12	2.30E-11
ENSDARG00000053424	myl2b	0.286619099	-1.802793349	2.49E-14	1.53E-13
ENSDARG00000022820	pde6g	0.286524368	-1.803270255	1.69E-62	7.14E-61
ENSDARG00000076981	zgc:198329	0.286429898	-1.803746002	2.25E-05	6.80E-05
ENSDARG00000071493	olfm3a	0.285402649	-1.80892937	3.46E-42	7.77E-41
ENSDARG00000059645	ntrk2b	0.285334226	-1.809275285	1.76E-38	3.41E-37
ENSDARG00000042114	BIN1 (1 of 2)	0.284637313	-1.812803296	8.37E-58	3.13E-56
ENSDARG00000045242	smyhc3	0.28435532	-1.814233298	3.28E-11	1.64E-10
ENSDARG00000062305	rims3	0.284315552	-1.814435079	1.86E-14	1.15E-13
ENSDARG00000077128	galnt13	0.284274129	-1.814645284	1.56E-42	3.56E-41
ENSDARG00000090115	HCN2 (2 of 2)	0.284267779	-1.814677513	2.50E-12	1.35E-11
ENSDARG00000077151	cbln2b	0.284022988	-1.815920392	2.77E-75	1.62E-73
ENSDARG00000011171	TENM2 (1 of 2)	0.283928849	-1.81639865	1.55E-127	2.74E-125
ENSDARG00000058462	zgc:158846	0.28372278	-1.817446106	2.83E-61	1.16E-59
ENSDARG00000070624	cacng7b	0.283645368	-1.817839788	1.77E-05	5.42E-05
ENSDARG00000045306	slc51a	0.283478059	-1.818691021	1.04E-15	6.99E-15
ENSDARG00000094881	si:ch211-197h24.8	0.283309941	-1.81954687	1.12E-05	3.51E-05
ENSDARG00000090676		0.282229747	-1.82505804	0.003268171	0.007500689
ENSDARG00000023151	ucp1	0.28209566	-1.825743623	2.62E-143	5.92E-141
ENSDARG00000009637		0.281697018	-1.827783801	4.07E-59	1.58E-57
ENSDARG00000039647	slc6a1b	0.281694577	-1.827796303	5.33E-115	7.69E-113
ENSDARG00000078665		0.28162174	-1.828169384	5.90E-11	2.90E-10

ENSDARG00000003970	trim35-13	0.281548289	-1.828545712	2.52E-21	2.33E-20
ENSDARG00000020771	tnr	0.281190789	-1.830378759	5.88E-106	6.88E-104
ENSDARG00000088161	BAI2 (2 of 2)	0.28107972	-1.830948726	7.53E-17	5.46E-16
ENSDARG00000057000	camkvl	0.280734507	-1.832721691	1.22E-39	2.47E-38
ENSDARG00000042970	gng8	0.28055612	-1.833638714	4.93E-16	3.37E-15
ENSDARG00000094533	si:dkey-246e1.3	0.279941518	-1.836802629	6.05E-05	0.000174752
ENSDARG00000077249	gmip	0.279853293	-1.837257369	1.08E-24	1.18E-23
ENSDARG00000090196	SYN3	0.279383237	-1.839682634	5.68E-18	4.35E-17
ENSDARG00000097209	cox8b	0.279238051	-1.84043255	8.07E-39	1.59E-37
ENSDARG00000029230	pnp4b	0.27922459	-1.840502098	5.69E-42	1.26E-40
ENSDARG00000091185	si:ch73-313h21.1	0.279064306	-1.841330488	0.000406078	0.001057165
ENSDARG00000055730	trim35-29	0.278987037	-1.841730005	0.002694253	0.006256277
ENSDARG00000021909	VSTM2A (1 of 2)	0.27859832	-1.843741539	6.80E-06	2.17E-05
ENSDARG00000018266	mthfd1a	0.278569664	-1.843889939	8.55E-146	2.06E-143
ENSDARG00000043460	wu:fj39g12	0.277442951	-1.849736947	7.78E-11	3.79E-10
ENSDARG00000096712	si:dkey-193p11.2	0.277401422	-1.849952913	6.99E-29	9.09E-28
ENSDARG00000044326	BX950188.1	0.277311469	-1.850420808	1.17E-08	4.83E-08
ENSDARG00000019566	neurod	0.277115615	-1.851440089	3.08E-104	3.50E-102
ENSDARG00000089326	arhgap20	0.27701394	-1.851969516	2.87E-38	5.48E-37
ENSDARG00000079298	CABZ01081359.1	0.276085898	-1.856810896	1.58E-18	1.25E-17
ENSDARG00000077563	GJC1	0.276066309	-1.856913264	2.71E-12	1.46E-11
ENSDARG00000004721	mpp5b	0.275693268	-1.858864055	1.60E-06	5.46E-06
ENSDARG00000062056	elmod1	0.275660643	-1.85903479	1.90E-109	2.43E-107
ENSDARG00000074515	GRIK2	0.275658984	-1.859043474	3.76E-33	5.85E-32
ENSDARG00000053678	SLC4A3	0.275644426	-1.859119665	8.84E-18	6.72E-17
ENSDARG00000079779	radil	0.275242087	-1.861227008	2.28E-30	3.16E-29
ENSDARG00000069518	aqp3b	0.274680835	-1.86417184	2.59E-08	1.04E-07
ENSDARG00000052289	CYGB (2 of 3)	0.274555338	-1.864831133	2.66E-07	9.77E-07
ENSDARG00000059945	SV2A	0.274301502	-1.866165574	6.37E-147	1.55E-144
ENSDARG00000002300	p2rx2	0.274269019	-1.866336428	4.20E-09	1.79E-08

ENSDARG00000068242	cngb1a	0.274238296	-1.866498047	0.000427761	0.00110975
ENSDARG00000028469	RPS6KA2	0.273767832	-1.868975159	5.43E-10	2.48E-09
ENSDARG00000093269	si:ch73-62b13.1	0.27364841	-1.869604619	8.07E-08	3.08E-07
ENSDARG00000074285	si:ch73-335m24.2	0.273546803	-1.870140402	5.99E-07	2.13E-06
ENSDARG00000094132	igf1	0.273489641	-1.870441908	2.22E-16	1.55E-15
ENSDARG00000071905	pcdh2ab3	0.273199609	-1.871972675	7.02E-06	2.24E-05
ENSDARG00000075928	hivep3a	0.273135348	-1.872312061	1.67E-86	1.27E-84
ENSDARG00000018896	mmp24	0.273093732	-1.872531891	5.30E-121	8.46E-119
ENSDARG00000034907	paqr7b	0.273053617	-1.872743826	5.54E-52	1.75E-50
ENSDARG00000087224	si:ch73-380n15.2	0.272556877	-1.875370774	0.003326791	0.007624756
ENSDARG00000023203	si:ch211-147g22.1	0.271898059	-1.878862241	0.004292765	0.009670207
ENSDARG00000074707	FUT9 (12 of 16)	0.271322425	-1.8819198	4.58E-05	0.000134375
ENSDARG00000068428		0.271055353	-1.883340596	1.98E-17	1.47E-16
ENSDARG00000068551	zgc:153394	0.271017951	-1.883539682	3.34E-11	1.67E-10
ENSDARG00000068240	trim110	0.270378716	-1.886946505	4.90E-06	1.58E-05
ENSDARG00000074075	antxr1b	0.269803573	-1.890018641	1.29E-06	4.42E-06
ENSDARG00000034011	CABZ01072856.1	0.269794934	-1.890064837	2.76E-52	8.78E-51
ENSDARG00000019660	rxfp2a	0.269602583	-1.891093774	8.54E-06	2.70E-05
ENSDARG00000092920	si:ch211-106h4.12	0.268763999	-1.895588195	2.06E-10	9.71E-10
ENSDARG00000056742	crmp1	0.268474395	-1.897143593	6.56E-135	1.37E-132
ENSDARG00000094217	si:dkey-17e16.15	0.268337353	-1.897880201	1.13E-12	6.21E-12
ENSDARG00000038439	fabp10a	0.26809696	-1.899173232	2.07E-11	1.05E-10
ENSDARG00000001910	rorab	0.267815496	-1.900688657	8.05E-154	2.18E-151
ENSDARG00000075948	slco4a1	0.267646386	-1.901599923	8.76E-12	4.56E-11
ENSDARG00000033954	CABZ01020188.1	0.267642033	-1.901623389	1.52E-09	6.73E-09
ENSDARG00000095614	CACNA1E (2 of 2)	0.267140033	-1.904331904	5.88E-20	5.07E-19
ENSDARG00000012948	asb18	0.266879544	-1.905739368	0.000405569	0.001055935
ENSDARG00000031768	roraa	0.266861982	-1.905834306	9.64E-155	2.63E-152
ENSDARG00000070511	CR933820.2	0.266551402	-1.907514323	1.71E-27	2.10E-26
ENSDARG00000095002	TNNC2 (2 of 2)	0.266441986	-1.908106656	5.28E-23	5.31E-22

ENSDARG00000075546	CABZ01109624.1	0.266214422	-1.909339367	9.21E-27	1.10E-25
ENSDARG00000055175	c1qtnf6a	0.266153457	-1.909669792	1.12E-33	1.79E-32
ENSDARG00000037448	SLC2A13 (1 of 3)	0.265661625	-1.91233825	7.48E-05	0.00021435
ENSDARG00000059466	WASF3 (1 of 2)	0.265617836	-1.912576069	7.07E-21	6.39E-20
ENSDARG00000032865	pacsin1a	0.265410989	-1.913699988	1.04E-112	1.44E-110
ENSDARG00000076932	CT025934.1	0.26516428	-1.915041648	2.89E-24	3.08E-23
ENSDARG00000058353	CSMD1	0.265102941	-1.915375417	3.10E-12	1.66E-11
ENSDARG00000060025	nsmfa	0.26505224	-1.91565136	4.86E-16	3.33E-15
ENSDARG00000063133	SLC4A10 (2 of 2)	0.26500211	-1.915924246	7.87E-153	2.07E-150
ENSDARG00000074064	rem2	0.264621695	-1.917996748	2.21E-28	2.81E-27
ENSDARG00000019179	lrit1a	0.263700225	-1.923029291	6.15E-08	2.37E-07
ENSDARG00000092452	si:dkey-95p16.1	0.263599151	-1.923582373	8.63E-15	5.48E-14
ENSDARG00000093021	FAM163A (2 of 2)	0.263297852	-1.92523234	0.00049463	0.001272424
ENSDARG00000070863	cspg5b	0.262831237	-1.927791349	6.97E-145	1.65E-142
ENSDARG00000038974	si:ch73-112n4.1	0.261730173	-1.933847843	1.65E-133	3.33E-131
ENSDARG00000069117	kcnh5b	0.261510872	-1.935057168	1.88E-10	8.87E-10
ENSDARG00000087413	BEAN1	0.260806131	-1.938950308	3.50E-07	1.27E-06
ENSDARG00000021352	gria1a	0.260565822	-1.940280235	2.76E-92	2.44E-90
ENSDARG00000062821	slc6a15	0.26055999	-1.940312528	7.84E-115	1.12E-112
ENSDARG00000024004	cacna1da	0.259985026	-1.94349956	4.25E-78	2.70E-76
ENSDARG00000056651	gfra4	0.259973187	-1.943565262	5.88E-24	6.17E-23
ENSDARG00000019492	shbg	0.259266065	-1.947494707	4.57E-05	0.000133933
ENSDARG00000021150	valopa	0.258578456	-1.951326017	3.70E-08	1.46E-07
ENSDARG00000068858	il2rga	0.258356259	-1.952566258	2.79E-05	8.36E-05
ENSDARG00000031438	kcnj11l	0.257714332	-1.956155324	3.79E-107	4.62E-105
ENSDARG00000045944	slc6a1a	0.257345343	-1.95822242	9.78E-163	2.93E-160
ENSDARG00000075513	ccdc136b	0.256911375	-1.960657324	2.09E-18	1.64E-17
ENSDARG00000063568	SYT7 (1 of 2)	0.256876048	-1.960855717	1.07E-16	7.66E-16
ENSDARG00000087133	BX005472.1	0.2566313	-1.962230955	2.78E-07	1.02E-06
ENSDARG00000026796	grm1a	0.256116725	-1.965126628	9.77E-68	4.74E-66

ENSDARG00000017108	KCNA1A	0.256079446	-1.965336632	5.55E-05	0.000161239
ENSDARG00000086391	cald1	0.255938076	-1.966133299	1.08E-48	3.04E-47
ENSDARG00000028478	si:ch211-173n18.3	0.255803574	-1.966891676	5.67E-08	2.20E-07
ENSDARG00000095214	si:ch211-183d21.2	0.255618985	-1.967933107	1.44E-10	6.89E-10
ENSDARG00000089276	TMEM235 (1 of 2)	0.255502709	-1.968589505	0.000116706	0.000326117
ENSDARG00000054817	ppp1r14c	0.255427712	-1.969013042	8.50E-138	1.79E-135
ENSDARG00000006212	MYT1L (1 of 2)	0.255267441	-1.969918558	7.17E-61	2.90E-59
ENSDARG00000057209	lppr5a	0.255096049	-1.970887543	4.58E-14	2.78E-13
ENSDARG00000062712	si:dkey-236e20.7	0.25499501	-1.971459079	4.74E-09	2.01E-08
ENSDARG00000097285	ANPEP (7 of 7)	0.254894991	-1.97202507	5.23E-23	5.27E-22
ENSDARG00000037691	pcdh2ab2	0.254747866	-1.972858032	0.000141792	0.000391748
ENSDARG00000091173		0.254619007	-1.973587978	1.71E-22	1.68E-21
ENSDARG00000045205	LRTM1	0.254365197	-1.975026807	3.94E-05	0.000116443
ENSDARG00000062640	kcnh8	0.254333893	-1.975204362	6.24E-16	4.24E-15
ENSDARG00000008209	myt1la	0.253976282	-1.97723432	4.52E-165	1.41E-162
ENSDARG00000063224	nxph2a	0.253471234	-1.980106068	5.53E-46	1.43E-44
ENSDARG00000087188	nfil3-6	0.253206242	-1.981615127	4.75E-54	1.60E-52
ENSDARG00000005739	gpm6ba	0.253200519	-1.981647731	1.24E-159	3.63E-157
ENSDARG00000089972	CR735107.1	0.253141872	-1.981981935	2.76E-12	1.48E-11
ENSDARG00000092987	zmat4a	0.253022568	-1.982662025	1.38E-23	1.43E-22
ENSDARG00000097613	IGFN1 (4 of 4)	0.252749399	-1.984220431	2.05E-155	5.66E-153
ENSDARG00000010619	themis	0.252486191	-1.985723608	1.19E-06	4.10E-06
ENSDARG00000095094	fxyd6	0.252372074	-1.986375815	2.31E-38	4.43E-37
ENSDARG00000042803	rab3b	0.252318046	-1.9866847	3.37E-38	6.40E-37
ENSDARG00000054683	prdm8b	0.25226724	-1.986975228	2.10E-132	4.15E-130
ENSDARG00000020102	kctd16b	0.25189343	-1.989114601	4.91E-07	1.76E-06
ENSDARG00000045204	CACNA2D3 (1 of 2)	0.251879313	-1.989195456	3.18E-12	1.70E-11
ENSDARG00000093062	si:ch73-370c12.1	0.251779024	-1.98977	1.36E-06	4.66E-06
ENSDARG00000076220	sez6a	0.251718607	-1.990116233	1.88E-51	5.79E-50
ENSDARG00000086640		0.251545903	-1.9911064	4.67E-05	0.000136819

ENSDARG00000057338	gstp2	0.251256035	-1.992769844	3.87E-21	3.55E-20
ENSDARG00000021405	chd3	0.250830013	-1.995218111	1.06E-143	2.44E-141
ENSDARG00000053022	purg	0.250471501	-1.997281636	6.52E-35	1.09E-33
ENSDARG00000070919	cpne5	0.250313829	-1.9981901	7.69E-20	6.59E-19
ENSDARG00000060680	si:ch73-127m5.1	0.249800016	-2.001154526	1.17E-133	2.36E-131
ENSDARG00000059391	gyg1b	0.2497935	-2.001192159	4.07E-121	6.53E-119
ENSDARG00000042293	ca4b	0.249233618	-2.004429417	5.95E-08	2.30E-07
ENSDARG00000057661	aldoca	0.248958764	-2.006021295	1.38E-06	4.72E-06
ENSDARG00000006560	irf4a	0.248449151	-2.008977482	0.000365304	0.000956889
ENSDARG00000001971	cthrc1b	0.247918885	-2.012059922	7.94E-07	2.79E-06
ENSDARG00000097652	si:dkey-234i14.2	0.247336836	-2.015450975	3.18E-28	4.01E-27
ENSDARG00000075321	CABZ01071964.1	0.247145418	-2.016567933	2.20E-11	1.11E-10
ENSDARG00000089090	mmd2b	0.246643133	-2.019502975	3.80E-16	2.62E-15
ENSDARG00000091404	BX323836.1	0.245661769	-2.025254739	0.000917533	0.002274461
ENSDARG00000074415		0.245241056	-2.027727571	7.14E-17	5.18E-16
ENSDARG00000039901	SH3GL2 (2 of 2)	0.245239393	-2.027737356	1.40E-27	1.73E-26
ENSDARG00000067727	BCL11B (2 of 2)	0.24476055	-2.030557047	8.26E-119	1.29E-116
ENSDARG00000034420	irx6a	0.244684616	-2.031004695	6.29E-56	2.26E-54
ENSDARG00000090121	CABZ01083267.1	0.244160793	-2.03409654	0.00076748	0.001923643
ENSDARG00000056004	sprn	0.244104244	-2.034430715	2.18E-60	8.71E-59
ENSDARG00000018109	mafga	0.244031937	-2.034858127	1.47E-20	1.31E-19
ENSDARG00000011184	grk1b	0.24392033	-2.035518089	4.04E-09	1.72E-08
ENSDARG00000044632	myo7ab	0.242359377	-2.044780192	4.51E-48	1.25E-46
ENSDARG00000077854	BX119910.8	0.242068365	-2.046513543	3.35E-13	1.92E-12
ENSDARG00000097855	si:ch211-3o3.9	0.241237892	-2.051471564	1.51E-65	6.86E-64
ENSDARG00000060095	kcnb1	0.241095321	-2.05232444	4.43E-18	3.42E-17
ENSDARG00000056652	SLC22A7 (3 of 5)	0.240597453	-2.055306726	0.000531215	0.00136038
ENSDARG00000077096	fndc7	0.240583524	-2.055390247	1.51E-29	2.02E-28
ENSDARG00000060578		0.240472732	-2.056054781	5.54E-16	3.77E-15
ENSDARG00000056084	igsf21b	0.240403416	-2.056470701	1.41E-47	3.85E-46

ENSDARG00000035891	acana	0.240313541	-2.05701015	1.35E-176	4.55E-174
ENSDARG00000045560	mybpc1	0.239299094	-2.06311316	1.85E-93	1.67E-91
ENSDARG00000089233	CSGALNACT1 (2 of 2)	0.238804513	-2.066097991	7.12E-06	2.27E-05
ENSDARG00000022668	grapb	0.238776716	-2.066265935	9.49E-06	2.98E-05
ENSDARG00000053939	tgfa	0.238700743	-2.066725038	1.23E-22	1.22E-21
ENSDARG00000075115	PCDHGC4 (5 of 9)	0.238684685	-2.066822097	4.61E-128	8.24E-126
ENSDARG00000025089	loxl4	0.238366755	-2.068745059	2.73E-51	8.37E-50
ENSDARG00000086394	RNF166 (1 of 2)	0.238320581	-2.069024549	0.000537668	0.001375666
ENSDARG00000089255	si:ch211-286c4.6	0.237995356	-2.070994671	1.39E-18	1.10E-17
ENSDARG00000095347	si:dkeyp-72h1.1	0.237582989	-2.073496554	9.13E-25	9.97E-24
ENSDARG00000078187	slc12a5b	0.23736003	-2.07485108	4.55E-77	2.80E-75
ENSDARG00000038056	fgfbp2b	0.237262099	-2.075446437	2.06E-179	7.43E-177
ENSDARG00000092039	si:dkey-70p6.1	0.236621639	-2.079346083	2.28E-91	1.96E-89
ENSDARG00000074197	ADAM23 (2 of 2)	0.236445152	-2.080422533	6.51E-07	2.30E-06
ENSDARG00000042586	PCDH8 (2 of 2)	0.235974306	-2.083298316	5.62E-79	3.63E-77
ENSDARG00000004789	lrp1ba	0.235807324	-2.084319569	2.54E-38	4.85E-37
ENSDARG00000096207	SRR (2 of 2)	0.235486051	-2.086286492	5.62E-10	2.56E-09
ENSDARG00000077945	HEPACAM (2 of 2)	0.234906027	-2.089844365	0.001115509	0.002733974
ENSDARG00000004753	mylkb	0.234866813	-2.09008522	8.62E-43	2.00E-41
ENSDARG00000074348	PCDHGC4 (4 of 9)	0.23440427	-2.092929242	5.22E-96	5.12E-94
ENSDARG00000056805	PLCH2 (1 of 2)	0.234328215	-2.093397416	1.03E-20	9.24E-20
ENSDARG00000070011	ASTL (9 of 9)	0.234313657	-2.09348705	6.84E-08	2.63E-07
ENSDARG00000096599	si:ch211-160d14.9	0.234091015	-2.094858536	8.11E-05	0.000231337
ENSDARG00000089458	rp1l1	0.233775362	-2.096805205	6.26E-46	1.62E-44
ENSDARG00000097355	si:dkey-86k10.2	0.233584032	-2.097986439	3.80E-24	4.02E-23
ENSDARG00000092534	si:ch211-62a1.3	0.233529984	-2.098320296	1.03E-08	4.26E-08
ENSDARG00000070107	six7	0.233383342	-2.099226504	1.46E-152	3.79E-150
ENSDARG00000053724	ADCYAP1R1 (2 of 2)	0.233169225	-2.100550707	6.05E-102	6.56E-100
ENSDARG00000053987	htr1b	0.233065678	-2.101191528	5.61E-15	3.61E-14
ENSDARG00000063544	pip4k2ab	0.232196804	-2.106579977	1.05E-66	4.95E-65

ENSDARG00000010096	slc1a6	0.23214575	-2.106897226	1.12E-47	3.07E-46
ENSDARG00000067509	slc24a4b	0.231877843	-2.108563126	1.38E-08	5.64E-08
ENSDARG00000037646	rgs11	0.231629003	-2.110112188	3.20E-35	5.43E-34
ENSDARG00000077275	crispld1a	0.230913386	-2.114576288	1.03E-38	2.01E-37
ENSDARG00000027638	adra1ab	0.230025274	-2.120135711	3.94E-16	2.71E-15
ENSDARG00000037487	1-Mar	0.229271156	-2.12487323	8.28E-11	4.02E-10
ENSDARG00000067507	kctd8	0.228884137	-2.127310616	2.18E-19	1.82E-18
ENSDARG00000076310	FBXO41 (1 of 2)	0.228529208	-2.129549527	2.98E-45	7.56E-44
ENSDARG00000005221	desmb	0.228336805	-2.130764671	3.06E-24	3.25E-23
ENSDARG00000077504	si:ch211-103n10.5	0.228036036	-2.132666264	2.97E-13	1.71E-12
ENSDARG00000011989	crx	0.227692109	-2.134843802	5.33E-188	2.16E-185
ENSDARG00000087558		0.227683316	-2.134899517	2.98E-14	1.83E-13
ENSDARG00000001881	cacnb3a	0.227247982	-2.137660614	2.71E-15	1.78E-14
ENSDARG00000058248	si:dkeyp-77h1.4	0.227141317	-2.138337937	1.14E-42	2.62E-41
ENSDARG00000062754	cpne4	0.226866629	-2.140083681	2.14E-11	1.09E-10
ENSDARG00000088541	tpbgl	0.226059325	-2.145226664	2.67E-10	1.25E-09
ENSDARG00000060846	cabp7b	0.225916645	-2.146137527	3.04E-16	2.11E-15
ENSDARG00000029066	rhcgb	0.225829825	-2.146692065	2.09E-09	9.13E-09
ENSDARG00000021369	mc3r	0.225163668	-2.15095404	0.000488402	0.001257769
ENSDARG00000093357	NYAP2 (2 of 2)	0.225047213	-2.151700398	9.17E-127	1.60E-124
ENSDARG00000009782	myh11a	0.224423283	-2.155705741	3.76E-147	9.22E-145
ENSDARG00000088491	CABZ01103847.1	0.224304515	-2.156469432	3.15E-07	1.15E-06
ENSDARG00000069737	pou4f2	0.22361886	-2.160886226	1.42E-187	5.70E-185
ENSDARG00000042529	gnat2	0.223614353	-2.160915305	1.48E-93	1.35E-91
ENSDARG00000087114		0.222964562	-2.165113671	2.47E-08	9.91E-08
ENSDARG00000035997	zfpm2b	0.222465353	-2.16834743	6.83E-138	1.45E-135
ENSDARG00000007382	ubtd1a	0.222150667	-2.17038962	1.04E-29	1.41E-28
ENSDARG00000075815	slc12a5a	0.222040947	-2.171102344	9.47E-56	3.38E-54
ENSDARG00000019063	FAT1 (2 of 2)	0.222013543	-2.171280409	4.96E-103	5.53E-101
ENSDARG00000001976	si:ch211-13k12.1	0.219858562	-2.185352376	7.33E-36	1.28E-34

ENSDARG00000087568	CABZ01092745.1	0.219632641	-2.186835619	0.000181133	0.000492539
ENSDARG00000018935	GABRB2 (1 of 2)	0.219267099	-2.189238746	5.71E-09	2.41E-08
ENSDARG00000077761	nlgn4b	0.219123174	-2.190186025	1.91E-31	2.77E-30
ENSDARG00000074181	LRRC24 (1 of 2)	0.218945327	-2.191357438	4.09E-27	4.93E-26
ENSDARG00000068989	gabra1	0.218449377	-2.194629102	1.07E-95	1.03E-93
ENSDARG00000030749	PHKA1 (2 of 2)	0.218374126	-2.195126168	1.82E-50	5.45E-49
ENSDARG00000009372	diras1b	0.218024469	-2.197438035	1.43E-10	6.81E-10
ENSDARG00000097311	si:dkey-201i2.1	0.21801966	-2.197469856	5.73E-05	0.000166127
ENSDARG00000088680	HHLA2 (4 of 4)	0.218000863	-2.197594246	2.76E-11	1.39E-10
ENSDARG00000078527	lingo4b	0.217878759	-2.198402541	4.35E-79	2.82E-77
ENSDARG00000070710	si:dkeyp-41g9.6	0.217826521	-2.198748481	2.05E-05	6.24E-05
ENSDARG00000051892	KCNG4 (1 of 2)	0.217704694	-2.199555578	1.70E-06	5.77E-06
ENSDARG00000037159	oprd1b	0.217631152	-2.200043017	3.49E-10	1.62E-09
ENSDARG00000055154	slc8a4a	0.217551254	-2.200572761	1.18E-74	6.84E-73
ENSDARG00000074990	BEND4	0.216964204	-2.204471055	7.38E-08	2.83E-07
ENSDARG00000089310	gc	0.216963804	-2.204473719	9.44E-10	4.25E-09
ENSDARG00000053364	pax6l	0.216819346	-2.205434604	1.32E-66	6.21E-65
ENSDARG00000003533	col8a1b	0.216629502	-2.206698361	2.12E-16	1.49E-15
ENSDARG00000097875	si:ch211-170a17.1	0.216430405	-2.208024905	2.01E-16	1.41E-15
ENSDARG00000071394	foxq2	0.216356495	-2.208517665	1.15E-14	7.25E-14
ENSDARG00000024032	coch	0.216259867	-2.209162136	5.87E-17	4.28E-16
ENSDARG00000074570	FP016199.1	0.215975956	-2.211057385	1.28E-12	7.04E-12
ENSDARG00000054543	samsn1a	0.215614065	-2.213476801	5.42E-95	5.18E-93
ENSDARG00000094702	si:dkey-61n16.5	0.21522185	-2.21610354	1.06E-13	6.27E-13
ENSDARG00000091459		0.21490893	-2.218202662	6.63E-08	2.55E-07
ENSDARG00000044475	ompa	0.214549728	-2.220616021	2.50E-19	2.08E-18
ENSDARG00000015134	CAMKK1 (1 of 2)	0.214228355	-2.222778652	3.93E-07	1.42E-06
ENSDARG00000056248	wu:fb15e04	0.2138251	-2.225496882	2.67E-42	6.05E-41
ENSDARG00000003210	ANO2 (1 of 2)	0.213035325	-2.23083542	2.38E-100	2.51E-98
ENSDARG00000068370	ralgps1	0.212412492	-2.235059479	2.01E-37	3.70E-36

ENSDARG00000076660	FOXO6 (2 of 2)	0.212326611	-2.235642901	2.59E-12	1.40E-11
ENSDARG00000088218	CABZ01035080.1	0.212160987	-2.236768701	0.000185888	0.000504984
ENSDARG00000086931	CABZ01029822.1	0.211877584	-2.23869713	3.06E-26	3.57E-25
ENSDARG00000057052	dscaml1	0.211875171	-2.238713562	6.37E-205	2.97E-202
ENSDARG00000078134	crygm2f	0.211513644	-2.241177364	0.000121262	0.000338117
ENSDARG00000075026	mkxb	0.211431801	-2.241735706	7.58E-10	3.43E-09
ENSDARG00000076706	CABZ01038708.1	0.211275896	-2.242799912	0.000509993	0.00130981
ENSDARG00000088634	adcy1b	0.210852801	-2.245691907	2.81E-17	2.08E-16
ENSDARG00000017532	KIF25	0.210601487	-2.247412469	6.76E-21	6.12E-20
ENSDARG00000002696	gnb3b	0.210290546	-2.249544105	1.40E-75	8.31E-74
ENSDARG00000043349	MYL2 (1 of 2)	0.209707773	-2.253547756	2.73E-56	9.87E-55
ENSDARG00000031013	C1QL1 (2 of 2)	0.209688031	-2.253683582	0.000581007	0.001479623
ENSDARG00000076583	pcdh2aa1	0.209488902	-2.255054277	2.62E-13	1.51E-12
ENSDARG00000097986	si:zfos-411a11.3	0.208939659	-2.258841739	2.56E-32	3.84E-31
ENSDARG00000016491	aglb	0.208189675	-2.264029576	2.39E-179	8.49E-177
ENSDARG00000015592	c1ql2	0.207823237	-2.266571124	6.74E-06	2.15E-05
ENSDARG00000010555	pdha1b	0.207799597	-2.266735238	5.56E-07	1.98E-06
ENSDARG00000086969	CR774179.1	0.206801088	-2.273684321	2.73E-10	1.27E-09
ENSDARG00000095011	si:ch211-67n3.9	0.206234558	-2.277641992	4.47E-08	1.75E-07
ENSDARG00000056292	vsx1	0.206127869	-2.278388519	3.20E-209	1.52E-206
ENSDARG00000078868	tmem74b	0.205746531	-2.28105999	3.64E-23	3.70E-22
ENSDARG00000077023	pcdhb	0.205323884	-2.284026641	8.62E-11	4.18E-10
ENSDARG00000037286		0.20449641	-2.289852577	3.32E-32	4.96E-31
ENSDARG00000078132	si:dkey-7e14.7	0.203549227	-2.296550355	8.94E-24	9.33E-23
ENSDARG00000078514	CCDC50 (1 of 2)	0.203313734	-2.298220418	2.44E-06	8.17E-06
ENSDARG00000067815	LINGO3 (2 of 2)	0.203295904	-2.298346948	3.79E-10	1.75E-09
ENSDARG00000070543	grin2ab	0.20316531	-2.299274006	6.92E-89	5.51E-87
ENSDARG00000092985	si:ch1073-309n14.1	0.201634796	-2.310183471	2.31E-21	2.14E-20
ENSDARG00000020794	neurod6b	0.201159634	-2.313587259	3.33E-185	1.28E-182
ENSDARG00000093893	CCBL2 (3 of 4)	0.200758822	-2.316464706	0.000333132	0.000876577

ENSDARG00000077893	ELFN2 (2 of 2)	0.200590237	-2.317676705	4.50E-18	3.47E-17
ENSDARG00000045180	acta2	0.200266931	-2.320003877	7.50E-139	1.63E-136
ENSDARG00000090815	KCNJ10A	0.199819243	-2.323232568	1.49E-27	1.84E-26
ENSDARG00000035137	EHD2 (1 of 2)	0.199140651	-2.32814034	0.000164719	0.0004511
ENSDARG00000062357	fam19a1	0.199137633	-2.328162205	2.41E-21	2.23E-20
ENSDARG00000037507	kctd12b	0.199088459	-2.328518505	2.63E-17	1.95E-16
ENSDARG00000012504	rlbp1a	0.198670749	-2.331548619	2.16E-145	5.16E-143
ENSDARG00000056411	adipoqb	0.198337364	-2.333971605	8.18E-05	0.000233152
ENSDARG00000036186	mbpa	0.198315165	-2.334133091	2.99E-47	8.06E-46
ENSDARG00000094908	lingo4a	0.198178877	-2.335124894	3.87E-06	1.27E-05
ENSDARG00000004643	cdhr1a	0.197681597	-2.338749522	7.60E-39	1.49E-37
ENSDARG00000022951	pth2	0.197297063	-2.341558614	6.14E-05	0.000177251
ENSDARG00000041115	cnfn	0.196544172	-2.347074507	0.000166207	0.000454824
ENSDARG00000055577	chrm2a	0.196511311	-2.347315738	3.06E-27	3.71E-26
ENSDARG00000021004	c5	0.196229776	-2.349384122	1.48E-221	7.62E-219
ENSDARG00000093888	si:dkeyp-87e3.1	0.195989606	-2.351150946	1.34E-18	1.06E-17
ENSDARG00000086619	CR847851.2	0.195086028	-2.357817639	4.64E-10	2.13E-09
ENSDARG00000075058	gabra6a	0.19444169	-2.362590516	6.04E-06	1.94E-05
ENSDARG00000095553	lenep	0.194175534	-2.36456666	1.40E-08	5.73E-08
ENSDARG00000004445	GRM5 (1 of 2)	0.193435576	-2.370074941	4.31E-21	3.94E-20
ENSDARG00000043961	PCDH10	0.193149438	-2.372210617	8.07E-90	6.54E-88
ENSDARG00000090659	SGCZ	0.192627156	-2.376116993	5.22E-07	1.86E-06
ENSDARG00000061466	CPNE5 (1 of 2)	0.192353713	-2.378166417	4.07E-29	5.34E-28
ENSDARG00000021055	isl1l	0.192335032	-2.378306536	0.000682147	0.001722371
ENSDARG00000097422	si:ch73-145l22.4	0.191885175	-2.381684838	0.000187676	0.000509549
ENSDARG00000010420	ndrg1b	0.19179804	-2.382340115	4.16E-180	1.51E-177
ENSDARG00000035798	gngt1	0.191717884	-2.382943174	8.02E-46	2.07E-44
ENSDARG00000076852	CABZ01113374.2	0.191367721	-2.385580592	1.20E-25	1.36E-24
ENSDARG00000088839	BX248120.1	0.191000106	-2.388354652	1.26E-08	5.19E-08
ENSDARG00000094639	si:ch211-223g7.1	0.190701806	-2.390609591	0.000255283	0.000680961

ENSDARG00000017634	pdcb	0.190266451	-2.393906894	2.42E-49	7.02E-48
ENSDARG00000011983	zgc:136908	0.189546369	-2.399377277	1.33E-157	3.78E-155
ENSDARG00000041735	tmem237a	0.189021211	-2.403379957	2.20E-20	1.94E-19
ENSDARG00000037921	gng13b	0.188098485	-2.410439869	5.90E-231	3.28E-228
ENSDARG00000078350	lrrc24	0.187242545	-2.417019819	5.26E-05	0.000153128
ENSDARG00000056909		0.187106639	-2.418067347	1.08E-08	4.47E-08
ENSDARG00000073695	mamdc2b	0.185259272	-2.432382343	8.71E-15	5.53E-14
ENSDARG00000089059	CABZ01063077.1	0.184549727	-2.437918491	1.06E-05	3.32E-05
ENSDARG00000044600	CABZ01025393.1	0.184364563	-2.439366719	5.40E-13	3.05E-12
ENSDARG00000043953	BX119910.1	0.182657571	-2.452786543	1.26E-14	7.94E-14
ENSDARG00000027495	elovl4b	0.180117208	-2.472992072	3.96E-187	1.56E-184
ENSDARG00000077341	ppp1r14c	0.179338183	-2.479245413	4.13E-178	1.45E-175
ENSDARG00000043847	tmem244	0.178789284	-2.483667827	1.42E-18	1.13E-17
ENSDARG00000061576	colm	0.178730109	-2.484145406	4.92E-16	3.37E-15
ENSDARG00000059381	KCTD20	0.177431877	-2.494662872	1.29E-43	3.09E-42
ENSDARG00000098044	si:ch211-136a13.3	0.176938674	-2.498678678	1.79E-18	1.42E-17
ENSDARG00000014745	epd	0.176382555	-2.503220212	1.75E-62	7.39E-61
ENSDARG00000025728	grin1b	0.176240942	-2.504378986	3.27E-237	1.90E-234
ENSDARG00000079773	NOX5	0.176129776	-2.505289271	4.33E-07	1.56E-06
ENSDARG00000040928	CABZ01059391.1	0.175539251	-2.510134441	0.000217571	0.000585625
ENSDARG00000020602	grk7a	0.175346471	-2.511719703	8.16E-46	2.10E-44
ENSDARG00000013134	CABZ01071407.1	0.175164833	-2.513214934	5.35E-20	4.62E-19
ENSDARG00000052138	slc1a2a	0.174700122	-2.517047483	6.02E-06	1.93E-05
ENSDARG00000017398	slc44a5a	0.174648555	-2.517473388	1.85E-09	8.12E-09
ENSDARG00000027153	gabrr3a	0.174433389	-2.51925188	3.88E-20	3.38E-19
ENSDARG00000079078	hbaa1	0.174335286	-2.52006349	0.000160253	0.000439717
ENSDARG00000023886	cacna2d4b	0.173320682	-2.528484278	1.07E-35	1.85E-34
ENSDARG00000044935	hpdb	0.173223194	-2.529295982	3.69E-123	6.12E-121
ENSDARG00000091428		0.172759082	-2.533166542	1.65E-15	1.09E-14
ENSDARG00000087857	si:ch211-180a12.2	0.172547743	-2.534932491	1.59E-38	3.08E-37

ENSDARG00000029482	ush2a	0.171778218	-2.541380981	7.86E-108	9.71E-106
ENSDARG00000071488	CABZ01041804.1	0.171722244	-2.541851167	3.80E-100	3.99E-98
ENSDARG00000092574	si:ch211-198d23.1	0.171280655	-2.545565875	8.07E-07	2.83E-06
ENSDARG00000058410	acbd5b	0.171081471	-2.547244575	4.18E-08	1.64E-07
ENSDARG00000094708	sftpba	0.170845848	-2.54923291	1.26E-10	6.03E-10
ENSDARG00000074839	impg1b	0.169032801	-2.564624863	6.59E-35	1.10E-33
ENSDARG00000052016	cabp2a	0.168277966	-2.571081806	4.38E-12	2.32E-11
ENSDARG00000029898	CNGA1 (2 of 2)	0.168120099	-2.572435884	5.39E-10	2.46E-09
ENSDARG00000007080	rhcgl1	0.167987552	-2.573573761	8.46E-149	2.11E-146
ENSDARG00000074887	CT583723.1	0.167709748	-2.575961548	1.53E-09	6.76E-09
ENSDARG00000006503	CABZ01067175.1	0.167361111	-2.578963761	8.89E-07	3.10E-06
ENSDARG00000074119		0.166488697	-2.586503863	4.68E-08	1.83E-07
ENSDARG00000026248	slc1a7b	0.166361088	-2.58761007	1.54E-09	6.79E-09
ENSDARG00000077858	asip	0.166155745	-2.589391917	9.07E-06	2.86E-05
ENSDARG00000029321		0.166150571	-2.589436848	3.23E-07	1.18E-06
ENSDARG00000006206	pou4f3	0.165776483	-2.592688733	3.23E-27	3.91E-26
ENSDARG00000002771	SLC4A5 (1 of 2)	0.165544929	-2.594705275	2.84E-22	2.75E-21
ENSDARG00000089523	LRRC30 (2 of 2)	0.165363965	-2.596283209	7.89E-14	4.71E-13
ENSDARG00000088475	pcdh1gb9	0.164632796	-2.602676338	8.27E-10	3.73E-09
ENSDARG00000096701	si:dkey-21e13.3	0.164428094	-2.604471281	4.75E-35	7.98E-34
ENSDARG00000095865	si:zfos-44h1.1	0.163888673	-2.609211951	1.32E-05	4.09E-05
ENSDARG00000014233	sept8b	0.163716638	-2.61072715	3.96E-40	8.18E-39
ENSDARG00000021336	pax4	0.163631534	-2.61147729	1.69E-09	7.45E-09
ENSDARG00000014697	KCNJ10B	0.161518452	-2.630229107	3.26E-09	1.40E-08
ENSDARG00000078226	CDH12 (2 of 2)	0.161435968	-2.630966049	1.60E-34	2.62E-33
ENSDARG00000038293	zgc:103559	0.160540581	-2.638990071	6.62E-05	0.000190522
ENSDARG00000067585	TPPP	0.159825008	-2.64543493	1.32E-20	1.17E-19
ENSDARG00000078736	DOC2A	0.159759871	-2.646023025	3.07E-18	2.40E-17
ENSDARG00000071419	asb10	0.15932007	-2.65000008	4.85E-40	9.98E-39
ENSDARG00000078411	hspb15	0.158314299	-2.659136529	3.55E-27	4.29E-26

ENSDARG00000010680	gngt2a	0.157745195	-2.664332033	5.41E-58	2.03E-56
ENSDARG00000035437	MYH13 (3 of 11)	0.15759899	-2.665669802	7.54E-79	4.86E-77
ENSDARG00000011886	pdca	0.157588864	-2.6657625	1.76E-27	2.16E-26
ENSDARG00000018478	agxtb	0.156883361	-2.672235747	4.15E-100	4.33E-98
ENSDARG00000037587	SYNPR (1 of 2)	0.156635618	-2.674515785	1.46E-75	8.61E-74
ENSDARG00000091627	si:dkey-271j15.3	0.155904275	-2.681267605	4.54E-11	2.25E-10
ENSDARG00000094310	si:ch211-255g12.6	0.155554135	-2.684511349	2.54E-199	1.13E-196
ENSDARG00000062319	si:dkey-103g5.3	0.154798378	-2.691537743	3.98E-176	1.33E-173
ENSDARG00000043226	nfixa	0.154772715	-2.691776933	6.98E-111	9.18E-109
ENSDARG00000075865	CABZ01076351.1	0.154307333	-2.696121475	6.53E-15	4.18E-14
ENSDARG00000052664	MSLN (1 of 2)	0.152528694	-2.712847423	1.80E-20	1.60E-19
ENSDARG00000078755	lppr4b	0.152403246	-2.714034462	3.43E-24	3.64E-23
ENSDARG00000069469	khdrbs2	0.152182724	-2.716123503	1.66E-186	6.45E-184
ENSDARG00000051981	STX3 (2 of 2)	0.151837937	-2.7193958	3.00E-101	3.22E-99
ENSDARG00000014840	prph2b	0.151080464	-2.726610976	9.22E-29	1.20E-27
ENSDARG00000075929		0.15089233	-2.728408621	1.95E-05	5.95E-05
ENSDARG00000094605		0.150772773	-2.729552167	1.95E-05	5.93E-05
ENSDARG00000010124	sp5l	0.150445381	-2.732688282	9.48E-44	2.30E-42
ENSDARG00000075054	RASGRF2 (2 of 2)	0.148588114	-2.750609381	1.68E-47	4.58E-46
ENSDARG00000075384	enpp2	0.148256133	-2.753836308	1.57E-65	7.13E-64
ENSDARG00000090126		0.147754607	-2.75872498	3.77E-11	1.88E-10
ENSDARG00000039964	fgfbp2a	0.147552869	-2.76069612	2.40E-45	6.10E-44
ENSDARG00000026855	cacna2d4a	0.147112484	-2.765008417	1.07E-38	2.09E-37
ENSDARG00000096398	si:ch211-276a17.5	0.146997869	-2.766132851	3.13E-20	2.73E-19
ENSDARG00000003326	cacng5a	0.146515161	-2.770878133	2.06E-10	9.73E-10
ENSDARG00000060893	col8a2	0.144956194	-2.786311118	1.95E-288	1.63E-285
ENSDARG00000079519	si:ch211-69e5.1	0.144531927	-2.790539877	4.22E-08	1.66E-07
ENSDARG00000045139	ca7	0.143969612	-2.796163767	2.54E-25	2.84E-24
ENSDARG00000016412	agt	0.142686507	-2.80907918	2.36E-196	1.03E-193
ENSDARG00000045677	opn1sw1	0.140939929	-2.826847701	2.08E-253	1.37E-250

ENSDARG00000023683	cacna1f	0.140521278	-2.831139491	1.60E-62	6.77E-61
ENSDARG00000076644	kcnv2a	0.139293132	-2.843803964	3.88E-12	2.06E-11
ENSDARG00000059342	avil	0.139092745	-2.845880922	5.92E-07	2.10E-06
ENSDARG00000061000	KLHDC8A	0.138925086	-2.847620964	9.80E-35	1.62E-33
ENSDARG00000086756	RGS9BP	0.138792971	-2.848993591	1.41E-44	3.51E-43
ENSDARG00000052982	gabrr2a	0.137746216	-2.859915409	4.25E-11	2.11E-10
ENSDARG00000069830	CX55.5	0.137504719	-2.862446969	2.14E-06	7.20E-06
ENSDARG00000035163	tmem136b	0.137199462	-2.865653266	1.15E-06	3.97E-06
ENSDARG00000070597	prelp	0.136602444	-2.871944796	8.01E-163	2.42E-160
ENSDARG00000078965	chd5	0.135499622	-2.883639267	3.22E-25	3.58E-24
ENSDARG00000041595	ces3	0.135472469	-2.883928406	2.66E-301	2.36E-298
ENSDARG00000061149	nrn1lb	0.134866694	-2.890393986	2.13E-20	1.88E-19
ENSDARG00000079569	CR385022.1	0.134529645	-2.894003973	2.91E-16	2.03E-15
ENSDARG00000052700	si:dkey-162b23.4	0.134086686	-2.898762106	9.02E-248	5.82E-245
ENSDARG00000091745	CABZ01055591.1	0.133229013	-2.908019802	3.19E-07	1.16E-06
ENSDARG00000040123	zfpm2a	0.133150619	-2.908868964	1.66E-182	6.30E-180
ENSDARG00000037925	rgs9a	0.132877221	-2.911834286	1.78E-21	1.66E-20
ENSDARG00000092731	mhc1uca	0.131926344	-2.922195416	1.01E-13	6.00E-13
ENSDARG00000041382	si:dkey-283b15.2	0.13127457	-2.929340628	3.50E-118	5.38E-116
ENSDARG00000090089	CABZ01015106.1	0.131261041	-2.929489312	2.44E-34	3.97E-33
ENSDARG00000093600	omd	0.130555054	-2.937269784	4.31E-16	2.96E-15
ENSDARG00000075067	AIPL1	0.130224257	-2.94092989	1.89E-19	1.58E-18
ENSDARG00000026840	arhgap23b	0.128164984	-2.963925934	7.53E-43	1.75E-41
ENSDARG00000042387		0.127758537	-2.9685084	8.58E-11	4.16E-10
ENSDARG00000017274	opn1sw2	0.126941316	-2.977766388	7.10E-157	2.00E-154
ENSDARG00000057792	cx52.7	0.126904441	-2.978185541	2.75E-05	8.24E-05
ENSDARG00000076448	serpinf2a	0.125451618	-2.994797014	1.57E-16	1.11E-15
ENSDARG00000042845	oxt	0.125406293	-2.995318349	1.60E-12	8.75E-12
ENSDARG00000089439	TMEM132A	0.124608788	-3.004522272	1.70E-22	1.67E-21
ENSDARG00000061836	nfixb	0.12326595	-3.020153761	2.52E-121	4.06E-119

ENSDARG00000010933	CACNA1F (1 of 2)	0.1221924	-3.03277354	4.46E-104	5.05E-102
ENSDARG00000005841	tnni2a.2	0.121732566	-3.038212929	1.01E-168	3.25E-166
ENSDARG00000075227	GRM5 (2 of 2)	0.120977667	-3.047187356	1.05E-21	9.87E-21
ENSDARG00000087873	si:ch211-170n20.3	0.120785064	-3.049486026	3.95E-153	1.05E-150
ENSDARG00000068483	neto1	0.120567401	-3.052088205	2.23E-07	8.24E-07
ENSDARG00000053845	cbln14	0.120121475	-3.057433994	4.50E-08	1.76E-07
ENSDARG00000027424	slc25a3a	0.119299062	-3.067345395	1.96E-47	5.33E-46
ENSDARG00000001760	TNXB	0.116994437	-3.095488164	0	0
ENSDARG00000068474	si:dkey-94e7.2	0.113996622	-3.132937016	2.65E-30	3.67E-29
ENSDARG00000013051	C5H11orf53 (1 of 2)	0.113894099	-3.134235096	2.55E-05	7.65E-05
ENSDARG00000078438	DPP10	0.11356877	-3.138361932	2.40E-08	9.63E-08
ENSDARG00000009386	MPP4 (1 of 3)	0.113358255	-3.141038635	2.90E-23	2.96E-22
ENSDARG00000076978	pmchl	0.112870704	-3.147257011	8.78E-09	3.65E-08
ENSDARG00000076043	si:dkeyp-73d8.9	0.1122591	-3.155095694	7.41E-14	4.43E-13
ENSDARG00000076015	PLBD1 (2 of 2)	0.111480266	-3.165139748	5.47E-06	1.76E-05
ENSDARG00000079413	PPEF2	0.111388594	-3.166326589	5.95E-11	2.92E-10
ENSDARG00000059416	EGFLAM	0.111197384	-3.168805244	1.31E-40	2.76E-39
ENSDARG00000070666	rhol	0.111114024	-3.169887185	8.86E-09	3.68E-08
ENSDARG00000045811		0.11016516	-3.182260053	3.93E-19	3.23E-18
ENSDARG00000070931	si:ch211-232m10.6	0.109994417	-3.184497798	2.20E-10	1.03E-09
ENSDARG00000038018	prph2a	0.108727144	-3.201215941	3.89E-45	9.83E-44
ENSDARG00000096558	si:ch211-80j13.1	0.108457415	-3.204799403	9.85E-14	5.84E-13
ENSDARG00000093318	si:dkey-57a22.15	0.107737177	-3.214411933	5.54E-111	7.35E-109
ENSDARG00000060792	si:dkey-97i18.6	0.107625218	-3.215911939	2.88E-06	9.54E-06
ENSDARG00000018530	mapkapk2b	0.107308871	-3.22015875	1.22E-23	1.27E-22
ENSDARG00000089411	TMEM136 (3 of 3)	0.106441923	-3.231861616	4.51E-09	1.91E-08
ENSDARG00000087663	PRR35	0.105939032	-3.238693869	8.61E-87	6.61E-85
ENSDARG00000092792	zgc:92658	0.105771029	-3.240983565	3.23E-22	3.12E-21
ENSDARG00000086030	PCBP3 (2 of 2)	0.105248939	-3.248122404	3.48E-292	2.99E-289
ENSDARG00000012126	zgc:109965	0.105090156	-3.250300562	2.97E-131	5.70E-129

ENSDARG00000039602	atoh1c	0.10442765	-3.259424337	1.11E-07	4.19E-07
ENSDARG00000068088	tcnl	0.1039776	-3.26565534	8.53E-47	2.27E-45
ENSDARG00000007788	atp2b1b	0.102712534	-3.28331585	4.81E-267	3.69E-264
ENSDARG00000063293	neto2b	0.102437481	-3.287184416	2.01E-11	1.02E-10
ENSDARG00000057790	ankrd6a	0.102423108	-3.287386845	8.40E-26	9.58E-25
ENSDARG00000044199	gnat1	0.100328644	-3.317194538	1.48E-121	2.40E-119
ENSDARG00000014805	fhl5	0.098833908	-3.338850108	5.01E-16	3.42E-15
ENSDARG00000059978	cplx4a	0.098557277	-3.34289379	3.83E-65	1.73E-63
ENSDARG00000016480	slc17a7	0.098355017	-3.34585755	5.69E-45	1.43E-43
ENSDARG00000023181	pcp4l1	0.09794762	-3.351845753	6.08E-98	6.15E-96
ENSDARG00000044861	opn1lw2	0.097657501	-3.356125332	0	0
ENSDARG00000078210	tulp1b	0.097273306	-3.361812232	1.80E-76	1.10E-74
ENSDARG00000075986	RIC3 (1 of 2)	0.096843079	-3.368207248	1.40E-18	1.11E-17
ENSDARG00000093467	MYH13 (9 of 11)	0.09678735	-3.369037686	5.20E-230	2.84E-227
ENSDARG00000040274	SCAMP5 (2 of 2)	0.096111664	-3.37914467	3.35E-70	1.72E-68
ENSDARG00000040008	neurod6a	0.093684032	-3.416053028	2.49E-164	7.69E-162
ENSDARG00000043466	kcnk3	0.093424573	-3.42005412	5.00E-06	1.62E-05
ENSDARG00000062906	kcnv2b	0.093400008	-3.42043351	2.23E-24	2.39E-23
ENSDARG00000070652	si:ch211-134a4.1	0.092153623	-3.4398153	1.45E-16	1.03E-15
ENSDARG00000036140		0.091955396	-3.442921962	3.83E-102	4.17E-100
ENSDARG00000052223	rcvrna	0.091950209	-3.443003341	1.82E-149	4.57E-147
ENSDARG00000028846	arl3l1	0.091893533	-3.44389286	2.37E-86	1.80E-84
ENSDARG00000034940	slc1a7a	0.090336509	-3.468547029	1.31E-29	1.76E-28
ENSDARG00000089997	gngt2b	0.089066943	-3.488966115	2.70E-262	2.02E-259
ENSDARG00000078272	CABP7 (2 of 2)	0.087957933	-3.507042486	1.05E-13	6.19E-13
ENSDARG00000087082	CNGB3 (2 of 2)	0.087838153	-3.509008464	3.17E-36	5.59E-35
ENSDARG00000053207	MYH13 (5 of 11)	0.087500711	-3.514561446	3.27E-237	1.90E-234
ENSDARG00000070726	cnga3a	0.087466964	-3.515117963	9.69E-67	4.60E-65
ENSDARG00000070092	KCNV1	0.086601343	-3.529466791	7.33E-13	4.09E-12
ENSDARG00000092142	si:ch211-136a13.2	0.085689031	-3.544745653	1.08E-16	7.74E-16

ENSDARG00000039444	FAIM2	0.084706088	-3.561390524	5.48E-76	3.30E-74
ENSDARG00000088751	IMPG2	0.084299543	-3.568331378	1.12E-05	3.50E-05
ENSDARG00000095831	si:ch211-175f12.2	0.084170677	-3.570538473	8.95E-23	8.89E-22
ENSDARG00000034007	prom1b	0.083698018	-3.578662732	2.15E-306	1.97E-303
ENSDARG00000095217	MYH13 (10 of 11)	0.083638261	-3.579693128	9.64E-105	1.10E-102
ENSDARG00000059824	soat2	0.083411434	-3.583611031	1.06E-132	2.13E-130
ENSDARG00000006467	pcdh8	0.081241797	-3.621634041	2.24E-237	1.36E-234
ENSDARG00000010958	glmnb	0.080551877	-3.63393799	5.15E-77	3.16E-75
ENSDARG00000012125	cnga1	0.080289273	-3.63864894	3.01E-16	2.09E-15
ENSDARG00000075295	tulp1a	0.079460405	-3.653620041	1.37E-134	2.84E-132
ENSDARG00000056511	arr3a	0.078973158	-3.662493805	0	0
ENSDARG00000030980	csrp1b	0.078454218	-3.672005167	1.35E-15	9.03E-15
ENSDARG00000054797	RDH13 (3 of 3)	0.078216731	-3.676378943	1.01E-96	1.00E-94
ENSDARG00000095751	C20H6orf58 (2 of 2)	0.076686449	-3.704884521	1.58E-07	5.90E-07
ENSDARG00000012610	saga	0.07577605	-3.722114256	1.86E-150	4.76E-148
ENSDARG00000040627	GRIK1 (1 of 2)	0.075105421	-3.734939146	8.44E-96	8.21E-94
ENSDARG00000044280	opn1mw2	0.074668703	-3.743352523	2.00E-26	2.36E-25
ENSDARG00000037337	cnrip1b	0.07211794	-3.793498003	2.59E-97	2.61E-95
ENSDARG00000034930	cx52.6	0.072070372	-3.794449902	1.95E-12	1.06E-11
ENSDARG00000038742	RBP1	0.069718441	-3.842315875	3.99E-08	1.57E-07
ENSDARG00000038378	sagb	0.069259635	-3.851841405	9.72E-181	3.59E-178
ENSDARG00000095802	C20H2orf71	0.069147346	-3.854182309	2.57E-57	9.49E-56
ENSDARG00000075837	si:dkey-1d7.3	0.069113936	-3.854879556	4.78E-63	2.04E-61
ENSDARG00000038770	zgc:103625	0.068940093	-3.858512935	2.35E-68	1.16E-66
ENSDARG00000045592	tnni2a.1	0.066213235	-3.916736581	9.75E-90	7.87E-88
ENSDARG00000076055	RPGRIP1	0.065822981	-3.925264819	9.35E-128	1.66E-125
ENSDARG00000011640	syt5b	0.064462284	-3.955400887	0	0
ENSDARG00000076872	MPP4 (3 of 3)	0.064051879	-3.964615307	3.80E-43	8.96E-42
ENSDARG00000020814	faimb	0.064010398	-3.965549917	4.92E-32	7.29E-31
ENSDARG00000002576	cabp5a	0.063982129	-3.966187202	2.43E-07	8.96E-07

ENSDARG00000042526	sebox	0.063568538	-3.975543285	4.29E-12	2.27E-11
ENSDARG00000026926	rom1b	0.062913373	-3.990489488	1.37E-57	5.13E-56
ENSDARG00000019902	rcv1	0.062089824	-4.009499344	0	0
ENSDARG00000010602	zgc:112294	0.060787892	-4.040072206	3.20E-42	7.20E-41
ENSDARG00000079995	OVOL3	0.060206435	-4.053938496	1.72E-07	6.40E-07
ENSDARG00000078052	emilin3a	0.059436239	-4.072513367	0	0
ENSDARG00000069827	crygm2d11	0.058741483	-4.089476503	0	0
ENSDARG00000078440	ccdc88aa	0.058561523	-4.093903128	6.10E-258	4.33E-255
ENSDARG00000069017	elnb	0.05755146	-4.119003666	4.25E-196	1.83E-193
ENSDARG00000016854	neurod2	0.057375294	-4.123426553	2.15E-77	1.33E-75
ENSDARG00000052575	arl13a	0.055444184	-4.172820068	4.73E-107	5.74E-105
ENSDARG00000013393	guca1b	0.054999698	-4.184432485	2.79E-06	9.26E-06
ENSDARG00000077875	slc2a5	0.05320888	-4.23218916	2.88E-40	5.98E-39
ENSDARG00000040948	olig1	0.052974288	-4.238563892	1.24E-37	2.30E-36
ENSDARG00000088638	si:dkey-188p4.1	0.052791029	-4.243563415	4.49E-09	1.91E-08
ENSDARG00000089270	FP102168.1	0.052749804	-4.244690444	1.39E-06	4.75E-06
ENSDARG00000030626	lrit2	0.052600142	-4.248789508	1.04E-14	6.57E-14
ENSDARG00000062661	abca4b	0.052596824	-4.248880492	1.13E-192	4.72E-190
ENSDARG00000095328	si:ch211-191i18.4	0.051293568	-4.285078267	9.48E-07	3.30E-06
ENSDARG00000037656	si:ch1073-417l11.1	0.050803353	-4.298932462	3.82E-50	1.14E-48
ENSDARG00000062902	TULP2	0.048090688	-4.37809861	2.20E-94	2.09E-92
ENSDARG00000052057	PCOLCE (1 of 2)	0.048056917	-4.379112094	6.69E-117	1.01E-114
ENSDARG00000043820	nme2a	0.047465733	-4.396969842	5.67E-57	2.07E-55
ENSDARG00000041205	slc6a1l	0.047431866	-4.397999554	1.07E-10	5.17E-10
ENSDARG00000007024	uox	0.046511042	-4.426282925	3.73E-83	2.67E-81
ENSDARG00000090722	C20H6orf58 (1 of 2)	0.046200605	-4.435944459	3.26E-181	1.22E-178
ENSDARG00000001993	myhb	0.045147367	-4.469214325	0	0
ENSDARG00000037588	bhlhe23	0.044872086	-4.478037948	1.74E-256	1.20E-253
ENSDARG00000057427	sv2ba	0.044429698	-4.49233185	1.04E-261	7.61E-259
ENSDARG00000019782		0.044135119	-4.501929112	4.17E-32	6.21E-31

ENSDARG00000021345	prph2l	0.044118472	-4.502473367	5.39E-60	2.14E-58
ENSDARG00000055053	CR558302.3	0.043330703	-4.528466559	6.33E-54	2.12E-52
ENSDARG00000069615	ckmt2a	0.043081841	-4.536776297	0	0
ENSDARG00000005776	guk1b	0.041556533	-4.588780889	2.30E-124	3.86E-122
ENSDARG00000092269	si:dkeyp-41f9.4	0.040578338	-4.623146398	6.43E-66	2.96E-64
ENSDARG00000058103	glra4b	0.040415118	-4.628961146	8.42E-30	1.14E-28
ENSDARG00000030750	gabrr3b	0.039847975	-4.649349765	8.00E-28	9.98E-27
ENSDARG00000030215	matn1	0.039710841	-4.654323257	0	0
ENSDARG00000076565	GPR151	0.039496456	-4.66213299	2.78E-16	1.93E-15
ENSDARG00000002193	rho	0.039299013	-4.669363097	0	0
ENSDARG00000086360	RP1 (2 of 2)	0.038618808	-4.694552553	2.38E-86	1.80E-84
ENSDARG00000034628	slc1a8a	0.038166951	-4.71153224	1.47E-91	1.28E-89
ENSDARG00000003317	si:dkeyp-35f12.3	0.035818155	-4.803165162	1.67E-47	4.56E-46
ENSDARG00000089063	lactbl1a	0.03444861	-4.859410408	1.97E-64	8.68E-63
ENSDARG00000038894	tmx3	0.031037389	-5.00984901	3.01E-141	6.62E-139
ENSDARG00000061101	snx19a	0.026527528	-5.236365964	1.10E-75	6.55E-74
ENSDARG00000004358	gnb3a	0.026011434	-5.264710274	0	0
ENSDARG00000041162	GRM1 (2 of 2)	0.025408959	-5.298518893	3.24E-14	1.98E-13
ENSDARG00000094594	si:dkey-8k3.1	0.025374822	-5.300458501	5.01E-08	1.95E-07
ENSDARG00000011113	chrna10a	0.024272441	-5.364536973	6.43E-15	4.12E-14
ENSDARG00000088025		0.023448109	-5.414384615	1.09E-08	4.52E-08
ENSDARG00000019752	rom1a	0.02178777	-5.520337629	4.76E-69	2.37E-67
ENSDARG00000060017	CLEC3A	0.021443757	-5.543298474	4.60E-45	1.16E-43
ENSDARG00000063519	PLEKHB1 (1 of 2)	0.021329005	-5.55103955	1.57E-09	6.92E-09
ENSDARG00000076643	GPR179 (1 of 2)	0.019723315	-5.663954157	3.55E-09	1.52E-08
ENSDARG00000027236	rs1a	0.016653928	-5.907993669	0	0
ENSDARG00000096756	si:ch211-196c10.13	0.010659574	-6.55170642	1.94E-52	6.18E-51
ENSDARG00000079443		0.006914078	-7.176247449	7.06E-31	1.00E-29
